# Supplementary material for: One-step syntheses of diaza-dioxa-fenestranes via the sequential (3 + 2) cycloadditions of linear precursors and their structural analyses
Source: Nat Commun. 2024 Jul 19;15:6087. doi: 10.1038/s41467-024-49935-1 (PMC11271617; doi:10.1038/s41467-024-49935-1)
Supplement: Supplementary file 1 — Supplementary Information [file 41467_2024_49935_MOESM1_ESM.pdf]

Supplementary Information

One-step syntheses of diaza-dioxa-fenestranes via the sequential (3+2) cycloadditions of linear precursors and their structural analyses

Shinichiro Fuse,*[1] Hiroki Ishikawa,[1] Hiroshi Kitamura,[1] Hisashi Masui,[1] and Takashi Takahashi[2]

[1]Graduate School of Pharmaceutical Sciences, Nagoya University, Nagoya, 464-8601, Japan
[2]Graduate School of Infection Control Sciences, Kitasato University, Tokyo 108-8641, Japan

Table of Contents

1. General techniques 2
2. Overview of syntheses 3
2.1. Overview of preparation of building blocks S2 and S7 3
2.2. Overview of preparation of building block S11 4
2.3. Overview of preparation of aldehydes 1 and oxymes 4 and their sequential cycloadditions 5
2.4. Overview of derivatizations of diaza-dioxa-fenestranes 2g, 2a, and 5a 6
3. Preparation of substrates for intramolecular cycloaddition 6
3.1. Preparation of alkyne unit 6
3.2. Preparation of aldehyde 12
3.3. Preparation of cyclization precursors 14
4. Preparation of hydroxylamine analogs 28
5. Optimal conditions for (3+2)/(3+2) cycloaddition of nitrone 37
6. Optimal conditions for (3+2)/(3+2) cycloaddition of nitrile oxide 37
7. Optimization of reaction conditions 38
8. General procedure for cycloaddition via nitrone 40
9. General procedure for cycloaddition via nitrile oxide 53
10. Derivatization of oxa-aza fenestranes 56
11. X-ray Crystallographic Data 63
12. DFT calculation 78
13. NMR spectra 85
14. References 180

1. General techniques

NMR spectra were recorded on a JEOL-ECS400 (400 MHz for ^{1}H, 100 MHz for ^{13}C) or JEOL-ECZ400 (400 MHz for ^{1}H, 100 MHz for ^{13}C, 376 MHz for ^{19}F) instrument in the indicated solvent. Chemical shifts are reported in units of parts per million (ppm) relative to tetramethylsilane (0.00 ppm) or CD_3OD (3.31 ppm) for ^{1}H NMR and $CDCl_3$ (77.16 ppm) or CD_3OD (49.00 ppm) for ^{13}C NMR and α,α,α-trifluorotoluene (-63.72 ppm) for ^{19}F NMR. Multiplicities were reported by using the following abbreviations: s; singlet, d; doublet, t; triplet, q; quartet, m; multiplet, br; broad, *J*; coupling constants in Hertz (Hz). IR spectra were recorded on a JASCO FT/IR-4100 Fourier Transform Infrared Spectrophotometer. Only the strongest and/or structurally important peaks were reported as the IR data given in cm^{-1}. High resolution mass spectra (HRMS) were obtained on a Bruker Daltonics Compact in electrospray ionization (ESI) method. Gel permeation chromatography (GPC) for purification was performed on Japan Analytical Industry Model LaboACE LC-5060 (recycling preparative HPLC) on a Japan Analytical Industry Model UV-254 LA ultra violet detector and RI-700 LA refractive index detector with a polystyrene gel column (JAIGEL-2HR, 20 mm × 600 mm), using chloroform as solvent (10 mL/min). Column chromatography was performed on Silica Gel PSQ 60B purchased from Fuji Silysia Chemical LTD. Reactions were monitored by thin-layer chromatography carried out on 0.25 mm E. Merck silica gel plates (60F-254) with UV light, visualized by ethanolic *p*-anisaldehyde containing acetic acid and H_2SO_4, aqueous $KMnO_4$ containing potassium carbonate and sodium hydroxide, and aqueous phosphomolybdic acid containing H_2SO_4. The low-temperature reactions were performed using Techno Sigma UCR-150N.

THF, diethyl ether, CH_2Cl_2, and 1,4-dioxane were dried by a Nikko Hansen Glasss Contour MINI. Diisopropylamine was purchased from Tokyo Chemical Industry, distilled from potassium hydroxide under argon, and stored over molecular sieves 4A. Other solvents and reagents were purchased from commercial suppliers (Alfa Aesar, BLD Pharmatech, FUJIFILM Wako Pure Chemical, Kanto Chemical, Sigma-Aldrich, and Tokyo Chemical Industry) and used without further purification.

2. Overview of syntheses

2.1. Overview of preparation of building blocks S2 and S7

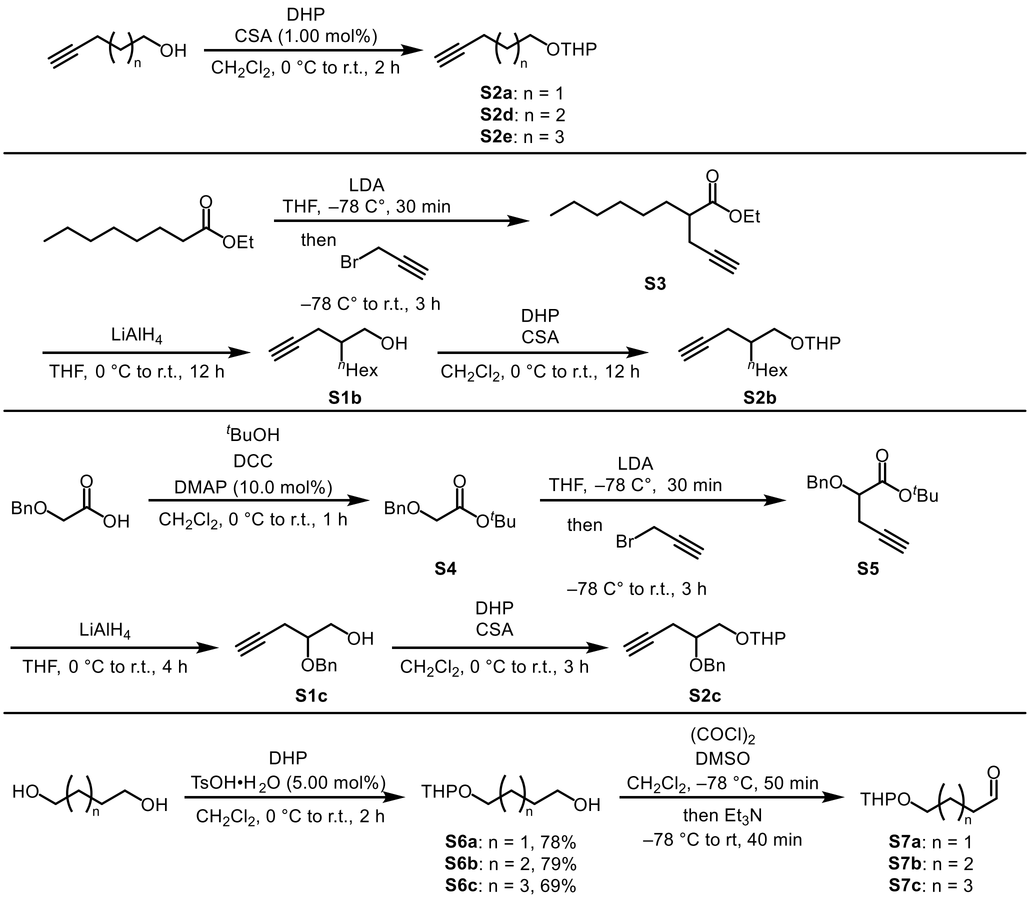

Supplementary Fig. 1 Preparation of building blocks S2 and S7.

2.2. Overview of preparation of building blocks S11

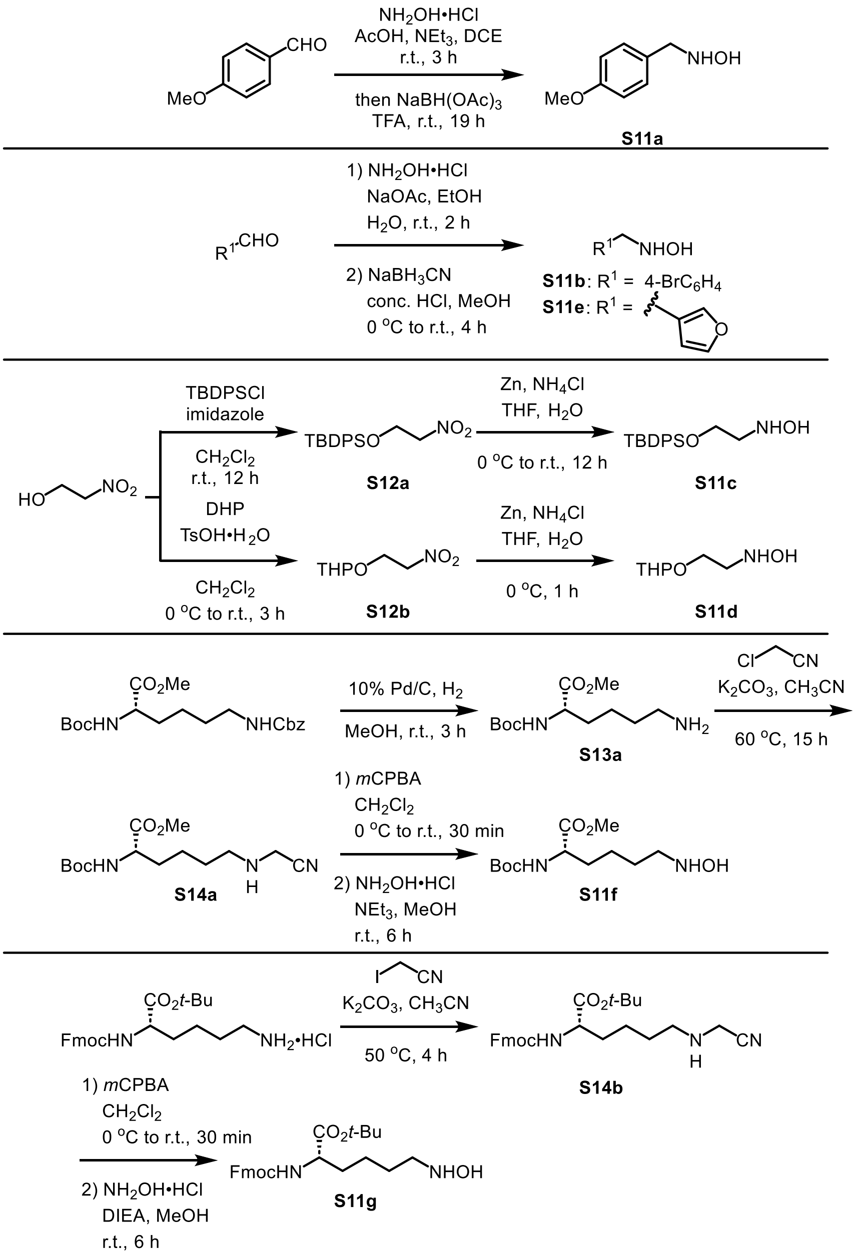

Supplementary Fig. 2 Preparation of building blocks S11.

2.3. Overview of preparation of aldehydes 1 and oxymes 4 and their sequential cycloadditions

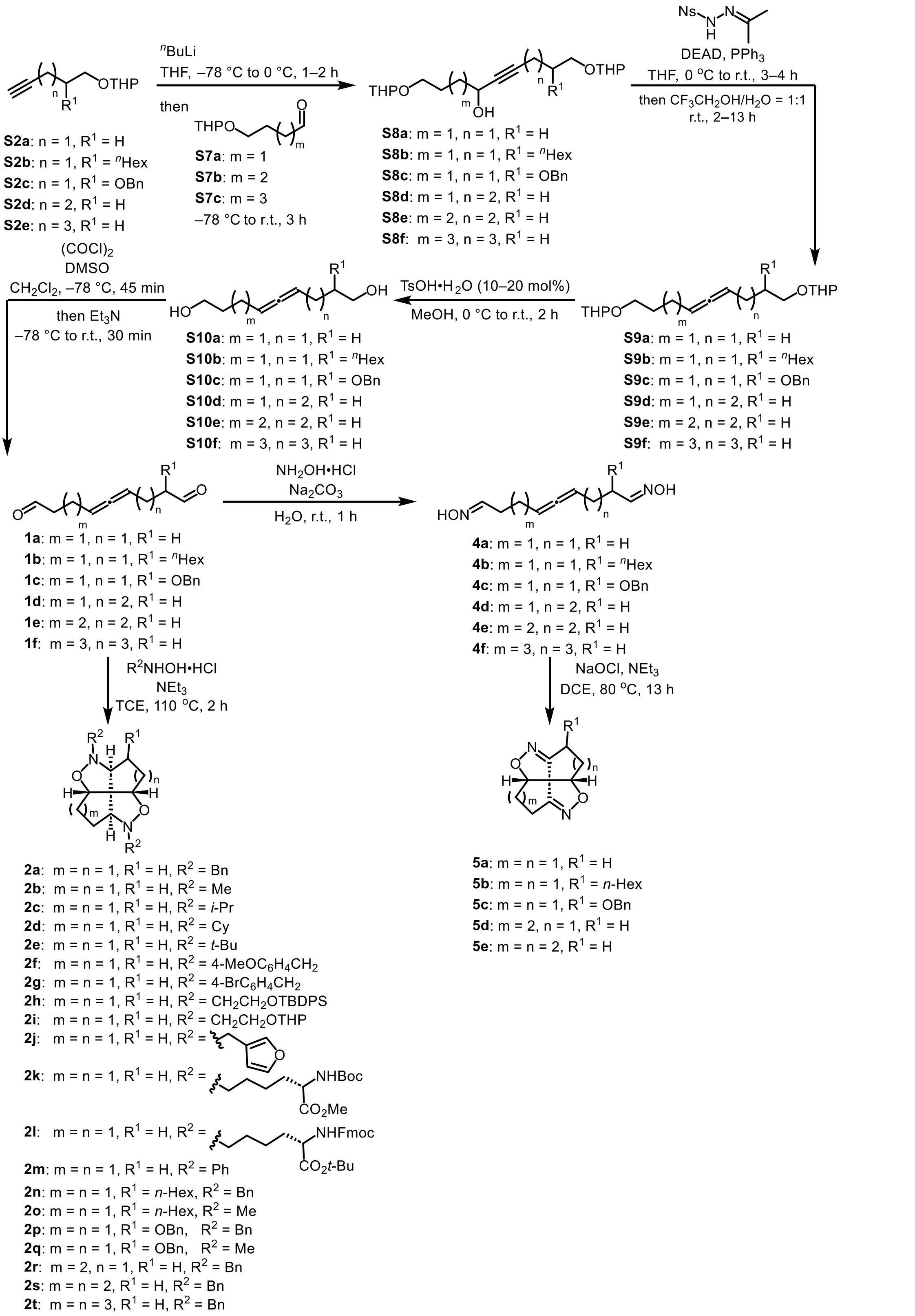

Supplementary Fig. 3 Preparation of aldehydes **1** and oxymes **4** and their cycloadditions.

2.4. Overview of derivatizations of diaza-dioxa-fenestranes 2g, 2a, and 5a

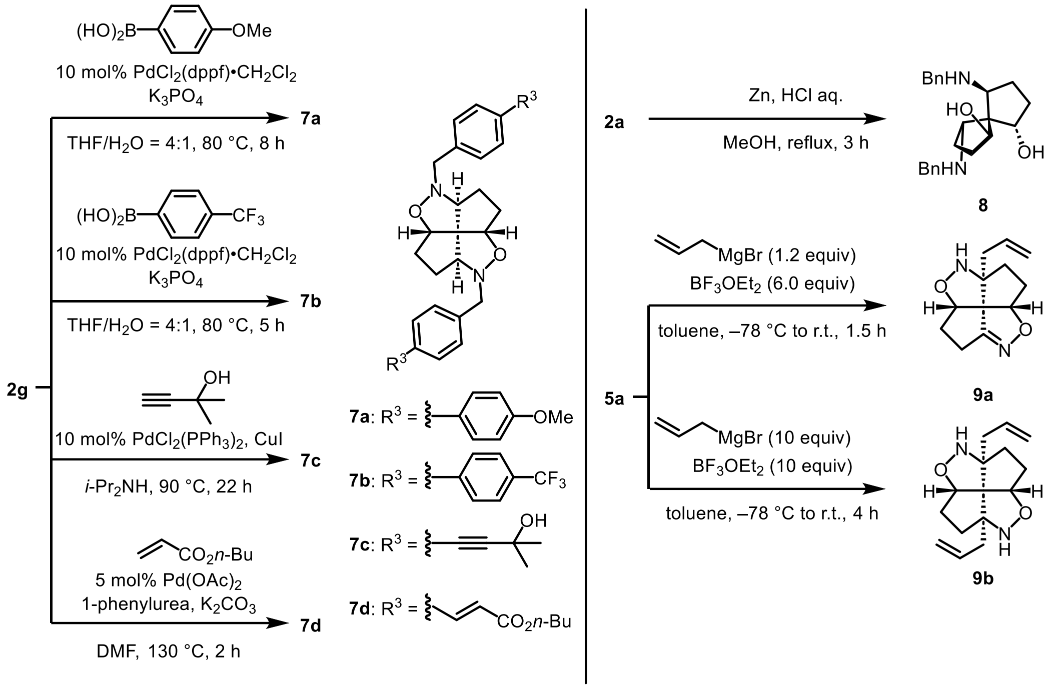

Supplementary Fig. 4 derivatizations of diaza-dioxa-fenestranes 2g, 2a, and 5a.

3. Preparation of substrates for intramolecular cycloaddition

3.1. Preparation of alkyne unit

General procedure A for the THP-protection of alkynyl alcohol S1

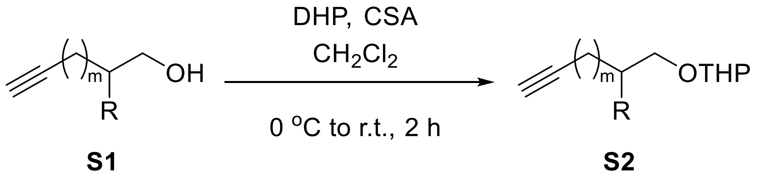

To a stirred solution of alcohol **S1** (1.00 equiv.) in anhydrous CH_2Cl_2 (0.500 M) were added 3,4-dihydro-2*H*-pyran (1.10 equiv.) and (+)-10-camphorsulfonic acid (1.00 mol%) at 0 °C under argon. After the resultant mixture was stirred at room temperature for 2 h, the reaction was quenched with sat. $NaHCO_3$ aq. and the aqueous layer was extracted with CH_2Cl_2 three times. The combined organic layer was washed with brine, dried over Na_2SO_4, filtered, and concentrated under reduced pressure. The residue was purified by column chromatography on silica gel to give the desired acetal **S2**.

2-(Pent-4-yn-1-yloxy)tetrahydro-2*H*-pyran (S2a)

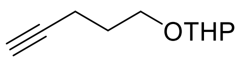

According to the **general procedure A** using 4-pentyn-1-ol (23.1 mL, 250 mmol, 1.00 equiv.), the crude product was purified by column chromatography on silica gel (hexane/ethyl acetate = 20 : 1) to give 2-(pent-4-yn-1-yloxy)tetrahydro-2*H*-pyran (**S2a**) (40.0 g, 230 mmol, 92%) as a colorless oil.
^{1}H NMR (400 MHz, $CDCl_3$): δ 4.60 (t, *J* = 3.4 Hz, 1H), 3.90-3.80 (m, 2H), 3.52-3.46 (m, 2H), 2.34-2.29 (m, 2H), 1.95-1.51 (m, 9H); ^{13}C NMR (100 MHz, $CDCl_3$): δ 99.0, 84.1, 68.6, 65.9, 62.4, 30.8, 28.8, 25.6, 19.7, 15.5.
^{1}H and ^{13}C NMR spectra were well consistent with those in the previous report[1].

Ethyl 2-(prop-2-yn-1-yl)octanoate (S3)

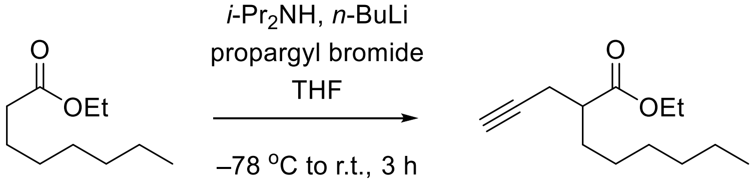

S3

Compound **S3** was prepared according to the modified procedure of Zhang et al[2].

To a solution of anhydrous diisopropylamine (8.43 mL, 60.0 mmol, 1.20 equiv.) was added *n*-BuLi (1.60 M in hexane, 34.4 mL, 55.0 mmol, 1.10 equiv.) at –78 °C under argon. To the reaction mixture was added dropwise over 50 min a solution of ethyl *n*-octanoate (9.90 mL, 50.0 mmol, 1.00 equiv.) in anhydrous THF (50 mL) at –78 °C. After the resultant mixture was stirred at the same temperature for 30 min, a solution of propargyl bromide (5.65 mL, 75.0 mmol, 1.50 equiv.) in anhydrous THF (25.0 mL) was added dropwise at the same temperature. After the resultant mixture was stirred at room temperature for 3 h, the reaction was quenched with sat. NH_4Cl aq. and the aqueous layer was extracted with hexane/ethyl acetate (1 : 1) three times. The combined organic layer was washed with brine, dried over Na_2SO_4, filtered, and concentrated under reduced pressure. The residue was purified by column chromatography on silica gel (hexane/ethyl acetate = 50 : 1) to give ethyl 2-(prop-2-yn-1-yl)octanoate (**S3**) (7.48 g, 35.6 mmol, 71%) as a yellow oil.
IR (neat): 3313, 2956, 2929, 2859, 2121, 1736, 1466, 1378, 1176, 637 cm^{-1}; ^{1}H NMR (400 MHz, $CDCl_3$): δ 4.17 (q, *J* = 7.2 Hz, 2H), 2.59-2.46 (m, 2H), 2.41-2.35 (m, 1H), 1.98 (t, *J* = 2.5 Hz, 1H), 1.73-1.59 (m, 2H), 1.29-1.25 (m, 11H), 0.88 (t, *J* = 6.9 Hz, 3H); ^{13}C NMR (100 MHz, $CDCl_3$): δ 174.7, 81.8, 69.8, 60.6, 44.7, 31.8, 31.5, 29.2, 27.0, 22.7, 21.3, 14.4, 14.2; HRMS (ESI): calcd. for ($[C_{13}H_{22}O_2+Na]^+$): 233.1512, found: 233.1514.

2-(Prop-2-yn-1-yl)octan-1-ol (S1b)

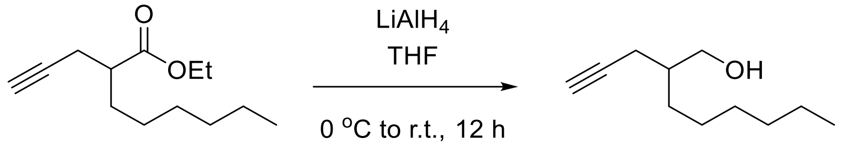

To a stirred suspension of lithium aluminum hydride (1.91 g, 50.3 mmol, 1.50 equiv.) in anhydrous THF (70.0 mL) was added dropwise a solution of **S3** (7.05 g, 33.5 mmol, 1.00 equiv.) in anhydrous THF (15.0 mL) at 0 °C under argon. After the resultant mixture was stirred at room temperature for 12 h, to the reaction mixture was added dropwise water (1.91 mL), 15% NaOH aq. (1.91 mL), and water (5.73 mL) at 0 °C. After the resultant mixture was stirred at room temperature for 30 min, the mixture was filtered through a pad of celite® and eluted with diethyl ether. The filtrate was concentrated under reduced pressure. The residue was purified by column chromatography on silica gel (hexane/ethyl acetate = 10 : 1) to give 2-(prop-2-yn-1-yl)octan-1-ol (**S1b**) (5.45 g, 32.4 mmol, 97%) as a yellow oil.

IR (neat): 3312, 2956, 2927, 2857, 2116, 1467, 1434, 1034, 632 cm^{-1}; ^{1}H NMR (400 MHz, $CDCl_3$): δ 3.71-3.59 (m, 2H), 2.37-2.23 (m, 2H), 1.97 (t, J = 2.8 Hz, 1H), 1.77-1.68 (m, 1H), 1.63 (t, J = 2.8 Hz, 1H), 1.38-1.29 (m, 10H), 0.88 (t, J = 6.4 Hz, 3H); ^{13}C NMR (100 MHz, $CDCl_3$): δ 82.8, 69.7, 65.3, 39.7, 31.9, 30.4, 29.6, 27.0, 22.8, 20.3, 14.2; HRMS (ESI): calcd. for ($[C_{11}H_{20}O+Na]^+$): 191.1406, found: 191.1405.

2-((2-(Prop-2-yn-1-yl)octyl)oxy)tetrahydro-2*H*-pyran (S2b)

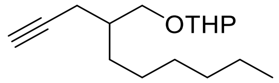

According to the **general procedure A** using **S1b** (5.22 g, 31.0 mmol, 1.00 equiv.), the crude product was purified by column chromatography on silica gel (hexane/ethyl acetate = 25 : 1) to give 2-((2-(prop-2-yn-1-yl)octyl)oxy)tetrahydro-2*H*-pyran (**S2b**) (7.61 g, 30.2 mmol, 97%, diastereomer mixture) as a colorless oil.

IR (neat): 3312, 2926, 2857, 2117, 1467, 1353, 1200, 1121, 1033, 977, 632 cm^{-1}; ^{1}H NMR (400 MHz, $CDCl_3$, diastereomer mixture): δ 4.59 (m, 1H), 3.89-3.83 (m, 1H), 3.76-3.66 (m, 1H), 3.53-3.48 (m, 1H), 3.38-3.28 (m, 1H), 2.37-2.25 (m, 2H), 1.93-1.92 (m, 1H), 1.86-1.28 (m, 17H), 0.88 (t, J = 6.6 Hz, 3H); ^{13}C NMR (100 MHz, $CDCl_3$, diastereomer mixture): δ 99.2, 98.5, 82.8, 82.7, 69.6, 69.3, 69.1, 62.2, 61.8, 37.7, 37.6, 31.8, 30.70, 30.65, 30.5, 30.4, 29.5, 26.90, 26.88, 25.6, 22.7, 20.5, 20.4, 19.6, 19.3, 14.1; HRMS (ESI): calcd. for ($[C_{16}H_{28}O_2+Na]^+$): 275.1982, found: 275.1985.

***t*-Butyl 2-(benzyloxy)acetate (S4)**

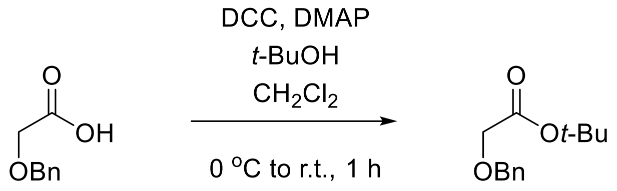

S4

Compound **S4** was prepared according to the modified procedure of Zakarian et al[3].

To a stirred solution of benzyloxyacetic acid (14.2 mL, 100 mmol, 1.00 equiv.), *t*-butyl alcohol (19.0 mL, 200 mmol, 2.00 equiv.), and 4-dimethylaminopyridine (1.22 g, 10.0 mmol, 10.0 mol%) in anhydrous CH_2Cl_2 (300 mL) was added *N,N'*-dicyclohexylcarbodiimide (26.8 g, 130 mmol, 1.30 equiv.) at 0 °C under argon. After the resultant mixture was stirred at room temperature for 1 h, the reaction mixture was filtered. The filtrate was washed with sat. $NaHCO_3$ aq. (30.0 mL) and brine (30.0 mL), dried over Na_2SO_4, filtered, and concentrated under reduced pressure. The residue was diluted with Et_2O/hexane (1 : 1, 60.0 mL) and stirred at room temperature for 1 h. The mixture was filtered and the filtrate was concentrated under reduced pressure. The residue was purified by column chromatography on silica gel (hexane/ethyl acetate = 50 : 1 to 20 : 1) to give *t*-butyl 2-(benzyloxy)acetate (**S4**) (18.4 g, 82.8 mmol, 83%) as a colorless oil.

^{1}H NMR (400 MHz, $CDCl_3$): δ 7.40-7.26 (m, 5H), 4.62 (s, 2H), 3.99 (s, 2H), 1.49 (s, 9H); ^{13}C NMR (100 MHz, $CDCl_3$): δ 169.7, 137.5, 128.6, 128.2, 128.0, 81.7, 73.3, 67.9, 28.3.

^{1}H and ^{13}C NMR spectra were well consistent with those in the previous report[3].

***t*-Butyl 2-(benzyloxy)pent-4-ynoate (S5)**

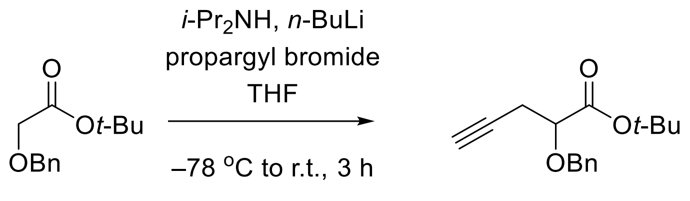

S4 **S5**

To a solution of anhydrous diisopropylamine (8.43 mL, 60.0 mmol, 1.20 equiv.) was added *n*-BuLi (1.60 M in hexane, 34.4 mL, 55.0 mmol, 1.10 equiv.) at –78 °C under argon. To the reaction mixture was added dropwise over 50 min a solution of **S4** (11.1 g, 50.0 mmol, 1.00 equiv.) in anhydrous THF (50.0 mL) at –78 °C. After the resultant mixture was stirred at the same temperature for 30 min, a solution of propargyl bromide (7.53 mL, 100 mmol, 2.00 equiv.) in anhydrous THF (25.0 mL) was added dropwise at the same temperature. After the resultant mixture was stirred at room temperature for 3 h, the reaction was quenched with sat. NH_4Cl aq. and the aqueous layer was extracted with

hexane/ethyl acetate (1 : 1) three times. The combined organic layer was washed with brine, dried over Na_2SO_4, filtered, and concentrated under reduced pressure. The residue was purified by column chromatography on silica gel (hexane/ethyl acetate = 30: 1) to give *t*-butyl 2-(benzyloxy)pent-4-ynoate (**S5**) (10.4 g, 39.9 mmol, 80%) as a colorless oil.

IR (neat): 3293, 2979, 1742, 1456, 1369, 1226, 1158, 1119, 740, 698 cm^{-1}; ^{1}H NMR (400 MHz, $CDCl_3$): δ 7.40-7.26 (m, 5H), 4.76 (d, J = 11.9 Hz, 1H), 4.53 (d, J = 11.9 Hz, 1H), 3.99-3.94 (m, 1H), 2.66-2.64 (m, 2H), 2.03 (t, J = 2.7 Hz, 1H), 1.50 (s, 9H); ^{13}C NMR (100 MHz, $CDCl_3$): δ 170.1, 137.5, 128.5, 128.2, 128.0, 82.2, 79.5, 76.7, 72.5, 70.6, 28.2, 23.3; HRMS (ESI): calcd. for ([$C_{16}H_{20}O_3$+Na]$^+$): 283.1305, found: 283.1304.

2-(Benzyloxy)pent-4-yn-1-ol (S1c)

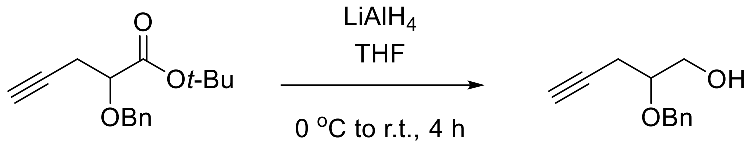

S5 **S1c**

To a stirred suspension of lithium aluminum hydride (2.14 g, 56.3 mmol, 1.50 equiv.) in anhydrous THF (75.0 mL) was added dropwise a solution of **21** (9.76 g, 37.5 mmol, 1.00 equiv.) in anhydrous THF (20.0 mL) at 0 °C under argon. After the resultant mixture was stirred at room temperature for 4 h, to the reaction mixture was added dropwise water (2.14 mL), 15% NaOH aq. (2.14 mL), and water (6.42 mL) at 0 °C. After the resultant mixture was stirred at room temperature for 30 min, the mixture was filtered through a pad of celite® and eluted with diethyl ether. The filtrate was concentrated under reduced pressure. The residue was purified by column chromatography on silica gel (hexane/ethyl acetate = 4 : 1) to give 2-(benzyloxy)pent-4-yn-1-ol (**S1c**) (6.82 g, 35.8 mmol, 95%) as a colorless oil.

IR (neat): 3421, 3293, 2925, 2877, 1455, 1350, 1102, 1055, 741, 699, 643 cm^{-1}; ^{1}H NMR (400 MHz, $CDCl_3$): δ 7.40-7.28 (m, 5H), 4.72 (d, J = 11.9 Hz, 1H), 4.58 (d, J = 11.9 Hz, 1H), 3.83-3.76 (m, 1H), 3.72-3.65 (m, 2H), 2.56-2.43 (m, 2H), 2.02 (t, J = 2.7 Hz, 1H), 1.99-1.94 (m, 1H); ^{13}C NMR (100 MHz, $CDCl_3$): δ 138.0, 128.7, 128.1, 128.0, 80.5, 77.8, 72.0, 70.6, 63.9, 20.8; HRMS (ESI): calcd. for ([$C_{12}H_{14}O_2$+Na]$^+$): 213.0886, found: 213.0886.

2-((2-(Benzyloxy)pent-4-yn-1-yl)oxy)tetrahydro-2*H*-pyran (S2c)

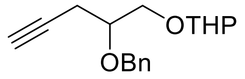

According to the **general procedure A** using **S1c** (6.47 g, 34.0 mmol, 1.00 equiv.), the crude product was purified by column chromatography on silica gel (hexane/ethyl acetate = 40 : 1) to give 2-((2-

(benzyloxy)pent-4-yn-1-yl)oxy)tetrahydro-2*H*-pyran (**S2c**) (9.28 g, 33.8 mmol, 99%, diastereomer mixture) as a colorless oil.
IR (neat): 3293, 2942, 2871, 1455, 1352, 1201, 1123, 1065, 1035, 738, 698 cm^{-1}; ^{1}H NMR (400 MHz, $CDCl_3$, diastereomer mixture): δ 7.39-7.26 (m, 5H), 4.69 (s, 2H), 4.75-4.60 (m, 1H), 3.92-3.83 (m, 2H), 3.80-3.72 (m, 1H), 3.59-3.48 (m, 2H), 2.60-2.44 (m, 2H), 2.00 (t, *J* = 2.5 Hz, 1H), 1.87-1.48 (m, 6H); ^{13}C NMR (100 MHz, $CDCl_3$, diastereomer mixture): δ 138.6, 138.5, 128.5, 127.9, 127.8, 127.7, 99.2, 98.8, 81.1, 80.9, 76.4, 76.2, 72.2, 72.0, 70.12, 70.07, 68.8, 68.1, 62.2, 62.0, 30.7, 30.6, 25.6, 21.82, 21.77, 19.4, 19.3; HRMS (ESI): calcd. for ($[C_{17}H_{22}O_3+Na]^+$): 297.1461, found: 297.1461.

2-(Hex-5-yn-1-yloxy)tetrahydro-2*H*-pyran (S2d)

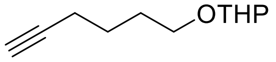

According to the **general procedure A** using 5-hexyn-1-ol (**S1d**) (10.8 mL, 100 mmol, 1.00 equiv.), the crude product was purified by column chromatography on silica gel (hexane/ethyl acetate = 15 : 1) to give 2-(hex-5-yn-1-yloxy)tetrahydro-2*H*-pyran (**S2d**) (17.9 g, 98.4 mmol, 98%) as a colorless oil.
^{1}H NMR (400 MHz, $CDCl_3$): δ 4.58 (t, *J* = 3.4 Hz, 1H), 3.89-3.73 (m, 2H), 3.53-3.39 (m, 2H), 2.24 (dt, *J* = 2.7, 7.1 Hz, 2H), 1.95 (t, *J* = 2.7 Hz, 1H), 1.86-1.51 (m, 10H); ^{13}C NMR (100 MHz, $CDCl_3$): δ 99.0, 84.5, 68.5, 67.1, 62.4, 30.9, 29.0, 25.6, 25.5, 19.8, 18.4.
^{1}H and ^{13}C NMR spectra were well consistent with those in the previous report[4].

2-(Hept-6-yn-1-yloxy)tetrahydro-2*H*-pyran (S2e)

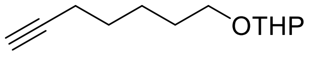

According to the **general procedure A** using 6-heptyn-1-ol (**S1e**) (8.72 mL, 70.0 mmol, 1.00 equiv.), the crude product was purified by column chromatography on silica gel (hexane/ethyl acetate = 25 : 1) to give 2-(hept-6-yn-1-yloxy)tetrahydro-2*H*-pyran (**S2e**) (13.6 g, 69.3 mmol, 99%) as a colorless oil.
^{1}H NMR (400 MHz, $CDCl_3$): δ 4.58 (t, *J* = 3.7 Hz, 1H), 3.90-3.72 (m, 2H), 3.53-3.39 (m, 2H), 2.20 (dt, *J* = 2.3, 6.9 Hz, 2H), 1.94-1.44 (m, 13H); ^{13}C NMR (100 MHz, $CDCl_3$): δ 99.0, 84.7, 68.3, 67.5, 62.5, 30.9, 29.4, 28.5, 25.63, 25.58, 19.8, 18.5.
^{1}H and ^{13}C NMR spectra were well consistent with those in the previous report[5].

3.2. Preparation of aldehyde

General procedure B for the mono-protection of alcohol S6

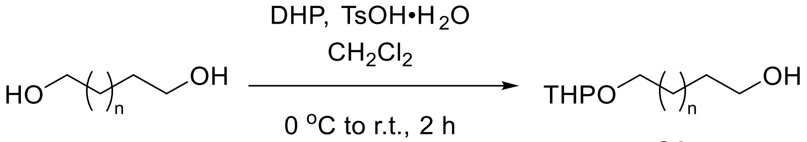

S6

To a stirred solution of diol (4.00–5.00 equiv.) and 3,4-dihydro-2*H*-pyran (1.00 equiv.) in anhydrous CH_2Cl_2 (0.250 M) was added *p*-toluenesulfonic acid monohydrate (5.00 mol%) at 0 °C under argon. After the resultant mixture was stirred at room temperature for 2 h, the reaction was quenched with sat. $NaHCO_3$ aq. and the aqueous layer was extracted with CH_2Cl_2 three times. The combined organic layer was washed with brine, dried over Na_2SO_4, filtered, and concentrated under reduced pressure. The residue was purified by column chromatography on silica gel to give the desired mono-protected alcohol **S6**.

General procedure C for the oxidation of alcohol S6

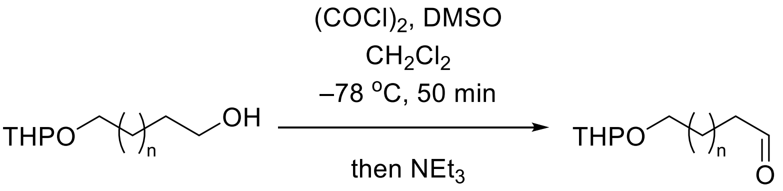

S6 **S7**

To a stirred solution of oxalyl chloride (1.50 equiv.) in anhydrous CH_2Cl_2 (0.300 M) was added dropwise a solution of dimethyl sulfoxide (3.00 equiv.) in anhydrous CH_2Cl_2 (10.0 mL) at –78 °C under argon. After the resultant mixture was stirred at –78 °C for 15 min, a solution of alcohol **S6** (1.00 equiv.) in anhydrous CH_2Cl_2 (20.0 mL) was added dropwise at –78 °C. After the resultant mixture was stirred at –78 °C for 50 min, to the reaction mixture was added trimethylamine (5.00 equiv.) at –78 °C. After the resultant mixture was stirred at room temperature for 40 min, the reaction was quenched with sat. NH_4Cl aq. and the aqueous layer was extracted with CH_2Cl_2 three times. The combined organic layer was washed with brine, dried over Na_2SO_4, filtered, and concentrated under reduced pressure. To remove excess amount of dimethyl sulfoxide, the residue was diluted with Et_2O/water (1 : 1) and the aqueous layer was extracted with Et_2O three times. The combined organic layer was washed with brine, dried over Na_2SO_4, filtered, and concentrated under reduced pressure. The residue of **S7** was used for the next reaction without further purification.

4-((Tetrahydro-2*H*-pyran-2-yl)oxy)butan-1-ol (S6a)

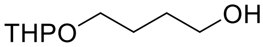

According to the **general procedure B** using 1,4-butanediol (17.7 mL, 200 mmol, 4.00 equiv.) and 3,4-dihydro-2*H*-pyran (4.52 mL, 50.0 mmol, 1.00 equiv.), the crude product was purified by column chromatography on silica gel (hexane/ethyl acetate = 2 : 1) to give 4-((tetrahydro-2*H*-pyran-2-yl)oxy)butan-1-ol (**S6a**) (6.80 g, 39.0 mmol, 78%) as a colorless oil.

^{1}H NMR (400 MHz, $CDCl_3$): δ 4.61 (t, J = 3.4 Hz, 1H), 3.89-3.78 (m, 2H), 3.67 (s, 2H), 3.54-3.43 (m, 2H), 2.20 (br, 1H), 1.86-1.51 (m, 10H); ^{13}C NMR (100 MHz, $CDCl_3$): δ 99.0, 67.7, 62.9, 62.5, 30.8, 30.3, 26.7, 25.5, 19.7.

^{1}H and ^{13}C NMR spectra were well consistent with those in the previous report[6].

4-((Tetrahydro-2*H*-pyran-2-yl)oxy)butanal (S7a)

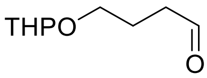

According to the **general procedure C** using **S6a** (6.10 g, 35.0 mmol, 1.00 equiv.), a crude product of **4a** was obtained. The residue was used for the next reaction without further purification.

5-((Tetrahydro-2*H*-pyran-2-yl)oxy)pentan-1-ol (S6b)

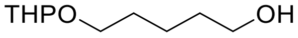

According to the **general procedure B** using 1,5-pentanediol (42.1 mL, 400 mmol, 5.00 equiv.) and 3,4-dihydro-2*H*-pyran (7.24 mL, 80.0 mmol, 1.00 equiv.), the crude product was purified by column chromatography on silica gel (hexane/ethyl acetate = 2 : 1) to give 5-((tetrahydro-2*H*-pyran-2-yl)oxy)pentan-1-ol (**S6b**) (11.9 g, 63.2 mmol, 79%) as a colorless oil.

^{1}H NMR (400 MHz, $CDCl_3$): δ 4.58 (t, J = 3.4 Hz, 1H), 3.90-3.82 (m, 1H), 3.78-3.73 (m, 1H), 3.67-3.63 (m, 2H), 3.53-3.45 (m, 1H), 3.43-3.38 (m, 1H), 1.86-1.42 (m, 13H); ^{13}C NMR (100 MHz, $CDCl_3$): δ 99.0, 67.6, 62.9, 62.5, 32.6, 30.9, 29.6, 25.6, 22.6, 19.8.

^{1}H and ^{13}C NMR spectra were well consistent with those in the previous report[7].

5-((Tetrahydro-2*H*-pyran-2-yl)oxy)pentanal (S7b)

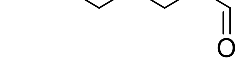

According to the **general procedure C** using **S6b** (5.65 g, 30.0 mmol, 1.00 equiv.), a crude product of **S7b** was obtained. The residue was used for the next reaction without further purification.

6-((Tetrahydro-2*H*-pyran-2-yl)oxy)hexan-1-ol (S6c)

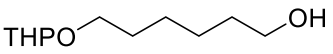

According to the **general procedure B** using 1,6-hexanediol (47.3 g, 400 mmol, 5.00 equiv.) and 3,4-dihydro-2*H*-pyran (7.24 mL, 80.0 mmol, 1.00 equiv.), the crude product was purified by column chromatography on silica gel (hexane/ethyl acetate = 3 : 1) to give 6-((tetrahydro-2*H*-pyran-2-yl)oxy)hexan-1-ol (**S6c**) (11.1 g, 54.9 mmol, 69%) as a colorless oil.

^{1}H NMR (400 MHz, $CDCl_3$): δ 4.58 (t, *J* = 6.9 Hz, 1H), 3.90-3.84 (m, 1H), 3.74 (dt, *J* = 6.9, 9.6 Hz, 1H), 3.65 (t, *J* = 6.6 Hz, 2H), 3.53-3.48 (m, 1H), 3.40 (dt, *J* = 6.4, 9.6 Hz, 1H), 1.86-1.38 (m, 15H); ^{13}C NMR (100 MHz, $CDCl_3$): δ 99.0, 67.7, 63.0, 62.6, 32.8, 30.9, 29.8, 26.2, 25.7, 25.6, 19.9.

^{1}H and ^{13}C NMR spectra were well consistent with those in the previous report[8].

6-((Tetrahydro-2*H*-pyran-2-yl)oxy)hexanal (S7c)

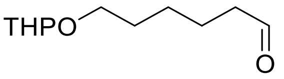

According to the **general procedure C** using **S6c** (5.05 g, 25.0 mmol, 1.00 equiv.), a crude product of **S7c** was obtained. The residue was used for the next reaction without further purification.

3.3. Preparation of cyclization precursors

General procedure D for 1,2-addition of alkynyl lithium to aldehyde S7

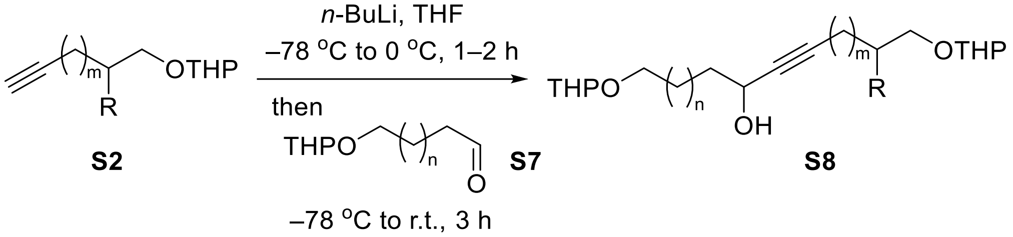

To a stirred solution of alkyne **S2** (1.50 equiv.) in anhydrous THF (0.333 M) was added dropwise *n*-BuLi (1.56 M in hexane, 1.50 equiv.) at –78 °C under argon. After the resultant mixture was stirred at 0 °C for 1–2 h, a solution of aldehyde **S7** (1.00 equiv.) in anhydrous THF (5.00 mL) was added dropwise at –78 °C. After the resultant mixture was stirred at room temperature for 3 h, the reaction was quenched with sat. NH_4Cl aq. and the aqueous layer was extracted with ethyl acetate three times. The combined organic layer was washed with brine, dried over Na_2SO_4, filtered, and concentrated under reduced pressure. The residue was purified by column chromatography on silica gel to give the desired propargyl alcohol **S8**.

General procedure E for the synthesis of allene S9

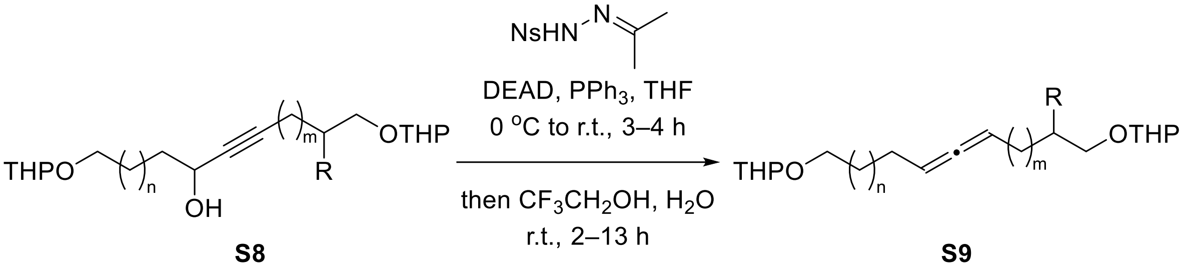

S8 **S9**

To a stirred solution of propargyl alcohol **S8** (1.00 equiv.), *N*'-isopropylidene-2-nitrobenzenesulfonohydrazide (2.30–2.50 equiv.), and triphenylphosphine (2.30–2.50 equiv.) in anhydrous THF (0.100 M) was added dropwise diethyl azodicarboxylate (2.20 M in toluene, 2.50 equiv.) at 0 °C under argon. After the resultant mixture was stirred at room temperature for 3–4 h, to the reaction mixture were added 2,2,2-trifluoroethanol (4.20 mL/1.00 mmol substrate) and water (4.20 mL/1.00 mmol substrate) at room temperature. After the resultant mixture was stirred at room temperature for 2–13 h, the reaction mixture was concentrated under reduced pressure. The residue was diluted with hexane/ethyl acetate (1 : 1) and water, and extracted with hexane/ethyl acetate (1 : 1) three times. The combined organic layer was washed with brine, dried over Na_2SO_4, filtered, and concentrated under reduced pressure. The residue was diluted with hexane/ethyl acetate (4 : 1, 100 mL). After the resultant mixture was stirred at room temperature for 30 min, the precipitate was filtered off and the filtrate was concentrated under reduced pressure. The residue was purified by column chromatography on silica gel to give the desired allene **S9**.

General procedure F for the synthesis of diol S10

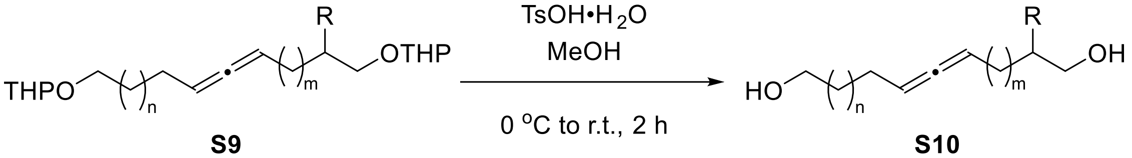

S9 **S10**

To a stirred solution of protected alcohol **S9** (1.00 equiv.) in MeOH (0.200 M) was added *p*-toluenesulfonic acid monohydrate (10.0–20.0 mol%) at 0 °C. After the resultant mixture was stirred at the same temperature for 2 h, the reaction was quenched with sat. $NaHCO_3$ aq. and the reaction mixture was concentrated under reduced pressure. The residue was diluted with $CHCl_3$/MeOH (4 : 1) and water, and extracted with $CHCl_3$/MeOH (4 : 1) three times. The combined organic layer was dried over Na_2SO_4, filtered, and concentrated under reduced pressure. The residue was purified by column chromatography on silica gel to give the desired diol **S10**.

General procedure G for the synthesis of bisaldehyde 1

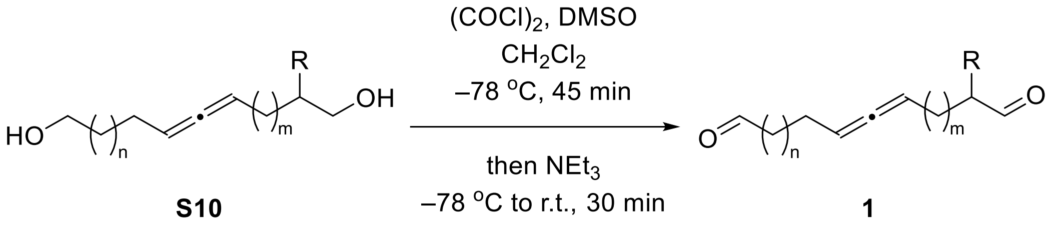

To a stirred solution of oxalyl chloride (4.00 equiv.) in anhydrous CH_2Cl_2 (0.125 M) was added dropwise a solution of dimethyl sulfoxide (6.00 equiv.) in anhydrous CH_2Cl_2 (2.00 mL) at –78 °C under argon. After the resultant mixture was stirred at –78 °C for 15 min, a solution of diol **S10** (1.00 equiv.) in anhydrous CH_2Cl_2 (5.00 mL) was added dropwise at –78 °C. After the resultant mixture was stirred at –78 °C for 45 min, to the reaction mixture was added triethylamine (10.0 equiv.) at –78 °C. After the resultant mixture was stirred at room temperature for 30 min, the reaction was quenched with sat. NH_4Cl aq. and the aqueous layer was extracted with CH_2Cl_2 three times. The combined organic layer was washed with brine, dried over Na_2SO_4, filtered, and concentrated under reduced pressure. The residue was purified by column chromatography on silica gel to give the desired bisaldehyde **1**.

General procedure H for the synthesis of bisoxime 4

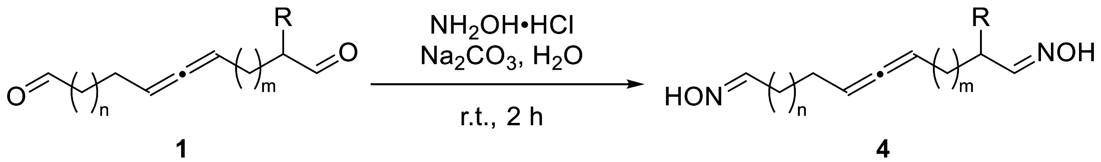

To a stirred solution of bisaldehyde **1** (1.00 equiv.) in water (0.300 M) were added sodium carbonate (1.00 equiv.) and hydroxylamine hydrochloride (2.50 equiv.) at room temperature. After the resultant mixture was stirred at room temperature for 1 h, the reaction mixture was diluted with ethyl acetate, and the aqueous layer was extracted with ethyl acetate three times. The combined organic layer was washed with brine, dried over Na_2SO_4, and concentrated under reduced pressure. The residue was purified by column chromatography on silica gel to give the desired bisoxime **4**.

1,9-Bis((tetrahydro-2*H*-pyran-2-yl)oxy)non-5-yn-4-ol (S8a)

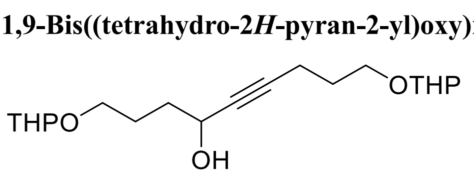

According to the **general procedure D** using alkyne **S2a** (3.36 g, 20.0 mmol, 1.50 equiv.) and aldehyde **S7a** (2.29 g, 13.3 mmol, 1.00 equiv.), the crude product was purified by column chromatography on silica gel (hexane/ethyl acetate = 5 : 1 to 3 : 1 to 2 : 1) to give 1,9-bis((tetrahydro-

2*H*-pyran-2-yl)oxy)non-5-yn-4-ol (**S8a**) (3.75 g, 11.0 mmol, 83%, diastereomer mixture) as a yellow oil.

IR (neat): 3432, 2942, 2870, 1137, 1119, 1075, 1063, 1034, 1023, 989 cm^{-1}; Although compound **S8a** should be a mixture of diastereomers, both ^{1}H and ^{13}C NMR spectra do not reflect this but the presence of a single isomer. ^{1}H NMR (400 MHz, $CDCl_3$): δ 4.63-4.58 (m, 2H), 4.44-4.40 (m, 1H), 3.89-3.76 (m, 4H), 3.54-3.41 (m, 4H), 2.59-2.53 (m, 1H), 2.33 (dt, *J* = 1.8, 7.3 Hz, 2H), 1.86-1.51 (m, 18H); ^{13}C NMR (100 MHz, $CDCl_3$): δ 98.5, 98.42, 98.38, 84.0, 81.6, 67.03, 66.99, 65.7, 61.9, 35.0, 30.4, 28.7, 25.4, 25.3, 19.2, 15.4; HRMS (ESI): calcd. for ($[C_{19}H_{32}O_5+Na]^+$): 363.2142, found: 363.2144.

1,9-Bis((tetrahydro-2*H*-pyran-2-yl)oxy)nona-4,5-diene (S9a)

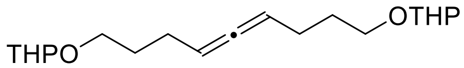

According to the **general procedure E** using propargyl alcohol **S8a** (3.06 g, 9.00 mmol, 1.00 equiv.), the crude product was purified by column chromatography on silica gel (hexane/ethyl acetate = 100 : 3 to 20 : 1) to give 1,9-bis((tetrahydro-2*H*-pyran-2-yl)oxy)nona-4,5-diene (**S9a**) (1.44 g, 4.43 mmol, 49%, diastereomer mixture) as a yellow oil.

IR (neat): 2940, 2869, 1962, 1137, 1119, 1076, 1063, 1034, 1021, 990 cm^{-1}; ^{1}H NMR (400 MHz, $CDCl_3$, diastereomer mixture): δ 5.15-5.10 (m, 2H), 4.58 (t, *J* = 3.4 Hz, 2H), 3.90-3.74 (m, 4H), 3.53-3.39 (m, 4H), 2.11-2.04 (m, 4H), 1.86-1.51 (m, 16H); ^{13}C NMR (100 MHz, $CDCl_3$, diastereomer mixture): δ 204.0, 99.0, 98.9, 91.0, 67.1, 67.0, 62.39, 62.36, 30.9, 29.34, 29.32, 25.7, 25.6, 19.8, 19.7; HRMS (ESI): calcd. for ($[C_{19}H_{32}O_4+Na]^+$): 347.2193, found: 347.2195.

Nona-4,5-diene-1,9-diol (S10a)

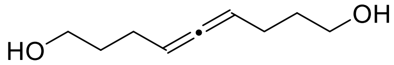

According to the **general procedure F** using protected diol **S9a** (1.17 g, 3.60 mmol, 1.00 equiv.), the crude product was purified by column chromatography on silica gel (CH_2Cl_2/MeOH = 98 : 2 to 97 : 3 to 95 : 5) to give nona-4,5-diene-1,9-diol (**S10a**) (548 mg, 3.51 mmol, 98%) as a colorless oil.

IR (neat): 3335, 2937, 2873, 1961, 1445, 1057, 877, 695 cm^{-1}; ^{1}H NMR (400 MHz, $CDCl_3$): δ 5.14-5.10 (m, 2H), 3.69 (t, *J* = 6.6 Hz, 4H), 2.13-2.06 (m, 4H), 1.77-1.63 (m, 6H); ^{13}C NMR (100 MHz, $CDCl_3$): δ 204.2, 90.8, 62.2, 31.9, 25.2; HRMS (ESI): calcd. for ($[C_9H_{16}O_2+Na]^+$): 179.1043, found: 179.1042.

Nona-4,5-dienedial (1a)

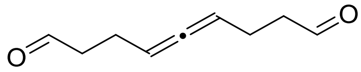

According to the **general procedure G** using diol **S10a** (548 mg, 3.51 mmol, 1.00 equiv.), the crude product was purified by column chromatography on silica gel (hexane/ethyl acetate = 7 : 1 to 5 : 1) to give nona-4,5-dienedial (**1a**) (430 mg, 2.83 mmol, 81%) as a yellow oil.

IR (neat): 2920, 2833, 2727, 1962, 1722, 1407, 1390, 1061 cm^{-1}; ^{1}H NMR (400 MHz, $CDCl_3$): δ 9.78 (t, *J* = 1.4 Hz, 2H), 5.27-5.22 (m, 2H), 2.55 (dt, *J* = 1.4, 7.1 Hz, 4H), 2.35-2.29 (m, 4H); ^{13}C NMR (100 MHz, $CDCl_3$): δ 204.1, 202.0, 91.5, 42.5, 21.3; HRMS (ESI): calcd. for ($[C_9H_{12}O_2+Na]^+$): 175.0730, found: 175.0735.

Nona-4,5-dienedial bisoxime (4a)

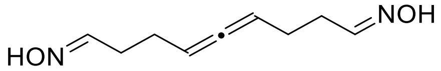

According to the **general procedure H** using bisaldehyde **1a** (350 mg, 2.30 mmol, 1.00 equiv.), the crude product was purified by column chromatography on silica gel (hexane/ethyl acetate = 3 : 2 to 1 : 1) to give nona-4,5-dienedial bisoxime (**4a**) (261 mg, 1.43 mmol, 61%, diastereomer mixture) as a colorless solid.

Mp 72–73 °C, IR (neat): 3197, 3086, 2905, 2853, 1966, 1667, 1433, 1308, 941 cm^{-1}; ^{1}H NMR (400 MHz, $CDCl_3$, diastereomer mixture): δ 8.05 (br, 2H), 7.46-7.43 (m, 1H), 6.76-6.73 (m, 1H), 5.23-5.16 (m, 2H), 2.54-2.47 (m, 2H), 2.36-2.31 (m, 2H), 2.23-2.17 (m, 4H); ^{13}C NMR (100 MHz, $CDCl_3$, diastereomer mixture): δ 204.4, 204.3, 204.2, 152.3, 152.2, 151.70, 151.66, 91.3, 91.1, 90.9, 28.9, 28.8, 25.8, 25.7, 25.15, 25.06, 24.3, 24.2; HRMS (ESI): calcd. for ($[C_9H_{14}N_2O_2+Na]^+$): 205.0947, found: 205.0949.

1-((Tetrahydro-2*H*-pyran-2-yl)oxy)-8-(((tetrahydro-2*H*-pyran-2-yl)oxy)methyl)tetradec-5-yn-4-ol (S8b)

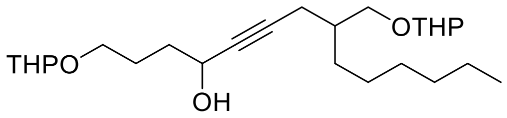

According to the **general procedure D** using alkyne **S2b** (7.57 g, 30.0 mmol, 1.50 equiv.) and aldehyde **S7a** (3.44 g, 20.0 mmol, 1.00 equiv.), the crude product was purified by column chromatography on silica gel (hexane/ethyl acetate = 7 : 1 to 5 : 1 to 3 : 1) to give 1-((tetrahydro-2*H*-pyran-2-yl)oxy)-8-(((tetrahydro-2*H*-pyran-2-yl)oxy)methyl)tetradec-5-yn-4-ol (**S8b**) (6.61 g, 15.6 mmol, 78%, diastereomer mixture) as a yellow oil.

IR (neat): 3445, 2927, 2857, 1455, 1353, 1200, 1137, 1119, 1077, 1033, 868 cm^{-1}; ^{1}H NMR (400 MHz, $CDCl_3$, diastereomer mixture): δ 4.62-4.57 (m, 2H), 4.42 (br, 1H), 3.89-3.65 (m, 4H), 3.52-3.42 (m, 3H), 3.36-3.27 (m, 1H), 2.68-2.62 (m, 1H), 2.35-2.31 (m, 2H), 1.84-1.67 (m, 9H), 1.60-1.51 (m, 8H), 1.39-1.28 (m, 10H), 0.88 (t, *J* = 6.6 Hz, 3H); ^{13}C NMR (100 MHz, $CDCl_3$, diastereomer mixture): δ

99.3, 98.9, 98.8, 98.7, 83.62, 83.59, 82.5, 69.8, 69.5, 67.4, 67.3, 62.5, 62.3, 62.0, 37.9, 37.8, 35.6, 31.9, 30.8, 30.73, 30.69, 29.6, 26.98, 26.95, 25.72, 25.70, 25.6, 25.5, 22.8, 20.9, 20.8, 19.63, 19.56, 19.4, 14.2; HRMS (ESI): calcd. for ($[C_{25}H_{44}O_5+Na]^+$): 447.3081, found: 447.3080.

2,2'-((2-Hexylnona-4,5-diene-1,9-diyl)bis(oxy))bis(tetrahydro-2*H*-pyran) (S9b)

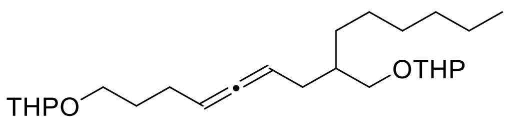

According to the **general procedure E** using propargyl alcohol **S8b** (6.20 g, 14.6 mmol, 1.00 equiv.), the crude product was purified by column chromatography on silica gel (hexane/ethyl acetate = 30 : 1 to 20 : 1) to give 2,2'-((2-hexylnona-4,5-diene-1,9-diyl)bis(oxy))bis(tetrahydro-2*H*-pyran) (**S9b**) (2.72 g, 6.66 mmol, 44%, diastereomer mixture) as a clear yellow oil.

IR (neat): 2926, 2870, 2856, 1961, 1454, 1352, 1200, 1137, 1120, 1077, 1034, 870 cm^{-1}; ^1H NMR (400 MHz, $CDCl_3$, diastereomer mixture): δ 5.08-5.02 (m, 2H), 4.58-4.54 (m, 2H), 3.89-3.82 (m, 2H), 3.77 (dt, J = 6.6, 9.6 Hz, 1H), 3.69-3.63 (m, 1H), 3.53-3.47 (m, 2H), 3.45-3.39 (m, 1H), 3.31-3.24 (m, 1H), 2.18-1.96 (m, 4H), 1.86-1.27 (m, 25H), 0.88 (t, J = 6.6 Hz, 3H); ^{13}C NMR (100 MHz, $CDCl_3$, diastereomer mixture): δ 204.8, 99.2, 99.02, 98.97, 98.9, 90.03, 90.00, 89.4, 89.34, 89.26, 89.2, 70.5, 70.4, 70.3, 70.2, 67.04, 66.98, 62.4, 62.33, 62.26, 62.2, 62.13, 62.08, 38.7, 38.6, 32.0, 31.4, 31.3, 31.2, 31.14, 31.11, 31.05, 31.0, 30.9, 29.8, 29.4, 27.02, 26.96, 26.9, 25.8, 25.70, 25.65, 22.8, 19.74, 19.72, 19.65, 19.60, 19.56, 14.2; HRMS (ESI): calcd. for ($[C_{25}H_{44}O_4+Na]^+$): 431.3132, found: 431.3134.

2-Hexylnona-4,5-diene-1,9-diol (S10b)

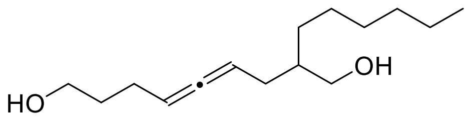

According to the **general procedure F** using protected diol **S9b** (2.55 g, 6.26 mmol, 1.00 equiv.), the crude product was purified by column chromatography on silica gel (hexane/ethyl acetate = 3 : 1) to give 2-hexylnona-4,5-diene-1,9-diol (**S10b**) (1.43 g, 5.95 mmol, 95%, diastereomer mixture) as a colorless oil.

IR (neat): 3334, 2925, 2856, 1962, 1457, 1377, 1056, 1036, 877 cm^{-1}; ^1H NMR (400 MHz, $CDCl_3$, diastereomer mixture): δ 5.10-5.03 (m, 2H), 3.71-3.54 (m, 4H), 2.15-2.00 (m, 4H), 1.84-1.54 (m, 5H), 1.38-1.28 (m, 10H), 0.88 (t, J = 6.9 Hz, 3H); ^{13}C NMR (100 MHz, $CDCl_3$, diastereomer mixture): δ 204.9, 90.1, 89.9, 89.5, 88.9, 65.5, 65.0, 62.0, 40.8, 40.6, 32.0, 31.93, 31.85, 31.1, 31.0, 30.7, 30.6, 29.8, 27.14, 27.07, 25.2, 22.8, 14.2; HRMS (ESI): calcd. for ($[C_{15}H_{28}O_2+Na]^+$): 263.1982, found: 263.1984.

2-Hexylnona-4,5-dienedial (1b)

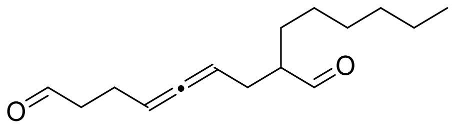

According to the **general procedure G** using diol **S10b** (962 mg, 4.00 mmol, 1.00 equiv.), the crude product was purified by column chromatography on silica gel (hexane/ethyl acetate = 15 : 1) to give 2-hexylnona-4,5-dienedial (**1b**) (779 mg, 3.30 mmol, 83%, diastereomer mixture) as a colorless oil.
IR (neat): 2955, 2927, 2856, 2720, 1963, 1725, 1458, 1389, 1057, 878, 724 cm^{-1}; ^{1}H NMR (400 MHz, $CDCl_3$, diastereomer mixture): δ 9.78-9.77 (m, 1H), 9.62-9.61 (m, 1H), 5.23-5.09 (m, 2H), 2.57-2.52 (m, 2H), 2.41-2.28 (m, 4H), 2.20-2.13 (m, 1H), 1.71-1.62 (m, 1H), 1.53-1.43 (m, 1H), 1.32-1.26 (m, 8H), 0.88 (t, J = 6.9 Hz, 3H); ^{13}C NMR (100 MHz, $CDCl_3$, diastereomer mixture): δ 204.8, 204.73, 204.70, 202.0, 201.9, 90.7, 90.6, 89.9, 89.8, 51.5, 51.4, 42.5, 31.7, 29.5, 28.8, 28.7, 28.4, 28.2, 27.02, 26.99, 22.7, 21.3, 14.2; HRMS (ESI): calcd. for ($[C_{15}H_{24}O_2+Na]^+$): 259.1669, found: 259.1666.

2-Hexylnona-4,5-dienedial bisoxime (4b)

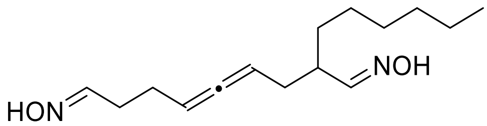

According to the **general procedure H** using bisaldehyde **1b** (355 mg, 1.50 mmol, 1.00 equiv.), the crude product was purified by column chromatography on silica gel (hexane/ethyl acetate = 5 : 1) to give 2-hexylnona-4,5-dienedial bisoxime (**4b**) (327 mg, 1.23 mmol, 82%, diastereomer mixture) as a colorless oil.
IR (neat): 3254, 3107, 2954, 2926, 2856, 1964, 1654, 1457, 1338, 931, 724 cm^{-1}; ^{1}H NMR (400 MHz, $CDCl_3$, ^{1}H NMR spectrum was observed as a mixture of two isomer groups): (major isomers) δ 9.09 (br, 2H), 7.44 (t, J = 6.0 Hz, 1H), 6.77-6.73 (m, 1H), 5.14-5.05 (m, 2H), 2.52-2.44 (m, 1H), 2.43-2.29 (m, 1H), 2.21-2.01 (m, 4H), 1.51-1.26 (m, 11H), 0.89-0.86 (m, 3H); (minor isomers) δ 9.09 (br, 2H), 7.27-7.23 (m, 1H), 6.53-6.51 (m, 1H), 5.14-5.05 (m, 2H), 2.52-2.44 (m, 1H), 2.43-2.29 (m, 1H), 2.21-2.01 (m, 4H), 1.51-1.26 (m, 11H), 0.89-0.86 (m, 3H); ^{13}C NMR (100 MHz, $CDCl_3$, diastereomer mixture): δ 205.0, 204.9, 204.8, 204.7, 155.9, 155.8, 155.31, 155.26, 152.3, 152.2, 152.16, 151.7, 151.6, 90.21, 90.19, 90.1, 90.04, 89.99, 89.9, 89.7, 89.54, 89.47, 89.4, 89.2, 40.04, 40.01, 39.8, 39.6, 35.2, 35.1, 34.9, 32.8, 32.7, 32.63, 32.60, 32.58, 32.5, 32.4, 32.3, 32.2, 32.0, 31.81, 31.79, 31.7, 29.42, 29.35, 28.9, 28.84, 28.81, 27.2, 26.99, 26.96, 25.8, 25.7, 25.6, 25.15, 25.13, 25.07, 25.0, 24.3, 22.74, 22.70, 14.2; HRMS (ESI): calcd. for ($[C_{15}H_{26}N_2O_2+Na]^+$): 289.1886, found: 289.1886.

8-(Benzyloxy)-1,9-bis((tetrahydro-2*H*-pyran-2-yl)oxy)non-5-yn-4-ol (S8c)

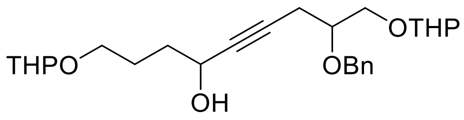

According to the **general procedure D** using alkyne **S2c** (8.23 g, 30.0 mmol, 1.50 equiv.) and aldehyde **S7a** (3.44 g, 20.0 mmol, 1.00 equiv.), the crude product was purified by column chromatography on silica gel (hexane/ethyl acetate = 5 : 1 to 3 : 1) to give 8-(benzyloxy)-1,9-bis((tetrahydro-2*H*-pyran-2-yl)oxy)non-5-yn-4-ol (**S8c**) (7.64 g, 17.1 mmol, 86%, diastereomer mixture) as a yellow oil.

IR (neat): 3421, 2941, 2870, 1455, 1352, 1200, 1120, 1075, 1033, 906 cm^{-1}; ^1H NMR (400 MHz, $CDCl_3$, diastereomer mixture): δ 7.38-7.25 (m, 5H), 4.68 (s, 2H), 4.64 (dt, J = 3.2, 3.7 Hz, 1H), 4.60-4.58 (m, 1H), 4.40-4.39 (m, 1H), 3.91-3.70 (m, 5H), 3.59-3.39 (m, 4H), 2.89-2.86 (m, 1H), 2.61-2.46 (m, 2H), 1.84-1.50 (m, 16H); ^{13}C NMR (100 MHz, $CDCl_3$, diastereomer mixture): δ 138.53, 138.49, 128.4, 127.8, 127.67, 127.66, 99.2, 98.8, 98.7, 98.6, 83.03, 83.01, 81.7, 81.5, 76.7, 76.5, 72.0, 71.9, 68.9, 68.2, 67.3, 67.2, 62.32, 62.26, 62.1, 61.9, 35.3, 30.7, 30.63, 30.59, 30.5, 25.7, 25.6, 25.5, 22.02, 21.96, 19.5, 19.3, 19.2; HRMS (ESI): calcd. for ([$C_{26}H_{38}O_6$+Na]$^+$): 469.2561, found: 469.2563.

2,2'-((2-(Benzyloxy)nona-4,5-diene-1,9-diyl)bis(oxy))bis(tetrahydro-2*H*-pyran) (S9c)

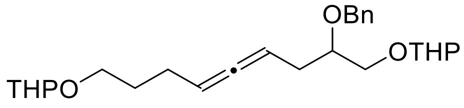

According to the **general procedure E** using propargyl alcohol **S8c** (6.52 g, 14.6 mmol, 1.00 equiv.), the crude product was purified by column chromatography on silica gel (hexane/ethyl acetate = 20 : 1) to give 2,2'-((2-(benzyloxy)nona-4,5-diene-1,9-diyl)bis(oxy))bis(tetrahydro-2*H*-pyran) (**S9c**) (2.87 g, 6.67 mmol, 46%, diastereomer mixture) as a clear yellow oil.

IR (neat): 2941, 2869, 1965, 1456, 1353, 1200, 1137, 1120, 1076, 1034 cm^{-1}; ^1H NMR (400 MHz, $CDCl_3$, diastereomer mixture): δ 7.38-7.22 (m, 5H), 5.18-5.10 (m, 2H), 4.71-4.57 (m, 4H), 3.89-3.64 (m, 5H), 3.53-3.37 (m, 4H), 2.33-2.26 (m, 2H), 2.10-2.05 (m, 2H), 1.89-1.50 (m, 14H); ^{13}C NMR (100 MHz, $CDCl_3$, diastereomer mixture): δ 205.00, 204.97, 139.03, 138.98, 128.4, 127.8, 127.6, 99.34, 99.25, 99.0, 98.9, 98.8, 98.7, 90.62, 90.60, 90.56, 87.6, 87.4, 78.2, 77.93, 77.90, 72.10, 72.06, 71.9, 69.9, 69.8, 69.10, 69.07, 67.0, 66.9, 62.4, 62.3, 62.23, 62.17, 61.94, 61.92, 31.9, 30.9, 30.7, 30.6, 29.34, 29.31, 29.29, 25.63, 25.60, 19.74, 19.72, 19.53, 19.49, 19.33, 19.31; HRMS (ESI): calcd. for ([$C_{26}H_{38}O_5$+Na]$^+$): 453.2611, found: 453.2608.

2-(Benzyloxy)nona-4,5-diene-1,9-diol (S10c)

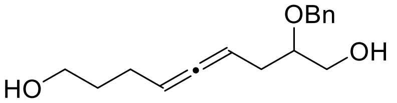

According to the **general procedure F** using protected diol **S9c** (2.67 g, 6.20 mmol, 1.00 equiv.), the crude product was purified by column chromatography on silica gel (hexane/ethyl acetate = 3 : 2 to 1 : 1) and GPC to give 2-(benzyloxy)nona-4,5-diene-1,9-diol (**S10c**) (1.40 g, 5.34 mmol, 86%, diastereomer mixture) as a colorless oil.
IR (neat): 3366, 2935, 2871, 1962, 1455, 1057, 1029, 740, 698 cm^{-1}; ^{1}H NMR (400 MHz, $CDCl_3$, diastereomer mixture): δ 7.40-7.27 (m, 5H), 5.15-5.04 (m, 2H), 4.67 (dd, J = 3.2, 11.5 Hz, 1H), 4.55 (d, J = 11.5 Hz, 1H), 3.76-3.54 (m, 5H), 2.39-2.20 (m, 2H), 2.13-2.02 (m, 2H), 1.94 (br, 2H), 1.72-1.59 (m, 2H); ^{13}C NMR (100 MHz, $CDCl_3$, diastereomer mixture): δ 205.3, 205.1, 138.4, 128.6, 127.94, 127.92, 127.87, 90.7, 90.6, 87.0, 79.4, 71.7, 71.6, 64.1, 63.7, 62.04, 61.97, 31.8, 30.6, 30.5, 25.03, 25.01; HRMS (ESI): calcd. for ($[C_{16}H_{22}O_3+Na]^+$): 285.1461, found: 285.1463.

2-(Benzyloxy)nona-4,5-dienedial (1c)

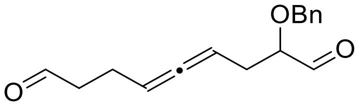

According to the **general procedure G** using diol **S10c** (1.10 g, 4.20 mmol, 1.00 equiv.), the crude product was purified by column chromatography on silica gel (hexane/ethyl acetate = 5 : 1) to give 2-(benzyloxy)nona-4,5-dienedial (**1c**) (767 mg, 2.97 mmol, 71%, diastereomer mixture) as a yellow oil.
IR (neat): 2915, 2831, 2724, 1964, 1729, 1455, 1104, 1075, 741, 699 cm^{-1}; ^{1}H NMR (400 MHz, $CDCl_3$, diastereomer mixture): δ 9.71-9.70 (m, 1H), 9.68-9.67 (m, 1H), 7.41-7.29 (m, 5H), 5.26-5.14 (m, 2H), 4.68 (dd, J = 1.6, 11.7 Hz, 1H), 4.60 (dd, J = 1.6, 11.7 Hz, 1H), 3.87-3.84 (m, 1H), 2.54-2.41 (m, 4H), 2.32-2.22 (m, 2H); ^{13}C NMR (100 MHz, $CDCl_3$, diastereomer mixture): δ 205.3, 205.2, 203.05, 202.98, 201.95, 201.92, 137.3, 128.7, 128.3, 128.2, 128.1, 90.7, 90.6, 87.4, 82.8, 72.7, 72.6, 42.4, 42.3, 30.29, 30.26, 21.1; HRMS (ESI): calcd. for ($[C_{16}H_{18}O_3+Na]^+$): 281.1148, found: 281.1147.

2-(Benzyloxy)nona-4,5-dienedial bisoxime (4c)

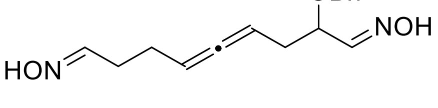

According to the **general procedure H** using bisaldehyde **1c** (310 mg, 1.20 mmol, 1.00 equiv.), the crude product was purified by column chromatography on silica gel (hexane/ethyl acetate = 2 : 1) to

give 2-(benzyloxy)nona-4,5-dienedial bisoxime (**4c**) (322 mg, 1.12 mmol, 93%, diastereomer mixture) as a colorless oil.

IR (neat): 3275, 2910, 1964, 1654, 1455, 1337, 1091, 1073, 928, 739, 698 cm^{-1}; ^{1}H NMR (400 MHz, $CDCl_3$, ^{1}H NMR spectrum was observed as a mixture of two isomer groups): (major isomers) δ 8.17 (br, 2H), 7.45-7.28 (m, 6H), 6.83-6.69 (m, 1H), 5.24-5.11 (m, 2H), 4.81-4.42 (m, 3H), 2.55-2.13 (m, 6H); (minor isomers) δ 8.17 (br, 2H), 7.45-7.28 (m, 6H), 6.83-6.69 (m, 1H), 5.24-5.11 (m, 2H), 4.81-4.42 (m, 2H), 4.12-3.99 (m, 1H), 2.55-2.13 (m, 6H); ^{13}C NMR (100 MHz, $CDCl_3$, diastereomer mixture): δ 205.6, 205.4, 205.30, 205.26, 205.2, 153.5, 153.4, 152.4, 151.94, 151.85, 151.8, 151.74, 151.69, 151.66, 137.9, 137.8, 128.5, 128.02, 127.99, 127.90, 127.86, 90.4, 90.3, 90.23, 90.19, 90.0, 89.9, 89.8, 87.7, 87.5, 87.41, 87.35, 87.19, 87.17, 87.0, 76.1, 75.8, 75.7, 72.0, 71.91, 71.87, 71.8, 71.5, 71.0, 33.73, 33.70, 33.5, 33.2, 32.6, 32.5, 32.2, 32.1, 28.7, 28.65, 28.60, 25.7, 25.5, 25.3, 25.0, 24.9, 24.8, 24.23, 24.16, 24.1; HRMS (ESI): calcd. for ($[C_{16}H_{20}N_2O_3+Na]^+$): 311.1366, found: 311.1365.

1,10-Bis((tetrahydro-2*H*-pyran-2-yl)oxy)dec-5-yn-4-ol (S8d)

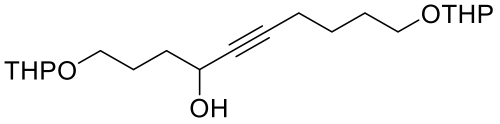

According to the **general procedure D** using alkyne **S2d** (5.47 g, 30.0 mmol, 1.50 equiv.) and aldehyde **S7a** (3.44 g, 20.0 mmol, 1.00 equiv.), the crude product was purified by column chromatography on silica gel (hexane/ethyl acetate = 3 : 1 to 2 : 1) to give 1,10-bis((tetrahydro-2*H*-pyran-2-yl)oxy)dec-5-yn-4-ol (**S8d**) (5.01 g, 14.1 mmol, 71%, diastereomer mixture) as a colorless oil.

IR (neat): 3433, 2942, 2869, 1352, 1200, 1137, 1119, 1075, 1034, 868 cm^{-1}; ^{1}H NMR (400 MHz, $CDCl_3$, diastereomer mixture): δ 4.65-4.53 (m, 2H), 4.41-4.21 (m, 1H), 3.92-3.71 (m, 4H), 3.54-3.36 (m, 4H), 2.67-2.61 (m, 1H), 2.31-2.20 (m, 2H), 1.86-1.51 (m, 20H); ^{13}C NMR (100 MHz, $CDCl_3$, diastereomer mixture): δ 99.0, 98.9, 98.8, 85.2, 81.6, 67.3, 67.1, 62.54, 62.47, 62.4, 35.6, 30.9, 30.74, 30.70, 29.1, 25.74, 25.71, 25.62, 25.60, 25.56, 19.8, 19.6, 18.7; HRMS (ESI): calcd. for ($[C_{20}H_{34}O_5+Na]^+$): 377.2298, found: 377.2300.

2,2'-(Deca-4,5-diene-1,10-diylbis(oxy))bis(tetrahydro-2*H*-pyran) (S9d)

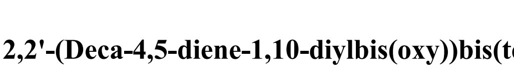

According to the **general procedure E** using propargyl alcohol **S8d** (4.43 g, 12.5 mmol, 1.00 equiv.), the crude product was purified by column chromatography on silica gel (hexane/ethyl acetate = 20 : 1) to give 2,2'-(deca-4,5-diene-1,10-diylbis(oxy))bis(tetrahydro-2*H*-pyran) (**S9d**) (2.06 g, 6.09 mmol, 49%, diastereomer mixture) as a colorless oil.

IR (neat): 2939, 2868, 1962, 1137, 1119, 1076, 1037, 1022, 869 cm^{-1}; Although compound **S9d** should be a mixture of diastereomers, both ^{1}H and ^{13}C NMR spectra do not reflect this but the presence of a single isomer. ^{1}H NMR (400 MHz, $CDCl_3$): δ 5.12-5.08 (m, 2H), 4.62-4.54 (m, 2H), 3.92-3.69 (m, 4H), 3.55-3.33 (m, 4H), 2.10-1.99 (m, 4H), 1.86-1.44 (m, 18H); ^{13}C NMR (100 MHz, $CDCl_3$): δ 204.0, 98.9, 98.8, 91.2, 90.6, 67.4, 66.95, 66.88, 62.31, 62.27, 30.82, 30.80, 29.3, 28.8, 26.0, 25.7, 25.6, 19.71, 19.68; HRMS (ESI): calcd. for ([$C_{20}H_{34}O_4$+Na]$^+$): 361.2349, found: 361.2350.

Deca-4,5-diene-1,10-diol (S10d)

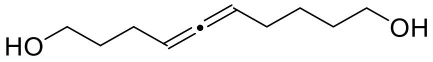

According to the **general procedure F** using protected diol **S9d** (1.76 g, 5.20 mmol, 1.00 equiv.), the crude product was purified by column chromatography on silica gel (hexane/ethyl acetate = 1 : 2 to 1 : 5) and GPC to give deca-4,5-diene-1,10-diol (**S10d**) (842 mg, 4.95 mmol, 95%) as a colorless oil.

IR (neat): 3334, 2935, 2862, 1961, 1441, 1375, 1339, 1058, 876, 694 cm^{-1}; ^{1}H NMR (400 MHz, $CDCl_3$): δ 5.14-5.08 (m, 2H), 3.71-3.64 (m, 4H), 2.15-1.96 (m, 4H), 1.77-1.41 (m, 8H); ^{13}C NMR (100 MHz, $CDCl_3$): δ 204.2, 91.3, 90.7, 62.8, 62.3, 32.2, 32.0, 28.7, 25.3, 25.2; HRMS (ESI): calcd. for ([$C_{10}H_{18}O_2$+Na]$^+$): 193.1199, found: 193.1197.

Deca-4,5-dienedial (1d)

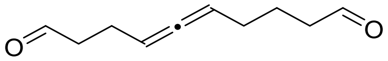

According to the **general procedure G** using diol **S10d** (715 mg, 4.20 mmol, 1.00 equiv.), the crude product was purified by column chromatography on silica gel (hexane/ethyl acetate = 7 : 1 to 5 : 1) to give deca-4,5-dienedial (**1d**) (508 mg, 3.06 mmol, 73%) as a colorless oil.

IR (neat): 2930, 2840, 2725, 1962, 1722, 1409, 1390, 877 cm^{-1}; ^{1}H NMR (400 MHz, $CDCl_3$): δ 9.78 (t, J = 1.4 Hz, 2H), 5.26-5.10 (m, 2H), 2.55 (dt, J = 1.4, 7.3 Hz, 2H), 2.48 (dt, J = 1.4, 7.3 Hz, 2H), 2.36-2.30 (m, 2H), 2.08-1.98 (m, 2H), 1.80-1.69 (m, 2H); ^{13}C NMR (100 MHz, $CDCl_3$): δ 204.3, 202.5, 202.1, 92.0, 90.2, 43.3, 42.5, 28.2, 21.5, 21.4; HRMS (ESI): calcd. for ([$C_{10}H_{14}O_2$+Na]$^+$): 189.0886, found: 189.0886.

Deca-4,5-dienedial bisoxime (4d)

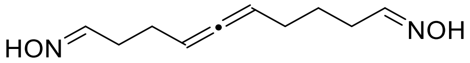

According to the **general procedure H** using bisaldehyde **1d** (312 mg, 1.88 mmol, 1.00 equiv.), the crude product was purified by column chromatography on silica gel (hexane/ethyl acetate = 1 : 1) to

give deca-4,5-dienedial bisoxime (**4d**) (308 mg, 1.60 mmol, 85%, diastereomer mixture) as a colorless solid.

Mp: 68–69 °C, IR (neat): 3198, 3092, 2897, 2860, 1962, 1445, 1327, 931, 705 cm^{-1}; ^{1}H NMR (400 MHz, $CDCl_3$, diastereomer mixture): δ 8.98 (br, 2H), 7.50-7.38 (m, 1H), 6.81-6.70 (m, 1H), 5.20-5.08 (m, 2H), 2.57-2.12 (m, 6H), 2.07-1.99 (m, 2H), 1.65-1.58 (m, 2H); ^{13}C NMR (100 MHz, $CDCl_3$, diastereomer mixture): δ 204.4, 204.3, 152.6, 152.5, 152.3, 152.2, 152.05, 152.02, 151.72, 151.67, 91.6, 91.4, 90.3, 90.1, 90.0, 28.94, 28.91, 28.6, 28.5, 28.2, 28.1, 25.91, 25.87, 25.5, 25.4, 25.2, 24.6, 24.3; HRMS (ESI): calcd. for ($[C_{10}H_{16}N_2O_2+Na]^+$): 219.1104, found: 219.1105.

1,11-Bis((tetrahydro-2*H*-pyran-2-yl)oxy)undec-6-yn-5-ol (S8e)

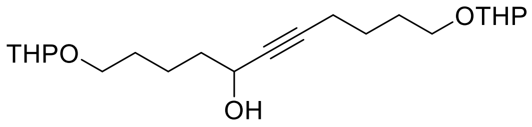

According to the **general procedure D** using alkyne **S2d** (5.47 g, 30.0 mmol, 1.50 equiv.) and aldehyde **S7b** (3.73 g, 20.0 mmol, 1.00 equiv.), the crude product was purified by column chromatography on silica gel (hexane/ethyl acetate = 4 : 1 to 2 : 1) to give 1,11-bis((tetrahydro-2*H*-pyran-2-yl)oxy)undec-6-yn-5-ol (**S8e**) (5.54 g, 15.0 mmol, 75%, diastereomer mixture) as a clear yellow oil.

IR (neat): 3444, 2941, 2867, 1353, 1200, 1137, 1119, 1076, 1033, 1023, 868 cm^{-1}; Although compound **S8e** should be a mixture of diastereomers, both ^{1}H and ^{13}C NMR spectra do not reflect this but the presence of a single isomer. ^{1}H NMR (400 MHz, $CDCl_3$): δ 4.59-4.57 (m, 2H), 4.38-4.34 (m, 1H), 3.89-3.73 (m, 4H), 3.53-3.38 (m, 4H), 2.25 (dt, J = 1.8, 7.1 Hz, 2H), 1.85-1.54 (m, 23H); ^{13}C NMR (100 MHz, $CDCl_3$): δ 99.0, 98.9, 85.2, 81.7, 67.5, 67.1, 62.7, 62.4, 38.1, 30.9, 29.5, 29.0, 25.6, 25.5, 22.14, 22.12, 19.7, 18.7; HRMS (ESI): calcd. for ($[C_{21}H_{36}O_5+Na]^+$): 391.2455, found: 391.2453.

1,11-Bis((tetrahydro-2*H*-pyran-2-yl)oxy)undeca-5,6-diene (S9e)

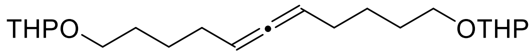

According to the **general procedure E** using propargyl alcohol **S8e** (5.16 g, 14.0 mmol, 1.00 equiv.), the crude product was purified by column chromatography on silica gel (hexane/ethyl acetate = 20 : 1) to give 1,11-bis((tetrahydro-2*H*-pyran-2-yl)oxy)undeca-5,6-diene (**S9e**) (2.45 g, 6.95 mmol, 50%, diastereomer mixture) as a colorless oil.

IR (neat): 2939, 2867, 1961, 1352, 1200, 1137, 1120, 1077, 1035, 1023, 869 cm^{-1}; Although compound **S9e** should be a mixture of diastereomers, both ^{1}H and ^{13}C NMR spectra do not reflect this but the presence of a single isomer. ^{1}H NMR (400 MHz, $CDCl_3$): δ 5.10-5.05 (m, 2H), 4.59-4.57 (m, 2H), 3.89-3.71 (m, 4H), 3.53-3.36 (m, 4H), 2.04-1.98 (m, 4H), 1.87-1.43 (m, 20H); ^{13}C NMR (100 MHz,

CDCl$_3$): δ 204.1, 98.9, 90.9, 67.5, 62.4, 30.9, 29.3, 28.9, 26.0, 25.6, 19.8; HRMS (ESI): calcd. for ([$C_{21}H_{36}O_4$+Na]$^+$): 375.2506, found: 375.2506.

Undeca-5,6-diene-1,11-diol (S10e)

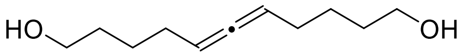

According to the **general procedure F** using protected diol **S9e** (2.19 g, 6.20 mmol, 1.00 equiv.), the crude product was purified by column chromatography on silica gel (CH_2Cl_2/MeOH = 97 : 3 to 95 : 5) to give undeca-5,6-diene-1,11-diol **(S10e)** (1.11 g, 6.02 mmol, 97%) as a colorless oil.

IR (neat): 3334, 2935, 2862, 1961, 1441, 1375, 1339, 1064, 874, 693 cm^{-1}; ^{1}H NMR (400 MHz, CDCl$_3$): δ 5.12-5.07 (m, 2H), 3.65 (t, J = 6.4 Hz, 4H), 2.05-1.99 (m, 4H), 1.66-1.45 (m, 10H); ^{13}C NMR (100 MHz, CDCl$_3$): δ 204.2, 91.0, 62.9, 32.3, 28.7, 25.3; HRMS (ESI): calcd. for ([$C_{11}H_{20}O_2$+Na]$^+$): 207.1356, found: 207.1355.

Undeca-5,6-dienedial (1e)

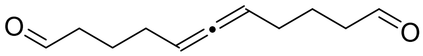

According to the **general procedure G** using diol **S10e** (1.01 g, 5.50 mmol, 1.00 equiv.), the crude product was purified by column chromatography on silica gel (hexane/ethyl acetate = 7 : 1 to 5 : 1) to give undeca-5,6-dienedial (**1e**) (775 mg, 4.30 mmol, 78%) as a colorless oil.

IR (neat): 2938, 2860, 2723, 1961, 1721, 1454, 1392, 877 cm^{-1}; ^{1}H NMR (400 MHz, CDCl$_3$): δ 9.78 (t, J = 1.8 Hz, 2H), 5.12-5.07 (m, 2H), 2.49 (dt, J = 1.8, 7.3 Hz, 4H), 2.07-2.01 (m, 4H), 1.79-1.72 (m, 4H); ^{13}C NMR (100 MHz, CDCl$_3$): δ 204.5, 202.6, 90.7, 43.3, 28.3, 21.6; HRMS (ESI): calcd. for ([$C_{11}H_{16}O_2$+Na]$^+$): 203.1043, found: 203.1046.

Undeca-5,6-dienedial bisoxime (4e)

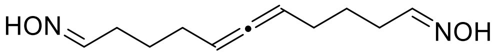

According to the **general procedure H** using bisaldehyde **1e** (297 mg, 1.65 mmol, 1.00 equiv.), the crude product was purified by column chromatography on silica gel (hexane/ethyl acetate = 3 : 2) to give undeca-5,6-dienedial bisoxime (**4e**) (300 mg, 1.43 mmol, 87%, diastereomer mixture) as a colorless solid.

Mp: 70–72 °C, IR (neat): 3210, 3093, 2930, 2859, 1961, 1700, 1665, 1452, 1327, 1284, 924, 888, 708 cm^{-1}; ^{1}H NMR (400 MHz, CD$_3$OD, diastereomer mixture): δ 7.36 (t, J = 6.2 Hz, 1H), 6.65 (t, J = 5.5 Hz, 1H), 5.15-5.10 (m, 2H), 2.41-2.16 (m, 4H), 2.07-2.00 (m, 4H), 1.76-1.56 (m, 4H); ^{13}C NMR (100

MHz, CD_3OD, diastereomer mixture): δ 205.59, 205.57, 152.7, 152.19, 152.17, 91.3, 29.8, 29.6, 29.3, 27.4, 26.7, 25.4; HRMS (ESI): calcd. for ($[C_{11}H_{18}N_2O_2+Na]^+$): 233.1260, found: 233.1258.

1,13-Bis((tetrahydro-2*H*-pyran-2-yl)oxy)tridec-7-yn-6-ol (S8f)

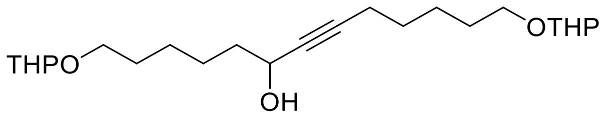

According to the **general procedure D** using alkyne **S2e** (5.89 g, 30.0 mmol, 1.50 equiv.) and aldehyde **S7c** (4.01g, 20.0 mmol, 1.00 equiv.), the crude product was purified by column chromatography on silica gel (hexane/ethyl acetate = 4 : 1 to 2 : 1) to give 1,13-bis((tetrahydro-2*H*-pyran-2-yl)oxy)tridec-7-yn-6-ol (**S8f**) (6.77 g, 17.1 mmol, 86%, diastereomer mixture) as a yellow oil. IR (neat): 3445, 2939, 2863, 1455, 1441, 1353, 1137, 1119, 1077, 1035, 1024 cm^{-1}; Although compound **S8f** should be a mixture of diastereomers, both ^1H and ^{13}C NMR spectra do not reflect this but the presence of a single isomer. ^1H NMR (400 MHz, $CDCl_3$): δ 4.58-4.57 (m, 2H), 4.35 (dt, J = 5.5, 6.4 Hz, 1H), 3.90-3.84 (m, 2H), 3.77-3.72 (m, 2H), 3.53-3.48 (m, 2H), 3.42-3.37 (m, 2H), 2.22 (dt, J = 1.8, 7.3 Hz, 2H), 1.86-1.36 (m, 27H); ^{13}C NMR (100 MHz, $CDCl_3$): δ 98.9, 85.2, 81.6, 67.6, 67.5, 62.6, 62.4, 38.2, 30.8, 29.8, 29.3, 28.6, 26.1, 26.0, 25.6, 25.2, 25.1, 19.7, 18.7; HRMS (ESI): calcd. for ($[C_{23}H_{40}O_5+Na]^+$): 419.2768, found: 419.2771.

1,13-Bis((tetrahydro-2*H*-pyran-2-yl)oxy)trideca-6,7-diene (S9f)

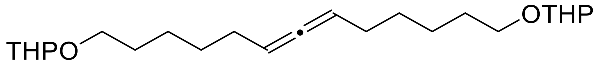

According to the **general procedure E** using propargyl alcohol **S8f** (5.95 g, 15.0 mmol, 1.00 equiv.), the crude product was purified by column chromatography on silica gel (hexane/ethyl acetate = 20 : 1) to give 1,13-bis((tetrahydro-2*H*-pyran-2-yl)oxy)trideca-6,7-diene (**S9f**) (2.38 g, 6.25 mmol, 42%, diastereomer mixture) as a colorless oil.

IR (neat): 2937, 2857, 1961, 1454, 1440, 1352, 1137, 1120, 1078, 1035, 1024 cm^{-1}; Although compound **S9f** should be a mixture of diastereomers, both ^1H and ^{13}C NMR spectra do not reflect this but the presence of a single isomer. ^1H NMR (400 MHz, $CDCl_3$): δ 5.09-5.04 (m, 2H), 4.59-4.57 (m, 2H), 3.90-3.84 (m, 2H), 3.74 (dt, J = 6.9, 9.6 Hz, 2H), 3.53-3.47 (m, 2H), 3.38 (dt, J = 6.9, 9.6 Hz, 2H), 2.04-1.96 (m, 4H), 1.88-1.35 (m, 24H); ^{13}C NMR (100 MHz, $CDCl_3$): δ 204.0, 98.9, 90.9, 67.7, 62.4, 30.9, 29.7, 29.2, 29.1, 25.9, 25.6, 19.8; HRMS (ESI): calcd. for ($[C_{23}H_{40}O_4+Na]^+$): 403.2819, found: 403.2816.

Trideca-6,7-diene-1,13-diol (S10f)

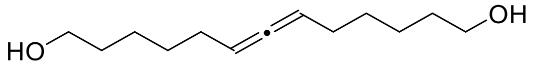

According to the **general procedure F** using protected diol **S9f** (2.06 g, 5.40 mmol, 1.00 equiv.), the crude product was purified by column chromatography on silica gel (CH_2Cl_2/MeOH = 98 : 2 to 97 : 3) to give trideca-6,7-diene-1,13-diol (**S10f**) (1.05 g, 4.95 mmol, 92%) as a colorless oil.
IR (neat): 3334, 2932, 2857, 1961, 1459, 1438, 1073, 1054, 876 cm^{-1}; ^{1}H NMR (400 MHz, $CDCl_3$): δ 5.10-5.05 (m, 2H), 3.64 (t, J = 6.6 Hz, 4H), 2.03-1.97 (m, 4H), 1.68-1.36 (m, 14H); ^{13}C NMR (100 MHz, $CDCl_3$): δ 204.1, 91.0, 63.1, 32.7, 29.0, 25.3; HRMS (ESI): calcd. for ($[C_{13}H_{24}O_2+Na]^+$): 235.1669, found: 235.1669.

Trideca-6,7-dienedial (1f)

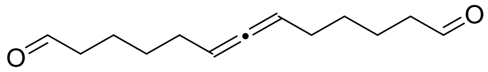

According to the **general procedure G** using diol **S10f** (955 mg, 4.50 mmol, 1.00 equiv.), the crude product was purified by column chromatography on silica gel (hexane/ethyl acetate = 7 : 1) to give trideca-6,7-dienedial (**1f**) (717 mg, 3.44 mmol, 76%) as a colorless oil.
IR (neat): 2934, 2859, 2722, 1960, 1723, 1457, 1391, 877 cm^{-1}; ^{1}H NMR (400 MHz, $CDCl_3$): δ 9.77 (t, J = 1.6 Hz, 2H), 5.10-5.05 (m, 2H), 2.46-2.35 (m, 4H), 2.04-1.98 (m, 4H), 1.71-1.64 (m, 4H), 1.50-1.40 (m, 4H); ^{13}C NMR (100 MHz, $CDCl_3$): δ 204.1, 202.8, 90.8, 43.8, 28.72, 28.67, 21.6; HRMS (ESI): calcd. for ($[C_{13}H_{20}O_2+Na]^+$): 231.1356, found: 231.1353.

4. Preparation of hydroxylamine analogs

(1) Synthesis of *N*-(4-methoxybenzyl)hydroxylamine (S11a)

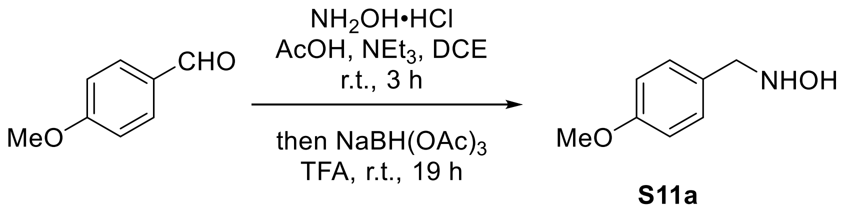

S11a

To a stirred solution of 4-methoxybenzaldehyde (681 µL, 5.00 mmol, 1.00 equiv.) and hydroxylamine hydrochloride (347 mg, 5.00 mmol, 1.00 equiv.) in 1,2-dichloroethane (80.0 mL) were added triethylamine (693 µL, 5.00 mmol, 1.00 equiv.) and acetic acid (572 µL, 10.0 mmol, 2.00 equiv.) at room temperature under argon. After the resultant mixture was stirred at room temperature for 3 h, to the reaction mixture were added sodium triacetoxyborohydride (4.24 g, 20.0 mmol, 4.00 equiv.) and trifluoroacetic acid (2.68 ml, 35.0 mmol, 7.00 equiv.). After the resultant mixture was stirred at room temperature for 19 h, the reaction was quenched with 1 M NaOH aq. and the aqueous layer was

extracted with ethyl acetate three times. The combined organic layer was washed with brine, dried over Na_2SO_4, filtered, and concentrated under reduced pressure. The residue was purified by column chromatography on silica gel (hexane/ethyl acetate = 1 : 2) to give *N*-(4-methoxybenzyl)hydroxylamine (**S11a**) (122 mg, 0.796 mmol, 16%) as a colorless solid.

^1H NMR (400 MHz, $CDCl_3$): δ 7.26-7.19 (m, 4H), 6.89-6.85 (m, 2H), 4.03 (s, 2H), 3.79 (s, 3H); ^{13}C NMR (100 MHz, $CDCl_3$): δ 160.2, 131.3, 124.4, 114.4, 56.6, 55.4.

^1H and ^{13}C NMR spectra were well consistent with those in the previous report[9].

(2) Synthesis of *N*-(4-bromobenzyl)hydroxylamine (S11b)

Compound **S11b** was prepared according to the literature procedure[10].

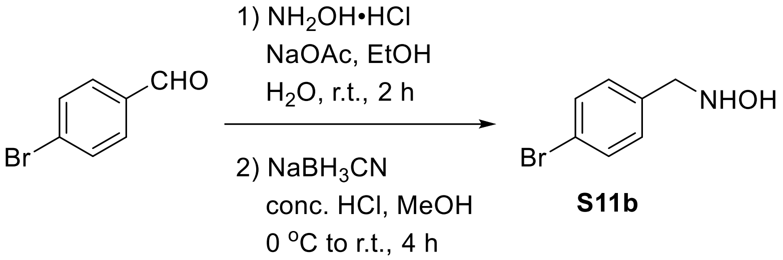

To a stirred solution of 4-bromobenzaldehyde (1.85 g, 10.0 mmol, 1.00 equiv.) and hydroxylamine hydrochloride (834 mg, 12.0 mmol, 1.20 equiv.) in EtOH (10.0 mL) and water (10.0 mL) was added sodium acetate (1.64 g, 20.0 mmol, 2.00 equiv.) at room temperature. After the resultant mixture was stirred at room temperature for 2 h, the reaction mixture was concentrated under reduced pressure. The residue was diluted with ethyl acetate and water, and extracted with ethyl acetate three times. The combined organic layer was dried over Na_2SO_4, filtered, and concentrated under reduced pressure. The residue was used for the next reaction without further purification.

To a stirred solution of oxime (2.00 g, 10.0 mmol, 1.00 equiv.) and sodium cyanoborohydride (754 mg, 12.0 mmol, 1.20 equiv.) in MeOH (20.0 mL) was added dropwise 12 M HCl (8.33 mL, 100 mmol, 10.0 equiv.) at 0 °C under argon. After the resultant mixture was stirred at room temperature for 4 h, the reaction mixture was poured into water and neutralized with KOH at 0 °C. The aqueous layer was extracted with ethyl acetate three times. The combined organic layer was washed with brine, dried over Na_2SO_4, filtered, and concentrated under reduced pressure. The residue was purified by column chromatography on silica gel (hexane/ethyl acetate = 1 : 2) to give *N*-(4-bromobenzyl)hydroxylamine (**S11b**) (1.71 g, 8.46 mmol, 85%) as a colorless solid.

^1H NMR (400 MHz, $CDCl_3$): δ 7.46 (d, J = 8.2 Hz, 2H), 7.19 (d, J = 8.2 Hz, 2H), 5.49 (br, 2H), 3.93 (s, 2H); ^{13}C NMR (100 MHz, $CDCl_3$): δ 136.3, 131.7, 131.0, 121.8, 57.5.

^1H and ^{13}C NMR spectra were well consistent with those in the previous report[10].

(3) Synthesis of *N*-(2-((*t*-butyldiphenylsilyl)oxy)ethyl)hydroxylamine (S11c)

Compound **S11c** was prepared according to the literature procedure[11].

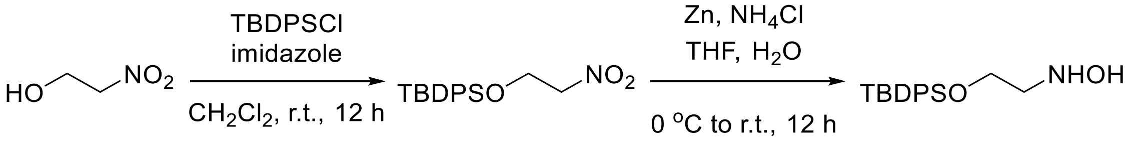

S12a **S11c**

***t*-Butyl(2-nitroethoxy)diphenylsilane (S12a)**

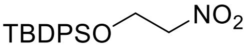

To a stirred solution of 2-nitroethan-1-ol (1.40 mL, 20.0 mmol, 1.00 equiv.) in anhydrous CH_2Cl_2 (40.0 mL) were added *t*-butylchlorodiphenylsilane (6.68 mL, 26.0 mmol, 1.30 equiv.) and imidazole (3.54 g, 52.0 mmol, 2.60 equiv.) at room temperature under argon. After the resultant mixture was stirred at room temperature for 12 h, the reaction was quenched with water and the aqueous layer was extracted with CH_2Cl_2 three times. The combined organic layer was washed with brine, dried over Na_2SO_4, filtered, and concentrated under reduced pressure. The residue was purified by column chromatography on silica gel (hexane/ethyl acetate = 20 : 1) to give *t*-butyl(2-nitroethoxy)diphenylsilane (**S12a**) (6.32 g, 19.2 mmol, 96%) as a colorless solid.

Mp: 78–79 °C, IR (neat): 2958, 2932, 2859, 1558, 1428, 1365, 1114, 738, 703 cm^{-1}; ^1H NMR (400 MHz, $CDCl_3$): δ 7.65-7.63 (m, 4H), 7.47-7.38 (m, 6H), 4.47-4.44 (m, 2H), 4.16-4.13 (m, 2H), 1.03 (s, 9H); ^{13}C NMR (100 MHz, $CDCl_3$): δ 135.7, 132.7, 130.2, 128.0, 77.5, 60.2, 26.7, 19.3; HRMS (ESI): calcd. for ($[C_{18}H_{23}NO_3Si + Na]^+$): 352.1339, found: 352.1337.

***N*-(2-((*t*-Butyldiphenylsilyl)oxy)ethyl)hydroxylamine (S11c)**

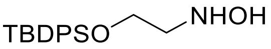

To a stirred solution of *t*-butyl(2-nitroethoxy)diphenylsilane (**S12a**) (2.31 g, 7.00 mmol, 1.00 equiv.) in anhydrous THF (21.0 mL) and water (3.50 mL) was added portion-wise Zn powder (961 mg, 14.7 mmol, 2.10 equiv., activated with 10% HCl aq., preliminarily) at 0 °C under argon. To a suspension was added dropwise a solution of NH_4Cl (3.00 g, 56.0 mmol, 8.00 equiv.) in water (10.5 mL) over 10 min. After the resultant mixture was stirred at room temperature for 12 h, the reaction was quenched with sat. $NaHCO_3$ aq. and the aqueous layer was extracted with ethyl acetate three times. The combined organic layer was washed with brine, dried over Na_2SO_4, filtered, and concentrated under reduced pressure. The residue was purified by column chromatography on silica gel (hexane/ethyl acetate = 2 : 1) to give *N*-(2-((*t*-butyldiphenylsilyl)oxy)ethyl)hydroxylamine (**S11c**) (1.59 g, 5.04 mmol, 72%) as a colorless solid.

^{1}H NMR (400 MHz, $CDCl_3$): δ 7.69-7.66 (m, 4H), 7.45-7.36 (m, 6H), 5.73 (br, 2H), 3.83 (t, J = 5.0 Hz, 2H), 3.06 (t, J = 5.0 Hz, 2H), 1.06 (s, 9H); ^{13}C NMR (100 MHz, $CDCl_3$): δ 135.7, 133.6, 129.9, 127.9, 60.3, 55.8, 27.0, 19.4.
^{1}H and ^{13}C NMR spectra were well consistent with those in the previous report[12].

(4) Synthesis of *N*-(2-((tetrahydro-2*H*-pyran-2-yl)oxy)ethyl)hydroxylamine (S11d)

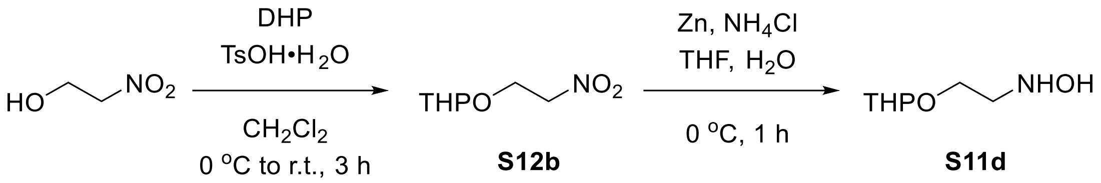

2-(2-Nitroethoxy)tetrahydro-2*H*-pyran (S12b)

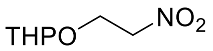

To a stirred solution of 2-nitroethan-1-ol (1.05 mL, 15.0 mmol, 1.00 equiv.) in anhydrous CH_2Cl_2 (30.0 mL) was added 3,4-dihydro-2*H*-pyran (1.49 mL, 16.5 mmol, 1.10 equiv.) and *p*-toluenesulfonic acid monohydrate (143 mg, 0.750 mmol, 5.00 mol%) at 0 °C under argon. After the resultant mixture was stirred at room temperature for 3 h, the reaction was quenched with sat. $NaHCO_3$ aq. and the aqueous layer was extracted with CH_2Cl_2 three times. The combined organic layer was washed with brine, dried over Na_2SO_4, filtered, and concentrated under reduced pressure. The residue was purified by column chromatography on silica gel (hexane/ethyl acetate = 7 : 1 to 5 : 1) to give 2-(2-nitroethoxy)tetrahydro-2*H*-pyran (**S12b**) (2.11 g, 12.0 mmol, 80%) as a pale yellow oil.
IR (neat): 2945, 2873, 1558, 1375, 1201, 1126, 1076, 1037 cm^{-1}; ^{1}H NMR (400 MHz, $CDCl_3$): δ 4.67 (t, J = 3.2 Hz, 1H), 4.59-4.55 (m, 2H), 4.25-4.20 (m, 1H), 4.02-3.96 (m, 1H), 3.83-3.77 (m, 1H), 3.57-3.51 (m, 1H), 1.82-1.50 (m, 6H); ^{13}C NMR (100 MHz, $CDCl_3$): δ 99.1, 75.3, 63.1, 62.2, 30.3, 25.4, 19.0; HRMS (ESI): calcd. for ($[C_7H_{13}NO_4+Na]^+$): 198.0737, found: 198.0734.

***N*-(2-((Tetrahydro-2*H*-pyran-2-yl)oxy)ethyl)hydroxylamine (S11d)**

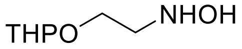

To a stirred solution of 2-(2-nitroethoxy)tetrahydro-2*H*-pyran (**S12b**) (876 mg, 5.00 mmol, 1.00 equiv.) in anhydrous THF (15.0 mL) and water (2.50 mL) was added portion-wise Zn powder (686 mg, 10.5 mmol, 2.10 equiv., activated with 10% HCl aq., preliminarily) at 0 °C under argon. To a suspension was added dropwise a solution of NH_4Cl (2.14 g, 40.0 mmol, 8.00 equiv.) in water (7.50 mL) over 10 min. After the resultant mixture was stirred at 0 °C for 1 h, the reaction was quenched

with sat. $NaHCO_3$ aq. and the aqueous layer was extracted with CH_2Cl_2 three times. The combined organic layer was washed with brine, dried over Na_2SO_4, filtered, and concentrated under reduced pressure. The residue was purified by column chromatography on silica gel (ethyl acetate) to give *N*-(2-((tetrahydro-2*H*-pyran-2-yl)oxy)ethyl)hydroxylamine (**S11d**) (402 mg, 2.49 mmol, 50%) as a blue oil.

IR (neat): 3396, 3270, 2942, 2871, 1135, 1120, 1076, 1033, 980, 870 cm^{-1}; ^{1}H NMR (400 MHz, $CDCl_3$): δ 6.07 (br, 2H), 4.62-4.60 (m, 1H), 3.93-3.85 (m, 2H), 3.66-3.61 (m, 1H), 3.55-3.50 (m, 1H), 3.14 (t, J = 5.3 Hz, 2H), 1.86-1.70 (m, 2H), 1.62-1.51 (m, 4H); ^{13}C NMR (100 MHz, $CDCl_3$): δ 99.4, 63.9, 62.7, 53.6, 30.7, 25.5, 19.8; HRMS (ESI): calcd. for ($[C_7H_{15}NO_3+Na]^+$): 184.0944, found: 184.0948.

(5) Synthesis of *N*-(furan-3-ylmethyl)hydroxylamine (S11e)

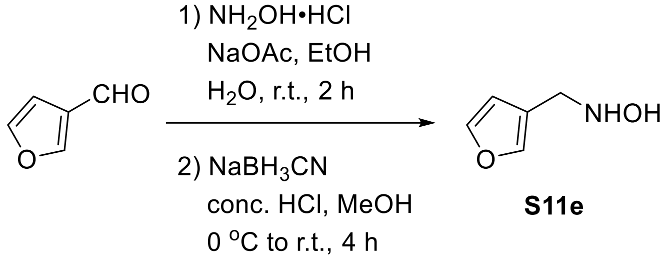

To a stirred solution of 3-furaldehyde (836 μL, 10.0 mmol, 1.00 equiv.) and hydroxylamine hydrochloride (834 mg, 12.0 mmol, 1.20 equiv.) in EtOH (10.0 mL) and water (10.0 mL) was added sodium acetate (1.64 g, 20.0 mmol, 2.00 equiv.) at room temperature. After the resultant mixture was stirred at room temperature for 2 h, the reaction mixture was concentrated under reduced pressure. The residue was diluted with ethyl acetate and water, and extracted with ethyl acetate three times. The combined organic layer was dried over Na_2SO_4, filtered, and concentrated under reduced pressure. The residue was used for the next reaction without further purification.

To a stirred solution of oxime (1.11 g, 10.0 mmol, 1.00 equiv.) and sodium cyanoborohydride (754 mg, 12.0 mmol, 1.20 equiv.) in MeOH (20.0 mL) was added dropwise 12 M HCl (8.33 mL, 100 mmol, 10.0 equiv.) at 0 °C under argon. After the resultant mixture was stirred at room temperature for 4 h, the reaction mixture was poured into water and neutralized with KOH at 0 °C. The aqueous layer was extracted with ethyl acetate three times. The combined organic layer was washed with brine, dried over Na_2SO_4, filtered, and concentrated under reduced pressure. The residue was purified by column chromatography on silica gel (CH_2Cl_2/MeOH = 98 : 2) to give *N*-(furan-3-ylmethyl)hydroxylamine (**S11e**) (268 mg, 2.37 mmol, 24%) as a yellow oil.

^{1}H NMR (400 MHz, $CDCl_3$): δ 7.41-7.25 (m, 2H), 6.41 (d, J = 0.9 Hz, 1H), 5.85 (br, 2H), 3.90 (s, 2H); ^{13}C NMR (100 MHz, $CDCl_3$): δ 143.3, 141.1, 120.9, 111.0, 48.6.

^{1}H and ^{13}C NMR spectra were well consistent with those in the previous report[10].

(6) Synthesis of methyl N^2-(*t*-butoxycarbonyl)-N^6-hydroxy-L-lysinate (S11f)

Compound **S11f** was prepared according to a modified literature procedure[13,14]

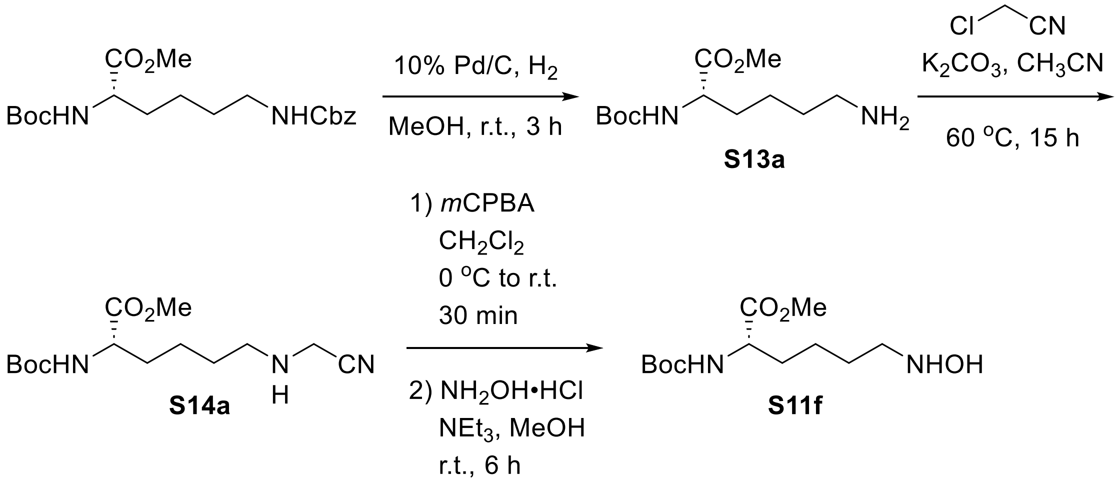

Methyl (*t*-butoxycarbonyl)-L-lysinate (S13a)

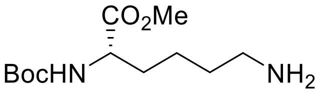

To a stirred solution of methyl N^6-((benzyloxy)carbonyl)-N^2-(*t*-butoxycarbonyl)-L-lysinate (1.97 g, 5.00 mmol, 1.00 equiv.) in MeOH (50.0 mL) was added Pd/C (10% Pd, 197 mg, 0.185 mmol, 3.70 mol%) at room temperature under argon. After the resultant mixture was stirred at room temperature for 3 h under hydrogen atmosphere (balloon), the reaction mixture was filtered through a pad of celite® and eluted with ethyl acetate. The filtrate was concentrated under reduced pressure to give methyl (*t*-butoxycarbonyl)-L-lysinate (**S13a**) (1.18 g, 4.53 mmol, 91%) as a pale yellow oil. The crude product was sufficiently pure and it was used for the next reaction without further purification.

^{1}H NMR (400 MHz, $CDCl_3$): δ 5.06-5.05 (m, 1H), 4.31-4.28 (m, 1H), 3.74 (s, 3H), 2.69 (t, J = 6.6 Hz, 2H), 1.86-1,77 (m, 1H), 1.68-1.59 (m, 1H), 1.52-1.35 (m, 15H); ^{13}C NMR (100 MHz, $CDCl_3$): δ 173.5, 155.5, 80.0, 53.5, 52.3, 42.0, 33.3, 32.7, 28.4, 22.7.

^{1}H and ^{13}C NMR spectra were well consistent with those in the previous report[15].

Methyl N^2-(*t*-butoxycarbonyl)-N^6-(cyanomethyl)-L-lysinate (S14a)

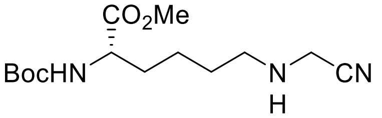

To a stirred solution of methyl (*t*-butoxycarbonyl)-L-lysinate (**S13a**) (1.09 g, 4.19 mmol, 1.00 equiv.) in acetonitrile (63.0 mL) were added potassium carbonate (1.39 g, 10.1 mmol, 2.40 equiv.) and

chloroacetonitrile (475 μL, 7.54 mmol, 1.80 equiv.) at room temperature under argon. After the resultant mixture was stirred at 60 °C for 18 h, the mixture was cooled to room temperature, filtered through a pad of celite®, and eluted with ethyl acetate. The filtrate was concentrated under reduced pressure. The residue was purified by column chromatography on silica gel (hexane/ethyl acetate = 1 : 1 to 1 : 2) to give methyl *N*2-(*t*-butoxycarbonyl)-*N*6-(cyanomethyl)- L-lysinate (**S14a**) (911 mg, 3.04 mmol, 73%) as a brown oil.

IR (neat): 3335, 2933, 2863, 1746, 1715, 1699, 1541, 1521, 1507, 1365, 1166 cm^{-1}; $[\alpha]^{25}_{D}$ = -23.2 (c 0.240, MeOH); ^1H NMR (400 MHz, $CDCl_3$): δ 5.05-5.04 (m, 1H), 4.31-4.30 (m, 1H), 3.75 (s, 3H), 3.59 (s, 2H), 2.73 (t, *J* = 6.9 Hz, 2H), 1.87-1.80 (m, 1H), 1.69-1.26 (m, 15H); ^{13}C NMR (100 MHz, $CDCl_3$): δ 173.4, 155.5, 117.9, 80.1, 53.3, 52.4, 48.6, 37.4, 32.7, 29.0, 28.4, 22.9; HRMS (ESI): calcd. for ($[C_{14}H_{25}N_3O_4+Na]^+$): 322.1737, found: 322.1735.

Methyl *N*2-(*t*-butoxycarbonyl)-*N*6-hydroxy- L-lysinate (S11f)

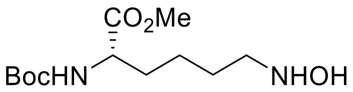

A stirred solution of **S14a** (868 mg, 2.90 mmol, 1.00 equiv.) in anhydrous CH_2Cl_2 (29.0 mL) under argon was cooled to 0 °C, and *m*CPBA was added in five portions (286 mg, 1.16 mmol each) at 5 min intervals. After the resultant mixture was stirred an additional 30 min, cooled to 0 °C, and $Na_2S_2O_3$ (1.49 g, 6.00 mmol) in water (6.00 mL) and sat. $NaHCO_3$ aq. (12.0 mL) were added. Stirring was continued until the mixture became homogeneous and CH_2Cl_2 (30.0 mL) and $NaHCO_3$ (20.0 mL) were added. The aqueous layer extracted with CH_2Cl_2 three times. The combined organic layer was washed with brine, dried over Na_2SO_4, filtered, and concentrated under reduced pressure. The crude nitrone was used for the next reaction without further purification.

To a stirred solution of crude nitrone (909 mg, 2.90 mmol, 1.00 equiv.) in MeOH (73.0 mL) was added triethylamine (2.01 mL, 14.5 mmol, 5.00 equiv.) and hydroxylamine hydrochloride (1.01 g, 14.5 mmol, 5.00 equiv.) at room temperature. After the resultant mixture was stirred at room temperature for 6 h, the reaction was quenched with sat. $NaHCO_3$ aq. at 0 °C and concentrated under reduced pressure. The residue was diluted with CH_2Cl_2 and water, and extracted with a solution of CH_2Cl_2 three times. The organic layer was dried over Na_2SO_4, filtered, and concentrated under reduced pressure. The residue was purified by column chromatography on silica gel (CH_2Cl_2/MeOH = 93 : 7) to give methyl *N*2-(*t*-butoxycarbonyl)-*N*6-hydroxy- L-lysinate (**S11f**) (266 mg, 0.963 mmol, 33% in 2 steps) as colorless solid.

This compound was immediately used for the next reaction because it was not suitable for the long-term storage.

Mp: 47–50 °C, IR (neat): 3362, 2935, 1743, 1699, 1521, 1508, 1366, 1165 cm^{-1}; $[\alpha]^{26}_D$ = -26.0 (c 0.220, MeOH); ^{1}H NMR (400 MHz, $CDCl_3$): δ 5.15-5.13 (m, 1H), 4.32 (br, 1H), 3.74 (s, 3H), 2.99-2.87 (m, 2H), 1.83-1.79 (m, 1H), 1.72-1.40 (m, 16H); ^{13}C NMR (100 MHz, $CDCl_3$): δ 173.5, 155.6, 80.0, 53.4, 53.3, 52.4, 32.7, 28.4, 26.5, 22.9; HRMS (ESI): calcd. for ($[C_{12}H_{24}N_2O_5+Na]^+$): 299.1577, found: 299.1577.

^{1}H and ^{13}C NMR spectra were well consistent with those in the previous report[10].

(7) Synthesis of *t*-butyl *N*^{2}-(((9*H*-fluoren-9-yl)methoxy)carbonyl)-*N*^{6}-hydroxy-L-lysinate (S11g)

Compound **S11g** was prepared according to a modified literature procedure[13,14].

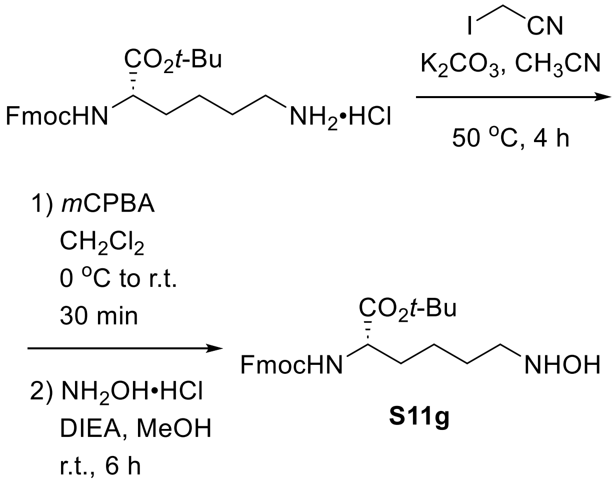

***t*-Butyl *N*^{2}-(((9*H*-fluoren-9-yl)methoxy)carbonyl)-*N*^{6}-(cyanomethyl)-L-lysinate (S14b)**

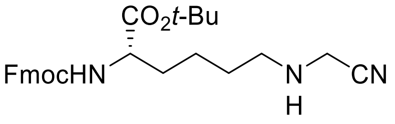

To a stirred solution of *t*-butyl (((9*H*-fluoren-9-yl)methoxy)carbonyl)-L-lysinate hydrochloride (2.31 g, 5.00 mmol, 1.00 equiv.) in acetonitrile (74.6 mL) were added potassium carbonate (1.38 g, 10.0 mmol, 2.00 equiv.) and iodoacetonitrile (542 μL, 7.50 mmol, 1.50 equiv.) at room temperature under argon. After the resultant mixture was stirred at 50 °C for 4 h, the reaction mixture was cooled to room temperature and concentrated under reduced pressure. The residue was diluted with CH_2Cl_2 and sat. $NaHCO_3$ aq., and extracted with CH_2Cl_2 three times. The combined organic layer was washed with brine, dried over Na_2SO_4, filtered, and concentrated under reduced pressure. The crude product was purified by column chromatography on silica gel (hexane/ethyl acetate = 2 : 1 to 1 : 1) to give *t*-butyl *N*^{2}-(((9*H*-fluoren-9-yl)methoxy)carbonyl)-*N*^{6}-(cyanomethyl)-L-lysinate (**S14b**) (1.75 g, 3.77 mmol, 75%) as a yellow oil.

IR (neat): 3330, 2976, 2935, 2863, 1715, 1522, 1452, 1250, 1228, 1156, 760, 741 cm^{-1}; $[\alpha]^{26}_D$ = -22.6 (c 0.405, MeOH); ^1H NMR (400 MHz, $CDCl_3$): δ 7.75 (d, *J* = 7.8 Hz, 2H), 7.60 (d, *J* = 7.3 Hz, 2H), 7.39 (t, *J* = 7.8 Hz, 2H), 7.30 (t, *J* = 7.3 Hz, 2H), 5.50 (d, *J* = 8.2 Hz, 1H), 4.40 (d, *J* = 6.9 Hz, 2H), 4.27-4.19 (m, 2H), 3.50 (s, 2H), 2.68 (t, *J* = 6.6 Hz, 2H), 1.86-1.79 (m, 1H), 1.69-1.30 (m, 15H); ^{13}C NMR (100 MHz, $CDCl_3$): δ 171.6, 156.0, 144.0, 143.9, 141.3, 127.7, 127.1, 125.1, 120.0, 117.9, 82.1, 66.8, 54.2, 48.4, 47.2, 37.2, 32.5, 28.9, 28.0, 22.6; HRMS (ESI): calcd. for ($[C_{27}H_{33}N_3O_4+Na]^+$): 486.2363, found: 486.2362.

_t_-Butyl _N_²-(((9_H_-fluoren-9-yl)methoxy)carbonyl)-_N_⁶-hydroxy-L-lysinate (S11g)

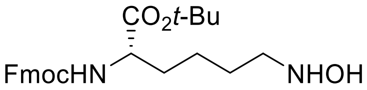

A stirred solution of **S14b** (1.69 g, 3.65 mmol, 1.00 equiv.) in anhydrous CH_2Cl_2 (29.0 mL) was added *m*CPBA (1.80 g, 7.30 mmol, 2.00 equiv.) in five portions at 0 °C under argon. After the resultant mixture was stirred at 0 °C for 30 min, the reaction was quenched with $Na_2S_2O_3$ (1.86 g, 7.50 mmol) in water (7.50 mL) and sat. $NaHCO_3$ aq. (15.0 mL). After the solution became homogeneous, the reaction mixture was diluted with CH_2Cl_2 (10.0 mL) and $NaHCO_3$ (25.0 mL). The aqueous layer was extracted with CH_2Cl_2 three times. The combined organic layer was washed with brine, dried over Na_2SO_4, filtered, and concentrated under reduced pressure. The crude nitrone was used for the next reaction without further purification.

To a solution of crude nitrone (1.74 g, 3.65 mmol, 1.00 equiv.) in MeOH (92.0 mL) were added *N*,*N*-diisopropylethylamine (3.11 mL, 18.3 mmol, 5.00 equiv.) and hydroxylamine hydrochloride (1.27 g, 18.3 mmol, 5.00 equiv.) at room temperature. After the resultant mixture was stirred at room temperature for 6 h, the reaction mixture was concentrated under reduced pressure. The residue was diluted with CH_2Cl_2 and sat. NH_4Cl aq., and extracted with CH_2Cl_2 three times. The combined organic layer was dried over Na_2SO_4, filtered, and concentrated under reduced pressure. The crude product was purified by column chromatography on silica gel (hexane/ethyl acetate = 2 : 3 to 1 : 4) to give *t*-butyl *N*²-(((9*H*-fluoren-9-yl)methoxy)carbonyl)-*N*⁶-hydroxy-L-lysinate (**S11g**) (792 mg, 1.80 mmol, 49% in 2 steps) as colorless solid.

This compound was immediately used for the next reaction because it was not suitable for the long-term storage.

Mp: 57–59 °C, IR (neat): 3325, 2976, 2938, 2863, 1716, 1523, 1450, 1249, 1230, 1155, 760, 741 cm^{-1}; $[\alpha]^{26}_D$ = -22.4 (c 1.03, MeOH); ^1H NMR (400 MHz, CD_3OD): δ 7.76 (d, *J* = 7.8 Hz, 2H), 7.65 (t, *J* = 7.3 Hz, 2H), 7.36 (t, *J* = 7.3 Hz, 2H), 7.28 (t, *J* = 7.8 Hz, 2H), 4.42-4.28 (m, 3H), 4.18 (t, *J* = 6.9 Hz, 1H), 4.06-4.03 (m, 1H), 2.84 (t, *J* = 7.3 Hz, 2H), 1.92-1.41 (m, 17H); ^{13}C NMR (100 MHz,

CD_3OD): δ 173.5, 158.6, 145.3, 145.1, 142.5, 128.7, 128.1, 126.22, 126.17, 120.9, 82.6, 67.8, 56.1, 54.4, 32.5, 28.3, 27.4, 24.5; HRMS (ESI): calcd. for ($[C_{25}H_{32}N_2O_5+H]^+$): 441.2384, found: 441.2382.

5. Optimal conditions for (3+2)/(3+2) cycloaddition of nitrone (Table 1, entry 4 in main text)

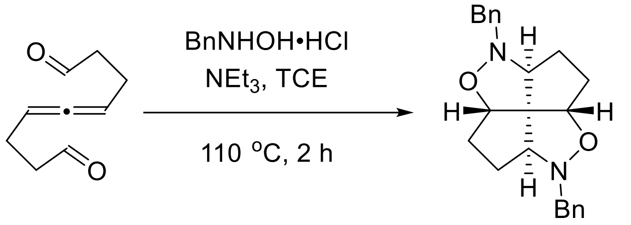

To a stirred solution of nona-4,5-dienedial (**1a**) (6.09 mg, 0.0400 mmol, 1.00 equiv.) in 1,1,2-trichloroethane (8.00 mL) were added *N*-benzylhydroxylamine hydrochloride (16.0 mg, 0.100 mmol, 2.50 equiv.) and triethylamine (13.9 µL, 0.100 mmol, 2.50 equiv.) at room temperature under argon. After the resultant mixture was stirred at 110 °C for 2 h, the reaction was quenched with sat. $NaHCO_3$ aq. at room temperature and the aqueous layer was extracted with CH_2Cl_2 three times. The combined organic layer was washed with brine, dried over Na_2SO_4, filtered, and concentrated under reduced pressure. The residue was purified by preparative TLC (hexane/ethyl acetate = 4 : 1) to give *c*,*c*,*c*,*c*-[5.5.5.5]-*N*,*N'*-dibenzyl-2,8-dioxa-3,9-diazafenestrane (**2a**) (13.2 mg, 0.0364 mmol, 91%) as a colorless solid.

Mp: 128–129 °C, IR (neat): 2929, 2855, 1496, 1456, 1351, 1339, 1052, 1030, 950, 750, 697 cm^{-1}; ^1H NMR (400 MHz, $CDCl_3$): δ 7.37-7.23 (m, 10H), 4.31 (d, J = 5.5 Hz, 2H), 3.88 (s, 4H), 3.05 (d, J = 5.5 Hz, 2H), 2.17-2.07 (m, 2H), 2.04-1.92 (m, 4H), 1.59-1.52 (m, 2H); ^{13}C NMR (100 MHz, $CDCl_3$): δ 137.5, 128.8, 128.4, 127.4, 86.8, 79.6, 61.1, 29.7, 28.0; HRMS (ESI): calcd. for ($[C_{23}H_{26}N_2O_2+Na]^+$): 385.1886, found: 385.1886.

6. Typical procedure for optimization of (3+2)/(3+2) cycloaddition of nitrile oxide (Table 2, entry 12 in main text)

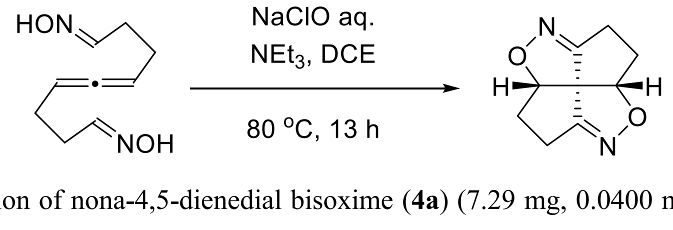

To a stirred solution of nona-4,5-dienedial bisoxime (**4a**) (7.29 mg, 0.0400 mmol, 1.00 equiv.) in 1,2-dichloroethane (8.00 mL) were added 12wt% NaOCl aq. (248 µL, 0.400 mmol, 10.0 equiv.) and triethylamine (55.4 µL, 0.400 mmol, 10.0 equiv.) at room temperature under argon. After the resultant mixture was stirred at 80 °C for 13 h, the reaction mixture was cooled to room temperature, diluted with water, and the aqueous layer was extracted with CH_2Cl_2 three times. The combined organic layer was washed with brine, dried over Na_2SO_4, filtered, and concentrated under reduced pressure. The

residue was purified by preparative TLC (hexane/ethyl acetate = 2 : 5) to give *c*,*c*-[5.5.5.5]-3,9-dioxa-1,7-diiminofenestrane (**5a**) (4.50 mg, 0.0253 mmol, 72%) as a colorless solid.

Mp: 129–131 °C, IR (neat): 2929, 2854, 1733, 1647, 1436, 1288, 1036, 896, 841, 772 cm^{-1}; ^{1}H NMR (400 MHz, $CDCl_3$): δ 5.06 (d, J = 5.0 Hz, 2H), 2.71-2.64 (m, 2H), 2.58-2.46 (m, 4H), 2.25-2.15 (m, 2H); ^{13}C NMR (100 MHz, $CDCl_3$): δ 163.7, 88.9, 85.2, 35.6, 21.6; HRMS (ESI): calcd. for ($[C_9H_{10}N_2O_2+Na]^+$): 201.0634, found: 201.0632.

7. Optimization of reaction conditions

Supplementary Table 1 Optimization of (3+2) cycloaddition conditions via nitrone formation.

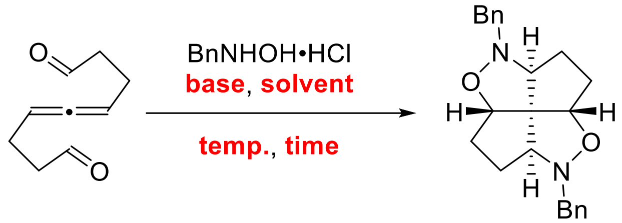

entry	base	solvent	temp. (°C)	time (h)	isolated yield (%)
1	none	EtOH	50	4	complex mixture
2	none	toluene	80	2	no reaction[a]
3	none	toluene	110	2	57
4	none	toluene	110	5	56
5	none	1,4-dioxane	100	2	75
6	none	TCE[b]	110	2	89
7	NEt_3	toluene	110	2	74
8	NEt_3	1,4-dioxane	100	2	58
9	NEt_3	TCE	110	2	91
10	DIEA	TCE	110	2	19
11	pyridine	TCE	110	2	73
12	$NaHCO_3$	TCE	110	2	77

[a] Determined by TLC analysis. [b] 1,1,2-trichloroethane.

Supplementary Table 2 Optimization of (3+2) cycloaddition conditions via nitrile oxide formation.

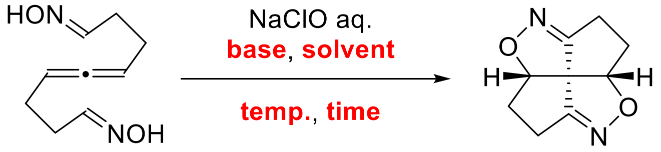

entry	base	solvent	temp. (°C)	time (h)	isolated yield (%)
1	NEt_3	CH_2Cl_2	r.t.	8	59
2	NEt_3	CH_2Cl_2	r.t.	13	69
3	NEt_3	CH_2Cl_2	r.t.	18	62
4	NEt_3	$CHCl_3$	r.t.	13	45
5	NEt_3	EtOH	r.t.	13	0
6	NEt_3	THF	r.t.	13	15
7	NEt_3	toluene	r.t.	13	20
8	pyridine	CH_2Cl_2	r.t.	13	50
9	$NaHCO_3$	CH_2Cl_2/H_2O = 5 : 1	r.t.	13	12
10	DIEA	CH_2Cl_2	r.t.	13	59
11	none	CH_2Cl_2	r.t.	13	62
12	NEt_3	CH_2Cl_2	0	13	28
13	NEt_3	CH_2Cl_2	40	13	66
14	NEt_3	DCE[a]	60	13	63
15	NEt_3	DCE	80	13	72
16	NEt_3	TCE	100	13	66
17	NEt_3	DCE	80	2	63

[a] 1,2-dichloroethane.

Supplementary Table 3 Examination of amount of oxidant and base for nitrile oxide formation.

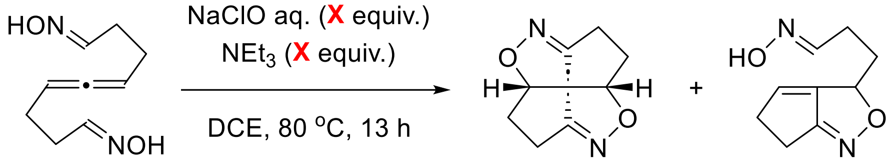

4a 5a S15a

entry	X (equiv.)	ratio[a]			isolated yield
		**5a**	**S15a**	**4a**	of **5a** (%)
1	3.00	0.60	0.12	0.28	35
2	5.00	0.84	0.08	0.08	51
3	10.0	0.93	0.07	0	72
4	15.0	0.93	0.07	0	46

[a] Determined by ^{1}H NMR analysis of the reaction mixture.

8. General procedure for cycloaddition via nitrone

Method A:

To a stirred solution of allene bisaldehyde **1** (0.250 mmol, 1.00 equiv.) in 1,1,2-trichloroethane (50.0 mL) were added hydroxylamine hydrochloride **S11** (0.625 mmol, 2.50 equiv.) and triethylamine (86.6 μL, 0.625 mmol, 2.50 equiv.) at room temperature under argon. After the resultant mixture was stirred at 110 °C for 2 h, the reaction was quenched with water at room temperature and the aqueous layer was extracted with CH_2Cl_2 three times. The combined organic layer was washed with brine, dried over Na_2SO_4, filtered, and concentrated under reduced pressure. The residue was purified by column chromatography on silica gel or preparative TLC to give the corresponding fenestrane **2**.

Method B:

To a stirred solution of allene bisaldehyde **1** (0.250 mmol, 1.00 equiv.) in 1,1,2-trichloroethane (50.0 mL) was added hydroxylamine **S11** (0.625 mmol, 2.50 equiv.) at room temperature under argon. After the resultant mixture was stirred at 110 °C for 2 h, the reaction mixture was cooled to room temperature and concentrated under reduced pressure. The residue was purified by column chromatography on silica gel to give the corresponding fenestrane **2**.

***c,c,c,c*-[5.5.5.5]-*N,N'*-Dimethyl-2,8-dioxa-3,9-diazafenestrane (2b)**

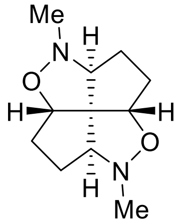

Reaction conditions: method A

Allene bisaldehyde: nona-4,5-dienedial (**1a**) (38.0 mg, 0.250 mmol, 1.00 equiv.)

Hydroxylamine hydrochloride: *N*-methylhydroxylamine hydrochloride (52.2 mg, 0.625 mmol, 2.50 equiv.)

Purification method: column chromatography on silica gel (hexane/ethyl acetate = 1 : 4 to 1 : 9)

Yield: 33.6 mg, 0.160 mmol, 64%

Colorless solid

Mp: 66–68 °C, IR (neat): 2953, 2930, 2850, 1459, 1433, 1353, 1342, 1078, 1050, 945 cm^{-1}; ^{1}H NMR (400 MHz, $CDCl_3$): δ 4.30 (d, J = 5.5 Hz, 2H), 2.81 (d, J = 5.0 Hz, 2H), 2.63 (s, 6H), 2.17-1.96 (m, 6H), 1.76-1.72 (m, 2H); ^{13}C NMR (100 MHz, $CDCl_3$): δ 87.9, 87.1, 81.4, 43.6, 29.8, 27.6; HRMS (ESI): calcd. for ($[C_{11}H_{18}N_2O_2+H]^+$): 211.1441, found: 211.1446.

***c,c,c,c*-[5.5.5.5]-*N,N'*-Diisopropyl-2,8-dioxa-3,9-diazafenestrane (2c)**

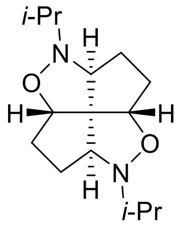

Reaction conditions: method A

Allene bisaldehyde: nona-4,5-dienedial (**1a**) (38.0 mg, 0.250 mmol, 1.00 equiv.)

Hydroxylamine hydrochloride: *N*-isopropylhydroxylamine hydrochloride (69.7 mg, 0.625 mmol, 2.50 equiv.)

Purification method: column chromatography on silica gel (hexane/ethyl acetate = 3 : 1)

Yield: 47.8 mg, 0.179 mmol, 72%

Colorless solid

Mp: 57–58 °C, IR (neat): 2971, 2931, 2880, 1540, 1456, 1336, 1044, 956 cm^{-1}; ^{1}H NMR (400 MHz, $CDCl_3$): δ 4.21 (d, J = 5.0 Hz, 2H), 3.14 (d, J = 5.5 Hz, 2H), 2.86-2.80 (m, 2H), 2.19-2.04 (m, 4H), 2.01-1.92 (m, 2H), 1.88-1.79 (m, 2H), 1.16 (d, J = 6.4 Hz, 6H), 1.09 (d, J = 6.4 Hz, 6H); ^{13}C NMR (100 MHz, $CDCl_3$): δ 88.1, 85.5, 78.5, 57.7, 29.9, 29.8, 20.71, 20.66; HRMS (ESI): calcd. for ($[C_{15}H_{26}N_2O_2+Na]^+$): 289.1886, found: 289.1884.

***c,c,c,c*-[5.5.5.5]-*N,N'*-Dicyclohexyl-2,8-dioxa-3,9-diazafenestrane (2d)**

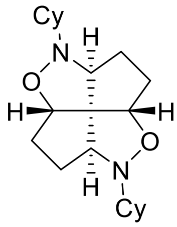

Reaction conditions: method A

Allene bisaldehyde: nona-4,5-dienedial (**1a**) (38.0 mg, 0.250 mmol, 1.00 equiv.)

Hydroxylamine hydrochloride: *N*-cyclohexylhydroxylamine hydrochloride (94.8 mg, 0.625 mmol, 2.50 equiv.)

Purification method: column chromatography on silica gel (hexane/ethyl acetate = 7 : 1)

Yield: 66.2 mg, 0.191 mmol, 76%

Colorless solid

Mp: 84–87 °C, IR (neat): 2929, 2854, 1345, 1149, 1087, 1063, 1050, 1037, 957 cm^{-1}; ^{1}H NMR (400 MHz, $CDCl_3$): δ 4.18 (d, *J* = 4.6 Hz, 2H), 3.17 (d, *J* = 5.5 Hz, 2H), 2.53-2.48 (m, 2H), 2.17-2.03 (m, 6H), 1.99-1.75 (m, 10H), 1.61 (d, *J* = 11.4 Hz, 2H), 1.31-1.13 (m, 10H); ^{13}C NMR (100 MHz, $CDCl_3$): δ 87.6, 85.3, 78.6, 66.6, 31.2, 30.8, 30.0, 29.7, 26.1, 25.5, 24.9; HRMS (ESI): calcd. for ($[C_{21}H_{34}N_2O_2+Na]^+$): 369.2512, found: 369.2517.

***c,c,c,c*-[5.5.5.5]-*N,N'*-Di(4-methoxybenzyl)-2,8-dioxa-3,9-diazafenestrane (2f)**

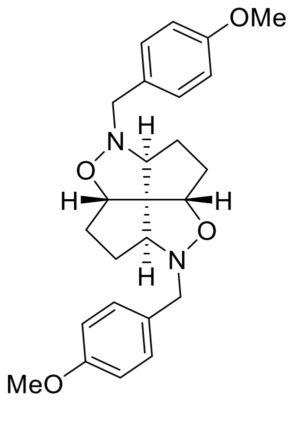

Reaction conditions: method B

Allene bisaldehyde: nona-4,5-dienedial (**1a**) (38.0 mg, 0.250 mmol, 1.00 equiv.)

Hydroxylamine: *N*-(4-methoxybenzyl)hydroxylamine (**S11a**) (95.7 mg, 0.625 mmol, 2.50 equiv.)

Purification method: column chromatography on silica gel (hexane/ethyl acetate = 4 : 1)

Yield: 50.4 mg, 0.119 mmol, 48%

Colorless solid

Mp: 120–121 °C, IR (neat): 2931, 2836, 1613, 1513, 1248, 1175, 1034, 818 cm^{-1}; ^{1}H NMR (400 MHz, $CDCl_3$): δ 7.28-7.25 (m, 4H), 6.86-6.82 (m, 4H), 4.28 (d, *J* = 5.5 Hz, 2H), 3.86-3.78 (m, 10H), 3.01 (d, *J* = 5.0 Hz, 2H), 2.14-1.90 (m, 6H), 1.55-1.49 (m, 2H); ^{13}C NMR (100 MHz, $CDCl_3$): δ 159.0, 130.2, 129.5, 113.8, 86.9, 86.8, 79.5, 60.7, 55.4, 29.8, 28.0; HRMS (ESI): calcd. for ($[C_{25}H_{30}N_2O_4+Na]^+$): 445.2098, found: 445.2098.

***c,c,c,c*-[5.5.5.5]-*N,N'*-Di(4-bromobenzyl)-2,8-dioxa-3,9-diazafenestrane (2g)**

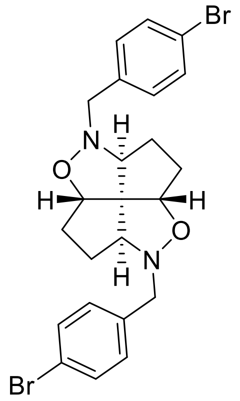

Reaction conditions: method B
Allene bisaldehyde: nona-4,5-dienedial (**1a**) (38.0 mg, 0.250 mmol, 1.00 equiv.)
Hydroxylamine: *N*-(4-bromobenzyl)hydroxylamine (**S11b**) (126 mg, 0.625 mmol, 2.50 equiv.)
Purification method: column chromatography on silica gel (hexane/ethyl acetate = 8 : 1 to 5 : 1)
Yield: 89.3 mg, 0.172 mmol, 69%
Colorless solid
Mp: 164–165 °C, IR (neat): 2932, 2884, 2859, 1487, 1425, 1340, 1068, 1011, 805, 785 cm^{-1}; ^{1}H NMR (400 MHz, $CDCl_3$): δ 7.43 (d, *J* = 8.2 Hz, 4H), 7.23 (d, *J* = 8.2 Hz, 4H), 4.28 (d, *J* = 5.5 Hz, 2H), 3.85 (d, *J* = 14.2 Hz, 2H), 3.76 (d, *J* = 14.2 Hz, 2H), 3.03 (d, *J* = 5.0 Hz, 2H), 2.17-1.93 (m, 6H), 1.65-1.58 (m, 2H); ^{13}C NMR (100 MHz, $CDCl_3$): δ 136.6, 131.5, 130.4, 121.2, 86.9, 86.8, 79.4, 60.2, 29.7, 27.9; HRMS (ESI): calcd. for ($[C_{23}H_{24}Br_2N_2O_2+Na]^+$): 543.0078, found: 543.0076.

***c,c,c,c*-[5.5.5.5]-*N,N'*-Di(2-((*t*-butyldiphenylsilyl)oxy)ethyl)-2,8-dioxa-3,9-diazafenestrane (2h)**

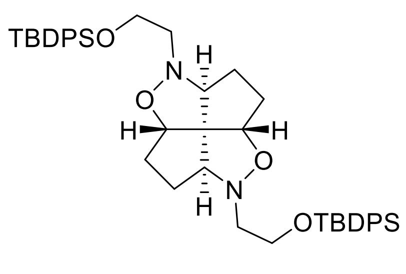

Reaction conditions: method B

Allene bisaldehyde: nona-4,5-dienedial (**1a**) (38.0 mg, 0.250 mmol, 1.00 equiv.)
Hydroxylamine: *N*-(2-((*t*-butyldiphenylsilyl)oxy)ethyl)hydroxylamine (**S11c**) (197 mg, 0.625 mmol, 2.50 equiv.)
Purification method: column chromatography on silica gel (hexane/ethyl acetate = 15 : 1)
Yield: 133 mg, 0.178 mmol, 71%
Colorless solid
Mp: 96–98 °C, IR (neat): 2956, 2930, 2886, 2856, 1540, 1507, 1428, 1112, 740, 701 cm^{-1}; ^{1}H NMR (400 MHz, $CDCl_3$): δ 7.68 (dt, *J* = 1.4, 7.8 Hz, 8H), 7.43-7.33 (m, 12H), 4.16 (d, *J* = 5.0 Hz, 2H), 3.88-3.84 (m, 4H), 2.97-2.90 (m, 4H), 2.84-2.78 (m, 2H), 2.08-1.85 (m, 6H), 1.72-1.67 (m, 2H), 1.04 (s, 18H); ^{13}C NMR (100 MHz, $CDCl_3$): δ 135.8, 135.7, 134.1, 134.0, 129.7, 127.73, 127.69, 86.9, 86.7, 79.6, 62.0, 59.2, 29.7, 27.7, 27.0, 19.4; HRMS (ESI): calcd. for ($[C_{45}H_{58}N_2O_4Si_2+Na]^+$): 769.3827, found: 769.3826.

***c*,*c*,*c*,*c*-[5.5.5.5]-*N*,*N'*-Di(2-((tetrahydro-2*H*-pyran-2-yl)oxy)ethyl)-2,8-dioxa-3,9-diazafenestrane (2i)**

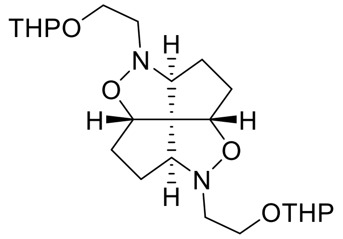

Reaction conditions: method B
Allene bisaldehyde: nona-4,5-dienedial (**1a**) (38.0 mg, 0.250 mmol, 1.00 equiv.)
Hydroxylamine: *N*-(2-((tetrahydro-2*H*-pyran-2-yl)oxy)ethyl)hydroxylamine (**S11d**) (101 mg, 0.625 mmol, 2.50 equiv.)
Purification method: column chromatography on silica gel (hexane/ethyl acetate = 2 : 1)
Yield: 75.0 mg, 0.171 mmol, 68%, diastereomer mixture
Colorless solid
Mp: 38–40 °C, IR (neat): 2939, 2879, 1138, 1122, 1076, 1065, 1035, 970 cm^{-1}; ^{1}H NMR (400 MHz, $CDCl_3$, ^{1}H NMR spectrum was observed as a single isomer): δ 4.64-4.60 (m, 2H), 4.26-4.25 (m, 2H), 3.92-3.84 (m, 4H), 3.69-3.62 (m, 2H), 3.51-3.48 (m, 2H), 3.03-2.83 (m, 6H), 2.15-1.50 (m, 20H); ^{13}C NMR (100 MHz, $CDCl_3$, diastereomer mixture): δ 99.4, 99.0, 86.84, 86.79, 79.9, 65.33, 65.31, 62.5, 57.2, 56.7, 30.8, 29.7, 27.7, 25.6, 19.7; HRMS (ESI): calcd. for ($[C_{23}H_{38}N_2O_6+Na]^+$): 461.2622, found: 461.2620.

c,c,c,c-[5.5.5.5]-N,N'-Di(furan-3-ylmethyl)-2,8-dioxa-3,9-diazafenestrane (2j)

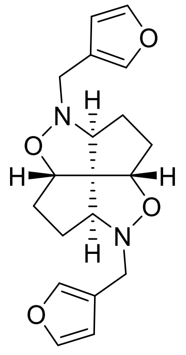

Reaction conditions: method B

Allene bisaldehyde: nona-4,5-dienedial (**1a**) (38.0 mg, 0.250 mmol, 1.00 equiv.)

Hydroxylamine: *N*-(furan-3-ylmethyl)hydroxylamine (**S11e**) (70.7 mg, 0.625 mmol, 2.50 equiv.)

Purification method: column chromatography on silica gel (hexane/ethyl acetate = 2 : 1)

Yield: 59.5 mg, 0.174 mmol, 70%

Colorless solid

Mp: 130–131 °C, IR (neat): 3130, 3115, 2932, 2860, 1501, 1145, 1431, 1340, 1158, 1050, 1020, 874, 787, 601 cm^{-1}; ^{1}H NMR (400 MHz, $CDCl_3$): δ 7.39-7.36 (m, 4H), 6.42 (d, J = 0.9 Hz, 2H), 4.31 (d, J = 5.5 Hz, 2H), 3.79-3.70 (m, 4H), 2.99 (d, J = 5.5 Hz, 2H), 2.18-1.94 (m, 6H), 1.68-1.61 (m, 2H); ^{13}C NMR (100 MHz, $CDCl_3$): δ 143.0, 140.8, 120.9, 111.5, 87.0, 86.8, 79.3, 51.6, 29.8, 28.0; HRMS (ESI): calcd. for ($[C_{19}H_{22}N_2O_4+Na]^+$): 365.1472, found: 365.1476.

c,c,c,c-[5.5.5.5]-N,N'-Di((5S)-5-((t-butoxycarbonyl)amino)-5-(methoxycarbonyl)pentyl)-2,8-dioxa-3,9-diazafenestrane (2k)

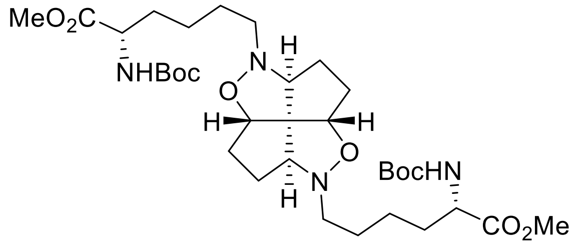

Reaction conditions: method B

Allene bisaldehyde: nona-4,5-dienedial (**1a**) (38.0 mg, 0.250 mmol, 1.00 equiv.)

Hydroxylamine: methyl N^2-(*t*-butoxycarbonyl)-N^6-hydroxy-L-lysinate (**S11f**) (173 mg, 0.625 mmol, 2.50 equiv.)

Purification method: column chromatography on silica gel (hexane/ethyl acetate = 1 : 1)

Yield: 122 mg, 0.182 mmol, 73%

Colorless oil

IR (neat): 3365, 2951, 2933, 2864, 1745, 1715, 1509, 1365, 1166 cm^{-1}; $[\alpha]^{25}_{D}$ = -13.7 (c 0.435, MeOH); ^{1}H NMR (400 MHz, $CDCl_3$): δ 5.01 (br, 2H), 4.29-4.23 (m, 4H), 3.73 (s, 6H), 2.84 (d, J = 5.0 Hz, 2H), 2.72-2.56 (m, 4H), 2.10-1.93 (m, 5H), 1.82-1.37 (m, 33H); ^{13}C NMR (100 MHz, $CDCl_3$): δ 173.4, 155.4, 86.6, 79.8, 57.0, 53.5, 52.2, 32.5, 32.4, 29.6, 28.4, 27.7, 27.6, 23.3; HRMS (ESI): calcd. for ($[C_{33}H_{56}N_4O_{10}+Na]^+$): 691.3889, found: 691.3891.

***c,c,c,c*-[5.5.5.5]-*N,N'*-Di((5*S*)-5-(*t*-butoxycarbonyl)-5-((9-fluorenylmethyloxycarbonyl)amino)pentyl)-2,8-dioxa-3,9-diazafenestrane (2l)**

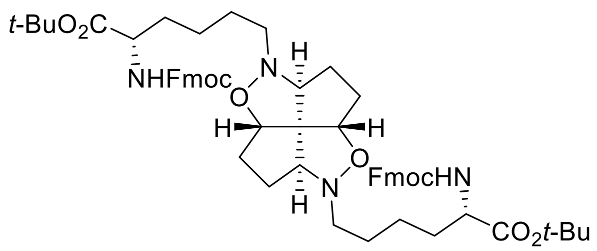

Reaction conditions: method B

Allene bisaldehyde: nona-4,5-dienedial (**1a**) (38.0 mg, 0.250 mmol, 1.00 equiv.)

Hydroxylamine: *t*-butyl N^2-(((9*H*-fluoren-9-yl)methoxy)carbonyl)-N^6-hydroxy-L-lysinate (**S11g**) (275 mg, 0.625 mmol, 2.50 equiv.)

Purification method: column chromatography on silica gel (hexane/ethyl acetate = 2 : 1)

Yield: 155 mg, 0.155 mmol, 62%

Colorless solid

Mp: 72–74 °C, IR (neat): 3336, 2932, 2864, 1718, 1522, 1508, 1449, 1247, 1227, 759, 740 cm^{-1}; $[\alpha]^{26}_{D}$ = -11.0 (c 0.405, MeOH); ^{1}H NMR (400 MHz, $CDCl_3$): δ 7.75 (d, J = 7.8 Hz, 4H), 7.61-7.58 (m, 4H), 7.38 (t, J = 7.8 Hz, 4H), 7.30 (t, J = 7.3 Hz, 4H), 5.40 (t, J = 7.4 Hz, 2H), 4.37 (d, J = 7.3 Hz, 4H), 4.28-4.19 (m, 6H), 2.83 (d, J = 5.0 Hz, 2H), 2.72-2.56 (m, 4H), 2.10-1.80 (m, 8H), 1.70-1.59 (m, 8H), 1.46-1.30 (m, 22H); ^{13}C NMR (100 MHz, $CDCl_3$): δ 171.8, 155.9, 144.0, 143.9, 141.3, 127.7, 127.1, 125.2, 120.0, 86.6, 82.0, 79.9, 79.8, 66.9, 57.1, 57.0, 54.4, 47.3, 32.6, 29.7, 28.1, 27.8, 23.2, 23.0; HRMS (ESI): calcd. for ($[C_{59}H_{72}N_4O_{10}+Na]^+$): 1019.5141, found: 1019.5145.

***c,c,c,c*-[5.5.5.5]-*N,N'*-Diphenyl-2,8-dioxa-3,9-diazafenestrane (2m)**

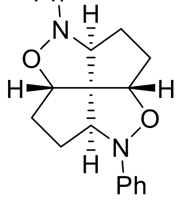

Reaction conditions: method B

Allene bisaldehyde: nona-4,5-dienedial (**1a**) (38.0 mg, 0.250 mmol, 1.00 equiv.)
Hydroxylamine: *N*-phenylhydroxylamine (68.2 mg, 0.625 mmol, 2.50 equiv.)
Purification method: column chromatography on silica gel (hexane/ethyl acetate = 20 : 1)
Yield: 29.4 mg, 0.0879 mmol, 35%
Colorless solid
Mp: 139–140 °C, IR (neat): 2960, 2933, 2887, 1597, 1491, 1350, 1271, 1259, 1039, 954, 755, 692 cm^{-1}; ^{1}H NMR (400 MHz, $CDCl_3$): δ 7.33-7.28 (m, 4H), 7.12-7.09 (m, 4H), 7.07-7.03 (m, 2H), 4.60 (d, *J* = 4.6 Hz, 2H), 3.73 (d, *J* = 6.0 Hz, 2H), 2.39-2.04 (m, 8H); ^{13}C NMR (100 MHz, $CDCl_3$): δ 149.2, 129.0, 123.5, 117.8, 87.4, 86.7, 77.6, 29.8, 29.6; HRMS (ESI): calcd. for ($[C_{21}H_{22}N_2O_2+Na]^+$): 357.1573, found: 357.1574.

***c,c,c,c*-[5.5.5.5]- 2,8-dioxa-3,9-diazafenestranes 2n and 2n'**

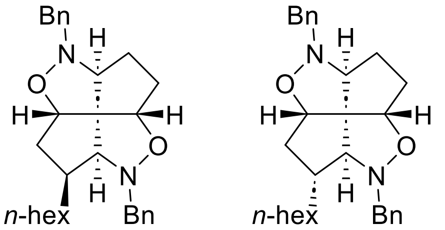

2n **2n'**

Reaction conditions: method A
Allene bisaldehyde: 2-hexylnona-4,5-dienedial (**1b**) (59.1 mg, 0.250 mmol, 1.00 equiv.)
Hydroxylamine hydrochloride: *N*-benzylhydroxylamine hydrochloride (99.8 mg, 0.625 mmol, 2.50 equiv.)
Purification method: column chromatography on silica gel (hexane/ethyl acetate = 20 : 1 to 15 : 1)

Compound **2n**
Yield: 16.4 mg, 0.0367 mmol, 15%
Colorless solid
Mp: 57–58 °C, IR (neat): 2954, 2926, 2855, 1496, 1455, 1347, 1030, 731, 697 cm^{-1}; ^{1}H NMR (400 MHz, $CDCl_3$): δ 7.38-7.36 (m, 4H), 7.31 (m, 4H), 7.27-7.22 (m, 2H), 4.23 (d, *J* = 4.6 Hz, 2H), 4.09 (d, *J* = 14.6 Hz, 1H), 3.90 (s, 2H), 3.65 (d, *J* = 14.6 Hz, 1H), 3.06 (d, *J* = 5.5 Hz, 1H), 3.00 (d, *J* = 5.5 Hz, 1H), 2.52-2.42 (m, 1H), 2.13-1.83 (m, 5H), 1.64-1.28 (m, 11H), 0.88 (t, *J* = 6.9 Hz, 3H); ^{13}C NMR (100 MHz, $CDCl_3$): δ 138.4, 137.5, 128.9, 128.6, 128.42, 128.37, 127.4, 127.2, 86.8, 86.6, 85.3, 80.0, 79.9, 62.2, 61.2, 41.5, 35.2, 31.9, 29.9, 29.8, 29.6, 29.1, 28.0, 22.7, 14.2; HRMS (ESI): calcd. for ($[C_{29}H_{38}N_2O_2+H]^+$): 447.3006, found: 447.3004.

Compound **2n'**

Yield: 45.4 mg, 0.102 mmol, 41%

Colorless oil

IR (neat): 2953, 2926, 2855, 1496, 1455, 1352, 1030, 731, 698 cm^{-1}; ^{1}H NMR (400 MHz, $CDCl_3$): δ 7.37-7.34 (m, 4H), 7.33-7.28 (m, 4H), 7.27-7.22 (m, 2H), 4.31-4.29 (m, 2H), 3.98-3.84 (m, 4H), 3.18 (d, J = 5.0 Hz, 1H), 2.86 (s, 1H), 2.31-2.24 (m, 1H), 2.10-1.90 (m, 3H), 1.83-1.79 (m, 2H), 1.59-1.17 (m, 11H), 0.90 (t, J = 6.9 Hz, 3H); ^{13}C NMR (100 MHz, $CDCl_3$): δ 137.5, 137.2, 129.1, 128.9, 128.4, 127.4, 87.5, 87.4, 86.3, 85.1, 80.1, 61.2, 43.7, 35.3, 35.1, 31.9, 29.3, 28.4, 28.1, 22.8, 14.3; HRMS (ESI): calcd. for ([$C_{29}H_{38}N_2O_2$+H]$^+$): 447.3006, found: 447.3005.

The stereochemistry of each diastereomer was determined by NMR analysis and DFT calculation.

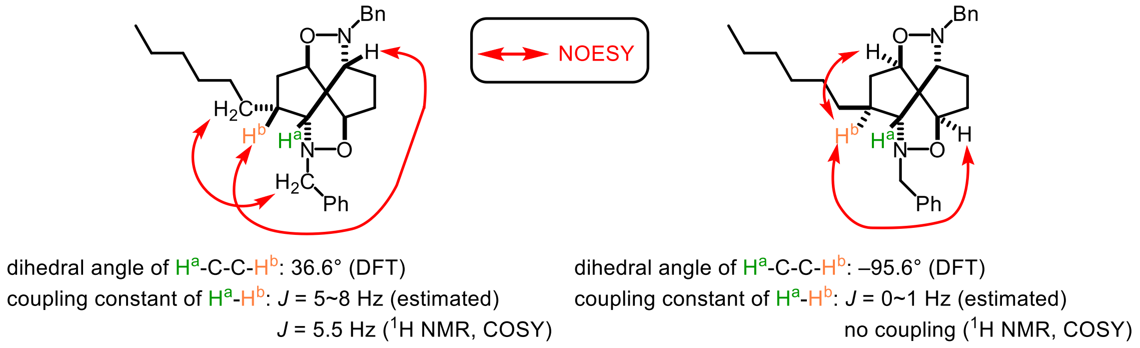

2n **2n'**

Supplementary Fig. 5 Selected NOESY correlations and coupling constants of 2n and 2n'.

***c,c,c,c*-[5.5.5.5]-*N,N'*-Dimethyl-5-hexyl-2,8-dioxa-3,9-diazafenestrane (2o)**

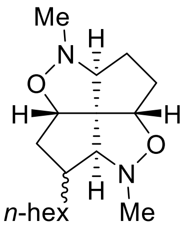

Reaction conditions: method A

Allene bisaldehyde: 2-hexylnona-4,5-dienedial (**1b**) (59.1 mg, 0.250 mmol, 1.00 equiv.)

Hydroxylamine hydrochloride: *N*-methylhydroxylamine hydrochloride (52.2 mg, 0.625 mmol, 2.50 equiv.)

Purification method: column chromatography on silica gel (hexane/ethyl acetate = 4 : 1)

Compound **2o**

Yield: 51.8 mg, 0.0176 mmol, 70%, diastereomer mixture

Colorless oil

IR (neat): 2954, 2927, 2854, 1458, 1432, 1350, 957 cm^{-1}; ^1H NMR (400 MHz, $CDCl_3$, ^1H NMR spectrum was observed as approximately 1 : 1 mixture of two diastereomers): (one isomer) δ 4.30-4.28 (m, 1H), 4.22 (d, *J* = 5.5 Hz, 1H), 2.81 (d, *J* = 5.0 Hz, 1H), 2.72-2.63 (m, 7H), 2.49-2.27 (m, 1H), 2.13-1.68 (m, 6H), 1.56-1.47 (m, 1H), 1.37-1.28 (m, 9H), 0.91-0.87 (m, 3H); (another isomer) δ 4.30-4.28 (m, 1H), 4.25 (d, *J* = 5.0 Hz, 1H), 2.95 (d, *J* = 4.6 Hz, 1H), 2.72-2.63 (m, 7H), 2.49-2.27 (m, 1H), 2.13-1.68 (m, 7H), 1.56-1.47 (m, 1H), 1.37-1.28 (m, 8H), 0.91-0.87 (m, 3H); ^{13}C NMR (100 MHz, $CDCl_3$, diastereomer mixture): δ 88.1, 87.7, 87.6, 87.3, 86.8, 86.6, 85.5, 82.54, 82.47, 81.7, 45.7, 44.0, 43.6, 43.52, 43.47, 41.2, 35.7, 35.3, 35.2, 31.89, 31.86, 29.8, 29.6, 29.5, 29.3, 29.0, 28.4, 27.8, 27.6, 22.7, 14.19, 14.18; HRMS (ESI): calcd. for ($[C_{17}H_{30}N_2O_2+H]^+$): 295.2380, found: 295.2376.

***c*,*c*,*c*,*c*-[5.5.5.5]-5*R*-*N*,*N’*-Dibenzyl-5-benzyloxyl-2,8-dioxa-3,9-diazafenestrane (2p) and *c*,*c*,*c*,*c*-[5.5.5.5]-5*S*-*N*,*N’*-dibenzyl-5-benzyloxyl-2,8-dioxa-3,9-diazafenestrane (2p’)**

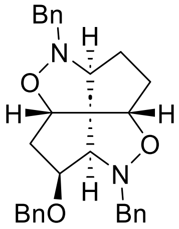

2p

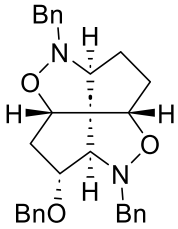

2p'

Reaction conditions: method A

Allene bisaldehyde: 2-(benzyloxy)nona-4,5-dienedial (**1c**) (64.6 mg, 0.250 mmol, 1.00 equiv.)

Hydroxylamine hydrochloride: *N*-benzylhydroxylamine hydrochloride (99.8 mg, 0.625 mmol, 2.50 equiv.)

Purification method: column chromatography on silica gel (hexane/ethyl acetate = 8 : 1 to 5 : 1)

Compound **2p**

Yield: 20.4 mg, 0.0435 mmol, 17%

Colorless solid

Mp: 120–122 °C, IR (neat): 3030, 2930, 2863, 1496, 1454, 1349, 1121, 1054, 1027, 736, 697 cm^{-1}; ^1H NMR (400 MHz, $CDCl_3$): δ 7.38-7.20 (m, 15H), 4.64 (d, *J* = 11.9 Hz, 1H), 4.53 (d, *J* = 11.9 Hz, 1H), 4.48-4.41 (m, 2H), 4.32 (d, *J* = 5.5 Hz, 1H), 4.25 (d, *J* = 6.0 Hz, 1H), 3.86 (dd, *J* = 14.2, 21.0 Hz, 2H), 3.70 (d, *J* = 14.6 Hz, 1H), 3.16 (d, *J* = 5.5 Hz, 1H), 3.01 (d, *J* = 5.0 Hz, 1H), 2.41-2.36 (m, 1H), 2.31-2.23 (m, 1H), 2.10-1.90 (m, 3H), 1.59-1.55 (m, 1H); ^{13}C NMR (100 MHz, $CDCl_3$): δ 138.44, 138.40, 137.3, 129.0, 128.8, 128.5, 128.4, 128.2, 127.7, 127.4, 127.3, 127.1, 87.6, 83.8, 82.5, 80.2, 79.3, 76.7,

72.6, 61.8, 61.0, 33.9, 29.5, 28.1; HRMS (ESI): calcd. for ([$C_{30}H_{32}N_2O_3$+Na]$^+$): 491.2305, found: 491.2301.

Compound **2p'**

Yield: 46.8 mg, 0.0999 mmol, 40%

Yellow oil

IR (neat): 3030, 2929, 2871, 1496, 1454, 1354, 1108, 1051, 1029, 735, 697 cm^{-1}; ^{1}H NMR (400 MHz, $CDCl_3$): δ 7.40-7.21 (m, 15H), 4.48 (d, J = 11.9 Hz, 1H), 4.37 (d, J = 5.0 Hz, 1H), 4.33-4.30 (m, 2H), 4.01-3.86 (m, 4H), 3.68 (d, J = 4.6 Hz, 1H), 3.34 (d, J = 5.5 Hz, 1H), 3.25 (s, 1H), 2.34-2.22 (m, 2H), 2.17-1.89 (m, 3H), 1.57-1.50 (m, 1H); ^{13}C NMR (100 MHz, $CDCl_3$): δ 138.5, 137.7, 137.0, 129.0, 128.9, 128.5, 128.45, 128.40, 127.7, 127.6, 127.3, 87.5, 86.6, 86.2, 84.3, 83.0, 79.7, 70.7, 61.5, 61.3, 36.0, 29.4, 27.9; HRMS (ESI): calcd. for ([$C_{30}H_{32}N_2O_3$+Na]$^+$): 491.2305, found: 491.2308.

The stereochemistry of each diastereomer was determined by NMR analysis and DFT calculation.

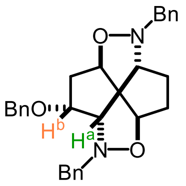

dihedral angle of H^a-C-C-H^b: 35.7° (DFT)
coupling constant of H^a-H^b: J = 5~8 Hz (estimated)
J = 5.5 Hz (^{1}H NMR, COSY)

2p

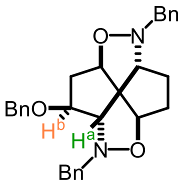

dihedral angle of H^a-C-C-H^b: –94.0° (DFT)
coupling constant of H^a-H^b: J = 0~1 Hz (estimated)
no coupling (^{1}H NMR, COSY)

2p'

Supplementary Fig. 6 Coupling constants of 2p and 2p'.

***c*,*c*,*c*,*c*-[5.5.5.5]-5*R*-*N*,*N'*-Dimethyl-5-benzyloxyl-2,8-dioxa-3,9-diazafenestrane (2q) and *c*,*c*,*c*,*c*-[5.5.5.5]-5*S*-*N*,*N'*-dimethyl-5-benzyloxyl-2,8-dioxa-3,9-diazafenestrane (2q')**

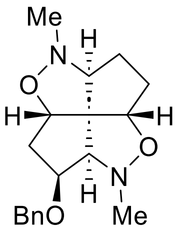

2q

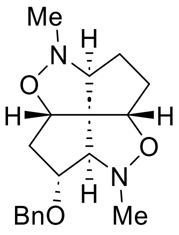

2q'

Reaction conditions: method A

Allene bisaldehyde: 2-(benzyloxy)nona-4,5-dienedial (**1c**) (64.6 mg, 0.250 mmol, 1.00 equiv.)

Hydroxylamine hydrochloride: *N*-methylhydroxylamine hydrochloride (52.2 mg, 0.625 mmol, 2.50 equiv.)

Purification method: column chromatography on silica gel (hexane/ethyl acetate = 1 : 1 to 1 : 2 to 1 : 5)

Compound **2q**

Yield: 18.1 mg, 0.0572 mmol, 23%

Yellow oil

IR (neat): 2959, 2930, 2856, 1456, 1432, 1352, 1119, 1069, 1052, 1025, 738, 698 cm^{-1}; ^{1}H NMR (400 MHz, $CDCl_3$): δ 7.37-7.27 (m, 5H), 4.62 (d, *J* = 11.9 Hz, 1H), 4.50 (d, *J* = 11.9 Hz, 1H), 4.42-4.34 (m, 2H), 4.22 (d, *J* = 6.0 Hz, 1H), 2.91 (d, *J* = 5.5 Hz, 1H), 2.79-2.77 (m, 4H), 2.60 (s, 3H), 2.38-2.33 (m, 1H), 2.24-1.90 (m, 4H), 1.77-1.68 (m, 1H); ^{13}C NMR (100 MHz, $CDCl_3$): δ 138.3, 128.5, 127.8, 127.4, 87.6, 85.2, 82.6, 81.3, 79.8, 79.5, 72.6, 45.4, 43.6, 34.0, 29.5, 27.8; HRMS (ESI): calcd. for ([$C_{18}H_{24}N_2O_3$+Na]$^+$): 339.1679, found: 339.1680.

Compound **2q’**

Yield: 26.2 mg, 0.0828 mmol, 33%

Orange oil

IR (neat): 2954, 2930, 2871, 1456, 1433, 1357, 1106, 1094, 1050, 1028, 738, 698 cm^{-1}; ^{1}H NMR (400 MHz, $CDCl_3$): δ 7.37-7.27 (m, 5H), 4.60 (d, *J* = 12.4 Hz, 1H), 4.48 (d, *J* = 12.4 Hz, 1H), 4.36 (d, *J* = 6.0 Hz, 1H), 4.30 (d, *J* = 5.0 Hz, 1H), 3.86 (d, *J* = 5.0 Hz, 1H), 3.10 (d, *J* = 5.0 Hz, 1H), 2.99 (s, 1H), 2.69 (s, 3H), 2.63 (s, 3H), 2.38-2.26 (m, 2H), 2.15-1.94 (m, 3H), 1.73-1.70 (m, 1H); ^{13}C NMR (100 MHz, $CDCl_3$): δ 138.3, 128.5, 127.8, 127.7, 87.7, 87.2, 86.9, 85.7, 82.4, 81.4, 70.7, 43.83, 43.78, 36.4, 29.5, 27.6; HRMS (ESI): calcd. for ([$C_{18}H_{24}N_2O_3$+Na]$^+$): 339.1679, found: 339.1679.

The stereochemistry of each diastereomer was determined by NMR analysis.

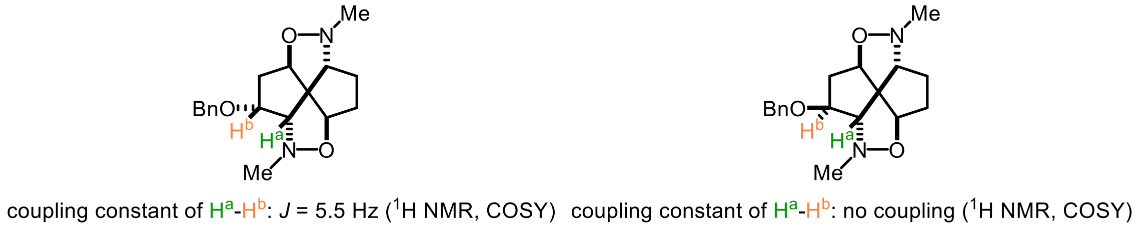

2q **2q’**

Supplementary Fig. 7 Coupling constants of 2q and 2q’.

***c,c,c,c*-[5.5.5.6]-*N,N’*-Dibenzyl-2,8-dioxa-3,9-diazafenestrane (2r)**

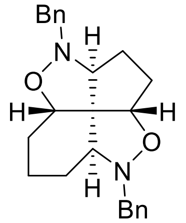

Reaction conditions: method A

Allene bisaldehyde: deca-4,5-dienedial (**1d**) (6.65 mg, 0.0400 mmol, 1.00 equiv.)

Hydroxylamine hydrochloride: *N*-benzylhydroxylamine hydrochloride (16.0 mg, 0.100 mmol, 2.50 equiv.)

Purification method: preparative TLC (hexane/ethyl acetate = 4 : 1)

Yield: 9.70 mg, 0.0258 mmol, 65%

Colorless solid

Mp: 104–106 °C, IR (neat): 2935, 2864, 1496, 1454, 1347, 1330, 1069, 1028, 1003, 734, 697 cm^{-1}; ^{1}H NMR (400 MHz, $CDCl_3$): δ 7.38-7.22 (m, 10H), 4.21 (d, *J* = 4.6 Hz, 1H), 4.03-3.87 (m, 4H), 3.69 (d, *J* = 14.2 Hz, 1H), 3.01 (br, 1H), 2.67 (br, 1H), 2.17-2.07 (m, 1H), 1.95-1.78 (m, 5H), 1.60-1.43 (m, 4H); ^{13}C NMR (100 MHz, $CDCl_3$): δ 137.7, 137.6, 128.9, 128.4, 127.32, 127.26, 88.8, 81.2, 78.3, 70.4, 68.5, 61.3, 59.9, 29.2, 27.8, 26.8, 26.0, 16.9; HRMS (ESI): calcd. for ($[C_{24}H_{28}N_2O_2+Na]^+$): 399.2043, found: 399.2042.

***c,c,c,c*-[5.6.5.6]-*N,N'*-Dibenzyl-2,9-dioxa-3,10-diazafenestrane (2s)**

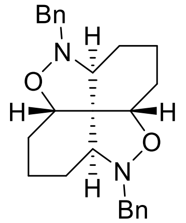

Reaction conditions: method A

Allene bisaldehyde: undeca-5,6-dienedial (**1e**) (45.1 mg, 0.250 mmol, 1.00 equiv.)

Hydroxylamine hydrochloride: *N*-benzylhydroxylamine hydrochloride (99.8 mg, 0.625 mmol, 2.50 equiv.)

Purification method: preparative TLC (hexane/ethyl acetate = 2 : 1)

Yield: 11.3 mg, 0.0289 mmol, 12%

Colorless solid

Mp: 87–89 °C, IR (neat): 2957, 2867, 1496, 1454, 1340, 1308, 1029, 949, 876, 732, 697 cm^{-1}; ^{1}H NMR (400 MHz, $CDCl_3$): δ 7.36-7.23 (m, 10H), 4.51 (d, *J* = 12.3 Hz, 2H), 4.15 (d, *J* = 12.3 Hz, 2H), 3.68-3.64 (m, 2H), 2.91 (dt, *J* = 7.3, 8.7 Hz, 2H), 1.97-1.79 (m, 4H), 1.69-1.46 (m, 8H); ^{13}C NMR

(100 MHz, $CDCl_3$): δ 137.8, 129.2, 128.4, 127.3, 121.3, 71.5, 65.9, 52.9, 34.1, 29.8, 25.1; HRMS (ESI): calcd. for ($[C_{25}H_{30}N_2O_2+Na]^+$): 413.2199, found: 413.2198.

9. General procedure for cycloaddition via nitrile oxide

To a stirred solution of bisoxime **4** (0.250 mmol, 1.00 equiv.) in 1,2-dichloroethane (50.0 mL) were added 12wt% NaOCl aq. (1.55 mL, 2.50 mmol, 10.0 equiv.) and triethylamine (347 μL, 2.50 mmol, 10.0 equiv.) at room temperature under argon. After the resultant mixture was stirred at 80 °C for 13 h, the reaction mixture was diluted with water at room temperature and the aqueous layer was extracted with CH_2Cl_2 three times. The combined organic layer was washed with brine, dried over Na_2SO_4, filtered, and concentrated under reduced pressure. The residue was purified by column chromatography on silica gel to give corresponding fenestrane **5**.

***c,c*-[5.5.5.5]-6-Hexyl-3,9-dioxa-1,7-diiminofenestrane (5b)**

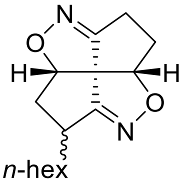

Allene bisoxime: 2-hexylnona-4,5-dienedial bisoxime (**4b**) (66.6 mg, 0.250 mmol, 1.00 equiv.)
Purification method: column chromatography on silica gel (hexane/ethyl acetate = 50 : 1 to 10 : 1)
Yield: 45.3 mg, 0.173 mmol, 69%, diastereomer mixture
Yellow solid
Mp: 48–50 °C, IR (neat): 2928, 2856, 1647, 1636, 1457, 1435, 1233, 890, 849 cm^{-1}; ^1H NMR (400 MHz, $CDCl_3$, ^1H NMR spectrum was observed as approximately 1 : 1 mixture of two diastereomers): (one isomer) δ 5.05-5.00 (m, 2H), 2.92-2.78 (m, 1H), 2.75-2.37 (m, 3H), 2.24-2.11 (m, 2H), 1.92-1.77 (m, 1H), 1.60-1.26 (m, 10H), 0.90-0.85 (m, 3H); (another isomer) δ 5.05-5.00 (m, 2H), 2.92-2.78 (m, 1H), 2.75-2.37 (m, 6H), 1.92-1.77 (m, 1H), 1.60-1.26 (m, 9H), 0.90-0.85 (m, 3H); ^{13}C NMR (100 MHz, $CDCl_3$, diastereomer mixture): δ 166.1, 165.7, 166.4, 163.9, 88.9, 88.3, 85.6, 85.5, 84.9, 84.5, 42.3, 41.4, 36.3, 35.5, 35.2, 35.1, 31.8, 31.7, 31.6, 29.6, 29.3, 28.7, 28.4, 27.9, 22.62, 22.57, 21.54, 21.46, 14.12, 14.08; HRMS (ESI): calcd. for ($[C_{15}H_{22}N_2O_2+Na]^+$): 285.1573, found: 285.1574.

***c,c*-[5.5.5.5]-6*R*-6-Benzyloxyl-3,9-dioxa-1,7-diiminofenestrane (5c)** and ***c,c*-[5.5.5.5]-6*S*-6-benzyloxyl-3,9-dioxa-1,7-diiminofenestrane (5c')**

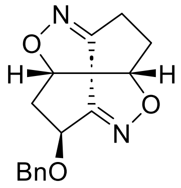

5c

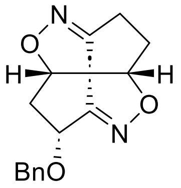

5c'

Allene bisoxime: 2-(benzyloxy)nona-4,5-dienedial bisoxime (**4c**) (72.1 mg, 0.250 mmol, 1.00 equiv.)
Purification method: column chromatography on silica gel (hexane/ethyl acetate = 3 : 1)

Compound **5c**
Yield: 26.5 mg, 0.0932 mmol, 37%
Yellow solid
Mp 124–125 °C, IR (neat): 2936, 2869, 1647, 1636, 1456, 1357, 1126, 888, 847, 742, 701 cm^{-1}; ^{1}H NMR (400 MHz, $CDCl_3$): δ 7.41-7.28 (m, 5H), 5.11 (d, *J* = 5.5 Hz, 1H), 5.00 (d, *J* = 5.5 Hz, 1H), 4.93 (d, *J* = 11.7 Hz, 1H), 4.81 (dd, *J* = 7.3, 10.1 Hz, 1H), 4.64 (d, *J* = 11.7 Hz, 1H), 2.77 (dd, *J* = 7.3, 14.2 Hz, 1H), 2.69-2.63 (m, 1H), 2.58-2.44 (m, 2H), 2.36-2.29 (m, 1H), 2.25-2.16 (m, 1H); ^{13}C NMR (100 MHz, $CDCl_3$): δ 163.8, 163.5, 137.1, 128.6, 128.2, 128.1, 86.5, 85.7, 82.6, 73.2, 72.1, 41.6, 35.4, 21.6; HRMS (ESI): calcd. for ($[C_{16}H_{16}N_2O_3+Na]^+$): 307.1053, found: 307.1055.

Compound **5c'**
Yield: 20.3 mg, 0.0714 mmol, 29%
Colorless solid
Mp: 91–92 °C, IR (neat): 2937, 2859, 1646, 1636, 1455, 1073, 860, 843, 741, 699 cm^{-1}; ^{1}H NMR (400 MHz, $CDCl_3$): δ 7.36-7.27 (m, 5H), 5.10-5.07 (m, 2H), 4.58-4.54 (m, 2H), 4.48 (d, *J* = 12.4 Hz, 1H), 2.74-2.65 (m, 2H), 2.54-2.49 (m, 1H), 2.45-2.38 (m, 1H), 2.35-2.18 (m, 2H); ^{13}C NMR (100 MHz, $CDCl_3$): δ 162.7, 162.6, 137.0, 128.6, 128.2, 128.1, 87.8, 86.0, 83.9, 71.9, 71.2, 43.0, 35.6, 21.4; HRMS (ESI): calcd. for $C_{16}H_{16}N_2O_3$ ($[M+Na]^+$): 307.1053, found: 307.1052.
The stereochemistry of each diastereomer was determined by NMR analysis and DFT calculation.

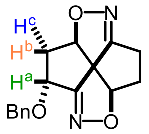

• dihedral angle of H^a-C-C-H^b: –40.4° (DFT)
coupling constant of H^a-H^b: J = 4~8 Hz (estimated)
J = 7.3 Hz (^{1}H NMR, COSY)

• dihedral angle of H^a-C-C-H^c: –160° (DFT)
coupling constant of H^a-H^c: J = 8~12 Hz (estimated)
J = 10 Hz (^{1}H NMR, COSY)

5c

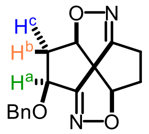

• dihedral angle of H^a-C-C-H^b: –28.2° (DFT)
coupling constant of H^a-H^b: J = 5~8 Hz (estimated)
J = 5.5 Hz (^{1}H NMR, COSY)

• dihedral angle of H^a-C-C-H^c: 91.2° (DFT)
coupling constant of H^a-H^c: J = 0~1 Hz (estimated)
no coupling (^{1}H NMR, COSY)

5c'

Supplementary Fig. 8 Coupling constants of 5c and 5c'.

c,c-[5.5.5.6]-3,9-Dioxa-1,7-diiminofenestrane (5d)

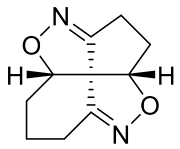

Allene bisoxime: deca-4,5-dienedial bisoxime (**4d**) (29.1 mg, 0.148 mmol, 1.00 equiv.)

Purification method: preparative TLC (hexane/ethyl acetate = 2 : 5)

Yield: 10.2 mg, 0.0531 mmol, 36%

Colorless solid

Mp: 110–111 °C, IR (neat): 2945, 1716, 1645, 1616, 1436, 1263, 1078, 1038, 901, 863, 849 cm^{-1}; ^{1}H NMR (400 MHz, $CDCl_3$): δ 4.85-4.82 (m, 2H), 2.78-2.58 (m, 2H), 2.50-2.19 (m, 4H), 2.13-1.99 (m, 2H), 1.89-1.80 (m, 1H), 1.69-1.61 (m, 1H); ^{13}C NMR (100 MHz, $CDCl_3$): δ 164.4, 153.0, 85.2, 85.0, 79.6, 36.3, 25.8, 22.1, 19.9, 15.8; HRMS (ESI): calcd. for ($[C_{10}H_{12}N_2O_2+Na]^+$): 215.0791, found: 215.0790.

10. Derivatization of oxa-aza fenestranes

(1) Synthesis of *c,c,c,c*-[5.5.5.5]-*N,N'*-di((4'-methoxy-[1,1'-biphenyl]-4-yl)methyl)-2,8-dioxa-3,9-diazafenestrane (7a)

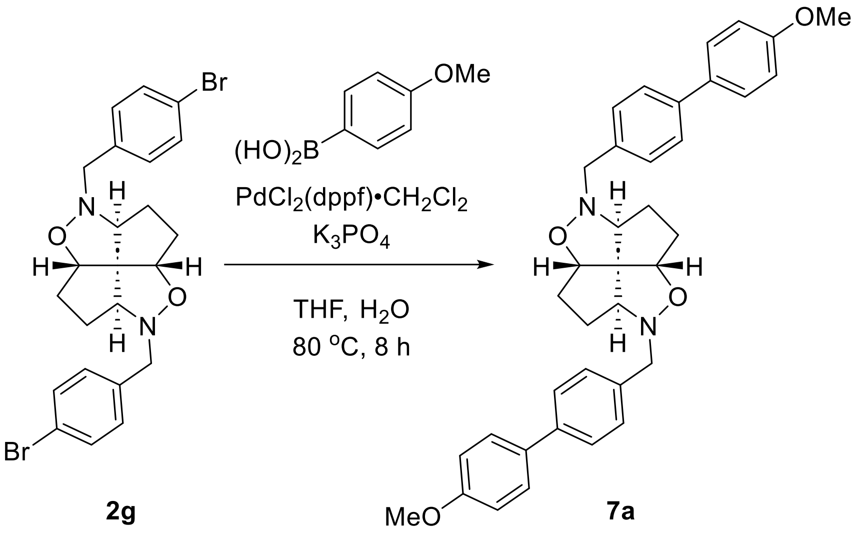

2g **7a**

Fenestrane **2g** (41.6 mg, 0.0800 mmol, 1.00 equiv.), 4-methoxyphenylboronic acid (36.5 mg, 0.240 mmol, 3.00 equiv.), $PdCl_2(dppf){\cdot}CH_2Cl_2$ (6.5 mg, 0.00800 mmol, 10.0 mol%) and tripotassium phosphate (102 mg, 0.480 mmol, 6.00 equiv.) in THF (1.28 mL) and water (0.320 mL) was stirred at 80 °C for 8 h under argon atmosphere. The reaction mixture was cooled to room temperature, diluted with ethyl acetate and water, and the aqueous layer was extracted with ethyl acetate three times. The combined organic layer was washed with brine, dried over Na_2SO_4, filtered, and concentrated under reduced pressure. The residue was purified by column chromatography on silica gel (hexane/ethyl acetate = 4 : 1 to 2 : 1) to give *c,c,c,c*-[5.5.5.5]-*N,N'*-di(4'-methoxy-[1,1'-biphenyl]-4-yl)methyl)-2,8-dioxa-3,9-diazafenestrane (**7a**) (43.6 mg, 0.0759 mmol, 95%) as a colorless solid.

Mp: 203–205 °C, IR (neat): 2959, 2931, 2861, 1607, 1499, 1249, 1179, 1039, 808 cm^{-1}; ^1H NMR (400 MHz, $CDCl_3$): δ 7.53-7.49 (m, 8H), 7.41 (d, J = 8.2 Hz, 4H), 6.98-6.95 (m, 4H), 4.34 (d, J = 5.5 Hz, 2H), 3.95-3.87 (m, 4H), 3.84 (s, 6H), 3.09 (d, J = 5.0 Hz, 2H), 2.19-1.96 (m, 6H), 1.67-1.62 (m, 2H); ^{13}C NMR (100 MHz, $CDCl_3$): δ 159.2, 139.9, 136.0, 133.7, 129.2, 128.2, 126.8, 114.3, 86.9, 86.8, 79.6, 60.8, 55.5, 29.8, 28.0; HRMS (ESI): calcd. for ($[C_{37}H_{38}N_2O_4+Na]^+$): 597.2724, found: 597.2728.

(2) Synthesis of *c,c,c,c*-[5.5.5.5]-*N,N'*-di((4'-(trifluoromethyl)-[1,1'-biphenyl]-4-yl)methyl)-2,8-dioxa-3,9-diazafenestrane (7b)

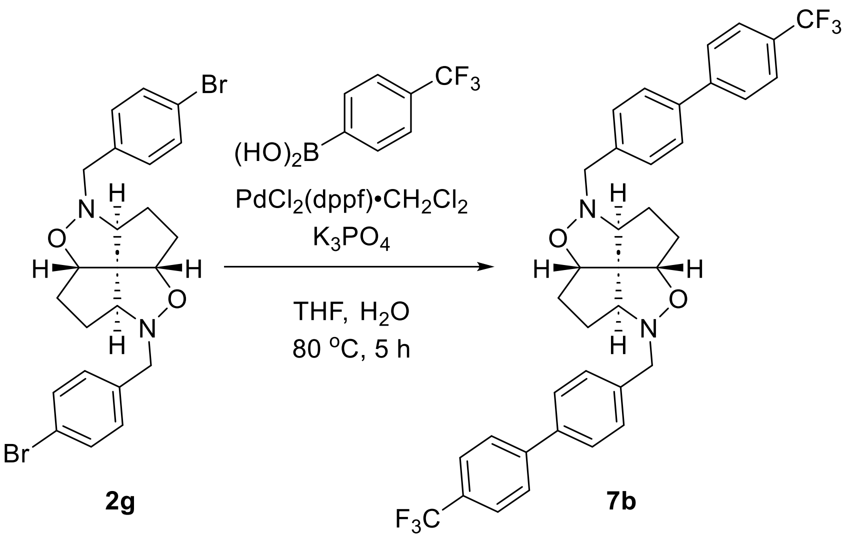

Fenestrane **2g** (52.0 mg, 0.100 mmol, 1.00 equiv.), 4-(trifluoromethyl)phenylboronic acid (57.0 mg, 0.300 mmol, 3.00 equiv.), $PdCl_2(dppf){\cdot}CH_2Cl_2$ (8.2 mg, 0.0100 mmol, 10.0 mol%) and tripotassium phosphate (127 mg, 0.600 mmol, 6.00 equiv.) in THF (1.60 mL) and water (0.400 ml) was stirred at 80 °C for 5 h under argon atmosphere. The reaction mixture was cooled to room temperature, diluted with ethyl acetate and water, and the aqueous layer was extracted with ethyl acetate three times. The combined organic layer was washed with brine, dried over Na_2SO_4, filtered, and concentrated under reduced pressure. The residue was purified by column chromatography on silica gel (hexane/ethyl acetate = 3 : 1 to 1 : 5) to give *c,c,c,c*-[5.5.5.5]-*N,N'*-di((4'-(trifluoromethyl)-[1,1'-biphenyl]-4-yl)methyl)-2,8-dioxa-3,9-diazafenestrane (**7b**) (55.2 mg, 0.0848 mmol, 85%) as a brown solid.

Mp: 230–233 °C, IR (neat): 2935, 2892, 2849, 1541, 1329, 1260, 1166, 1122, 1073, 806 cm^{-1}; ^{1}H NMR (400 MHz, $CDCl_3$): δ 7.68 (s, 8H), 7.56 (d, *J* = 8.2 Hz, 4H), 7.48 (d, *J* = 8.2 Hz, 4H), 4.36 (d, *J* = 5.0 Hz, 2H), 3.98 (d, *J* = 14.2 Hz, 2H), 3.89 (d, *J* = 14.2 Hz, 2H), 3.12 (d, *J* = 5.0 Hz, 2H), 2.21-1.98 (m, 6H), 1.72-1.67 (m, 2H); ^{13}C NMR (100 MHz, $CDCl_3$): δ 144.7, 138.8, 137.9, 129.38, 129.37 (q, *J* = 32.6 Hz), 127.5, 127.4, 125.8 (q, *J* = 3.8 Hz), 124.5 (q, *J* = 271.9 Hz), 87.1, 86.8, 79.6, 60.7, 29.8, 28.0; ^{19}F NMR (376 MHz, $CDCl_3$): δ -63.38; HRMS (ESI): calcd. for ($[C_{37}H_{32}F_6N_2O_2+H]^+$): 651.2441, found: 651.2441.

(3) Synthesis of *c,c,c,c*-[5.5.5.5]-*N,N'*-di((4-(3-hydroxy-3-methylbut-1-yn-1-yl)phenyl)methyl)-2,8-dioxa-3,9-diazafenestrane (7c)

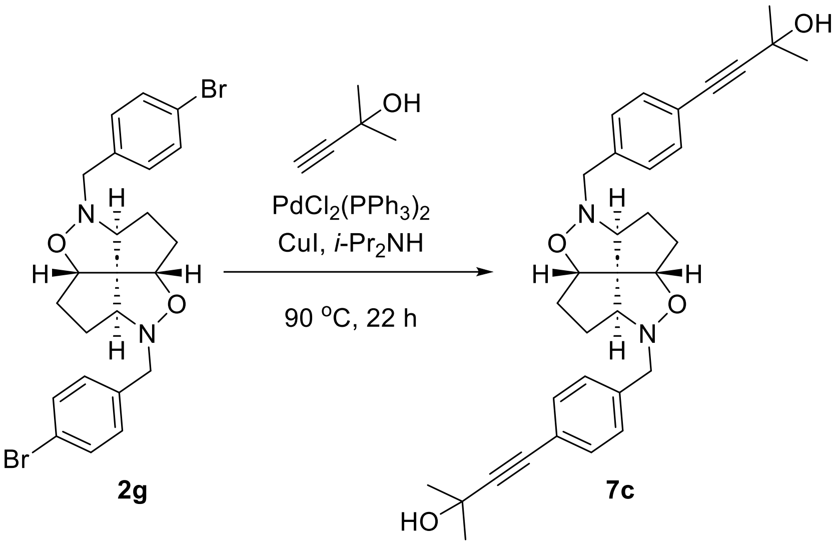

Fenestrane **2g** (28.6 mg, 0.0550 mmol, 1.00 equiv.), $PdCl_2(PPh_3)_2$ (3.9 mg, 0.00550 mmol, 10.0 mol%), copper(I) iodide (1.1 mg, 0.00550 mmol, 10.0 mol%) and 2-methyl-3-butyn-2-ol (16.1 μL, 0.165 mmol, 3.00 equiv.) in anhydrous diisopropylamine (220 μL) was stirred at 90 °C for 22 h under argon atmosphere. The reaction mixture was cooled to room temperature, diluted with ethyl acetate, filtered through a thin silica gel pad, and eluted with ethyl acetate. The filtrate was concentrated under reduced pressure. The residue was purified by column chromatography on silica gel (hexane/ethyl acetate = 2 : 1 to 1 : 1) to give *c,c,c,c*-[5.5.5.5]-*N,N'*-di((4-(3-hydroxy-3-methylbut-1-yn-1-yl)phenyl)methyl)-2,8-dioxa-3,9-diazafenestrane (**7c**) (27.8 mg, 0.0528 mmol, 96%) as a colorless solid.

Mp: 194–195 °C, IR (neat): 3393, 2979, 2932, 2871, 1508, 1362, 1339, 1272, 1165, 962, 814, 738 cm^{-1}; ^1H NMR (400 MHz, $CDCl_3$): δ 7.36 (d, J = 8.2 Hz, 2H), 7.29 (d, J = 8.2 Hz, 4H), 4.30 (d, J = 5.5 Hz, 2H), 3.90-3.82 (m, 4H), 3.03 (d, J = 5.0 Hz, 2H), 2.16-1.92 (m, 8H), 1.61-1.54 (m, 14H); ^{13}C NMR (100 MHz, $CDCl_3$): δ 137.7, 131.7, 128.7, 121.7, 93.7, 86.94, 86.88, 82.3, 79.5, 65.8, 60.8, 31.7, 29.7, 28.0; HRMS (ESI): calcd. for ($[C_{33}H_{38}N_2O_4+Na]^+$): 549.2724, found: 549.2724.

(4) Synthesis of *c,c,c,c*-[5.5.5.5]-*N,N'*-di((*E*)-(4-(3-butoxy-3-oxoprop-1-en-1-yl)phenyl)methyl)-2,8-dioxa-3,9-diazafenestrane (7d)

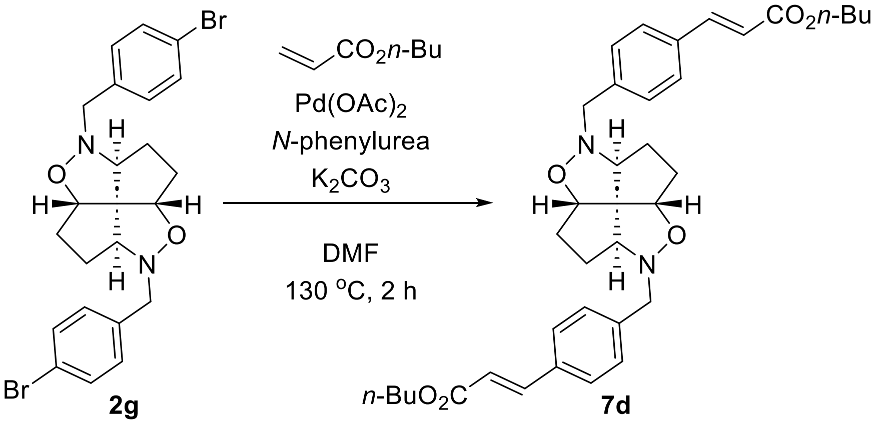

Fenestrane **2g** (52.0 mg, 0.100 mmol, 1.00 equiv.), $Pd(OAc)_2$ (1.1 mg, 0.00500 mmol, 5.00 mol%), *N*-phenylurea (1.4 mg, 0.0100 mmol, 10.0 mol%), potassium carbonate (55.3 mg, 0.400 mmol, 4.00 equiv.) and butyl acrylate (42.7 μL, 0.300 mmol, 3.00 equiv.) in anhydrous DMF (1.00 mL) was stirred at 130 °C for 2 h under argon atmosphere. The reaction mixture was cooled to room temperature, diluted with ethyl acetate and water, and the aqueous layer was extracted with ethyl acetate three times. The combined organic layer was washed with brine, dried over Na_2SO_4, filtered, and concentrated under reduced pressure. The residue was purified by column chromatography on silica gel (hexane/ethyl acetate = 4 : 1) to give *c,c,c,c*-[5.5.5.5]-*N,N'*-di((*E*)-(4-(3-butoxy-3-oxoprop-1-en-1-yl)phenyl)methyl)-2,8-dioxa-3,9-diazafenestrane (**7d**) (35.1 mg, 0.0571 mmol, 57%) as a colorless solid.

Mp 136–137 °C, IR (neat): 2958, 2934, 2887, 2853, 1704, 1636, 1314, 1203, 1184, 1174, 989 cm^{-1}; ^{1}H NMR (400 MHz, $CDCl_3$): δ 7.66 (d, *J* = 16.0 Hz, 2H), 7.48 (d, *J* = 8.2 Hz, 4H), 7.38 (d, *J* = 8.2 Hz, 4H), 6.41 (d, *J* = 16.0 Hz, 2H), 4.31 (d, *J* = 5.5 Hz, 2H), 4.20 (t, *J* = 6.6 Hz, 4H), 3.92 (d, *J* = 14.6 Hz, 2H), 3.83 (d, *J* = 14.6 Hz, 2H), 3.07 (d, *J* = 5.0 Hz, 2H), 2.17-1.94 (m, 6H), 1.72-1.60 (m, 6H), 1.48-1.39 (m, 4H), 0.96 (t, *J* = 7.5Hz, 6H); ^{13}C NMR (100 MHz, $CDCl_3$): δ 167.3, 144.5, 140.1, 133.6, 129.2, 128.2, 118.0, 87.0, 86.8, 79.5, 64.5, 60.6, 30.9, 29.7, 27.9, 19.3, 13.9; HRMS (ESI): calcd. for ($[C_{37}H_{46}N_2O_6+Na]^+$): 637.3248, found: 637.3249.

(5) Synthesis of (1*R*,4*R*,5*S*,6*R*,9*R*)-4,9-bis(benzylamino)spiro[4.4]nonane-1,6-diol (8)

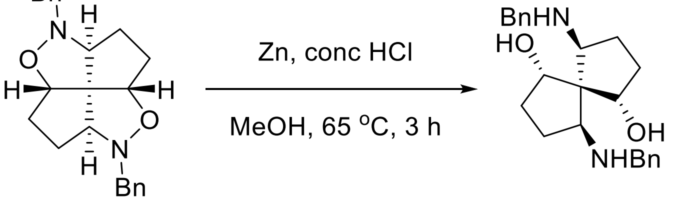

To a stirred solution of *c,c,c,c*-[5.5.5.5]-*N,N'*-dibenzyl-2,8-dioxa-3,9-diazafenestrane (**2a**) (9.1 mg, 0.025 mmol, 1.00 equiv.) in MeOH (2.50 mL) was added portion-wise Zn powder (32.7 mg, 0.500 mmol, 20.0 equiv., activated with 10% HCl aq., preliminarily) and conc. HCl (41.8 μL, 0.500 mmol, 20.0 equiv.) at room temperature under argon. After the resultant mixture was stirred at 65 °C for 3 h, the reaction mixture was cooled to room temperature and concentrated under reduced pressure. The residue was diluted with $CHCl_3$/MeOH (5 : 1) and sat. $NaHCO_3$ aq., and extracted with $CHCl_3$/MeOH (5 : 1) three times. The combined organic layer was dried over Na_2SO_4, filtered, and concentrated under reduced pressure. The crude product was purified by column chromatography on silica gel (CH_2Cl_2/MeOH = 9 : 1) to give (1*R*,4*R*,5*S*,6*R*,9*R*)-4,9-bis(benzylamino)spiro[4.4]nonane-1,6-diol (**8**) (8.5 mg, 0.0232 mmol, 93%) as a colorless oil.

IR (neat): 3273, 3029, 2932, 2870, 1454, 1373, 1349, 1110, 1037, 741, 699 cm^{-1}; ^{1}H NMR (400 MHz, CD_3OD): δ 7.33-7.22 (m, 10H), 3.95 (dd, *J* = 2.9, 5.7 Hz, 2H), 3.86 (d, *J* = 12.8 Hz, 2H), 3.64-3.56 (m, 4H), 2.26-2.07 (m, 4H), 1.69-1.51 (m, 4H); ^{13}C NMR (100 MHz, CD_3OD): δ 140.2, 129.6, 129.5, 128.5, 79.1, 62.3, 58.7, 53.5, 32.3, 29.1; HRMS (ESI): calcd. for ($[C_{23}H_{30}N_2O_2+Na]^+$): 389.2199, found: 389.2199.

(6) Synthesis of *c,c,c*-[5.5.5.5]-3,9-dioxa-2-aza-1-(2-propenyl)-7-iminofenestrane (9a)

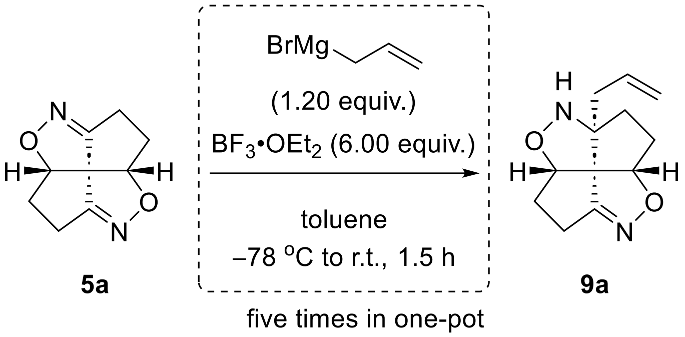

To a stirred solution of diisoxazoline **5a** (8.9 mg, 0.050 mmol, 1.00 equiv.) in anhydrous toluene (1.00 mL) was added dropwise boron trifluoride-ethyl ether complex (37.7 μL, 0.300 mmol, 6.00 equiv.) at –78 °C under argon. After the resultant mixture was stirred at –78 °C for 30 min, to the reaction mixture was added allylmagnesium bromide (0.7 M in Et_2O, 85.7 μL, 0.0600 mmol, 1.20 equiv.) dropwise at –78 °C. After the resultant mixture was stirred at room temperature for 1.5 h, the mixture was cooled to –78 °C, and the second portions of boron trifluoride-ethyl ether complex (37.7 μL, 0.300 mmol, 6.00 equiv.) and allylmagnesium bromide (0.7 M in Et_2O, 85.7 μL, 0.0600 mmol, 1.20 equiv.) were added. The reaction mixture was again allowed to warm to room temperature and stirred for 1.5 h. The mixture was cooled to –78 °C, and the third portions of boron trifluoride-ethyl ether complex (37.7 μL, 0.300 mmol, 6.00 equiv.) and allylmagnesium bromide (0.7 M in Et_2O, 85.7 μL, 0.0600 mmol, 1.20 equiv.) were added. The reaction mixture was again allowed to warm to room

temperature and stirred for 1.5 h. The mixture was cooled to –78 °C, and the fourth portions of boron trifluoride-ethyl ether complex (37.7 μL, 0.300 mmol, 6.00 equiv.) and allylmagnesium bromide (0.7 M in Et_2O, 85.7 μL, 0.0600 mmol, 1.20 equiv.) were added. The reaction mixture was again allowed to warm to room temperature and stirred for 1.5 h. The mixture was cooled to –78 °C, and the fifth portions of boron trifluoride-ethyl ether complex (37.7 μL, 0.300 mmol, 6.00 equiv.) and allylmagnesium bromide (0.7 M in Et_2O, 85.7 μL, 0.0600 mmol, 1.20 equiv.) were added. The reaction mixture was again allowed to warm to room temperature and stirred for 1.5 h, and quenched with water. The aqueous layer was extracted with $CHCl_3$/MeOH (4 : 1) three times. The combined organic layer was dried over Na_2SO_4, filtered, and concentrated under reduced pressure. The residue was purified by preparative TLC (CH_2Cl_2/MeOH = 95 : 5) to give *c,c,c*-[5.5.5.5]-3,9-dioxa-2-aza-1-(2-propenyl)-7-iminofenestrane (**9a**) (6.4 mg, 0.0291 mmol, 58%) as a colorless solid.
Mp: 145–147 °C, IR (neat): 3220, 3074, 2936, 2856, 1717, 1637, 1439, 1066, 1018, 920, 901, 876, 811 cm^{-1}; ^1H NMR (400 MHz, $CDCl_3$): δ 5.95-5.85 (m, 1H), 5.29 (br, 1H), 5.18-5.11 (m, 2H), 4.91 (d, J = 3.2 Hz, 1H), 4.40 (d, J = 6.4 Hz, 1H), 2.69-2.55 (m, 3H), 2.42-2.28 (m, 2H), 2.24-2.04 (m, 4H), 1.83-1.75 (m, 1H); ^{13}C NMR (100 MHz, $CDCl_3$): δ 170.2, 133.9, 118.5, 92.6, 88.9, 87.9, 82.3, 38.9, 32.0, 30.7, 29.2, 23.6; HRMS (ESI): calcd. for ($[C_{12}H_{16}N_2O_2+Na]^+$): 243.1104, found: 243.1107.

The product was obtained as a single diastereomer. We speculated that the obtained isomer is *c,c,c*-**9a** because this is the most stable isomer according to DFT calculations (We additionally calculate the diastereomers **9a"** and **9a'"** to examine the influence of the stereochemistry on the energy levels of the isomers, although these diastereomers should not be generated in the examined reaction). Although we attempted to determine the structure by NOESY, no significant correlation was observed.

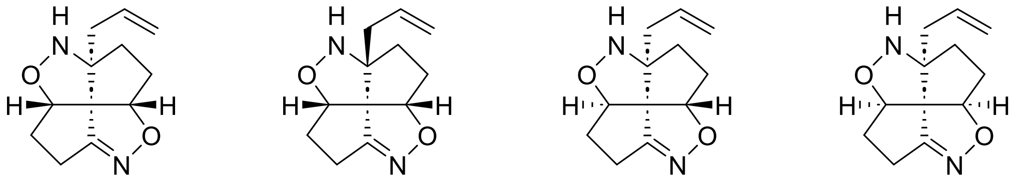

9a (***c,c,c*-9a**)
0 kcal/mol (DFT)

***t,c,c*-9a'**
+18.8 kcal/mol (DFT)

***c,c,t*-9a"**
+23.0 kcal/mol (DFT)

***c,t,c*-9a'"**
+59.6 kcal/mol (DFT)

Supplementary Fig. 9 Relative energy of each isomer calculated by DFT.

(7) Synthesis of *c*,*c*,*c*,*c*-[5.5.5.5]-3,9-dioxa-2,8-diaza-1,7-di(2-propenyl)fenestrane (9b)

Compound **9b** was prepared according to a modified literature procedure[16].

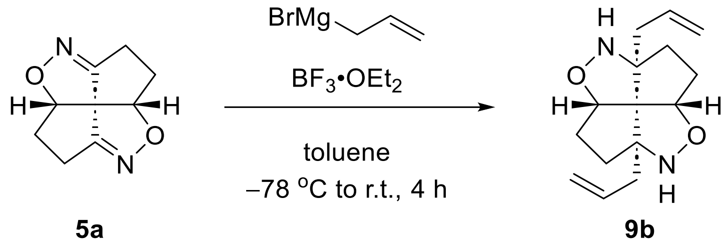

To a stirred solution of diisoxazoline **5a** (8.9 mg, 0.050 mmol, 1.00 equiv.) in anhydrous toluene (1.00 mL) was added dropwise boron trifluoride-ethyl ether complex (71.0 μL, 0.500 mmol, 10.0 equiv.) at –78 °C under argon. After the resultant mixture was stirred at –78 °C for 30 min, to the reaction mixture was added a solution of allylmagnesium bromide (0.7 M in Et_2O, 714 μL, 10.0 mmol, 10.0 equiv.) dropwise at –78 °C. After the resultant mixture was stirred at room temperature for 4 h, the reaction was quenched with water and the aqueous layer was extracted with $CHCl_3$/MeOH (4 : 1) three times. The combined organic layer was dried over Na_2SO_4, filtered, and concentrated under reduced pressure. The residue was purified by preparative TLC (CH_2Cl_2/MeOH = 96 : 4) to give *c*,*c*,*c*,*c*-[5.5.5.5]-3,9-dioxa-2,8-diaza-1,7-di(2-propenyl)fenestrane (**9b**) (6.7 mg, 0.0255 mmol, 51%) as a colorless solid.

Mp: 101–103 °C, IR (neat): 3211, 3073, 2959, 2927, 2871, 1717, 1637, 1456, 1101, 1022, 912 cm^{-1}; ^1H NMR (400 MHz, $CDCl_3$): δ 5.98-5.73 (m, 2H), 5.41-4.91 (m, 6H), 4.58 (dd, J = 4.6, 5.5 Hz, 2H), 2.73-2.65 (m, 2H), 2.57-2.52 (m, 2H), 2.15-1.99 (m, 6H), 1.83-1.76 (m, 2H); ^{13}C NMR (100 MHz, $CDCl_3$): δ 135.2, 118.1, 96.8, 77.8, 70.7, 40.2, 34.2, 28.9; HRMS (ESI): calcd. for ($[C_{15}H_{22}N_2O_2+Na]^+$): 285.1573, found: 285.1578.

The product was obtained as a single diastereomer. We speculated that the obtained isomer is *c,c,c,c*-**9b** because this is the most stable isomer according to DFT calculations. (We additionally calculate the diastereomers **9b'''** and **9b''''** to examine the influence of the stereochemistry on the energy levels of the isomers, although these diastereomers should not be generated in the examined reaction). Although we attempted to determine the structure by NOESY, no significant correlation was observed.

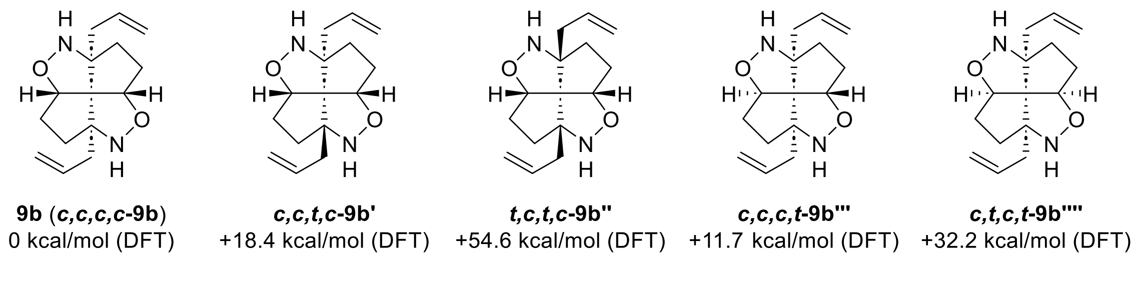

Supplementary Fig. 10 Relative energy of each isomer calculated by DFT.

11. X-ray Crystallographic Data

All the crystallographic data for this paper are available from the Cambridge Crystallographic Data Centre (CCDC) via www.ccdc.cam.ac.uk/data_request/cif.

(1) X-ray Diffraction Analysis of 2a (CCDC2233428)

Diffraction data were collected in θ ranges specified in Supplementary Table 4 at 123 K on a Rigaku R-AXIS Rapid diffractometer with graphite monochromatized Cu-Ka radiation (λ = 1.54187 Å). The structure was solved by direct methods and refined by the full-matrix least-squares on F^2. All non-hydrogen atoms were refined with anisotropic displacement parameters. All hydrogen atoms were refined using the riding model. Final refinement details are compiled in Supplementary Table 4.

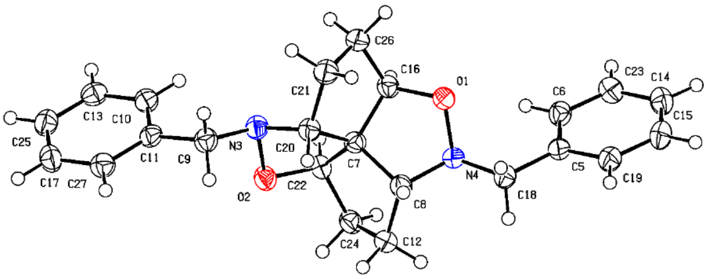

Supplementary Fig. 11 ORTEP diagram of **2a** (CCDC2233428).

Supplementary Table 4 Selected crystallographic data and collection parameters for **2a**.

formula	$C_{23}H_{26}N_2O_2$
formula weight	362.47
crystal system	orthorhombic
space group	Pbca (#61)
a	10.2832(4) Å
b	14.7846(5) Å
c	25.0619(9) Å
alpha	90 deg.
beta	90 deg.
gamma	90 deg.
volume	3810.2(2) Å^3
Z	8
D (calcd)	1.264 g/cm^3
μ	6.389 cm^{-1}
F(000)	1552.00
crystal size	0.400 × 0.300 × 0.300 mm
maximum 2θ	136.4 deg.
reflections collected	39310
independent reflections [*R*(int)]	0.0671
max. and min. transmission	0.826/0.594
goodness-of-fit on F^2	1.009
R_1 [$I > 2\sigma(I)$]	0.0441
R, wR_2 (all data)	0.1062
Weighting scheme	$R_1 = \Sigma \|\|Fo\| - \|Fc\|\| / \Sigma \|Fo\|$<br>$wR_2 = [\Sigma (w (Fo^2 - Fc^2)^2)/ \Sigma w(Fo^2)^2]^{1/2}$
largest diff. peak and hole	0.28 and -0.44 e Å^{-3}

Check CIF file of **2a** (CCDC2233428)

checkCIF/PLATON report

Structure factors have been supplied for datablock(s) HI9-1-90-1

THIS REPORT IS FOR GUIDANCE ONLY. IF USED AS PART OF A REVIEW PROCEDURE FOR PUBLICATION, IT SHOULD NOT REPLACE THE EXPERTISE OF AN EXPERIENCED CRYSTALLOGRAPHIC REFEREE.

No syntax errors found. CIF dictionary Interpreting this report

Datablock: HI9-1-90-1

```
Bond precision:    C-C = 0.0020 A          Wavelength=1.54187

Cell:               a=10.2832(4)      b=14.7846(5)      c=25.0619(9)
                    alpha=90          beta=90           gamma=90
Temperature:        123 K

                  Calculated                    Reported
Volume            3810.2(2)                     3810.2(2)
Space group       P b c a                       P b c a
Hall group        -P 2ac 2ab                    -P 2ac 2ab
Moiety formula    C23 H26 N2 O2                 C23 H26 N2 O2
Sum formula       C23 H26 N2 O2                 C23 H26 N2 O2
Mr                362.46                        362.47
Dx,g cm-3         1.264                         1.264
Z                 8                             8
Mu (mm-1)         0.638                         0.639
F000              1552.0                        1552.0
F000'             1556.36
h,k,lmax          12,17,30                      12,17,30
Nref              3487                          3486
Tmin,Tmax         0.795,0.826                   0.594,0.826
Tmin'             0.774

Correction method= # Reported T Limits: Tmin=0.594 Tmax=0.826
AbsCorr = MULTI-SCAN

Data completeness= 1.000            Theta(max)= 68.190

R(reflections)= 0.0441( 2576)                        wR2(reflections)=
                                                     0.1062( 3486)
S = 1.009                  Npar= 244
```

```
The following ALERTS were generated. Each ALERT has the format
       test-name_ALERT_alert-type_alert-level.
Click on the hyperlinks for more details of the test.
```

Alert level C

```
PLAT906_ALERT_3_C Large K Value in the Analysis of Variance ......      4.226 Check
```

Alert level G

```
CHEMS02_ALERT_1_G  Please check that you have entered the correct
            _publ_requested_category classification of your compound;
            FI or CI or EI for inorganic; FM or CM or EM for metal-organic;
            FO or CO or EO for organic.
            From the CIF: _publ_requested_category   CHOOSE FI FM FO CI CM CO or A
            From the CIF: _chemical_formula_sum :C23 H26 N2 O2
PLAT005_ALERT_5_G No Embedded Refinement Details Found  in the CIF     Please Do !
PLAT395_ALERT_2_G Deviating  X-O-Y   Angle From 120 for O1        .      101.4 Degree
PLAT395_ALERT_2_G Deviating  X-O-Y   Angle From 120 for O2        .      101.3 Degree
PLAT793_ALERT_4_G Model has Chirality at C8          (Centro SPGR)          R Verify
PLAT793_ALERT_4_G Model has Chirality at C16         (Centro SPGR)          R Verify
PLAT793_ALERT_4_G Model has Chirality at C20         (Centro SPGR)          R Verify
PLAT793_ALERT_4_G Model has Chirality at C22         (Centro SPGR)          R Verify
PLAT912_ALERT_4_G Missing # of FCF Reflections Above STh/L=  0.600          2 Note
PLAT978_ALERT_2_G Number C-C Bonds with Positive Residual Density.          0 Info
```

```
   0 ALERT level A = Most likely a serious problem - resolve or explain
   0 ALERT level B = A potentially serious problem, consider carefully
   1 ALERT level C = Check. Ensure it is not caused by an omission or oversight
  10 ALERT level G = General information/check it is not something unexpected

   1 ALERT type 1 CIF construction/syntax error, inconsistent or missing data
   3 ALERT type 2 Indicator that the structure model may be wrong or deficient
   1 ALERT type 3 Indicator that the structure quality may be low
   5 ALERT type 4 Improvement, methodology, query or suggestion
   1 ALERT type 5 Informative message, check
```

It is advisable to attempt to resolve as many as possible of the alerts in all categories. Often the minor alerts point to easily fixed oversights, errors and omissions in your CIF or refinement strategy, so attention to these fine details can be worthwhile. In order to resolve some of the more serious problems it may be necessary to carry out additional measurements or structure refinements. However, the purpose of your study may justify the reported deviations and the more serious of these should normally be commented upon in the discussion or experimental section of a paper or in the "special_details" fields of the CIF. checkCIF was carefully designed to identify outliers and unusual parameters, but every test has its limitations and alerts that are not important in a particular case may appear. Conversely, the absence of alerts does not guarantee there are no aspects of the results needing attention. It is up to the individual to critically assess their own results and, if necessary, seek expert advice.

Publication of your CIF in IUCr journals

A basic structural check has been run on your CIF. These basic checks will be run on all CIFs submitted for publication in IUCr journals (*Acta Crystallographica*, *Journal of Applied Crystallography*, *Journal of Synchrotron Radiation*); however, if you intend to submit to *Acta Crystallographica Section C* or *E* or *IUCrData*, you should make sure that full publication checks are run on the final version of your CIF prior to submission.

Publication of your CIF in other journals

Please refer to the *Notes for Authors* of the relevant journal for any special instructions relating to CIF submission.

PLATON version of 28/11/2022; check.def file version of 28/11/2022

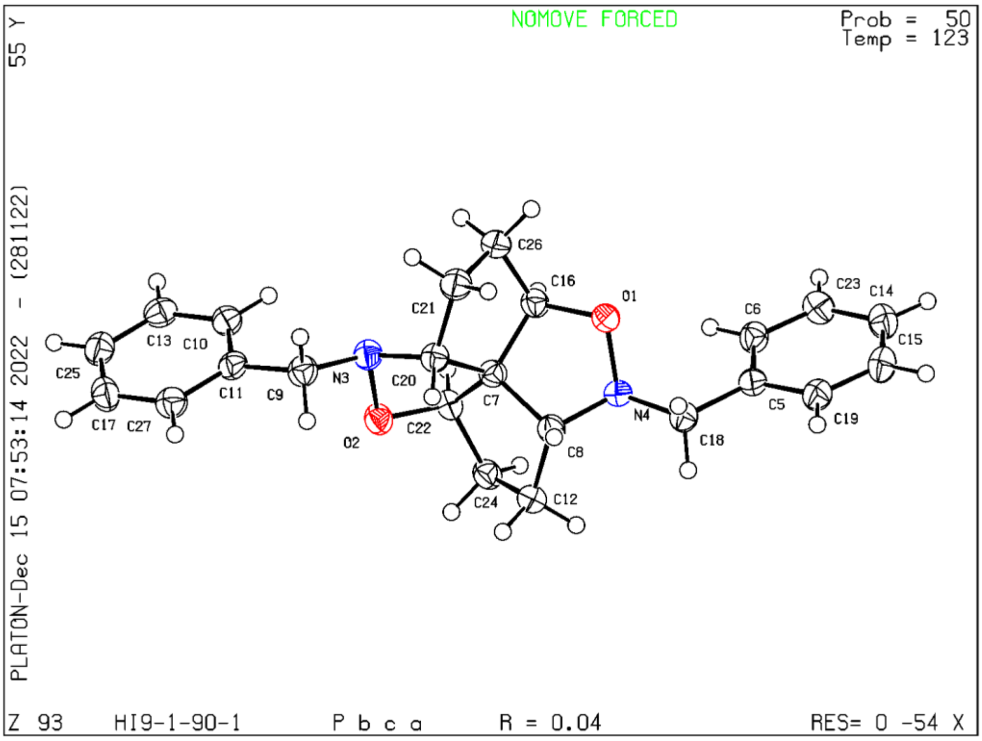

(2) X-ray Diffraction Analysis of 2r (CCDC2233429)

Diffraction data were collected in θ ranges specified in Supplementary Table 5 at 123 K on a Rigaku R-AXIS Rapid diffractometer with graphite monochromatized Cu-Ka radiation (λ = 1.54187 Å). The structure was solved by direct methods and refined by the full-matrix least-squares on F^2. All non-hydrogen atoms were refined with anisotropic displacement parameters. All hydrogen atoms were refined using the riding model. Final refinement details are compiled in Supplementary Table 5.

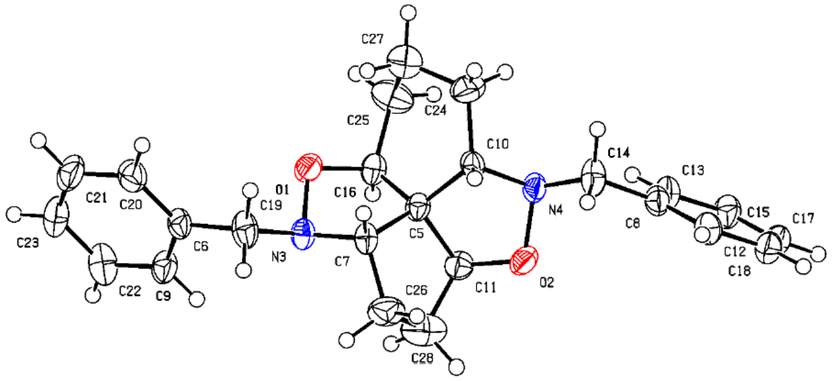

Supplementary Fig. 12 ORTEP diagram of **2r** (CCDC2233429)

Supplementary Table 5 Selected crystallographic data and collection parameters for **2r**.

formula	$C_{24}H_{28}N_2O_2$
formula weight	376.50
crystal system	triclinic
space group	P-1 (#2)
a	5.7654(2) Å
b	11.4978(4) Å
c	15.6915(6) Å
alpha	75.832(5) deg.
beta	79.279(6) deg.
gamma	82.509(6) deg.
volume	987.00(7) Å^3
Z	2
D (calcd)	1.267 g/cm^3
μ	6.351 cm^{-1}
F(000)	404.0
crystal size	0.800 × 0.400 × 0.200 mm
maximum 2θ	136.3 deg.
reflections collected	9933
independent reflections [*R*(int)]	0.0629
max. and min. transmission	0.881/0.560
goodness-of-fit on F^2	1.082
R_1 [$I > 2\sigma(I)$]	0.0776
R, wR_2 (all data)	0.2744
Weighting scheme	$R_1 = \Sigma \|\|Fo\| - \|Fc\|\| / \Sigma \|Fo\|$<br>$wR_2 = [\Sigma (w (Fo^2 - Fc^2)^2)/ \Sigma w(Fo^2)^2]^{1/2}$
largest diff. peak and hole	0.68 and -0.39 e Å^{-3}

Check CIF file of **2r** (CCDC2233429)

checkCIF/PLATON report

Structure factors have been supplied for datablock(s) HI9-4-34

THIS REPORT IS FOR GUIDANCE ONLY. IF USED AS PART OF A REVIEW PROCEDURE FOR PUBLICATION, IT SHOULD NOT REPLACE THE EXPERTISE OF AN EXPERIENCED CRYSTALLOGRAPHIC REFEREE.

No syntax errors found. CIF dictionary Interpreting this report

Datablock: HI9-4-34

Bond precision: C-C = 0.0053 A Wavelength=1.54187

Cell:	a=5.7654(2)	b=11.4978(4)	c=15.6915(6)
	alpha=75.832(5)	beta=79.279(6)	gamma=82.509(6)
Temperature:	123 K		

	Calculated	Reported
Volume	987.00(7)	987.00(7)
Space group	P -1	P -1
Hall group	-P 1	-P 1
Moiety formula	C24 H28 N2 O2	C24 H28 N2 O2
Sum formula	C24 H28 N2 O2	C24 H28 N2 O2
Mr	376.48	376.50
Dx,g cm-3	1.267	1.267
Z	2	2
Mu (mm-1)	0.634	0.635
F000	404.0	404.0
F000'	405.13	
h,k,lmax	6,13,18	6,13,18
Nref	3591	3459
Tmin,Tmax	0.774,0.881	0.560,0.881
Tmin'	0.573	

Correction method= # Reported T Limits: Tmin=0.560 Tmax=0.881
AbsCorr = MULTI-SCAN

Data completeness= 0.963 Theta(max)= 68.130

R(reflections)= 0.0776(2148) wR2(reflections)= 0.2744(3459)

S = 1.082 Npar= 253

```
The following ALERTS were generated. Each ALERT has the format
       test-name_ALERT_alert-type_alert-level.
Click on the hyperlinks for more details of the test.
```

Alert level C

```
DIFMX02_ALERT_1_C  The maximum difference density is > 0.1*ZMAX*0.75
            The relevant atom site should be identified.
PLAT029_ALERT_3_C _diffrn_measured_fraction_theta_full value Low .      0.963 Why?
PLAT084_ALERT_3_C High wR2 Value (i.e. > 0.25) ...................       0.27 Report
PLAT097_ALERT_2_C Large Reported Max.  (Positive) Residual Density       0.68 eA-3
PLAT155_ALERT_4_C The Triclinic Unitcell is NOT Reduced ..........     Please Do !
PLAT241_ALERT_2_C High   'MainMol' Ueq as Compared to Neighbors of        C28 Check
PLAT340_ALERT_3_C Low Bond Precision on  C-C Bonds ...............    0.00528 Ang.
PLAT906_ALERT_3_C Large K Value in the Analysis of Variance ......      5.740 Check
PLAT911_ALERT_3_C Missing FCF Refl Between Thmin & STh/L=    0.600        125 Report
PLAT918_ALERT_3_C Reflection(s) with I(obs) much Smaller I(calc) .          1 Check
```

Alert level G

```
CHEMS02_ALERT_1_G  Please check that you have entered the correct
            _publ_requested_category classification of your compound;
            FI or CI or EI for inorganic; FM or CM or EM for metal-organic;
            FO or CO or EO for organic.
            From the CIF: _publ_requested_category   CHOOSE FI FM FO CI CM CO or A
            From the CIF: _chemical_formula_sum :C24 H28 N2 O2
PLAT005_ALERT_5_G No Embedded Refinement Details Found  in the CIF     Please Do !
PLAT063_ALERT_4_G Crystal Size Possibly too Large for Beam Size ..       0.80 mm
PLAT072_ALERT_2_G SHELXL First  Parameter in WGHT  Unusually Large       0.16 Report
PLAT395_ALERT_2_G Deviating  X-O-Y   Angle From 120 for O1        .     102.9 Degree
PLAT395_ALERT_2_G Deviating  X-O-Y   Angle From 120 for O2        .     102.1 Degree
PLAT793_ALERT_4_G Model has Chirality at C7           (Centro SPGR)         S Verify
PLAT793_ALERT_4_G Model has Chirality at C10          (Centro SPGR)         S Verify
PLAT793_ALERT_4_G Model has Chirality at C11          (Centro SPGR)         S Verify
PLAT793_ALERT_4_G Model has Chirality at C16          (Centro SPGR)         S Verify
PLAT909_ALERT_3_G Percentage of I>2sig(I) Data at Theta(Max) Still        41% Note
PLAT912_ALERT_4_G Missing # of FCF Reflections Above STh/L=  0.600          9 Note
PLAT978_ALERT_2_G Number C-C Bonds with Positive Residual Density.          1 Info
PLAT992_ALERT_5_G Repd & Actual _reflns_number_gt Values Differ by          2 Check
```

```
   0 ALERT level A = Most likely a serious problem - resolve or explain
   0 ALERT level B = A potentially serious problem, consider carefully
  10 ALERT level C = Check. Ensure it is not caused by an omission or oversight
  14 ALERT level G = General information/check it is not something unexpected

   2 ALERT type 1 CIF construction/syntax error, inconsistent or missing data
   6 ALERT type 2 Indicator that the structure model may be wrong or deficient
   7 ALERT type 3 Indicator that the structure quality may be low
   7 ALERT type 4 Improvement, methodology, query or suggestion
   2 ALERT type 5 Informative message, check
```

It is advisable to attempt to resolve as many as possible of the alerts in all categories. Often the minor alerts point to easily fixed oversights, errors and omissions in your CIF or refinement strategy, so attention to these fine details can be worthwhile. In order to resolve some of the more serious problems it may be necessary to carry out additional measurements or structure refinements. However, the purpose of your study may justify the reported deviations and the more serious of these should normally be commented upon in the discussion or experimental section of a paper or in the "special_details" fields of the CIF. checkCIF was carefully designed to identify outliers and unusual parameters, but every test has its limitations and alerts that are not important in a particular case may appear. Conversely, the absence of alerts does not guarantee there are no aspects of the results needing attention. It is up to the individual to critically assess their own results and, if necessary, seek expert advice.

Publication of your CIF in IUCr journals

A basic structural check has been run on your CIF. These basic checks will be run on all CIFs submitted for publication in IUCr journals (*Acta Crystallographica*, *Journal of Applied Crystallography*, *Journal of Synchrotron Radiation*); however, if you intend to submit to *Acta Crystallographica Section C* or *E* or *IUCrData*, you should make sure that full publication checks are run on the final version of your CIF prior to submission.

Publication of your CIF in other journals

Please refer to the *Notes for Authors* of the relevant journal for any special instructions relating to CIF submission.

PLATON version of 28/11/2022; check.def file version of 28/11/2022

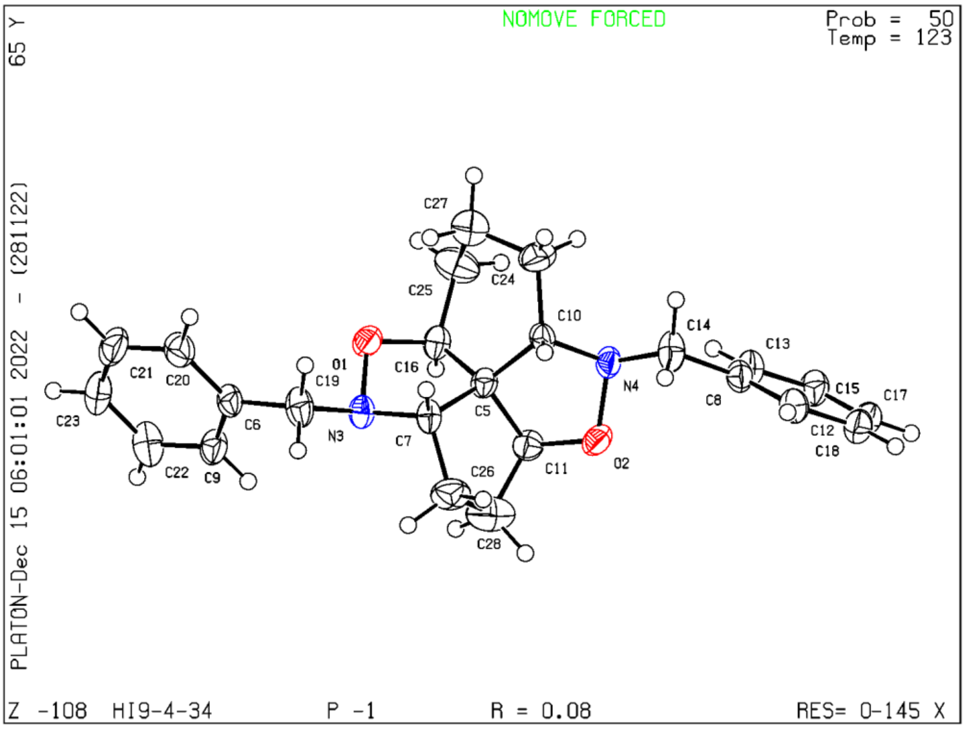

(3) X-ray Diffraction Analysis of 5a (CCDC2233425)

Diffraction data were collected in θ ranges specified in Supplementary Table 6 at 123 K on a Rigaku R-AXIS Rapid diffractometer with graphite monochromatized Cu-Ka radiation (λ = 1.54187 Å). The structure was solved by direct methods and refined by the full-matrix least-squares on F^2. All non-hydrogen atoms were refined with anisotropic displacement parameters. All hydrogen atoms were refined using the riding model. Final refinement details are compiled in Supplementary Table 6.

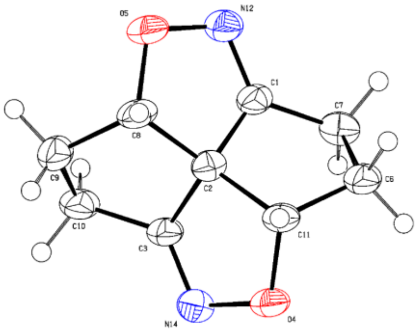

Supplementary Fig. 13 ORTEP diagram of **5a** (CCDC2233425)

Supplementary Table 6 Selected crystallographic data and collection parameters for **5a**.

formula	$C_9H_{10}N_2O_2$
formula weight	178.19
crystal system	triclinic
space group	P-1 (#2)
a	6.0825(5) Å
b	6.7565(5) Å
c	10.4390(8) Å
alpha	98.170(7) deg.
beta	105.510(7) deg.
gamma	99.688(7) deg.
volume	399.50(6) $Å^3$
Z	2
D (calcd)	1.481 g/cm^3
μ	8.877 cm^{-1}
F(000)	188.00
crystal size	0.700 × 0.700 × 0.600 mm
maximum 2θ	136.5 deg.
reflections collected	4078
independent reflections [*R*(int)]	0.0762
max. and min. transmission	0.587/0.362
goodness-of-fit on F^2	1.067
R_1 [$I > 2\sigma(I)$]	0.0781
R, wR_2 (all data)	0.2452
Weighting scheme	$R_1 = \Sigma \|\|Fo\| - \|Fc\|\| / \Sigma \|Fo\|$<br>$wR_2 = [\Sigma (w (Fo^2 - Fc^2)^2)/ \Sigma w(Fo^2)^2]^{1/2}$
largest diff. peak and hole	0.32 and -0.55 e $Å^{-3}$

Check CIF file of **5a** (CCDC2233425)

checkCIF/PLATON report

Structure factors have been supplied for datablock(s) HI9-3-58

THIS REPORT IS FOR GUIDANCE ONLY. IF USED AS PART OF A REVIEW PROCEDURE FOR PUBLICATION, IT SHOULD NOT REPLACE THE EXPERTISE OF AN EXPERIENCED CRYSTALLOGRAPHIC REFEREE.

No syntax errors found. CIF dictionary Interpreting this report

Datablock: HI9-3-58

Bond precision: C-C = 0.0065 A Wavelength=1.54187

Cell:	a=6.0825(5)	b=6.7565(5)	c=10.4390(8)
	alpha=98.170(7)	beta=105.510(7)	gamma=99.688(7)
Temperature:	123 K		

	Calculated	Reported
Volume	399.50(6)	399.50(6)
Space group	P -1	P -1
Hall group	-P 1	-P 1
Moiety formula	C9 H10 N2 O2	C9 H10 N2 O2
Sum formula	C9 H10 N2 O2	C9 H10 N2 O2
Mr	178.19	178.19
Dx,g cm-3	1.481	1.481
Z	2	2
Mu (mm-1)	0.886	0.888
F000	188.0	188.0
F000'	188.61	
h,k,lmax	7,8,12	7,8,12
Nref	1464	1413
Tmin,Tmax	0.564,0.587	0.362,0.587
Tmin'	0.512	

Correction method= # Reported T Limits: Tmin=0.362 Tmax=0.587
AbsCorr = MULTI-SCAN

Data completeness= 0.965 Theta(max)= 68.230

R(reflections)= 0.0781(844) wR2(reflections)= 0.2452(1413)

S = 1.067 Npar= 119

The following ALERTS were generated. Each ALERT has the format
test-name_ALERT_alert-type_alert-level.
Click on the hyperlinks for more details of the test.

Alert level C

```
PLAT029_ALERT_3_C _diffrn_measured_fraction_theta_full value Low .      0.965 Why?
PLAT250_ALERT_2_C Large U3/U1 Ratio for Average U(i,j) Tensor ....        2.1 Note
PLAT340_ALERT_3_C Low Bond Precision on  C-C Bonds ..............    0.0065 Ang.
PLAT906_ALERT_3_C Large K Value in the Analysis of Variance ......      4.206 Check
PLAT911_ALERT_3_C Missing FCF Refl Between Thmin & STh/L=    0.600         45 Report
PLAT934_ALERT_3_C Number of (Iobs-Icalc)/Sigma(W) > 10 Outliers ..          1 Check
```

Alert level G

```
CHEMS02_ALERT_1_G  Please check that you have entered the correct
            _publ_requested_category classification of your compound;
            FI or CI or EI for inorganic; FM or CM or EM for metal-organic;
            FO or CO or EO for organic.
            From the CIF: _publ_requested_category   CHOOSE FI FM FO CI CM CO or A
            From the CIF: _chemical_formula_sum :C9 H10 N2 O2
PLAT005_ALERT_5_G No Embedded Refinement Details Found  in the CIF     Please Do !
PLAT063_ALERT_4_G Crystal Size Possibly too Large for Beam Size ..       0.70 mm
PLAT154_ALERT_1_G The s.u.'s on the Cell Angles are Equal ..(Note)      0.007 Degree
PLAT180_ALERT_4_G Check Cell Rounding: # of Values Ending with 0 =          3 Note
PLAT343_ALERT_2_G Unusual sp3      Angle Range in Main Residue for         C2 Check
PLAT793_ALERT_4_G Model has Chirality at C8          (Centro SPGR)          S Verify
PLAT793_ALERT_4_G Model has Chirality at C11         (Centro SPGR)          S Verify
PLAT912_ALERT_4_G Missing # of FCF Reflections Above STh/L=  0.600          6 Note
PLAT978_ALERT_2_G Number C-C Bonds with Positive Residual Density.          0 Info
```

```
   0 ALERT level A = Most likely a serious problem - resolve or explain
   0 ALERT level B = A potentially serious problem, consider carefully
   6 ALERT level C = Check. Ensure it is not caused by an omission or oversight
  10 ALERT level G = General information/check it is not something unexpected

   2 ALERT type 1 CIF construction/syntax error, inconsistent or missing data
   3 ALERT type 2 Indicator that the structure model may be wrong or deficient
   5 ALERT type 3 Indicator that the structure quality may be low
   5 ALERT type 4 Improvement, methodology, query or suggestion
   1 ALERT type 5 Informative message, check
```

It is advisable to attempt to resolve as many as possible of the alerts in all categories. Often the minor alerts point to easily fixed oversights, errors and omissions in your CIF or refinement strategy, so attention to these fine details can be worthwhile. In order to resolve some of the more serious problems it may be necessary to carry out additional measurements or structure refinements. However, the purpose of your study may justify the reported deviations and the more serious of these should normally be commented upon in the discussion or experimental section of a paper or in the "special_details" fields of the CIF. checkCIF was carefully designed to identify outliers and unusual parameters, but every test has its limitations and alerts that are not important in a particular case may appear. Conversely, the absence of alerts does not guarantee there are no aspects of the results needing attention. It is up to the individual to critically assess their own results and, if necessary, seek expert advice.

Publication of your CIF in IUCr journals

A basic structural check has been run on your CIF. These basic checks will be run on all CIFs submitted for publication in IUCr journals (*Acta Crystallographica*, *Journal of Applied Crystallography*, *Journal of Synchrotron Radiation*); however, if you intend to submit to *Acta Crystallographica Section C* or *E* or *IUCrData*, you should make sure that full publication checks are run on the final version of your CIF prior to submission.

Publication of your CIF in other journals

Please refer to the *Notes for Authors* of the relevant journal for any special instructions relating to CIF submission.

PLATON version of 28/11/2022; check.def file version of 28/11/2022

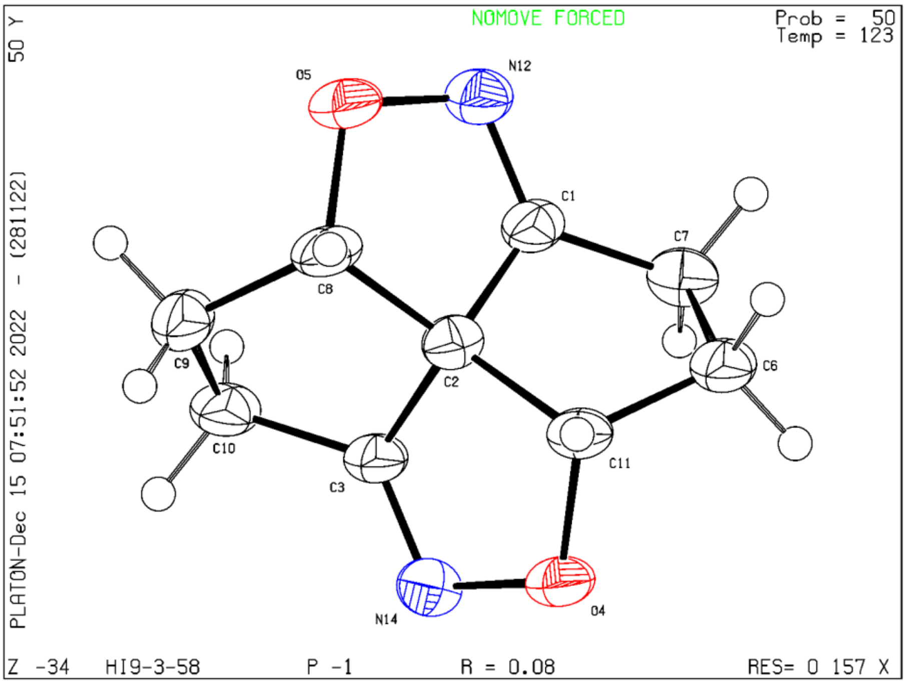

12. DFT calculation

(1) Calculation Details

All calculations were carried out using the Gaussian 16 program[17]. The DFT calculations were carried out using the B3LYP functional[18]. The 6-31G+(d,p) basis set was used for all atoms[19-22]. The optimized molecular structures were verified by vibrational analysis; equilibrium structures did not have imaginary frequencies and transition state structures had only one imaginary frequency corresponding to the reaction coordinate. The solvent effect was calculated at optimized geometry by using the PCM[23] and SMD[24] model.

(2) Calculation results and discussions

Supplementary Table 7 Calculated central angle of isoxazolidine fenestrane.

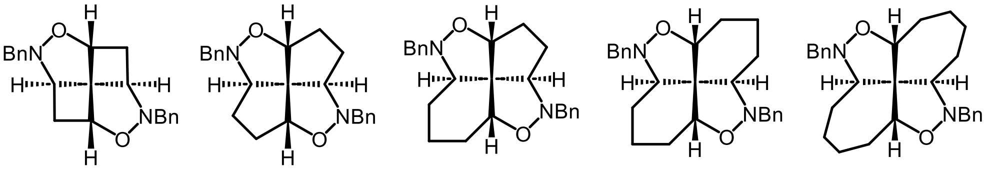

	c,c,c,c-[4.5.4.5] isoxazolidine	*c,c,c,c*-[5.5.5.5] isoxazolidine **2a**	*c,c,c,c*-[5.5.5.6] isoxazolidine **2r**	*c,c,c,c*-[5.6.5.6] isoxazolidine **2s**	*c,c,c,c*-[5.7.5.7] isoxazolidine **2t**
central angle	α = 131.0°<br>β = 126.7°	α = 117.3°<br>β = 116.6°	α = 114.1°<br>β = 114.3°	α = 110.2°<br>β = 116.6°	α = 110.5°<br>β = 112.6°

B3LYP/6-31G+(d,p)

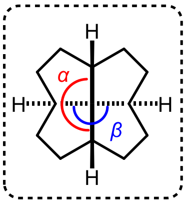

To estimate the extent of the flattening effect derived from the ring size, α and β values of the calculated most stable conformers of isoxazolidine fenestranes are shown in Supplementary Table 7. The α and β values of **2a** (α = 117.3°; β = 116.6°) and **2r** (α = 114.1°; β = 114.3°) based on the DFT calculation were similar to those of **2a** (α = 117.4°; β = 117.0°, Fig. 3) and **2r** (α = 114.4°; β = 114.2°, Fig. 3) based on the X-ray crystallographic analysis. The calculation results indicated that the ring contraction increases the degree of flattening of the quaternary carbon center, whereas the ring expansion decreases the degree of flattening. This tendency is consistent with that previously reported.[25]

Supplementary Table 8 Calculated central angle of isoxazoline fenestrane.

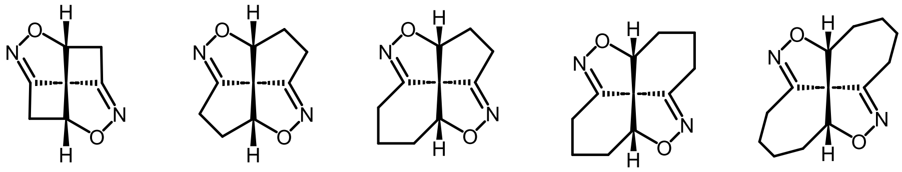

	c,c,c,c-[4.5.4.5] isooxazoline	*c,c,c,c*-[5.5.5.5] isooxazoline **5a**	*c,c,c,c*-[5.5.5.6] isooxazoline **5d**	*c,c,c,c*-[5.6.5.6] isooxazoline **5e**	*c,c,c,c*-[5.7.5.7] isooxazoline
central angle	α = 159.6°<br>β = 119.1°	α = 133.1°<br>β = 117.1°	α = 125.5°<br>β = 116.8°	α = 119.5°<br>β = 115.9°	α = 113.3°<br>β = 111.0°

B3LYP/6-31G+(d,p)

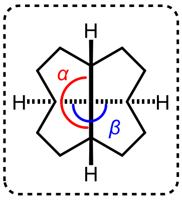

To estimate the extent of the flattening effect derived from the ring size, α and β values of the calculated most stable conformers of isoxazoline fenestranes are shown in Supplementary Table 8. The α and β values of **5a** (α = 133.1°; β = 117.1°) based on the DFT calculation were similar to those of **5a** (α = 134.7°; β = 114.9°, Fig. 3) based on the X-ray crystallographic analysis. Again, the calculation results indicated that the ring contraction increases the degree of flattening of the quaternary carbon center, whereas the ring expansion decreases the degree of flattening.

Supplementary Table 9 Conformation search for fenestranes **2b** and **5a**.

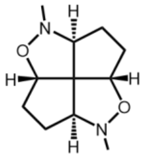

2b

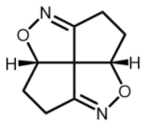

5a

compound	conformer	relative energy (kcal/mol)[a]	α	β
	1	0.00	117.6°	116.8°
2b	2	5.23	118.9°	116.8°
	3	5.31	117.6°	118.4°
	4	5.98	118.2°	118.6°
	1	0.00	133.1°	117.1°
5a	2	0.00	133.0°	117.1°

[a]B3LYP/6-31G+(d,p)

A conformation search was performed for diaza-dioxa-fenestranes **2b** (benzyl groups in **2a** were replaced with methyl groups to reduce the calculation cost) and **5a** (Supplementary Table 9). The four most stable conformers 1-4 of **2b** as well as the two most stable conformers 1 and 2 of **5a** were shown

with relative energy levels and α and β values. The calculated α and β values for the most stable conformer 1 of **2b** (α = 117.6°; β = 116.8°) were similar to those for **2a** determined by DFT calculation (α = 117.3°; β = 116.6°) and X-ray crystallography (α = 117.4°; β = 117.0°). Our calculation results indicated that the less stable conformers 2-4 have more flattened quaternary carbon centers. No stable conformer that has a less flattened quaternary carbon center than that in conformer 1 was found by the conformation search. On the other hand, only two stable conformers 1 and 2 were found in the case of **5a** presumably due to its rigid structure. These conformers are structurally very similar.

Supplementary Table 10 DFT calculations for sequential (3+2) cycloaddition of bisnitrile oxide in vacuum and solvents (DCE, THF, and toluene). **SM**: bisnitrile oxide substrate, **TS1**: transition state of the first cyclization of **SM**, **IM**: intermediate for the all-*cis*-fused diastereomer of the fenestrane, **TS2**: transition state of the second cyclization of **IM**, **TM**: all-*cis*-fused diastereomer of the fenestrane **5a**.

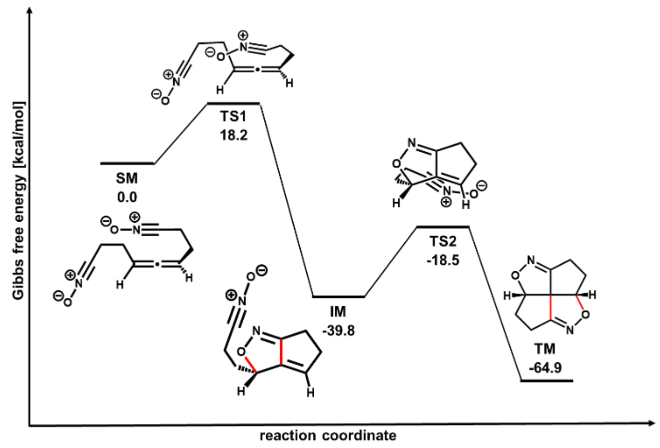

entry	solvent	Method	SM	TS1	IM	TS2	TM
1	-	-	0.0	18.2	-39.8 (0.0)	-18.5 (21.3)	-64.9 (-25.2)
2	DCE	PCM	0.0	18.5	-40.5 (0.0)	-17.6 (22.9)	-64.3 (-23.7)
3		SMD	0.0	19.0	-40.9 (0.0)	-17.4 (23.6)	-64.5 (-23.6)
4	THF	PCM	0.0	18.5	-40.5 (0.0)	-17.7 (22.8)	-64.3 (-23.8)
5		SMD	0.0	19.0	-40.7 (0.0)	-17.2 (23.5)	-64.3 (-23.6)
6	Toluene	PCM	0.0	18.4	-40.1 (0.0)	-17.9 (22.2)	-64.5 (-24.4)
7		SMD	0.0	19.0	-39.8 (0.0)	-16.6 (23.2)	-63.2 (-23.4)

Supplementary Tables 11 and 12 DFT calculations for sequential (3+2) cycloaddition of nitrone in vacuum and solvents (TCE, THF, and toluene). **a**, DFT calculation for the first (3+2) cycloaddition. **SM**$_{E}$: nitrone substrate with *E*-configuration, **SM**$_{Z}$: nitrone substrate with *Z*-configuration, **TS**$_{E,exo}$: transition state of *exo*-cyclization of **SM**$_{E}$, **TS**$_{E,endo}$: transition state of *endo-cyclization* of **SM**$_{E}$, **TS**$_{Z,exo}$: transition state of *exo*-cyclization of **SM**$_{Z}$, **TS**$_{Z,endo}$: transition state of *endo*-cyclization of **SM**$_{Z}$. **IM**$_{cis}$: bicyclic intermediate for the all-*cis*-fused diastereomer of the fenestrane. **IM**$_{trans}$: bicyclic intermediate for the *trans*-fused diastereomer of the fenestrane. **b**, DFT calculation for the second (3+2) cycloaddition. **IM**$_{E}$: nitrone intermediate with *E*-configuration, **IM**$_{Z}$: nitrone intermediate with *Z*-configuration, **TS**$_{E,exo}$: transition state of *exo*-cyclization of **IM**$_{E}$, **TS**$_{E,endo}$: transition state of *endo*-

cyclization of $\mathbf{IM}_{\boldsymbol{E}}$, $\mathbf{TS}_{\boldsymbol{Z,exo}}$: transition state of *exo*-cyclization of $\mathbf{IM}_{\boldsymbol{Z}}$, $\mathbf{TS}_{\boldsymbol{Z,endo}}$: transition state of *endo*-cyclization of $\mathbf{IM}_{\boldsymbol{Z}}$. $\mathbf{TM}_{\boldsymbol{trans}}$: *trans*-fused diastereomer of the fenestrane. $\mathbf{TM}_{\boldsymbol{cis}}$: all-*cis*-fused diastereomer of the fenestrane.

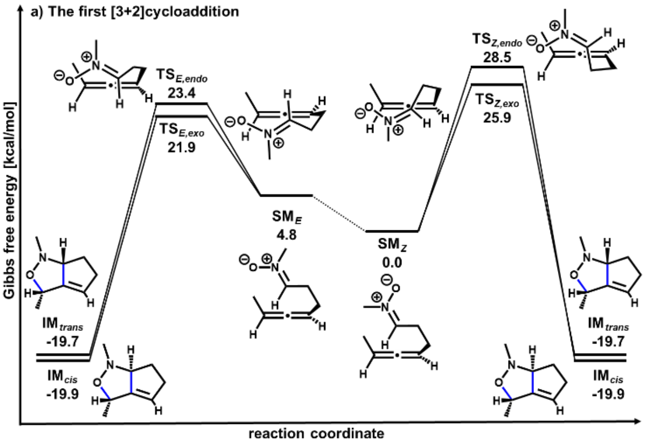

entry	solvent	Method	SM$_E$	TS$_{E,endo}$	IM$_{trans}$
1	-	-	4.8	23.4	-19.7
2	DCE	PCM	3.6	24.8	-16.8
3		SMD	3.5	25.4	-16.5
4	THF	PCM	4.1	24.2	-18.1
5		SMD	4.0	24.4	-18.3
6	Toluene	PCM	4.1	24.2	-18.0
7		SMD	4.0	25.0	-17.6

entry	solvent	Method	SM$_Z$	TS$_{Z,endo}$	IM$_{cis}$
1	-	-	0.0	28.5	-19.9
2	DCE	PCM	0.0	29.8	-17.0
3		SMD	0.0	30.2	-16.6
4	THF	PCM	0.0	29.2	-18.3
5		SMD	0.0	29.2	-18.5
6	Toluene	PCM	0.0	29.3	-18.2
7		SMD	0.0	29.8	-17.7

entry	solvent	Method	SM$_E$	TS$_{E,exo}$	IM$_{cis}$
1	-	-	4.8	21.9	-19.9
2	DCE	PCM	3.6	23.4	-17.0
3		SMD	3.5	23.9	-16.6
4	THF	PCM	4.1	22.7	-18.3
5		SMD	4.0	22.9	-18.5
6	Toluene	PCM	4.1	22.7	-18.2
7		SMD	4.0	23.4	-17.7

entry	solvent	Method	SM$_Z$	TS$_{Z,exo}$	IM$_{trans}$
1	-	-	0.0	25.9	-19.7
2	DCE	PCM	0.0	27.1	-16.8
3		SMD	0.0	27.6	-16.5
4	THF	PCM	0.0	26.6	-18.1
5		SMD	0.0	26.8	-18.3
6	Toluene	PCM	0.0	26.7	-18.0
7		SMD	0.0	27.3	-17.6

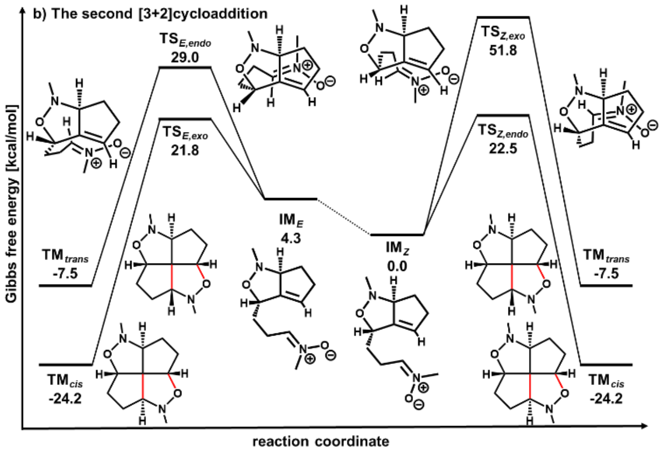

entry	solvent	Method	IM$_E$	TS$_{E,endo}$	TM$_{trans}$
1	-	-	4.3	29.0	-7.5
2	TCE	PCM	3.8	29.8	-4.4
3		SMD	3.9	30.5	-3.6
4	THF	PCM	4.0	29.4	-5.9
5		SMD	4.1	30.0	-5.6
6	Toluene	PCM	3.9	29.4	-5.7
7		SMD	4.1	30.6	-4.7

entry	solvent	Method	IM$_Z$	TS$_{Z,exo}$	IM$_{trans}$
1	-	-	0.0	51.8	-7.5
2	TCE	PCM	0.0	53.1	-4.4
3		SMD	0.0	54.1	-3.6
4	THF	PCM	0.0	52.5	-5.9
5		SMD	0.0	53.2	-5.6
6	Toluene	PCM	0.0	52.6	-5.7
7		SMD	0.0	53.9	-4.7

entry	solvent	Method	IM$_E$	TS$_{E,exo}$	TM$_{cis}$
1	-	-	4.3	21.8	-24.2
2	TCE	PCM	3.8	23.3	-21.1
3		SMD	3.9	24.2	-20.4
4	THF	PCM	4.0	22.6	-22.6
5		SMD	4.1	23.1	-22.4
6	Toluene	PCM	3.9	22.7	-22.5
7		SMD	4.1	23.8	-21.5

entry	solvent	Method	IM$_Z$	TS$_{Z,endo}$	IM$_{cis}$
1	-	-	0.0	22.5	-24.2
2	TCE	PCM	0.0	24.0	-21.1
3		SMD	0.0	25.0	-20.4
4	THF	PCM	0.0	23.3	-22.6
5		SMD	0.0	23.9	-22.4
6	Toluene	PCM	0.0	23.4	-22.5
7		SMD	0.0	24.5	-21.5

(3) Energy of calculated structures.

entry	Figure or Table	molecule	EQ or TS	Energy [hartree]
**1**	**Supplementary Fig. 5**	***c,c,c,c*-[5.5.5.5]-5*R*-*N*,*N′*-dibenzyl-5-hexyl-2,8-dioxa-3,9-diazafenestrane (2n)**	EQ	-1387.921701
**2**		***c,c,c,c*-[5.5.5.5]-5*S*-*N*,*N′*-dibenzyl-5-hexyl-2,8-dioxa-3,9-diazafenestrane (2n')**	EQ	-1387.920477
**3**	**Supplementary Fig. 6**	***c,c,c,c*-[5.5.5.5]-5*R*-*N*,*N′*-dibenzyl-5-benzyloxyl-2,8-dioxa-3,9-diazafenestrane (2p)**	EQ	-1497.6097906
**4**		***c,c,c,c*-[5.5.5.5]-5*S*-*N*,*N′*-dibenzyl-5-benzyloxyl-2,8-dioxa-3,9-diazafenestrane (2p')**	EQ	-1497.609669

5	Supplementary Fig. 8	*c,c*-[5.5.5.5]-6*R*-6- benzyloxyl -3,9-dioxa-1,7-diiminofenestrane (5c)	EQ	-954.4205114
6		*c,c*-[5.5.5.5]-6*S*-6- benzyloxyl -3,9-dioxa-1,7-diiminofenestrane (5c')	EQ	-954.4208855
7	Supplementary Fig. 9	*c,c,c*-[5.5.5.5]-3,9-dioxa-2-aza-1-(2-propenyl)-7-iminofenestrane (9a)	EQ	-726.759469
8		*t,c,c*-[5.5.5.5]-3,9-dioxa-2-aza-1-(2-propenyl)-7-iminofenestrane (9a')	EQ	-726.729537
9		*c,c,t*-[5.5.5.5]-3,9-dioxa-2-aza-1-(2-propenyl)-7-iminofenestrane (9a")	EQ	-726.722800
10		*c,t,c*-[5.5.5.5]-3,9-dioxa-2-aza-1-(2-propenyl)-7-iminofenestrane (9a"')	EQ	-726.664452
11	Supplementary Fig. 10	*c,c,c,c*-[5.5.5.5]-3,9-dioxa-2,8-diaza-1,7-di(2-propenyl)fenestrane (9b)	EQ	-844.678532
12		*c,c,t,c*-[5.5.5.5]-3,9-dioxa-2,8-diaza-1,7-di(2-propenyl)fenestrane (9b')	EQ	-844.649253
13		*t,c,t,c*-[5.5.5.5]-3,9-dioxa-2,8-diaza-1,7-di(2-propenyl)fenestrane (9b")	EQ	-844.591476
14		*c,c,c,t*-[5.5.5.5]-3,9-dioxa-2,8-diaza-1,7-di(2-propenyl)fenestrane (9b"')	EQ	-844.659885
15		*c,t,c,t*-[5.5.5.5]-3,9-dioxa-2,8-diaza-1,7-di(2-propenyl)fenestrane (9b"")	EQ	-844.627256
16	Supplementary Table 7	*c,c,c,c*-[4.5.4.5]isoxazolidine	EQ	-1073.3058523
17		(±)-*c,c,c,c*-[5.5.5.5]isoxazolidine 2a	EQ	-1152.0196686
18			EQ	-1152.0202112
19		(±)-*c,c,c,c*-[5.5.5.6]isoxazolidine 2r	EQ	-1191.3350151
20			EQ	-1191.335015
21		*c,c,c,c*-[5.6.5.6]isoxazolidine 2s	EQ	-1230.6476115
22		*c,c,c,c*-[5.7.5.7]isoxazolidine 2t	EQ	-1309.2642183
23	Supplementary Table 8	*c,c,c,c*-[4.5.4.5]isoxazoline	EQ	-530.0973371
24		(±)-*c,c*-[5.5.5.5]isoxazoline (5a)	EQ	-608.8323233
25			EQ	-608.8323233
26		*c,c,c,c*-[5.5.5.6]isoxazoline 5d	EQ	-648.1582435
27		*c,c,c,c*-[5.6.5.6]isoxazoline 5e	EQ	-687.4855972
28		*c,c,c,c*-[5.7.5.7]isoxazoline	EQ	-766.1226392

**29**	**Supplementary Table 10**	**SM**	EQ	-608.728840
**30**		**TS1**	TS	-608.699791
**31**		**IM**	EQ	-608.792203
**32**		**TS2**	TS	-608.758291
**33**		**TM**	EQ	-608.832323
**34**	**Fig. 4a**	$\mathbf{SM}_{E}$	EQ	-442.559686
**35**		$\mathbf{SM}_{Z}$	EQ	-442.567293
**36**		$\mathbf{TS}_{E,endo}$	TS	-442.529947
**37**		$\mathbf{TS}_{E,exo}$	TS	-442.532410
**38**		$\mathbf{TS}_{Z,endo}$	TS	-442.521929
**39**		$\mathbf{TS}_{Z,exo}$	TS	-442.526002
**40**		$\mathbf{IM}_{trans}$	EQ	-442.598743
**41**		$\mathbf{IM}_{cis}$	EQ	-442.598929
**42**	**Fig. 4b**	$\mathbf{IM}_{E}$	EQ	-689.845621
**43**		$\mathbf{IM}_{Z}$	EQ	-689.852487
**44**		$\mathbf{TS}_{E,endo}$	TS	-689.806346
**45**		$\mathbf{TS}_{E,exo}$	TS	-689.817748
**46**		$\mathbf{TS}_{Z,endo}$	TS	-689.770012
**47**		$\mathbf{TS}_{Z,exo}$	TS	-689.816654
**48**		$\mathbf{TM}_{cis}$	EQ	-689.891127
**49**		$\mathbf{TM}_{trans}$	EQ	-689.864504

13. NMR spectra

2-(Pent-4-yn-1-yloxy)tetrahydro-2*H*-pyran (S2a)

(^{1}H NMR, 400 MHz, $CDCl_3$)

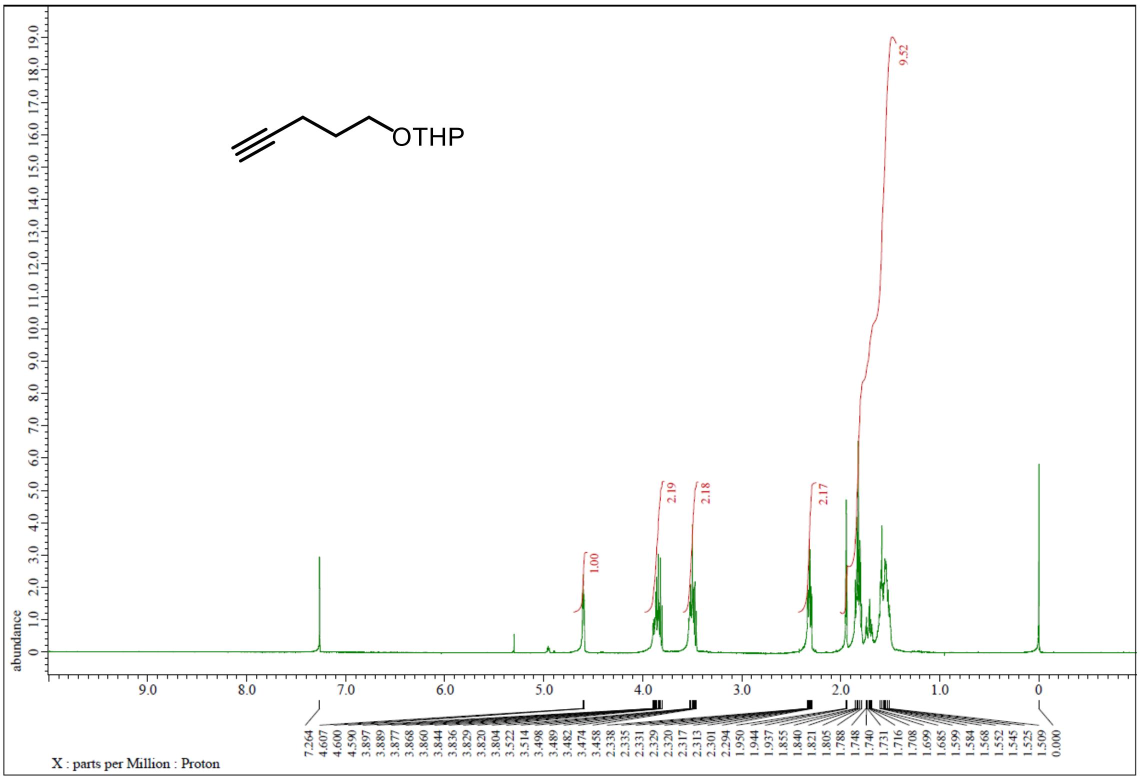

(^{13}C NMR, 100 MHz, $CDCl_3$)

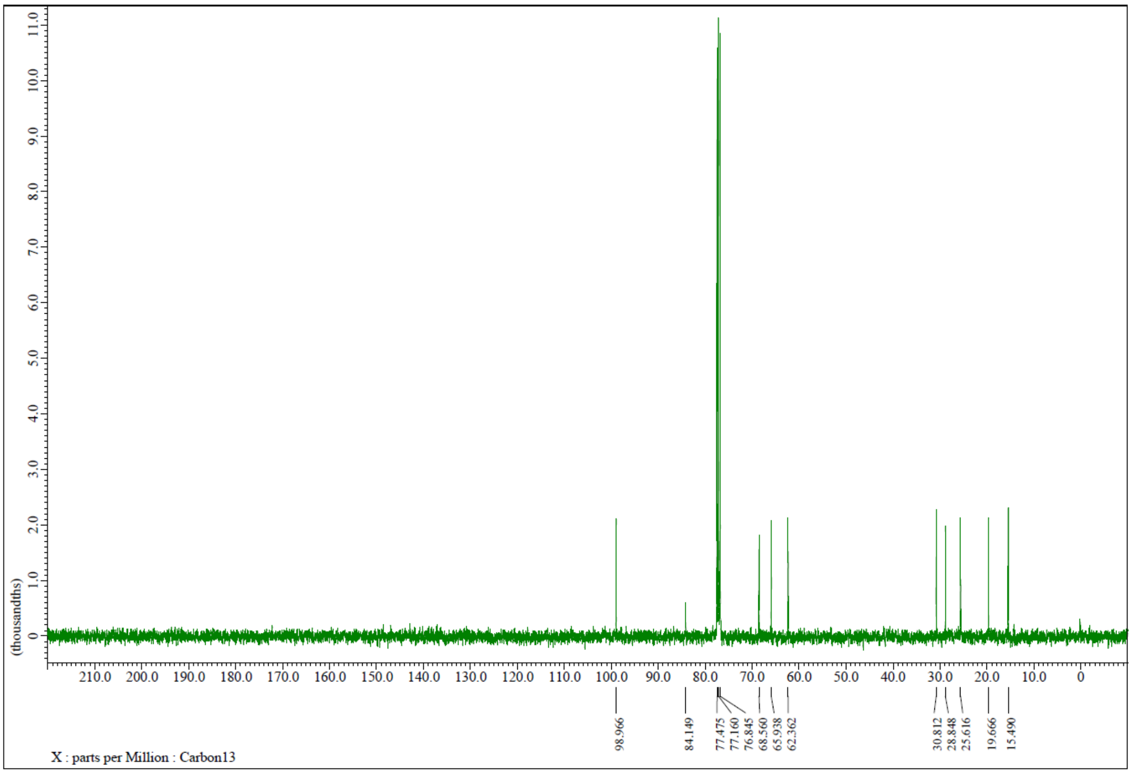

Ethyl 2-(prop-2-yn-1-yl)octanoate (S3)

(^{1}H NMR, 400 MHz, $CDCl_3$)

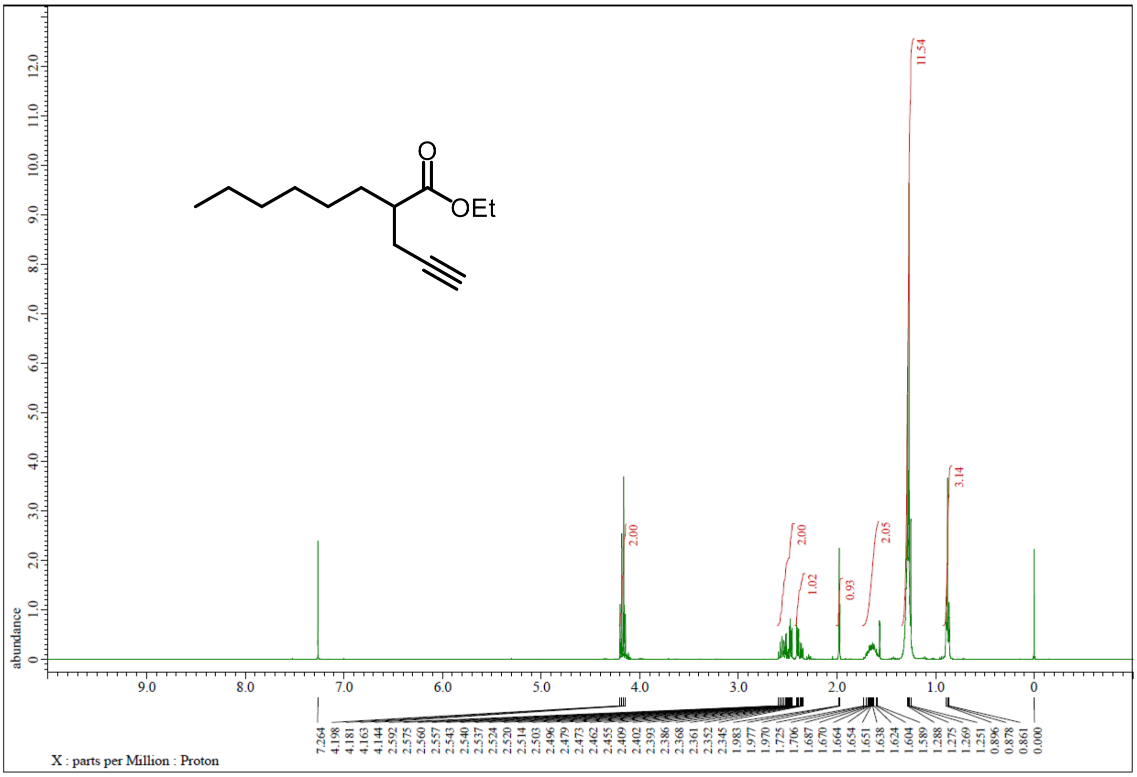

(^{13}C NMR, 100 MHz, $CDCl_3$)

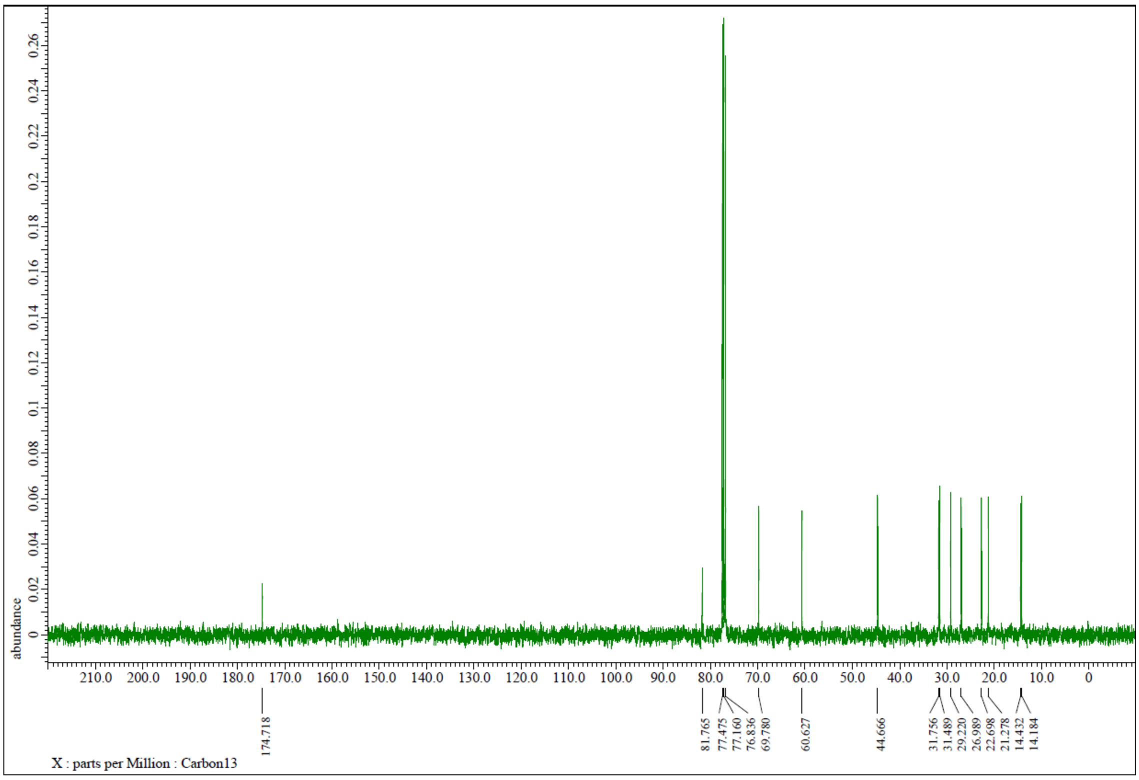

2-(Prop-2-yn-1-yl)octan-1-ol (S1b)

(^{1}H NMR, 400 MHz, $CDCl_3$)

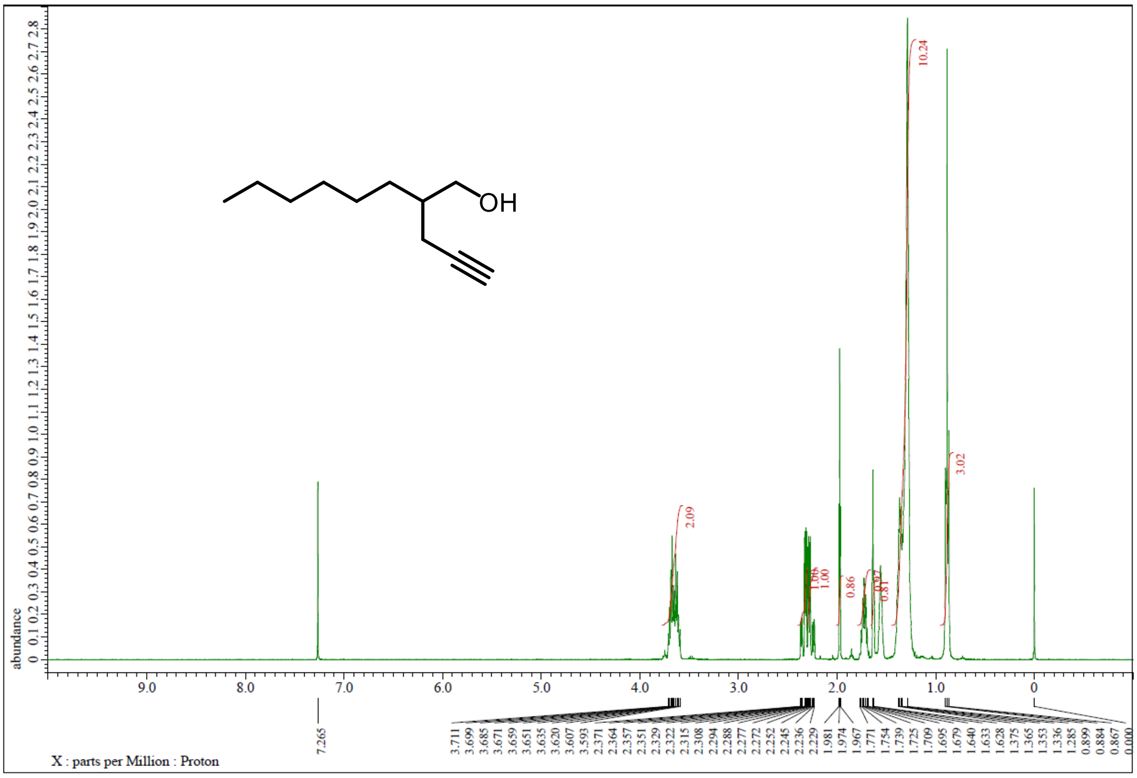

(^{13}C NMR, 100 MHz, $CDCl_3$)

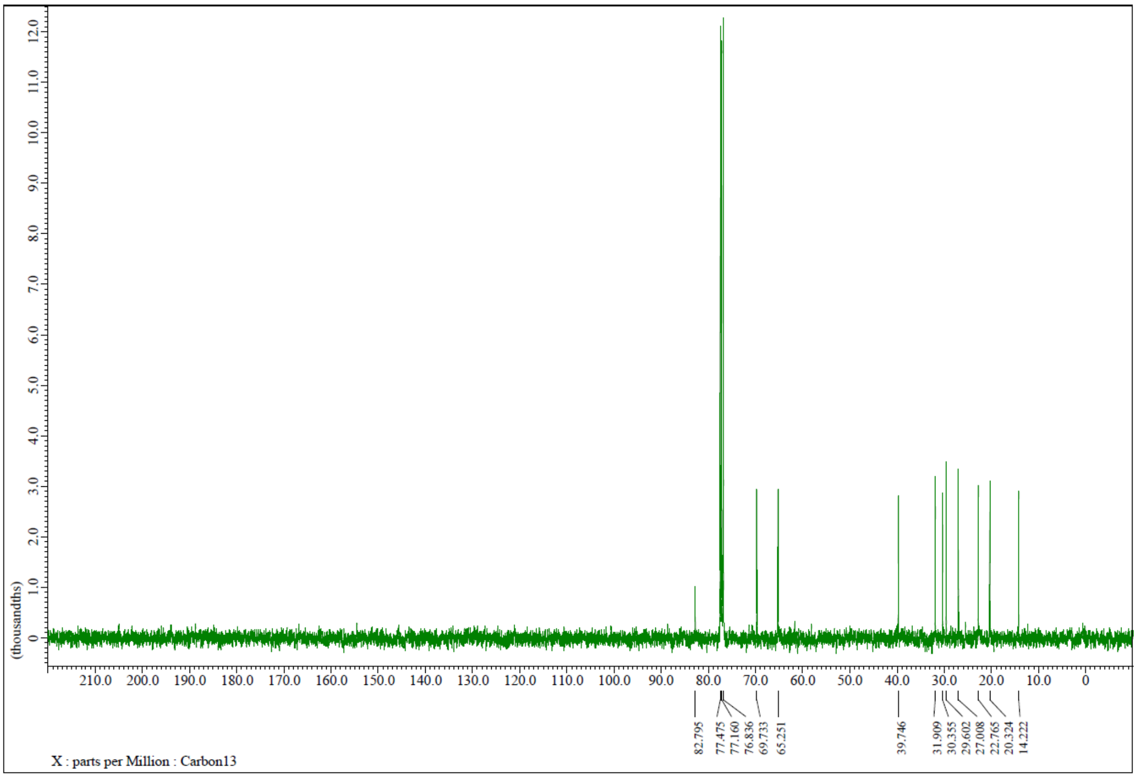

2-((2-(Prop-2-yn-1-yl)octyl)oxy)tetrahydro-2*H*-pyran (S2b)

(^{1}H NMR, 400 MHz, $CDCl_3$)

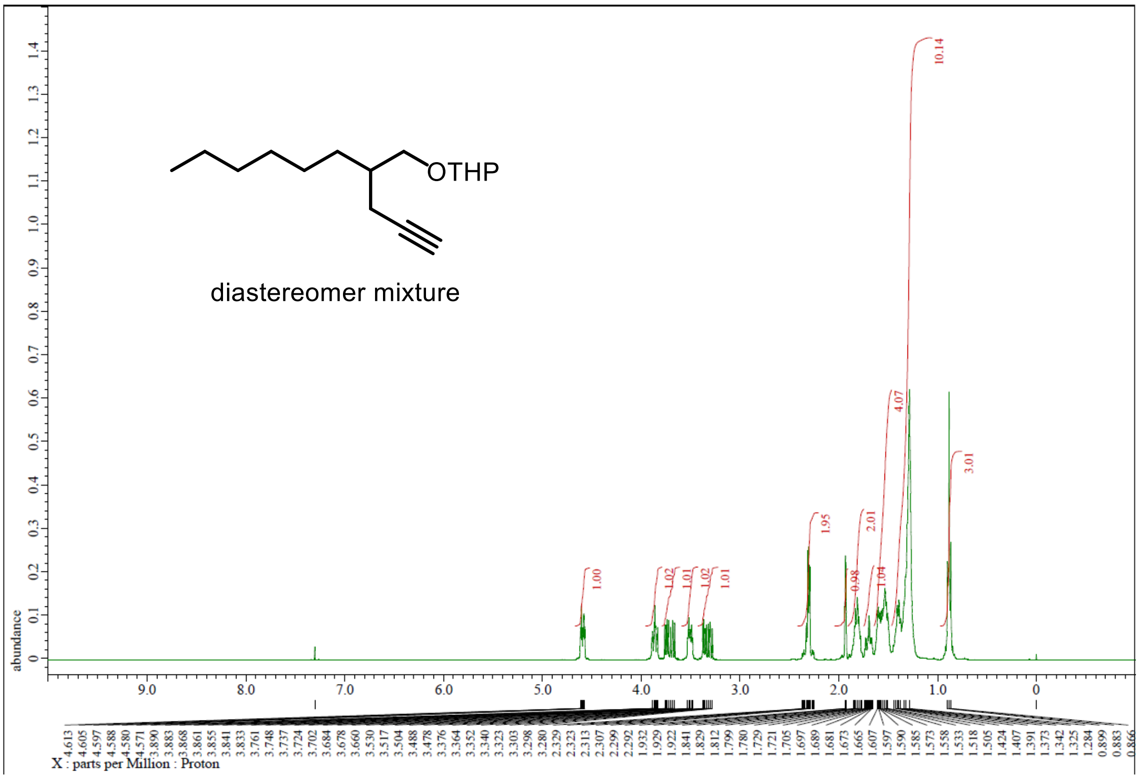

(^{13}C NMR, 100 MHz, $CDCl_3$)

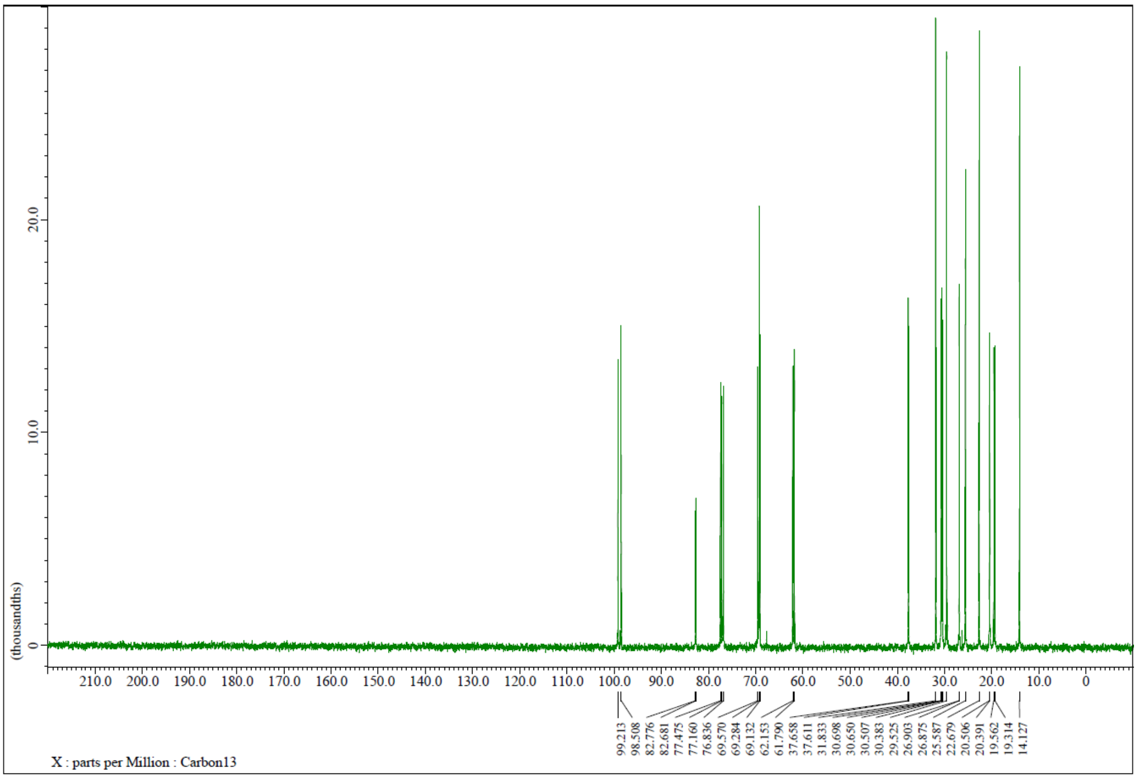

***t*-Butyl 2-(benzyloxy)acetate (S4)**

(^{1}H NMR, 400 MHz, $CDCl_3$)

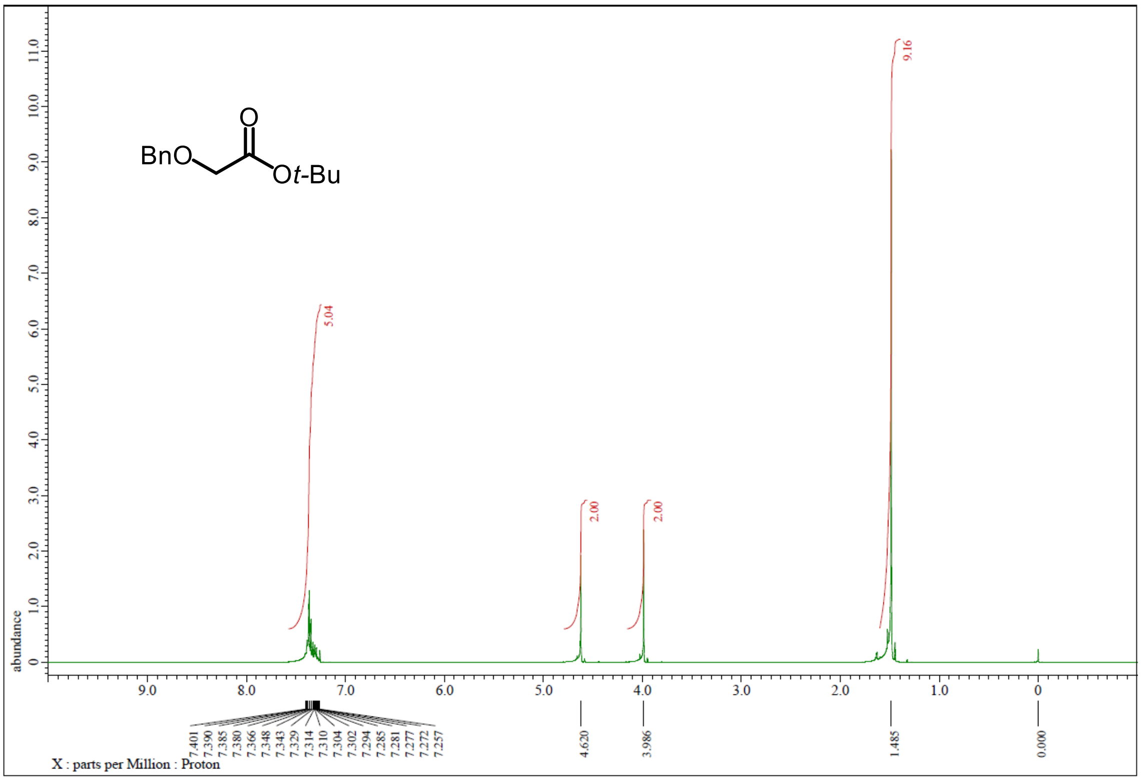

(^{13}C NMR, 100 MHz, $CDCl_3$)

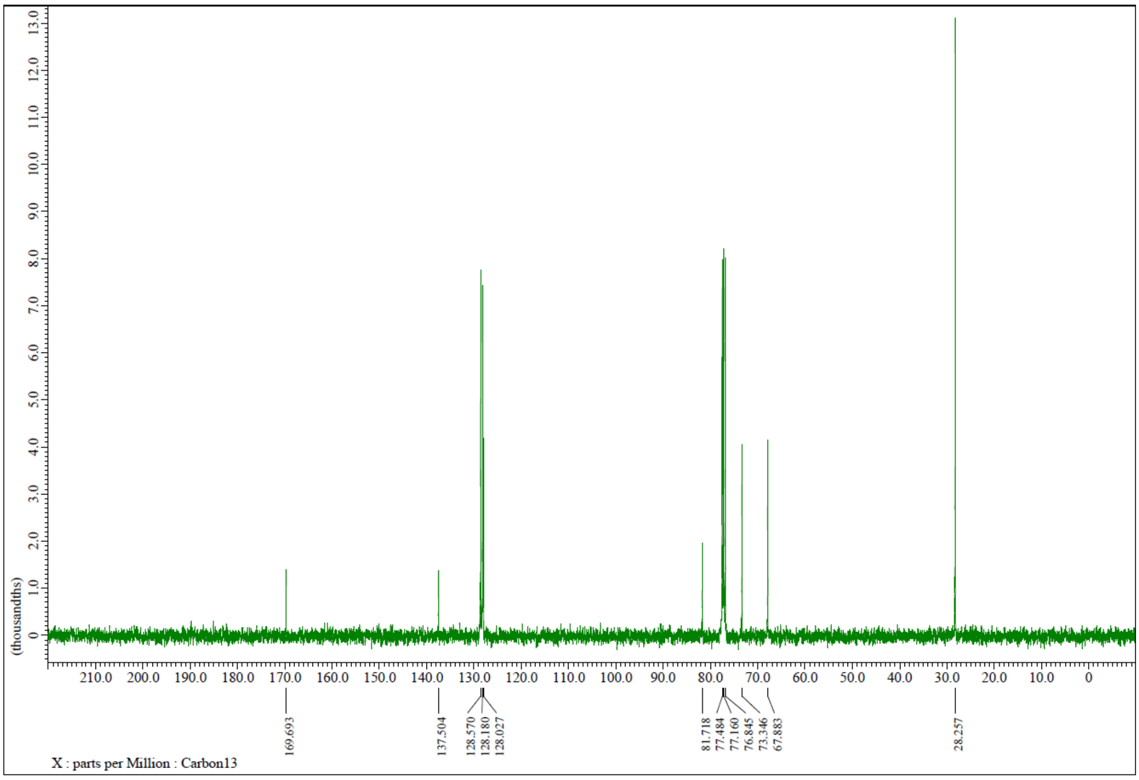

t-Butyl 2-(benzyloxy)pent-4-ynoate (S5)

(^{1}H NMR, 400 MHz, $CDCl_3$)

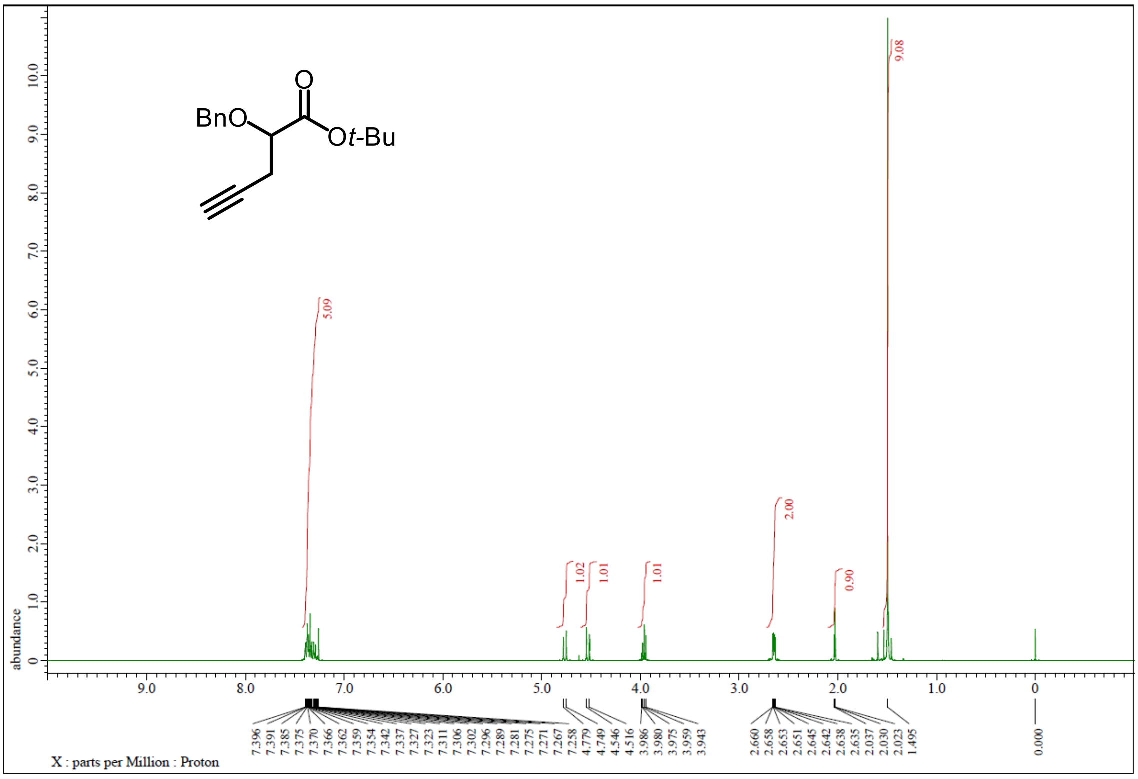

(^{13}C NMR, 100 MHz, $CDCl_3$)

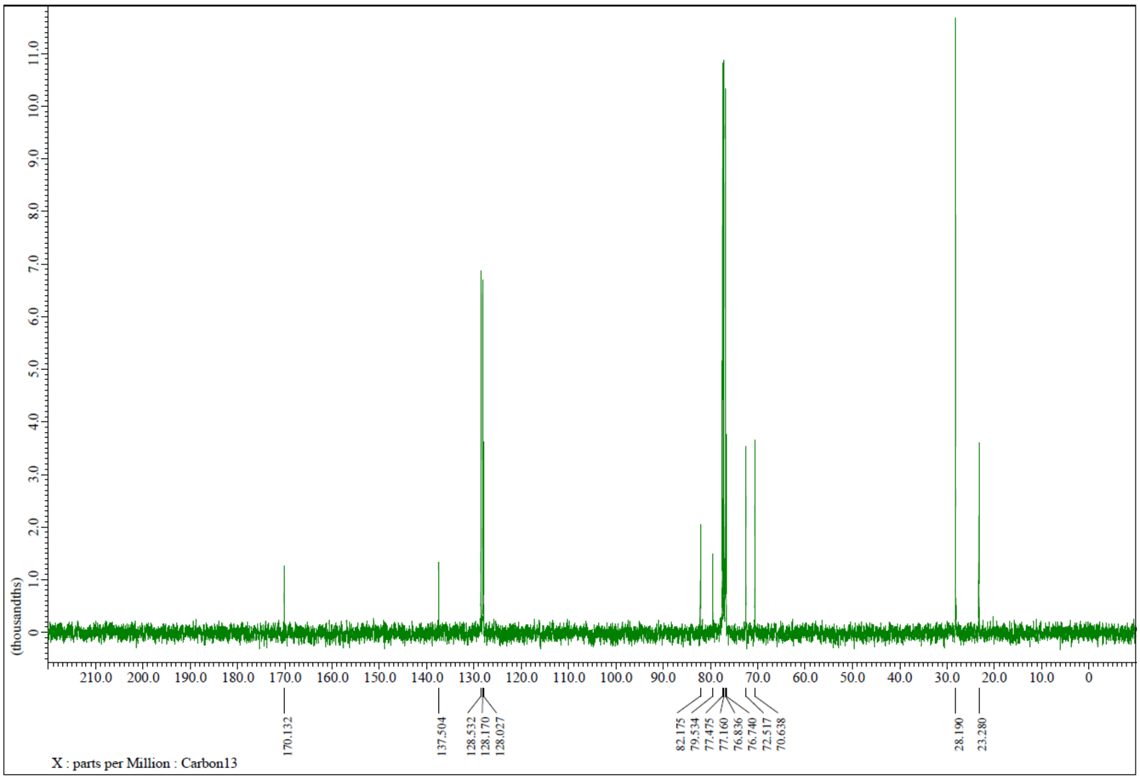

2-(Benzyloxy)pent-4-yn-1-ol (S1c)

(^{1}H NMR, 400 MHz, $CDCl_3$)

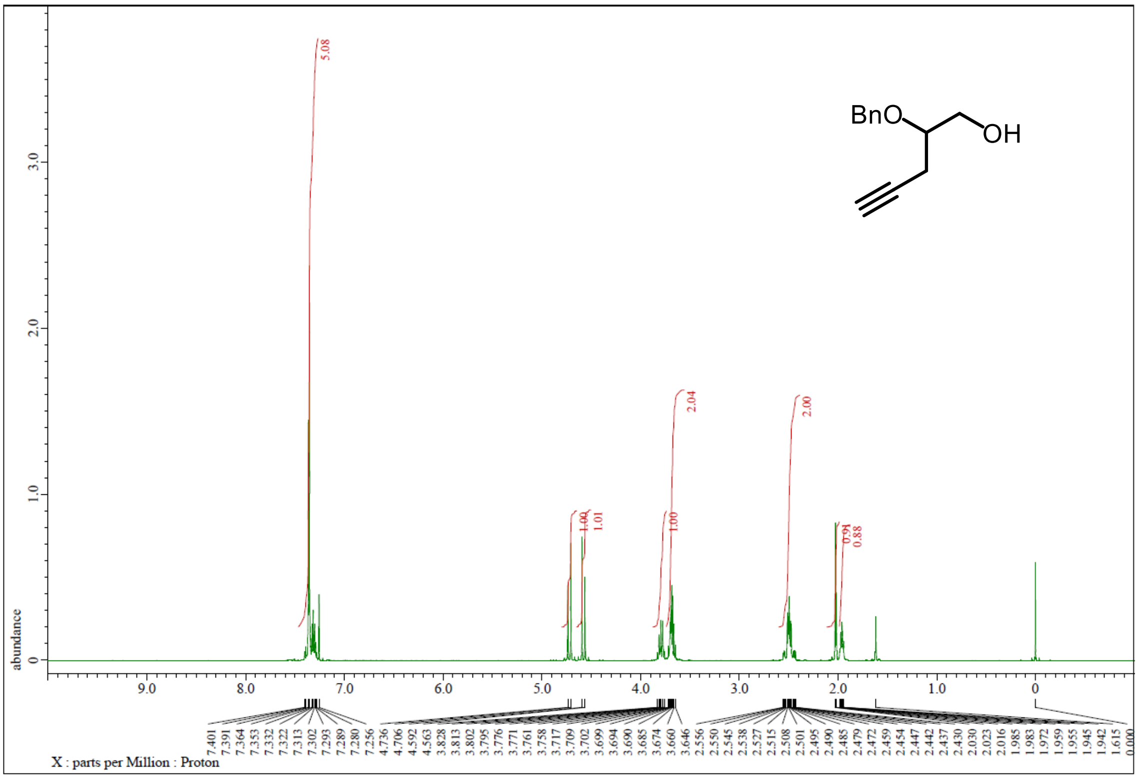

(^{13}C NMR, 100 MHz, $CDCl_3$)

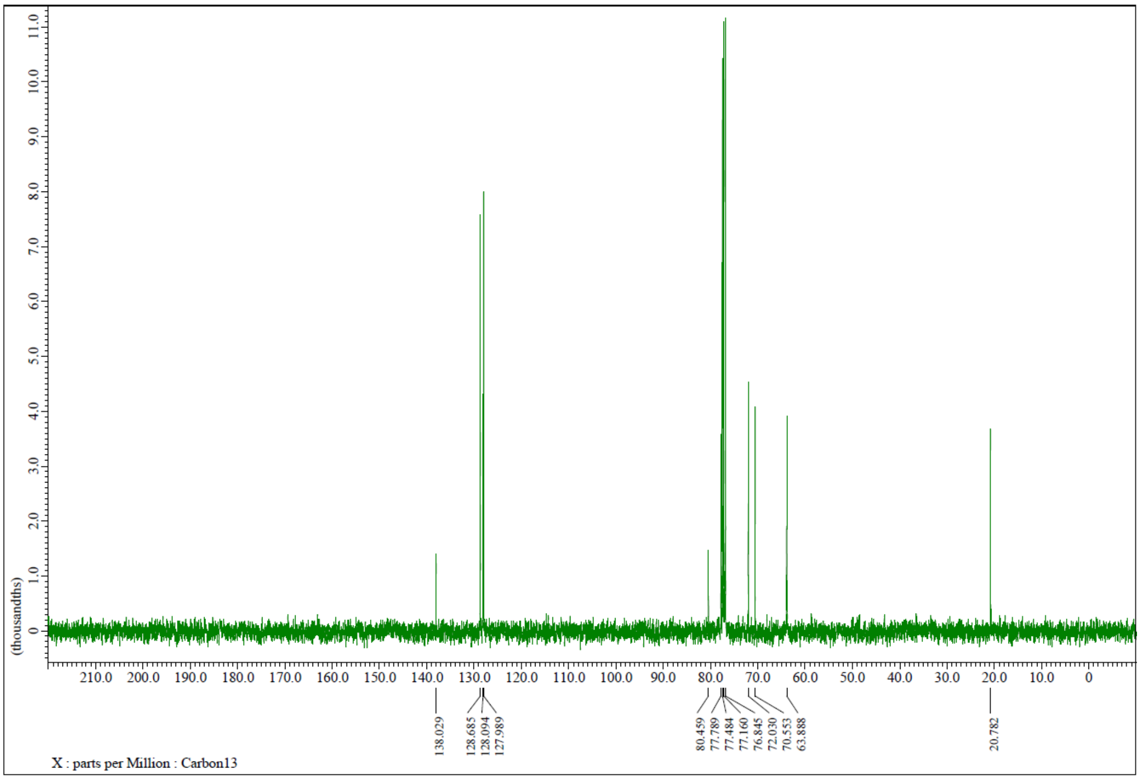

2-((2-(Benzyloxy)pent-4-yn-1-yl)oxy)tetrahydro-2*H*-pyran (S2c)

(^{1}H NMR, 400 MHz, $CDCl_3$)

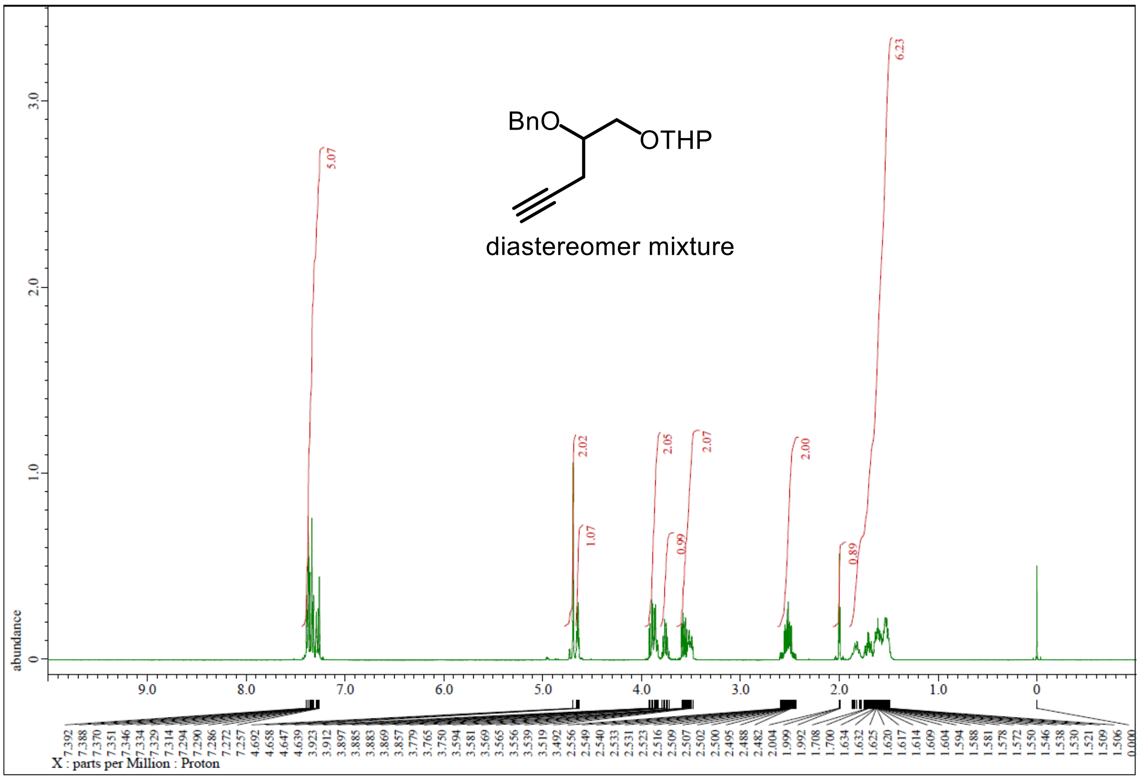

(^{13}C NMR, 100 MHz, $CDCl_3$)

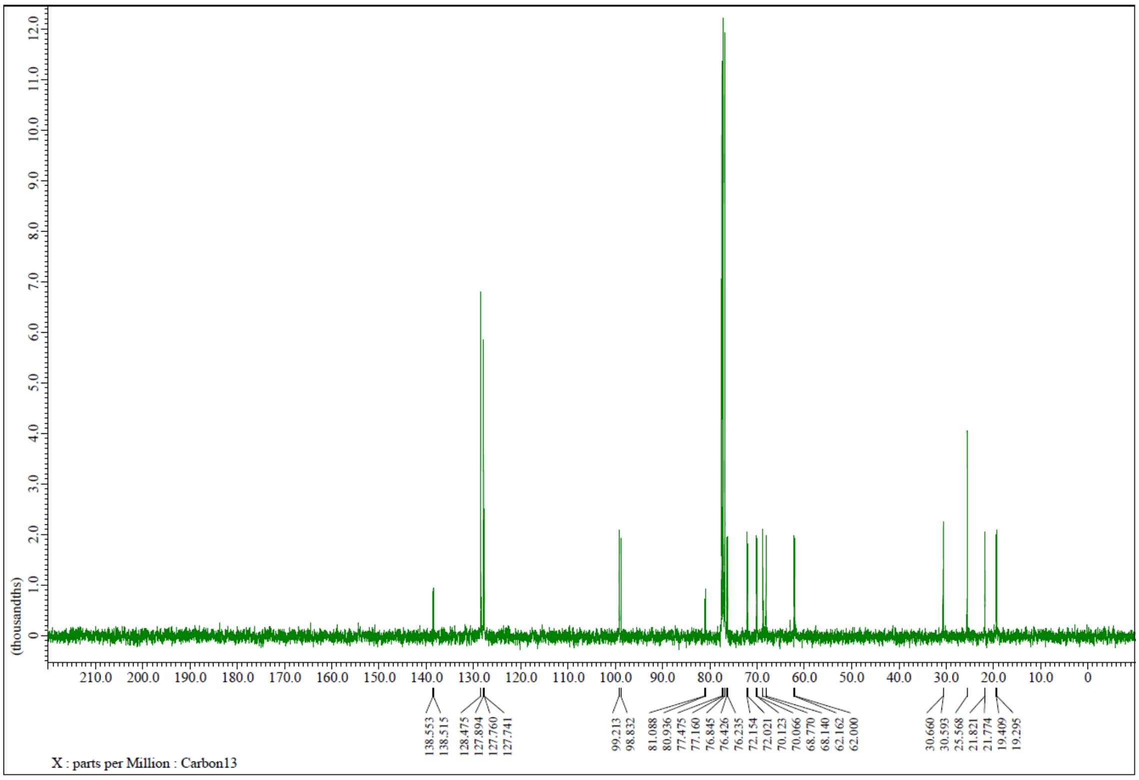

2-(Hex-5-yn-1-yloxy)tetrahydro-2*H*-pyran (S2d)

(^{1}H NMR, 400 MHz, $CDCl_3$)

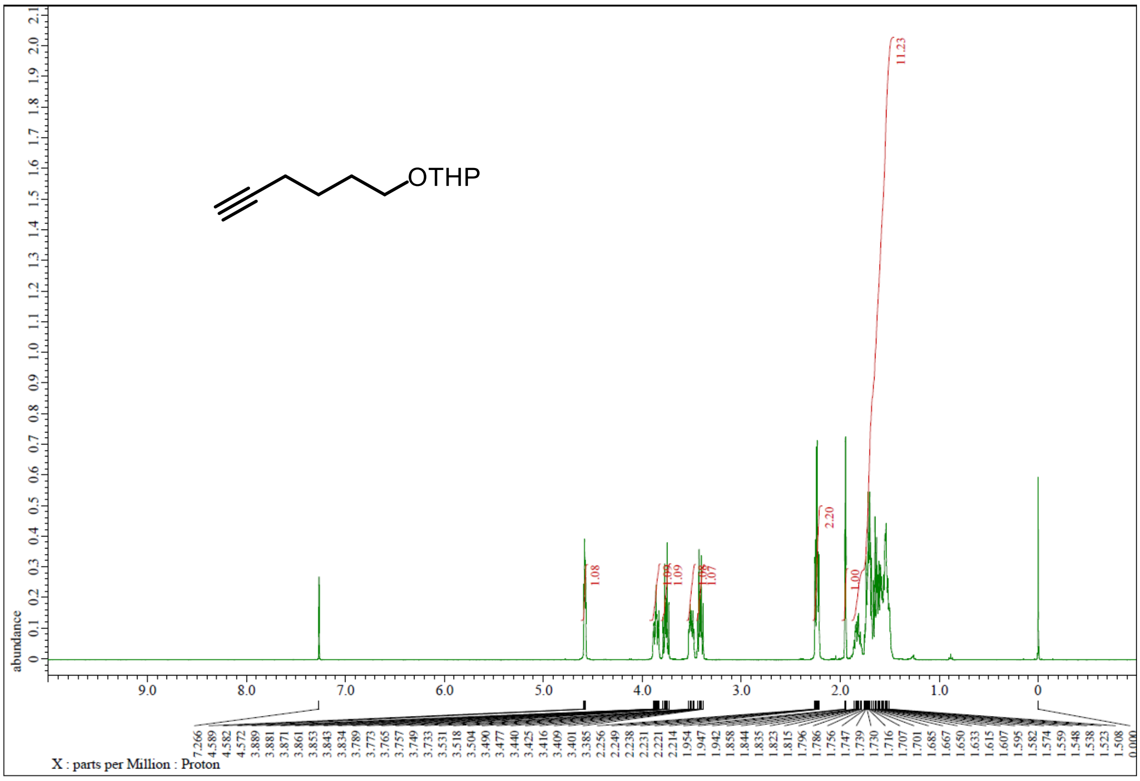

(^{13}C NMR, 100 MHz, $CDCl_3$)

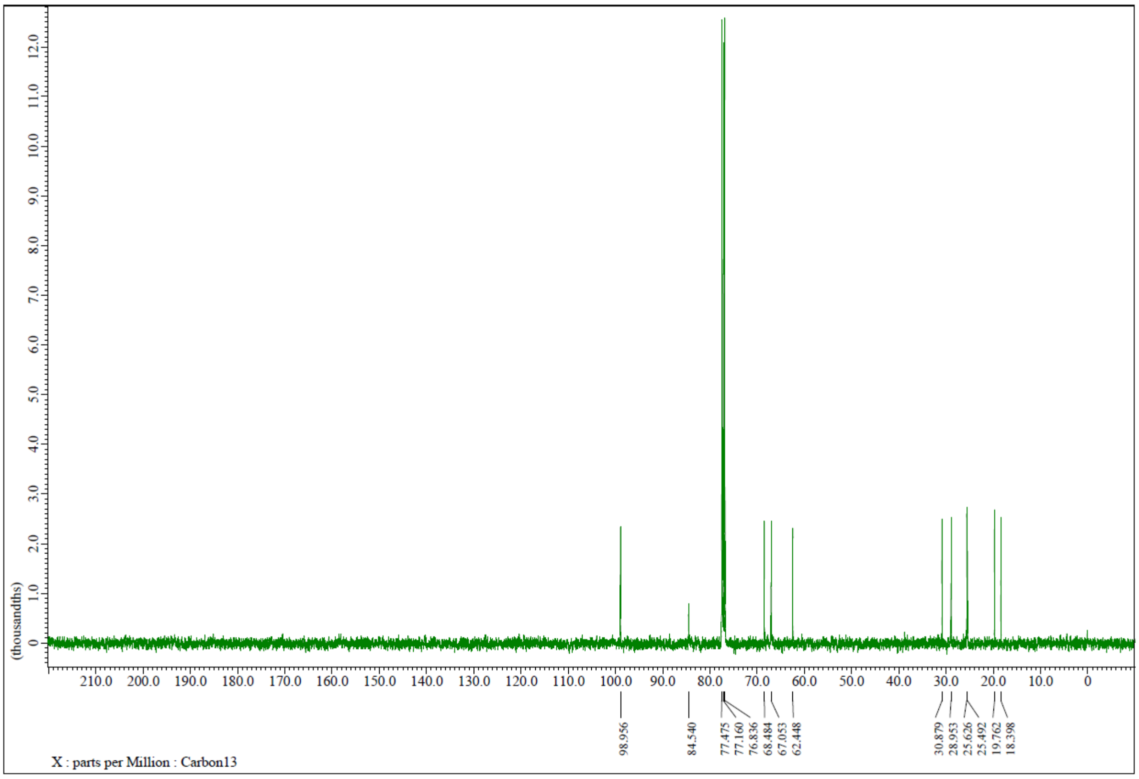

2-(Hept-6-yn-1-yloxy)tetrahydro-2H-pyran (S2e)

(^1H NMR, 400 MHz, $CDCl_3$)

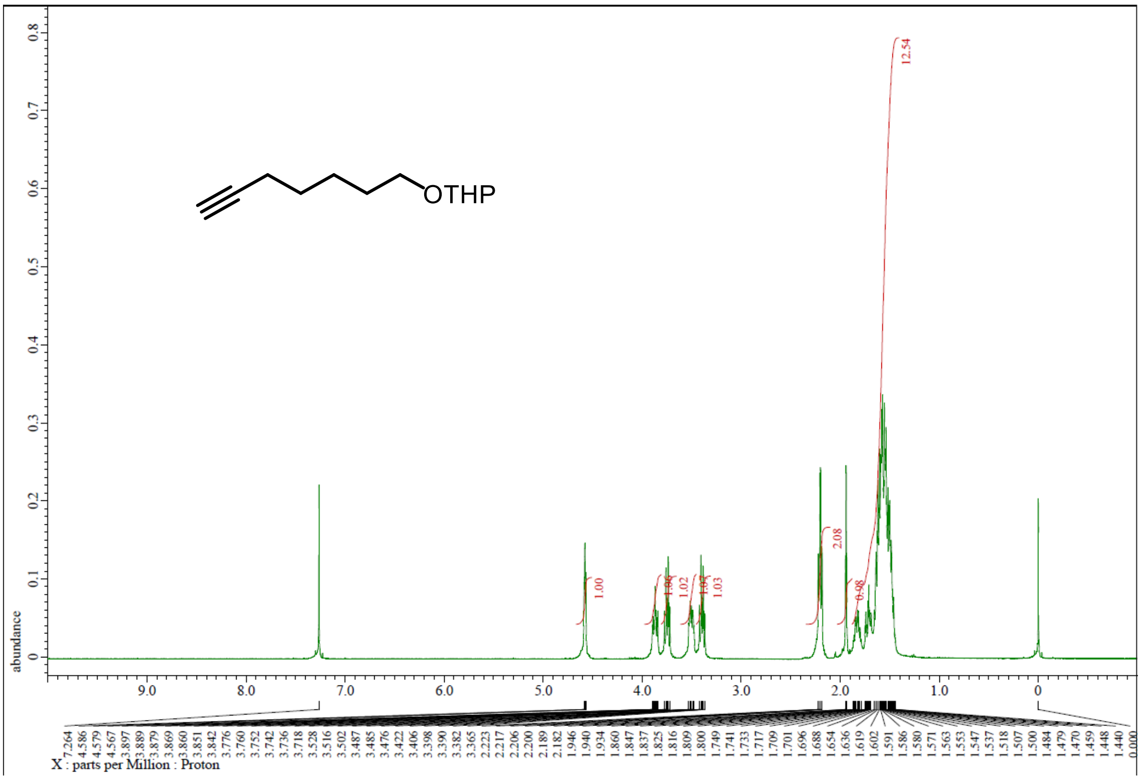

(^{13}C NMR, 100 MHz, $CDCl_3$)

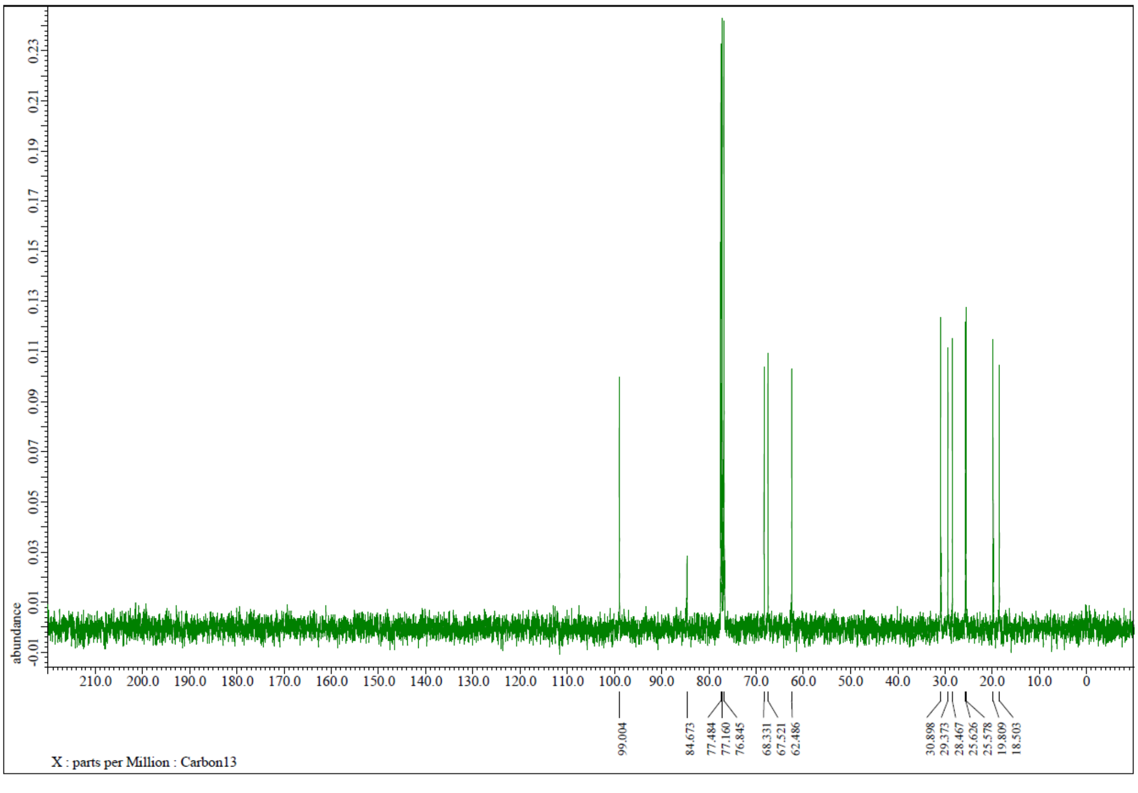

4-((Tetrahydro-2*H*-pyran-2-yl)oxy)butan-1-ol (S6a)

(^{1}H NMR, 400 MHz, $CDCl_3$)

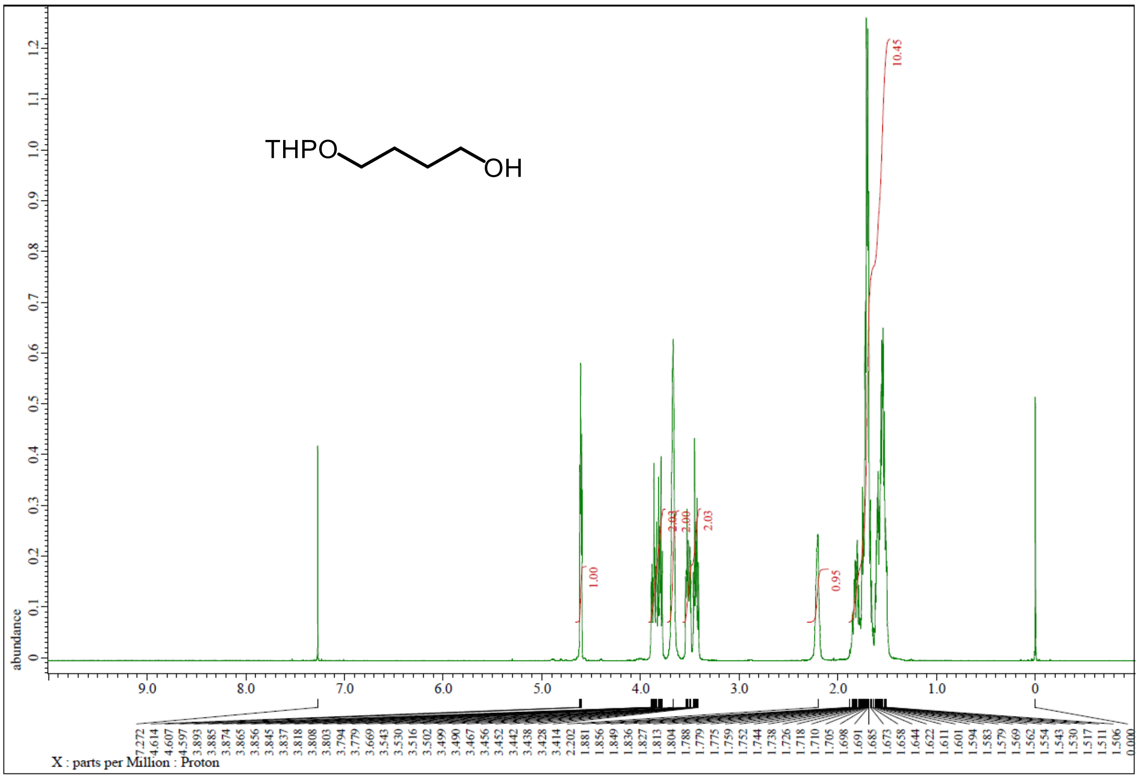

(^{13}C NMR, 100 MHz, $CDCl_3$)

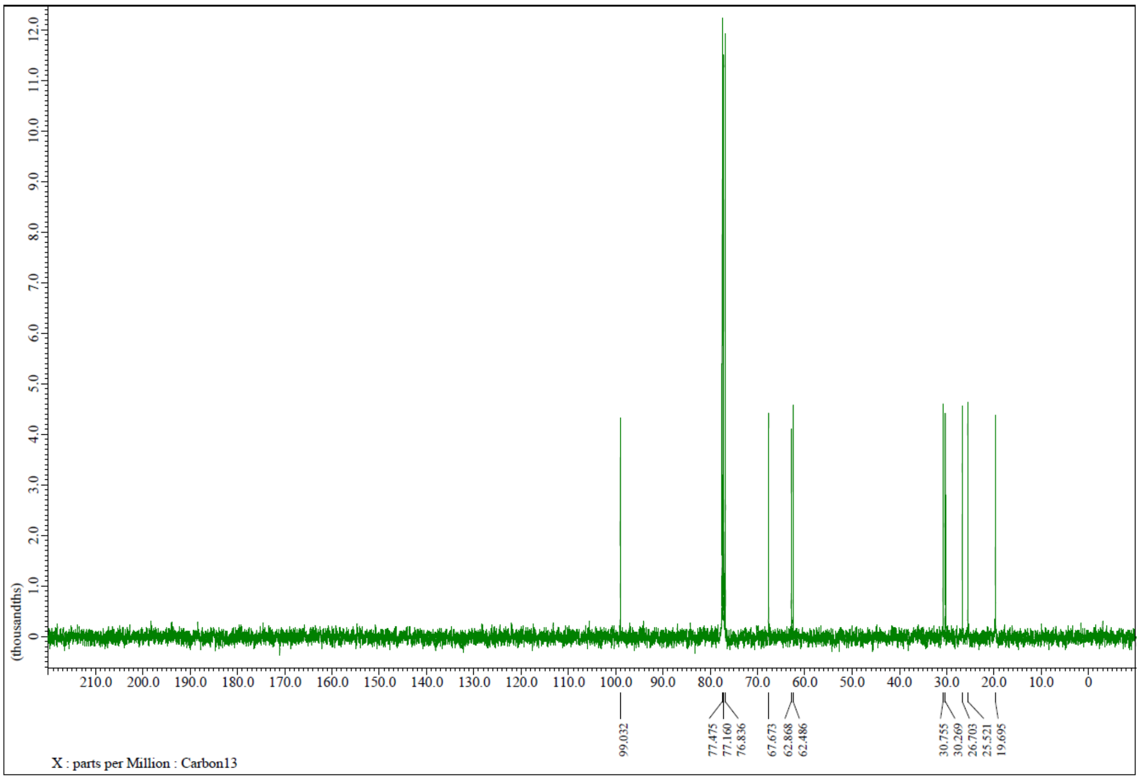

5-((Tetrahydro-2*H*-pyran-2-yl)oxy)pentan-1-ol (S6b)

(^{1}H NMR, 400 MHz, $CDCl_3$)

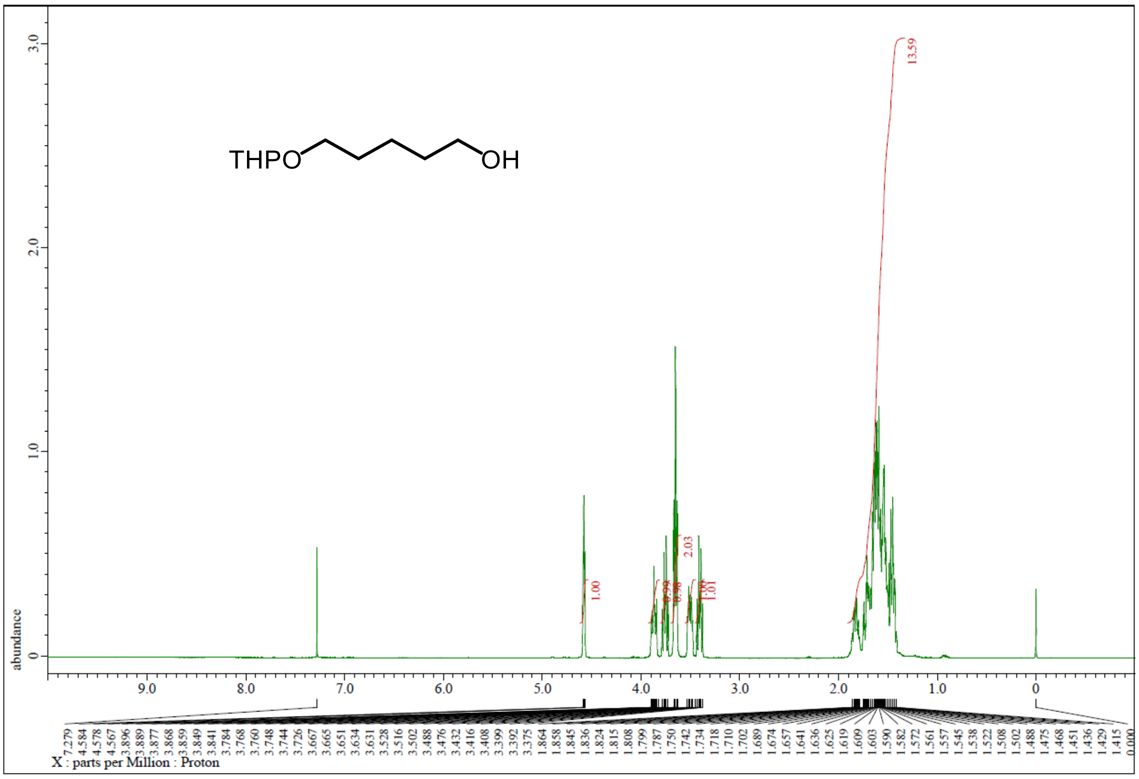

(^{13}C NMR, 100 MHz, $CDCl_3$)

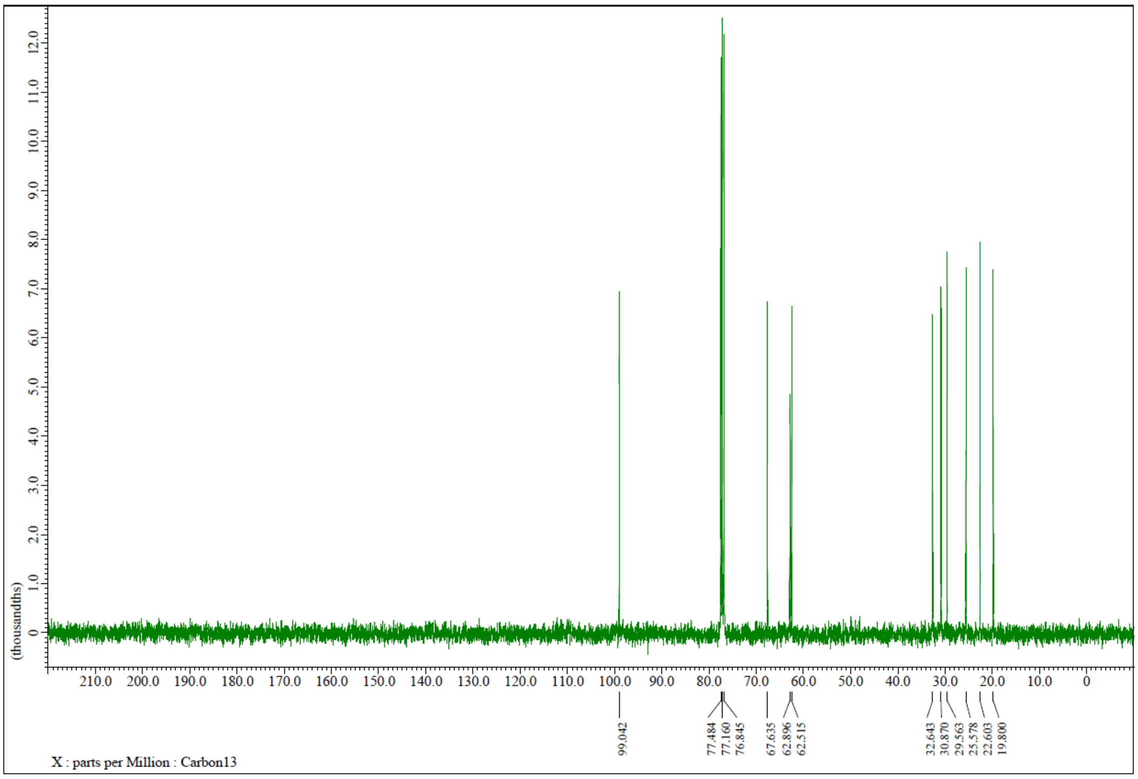

6-((Tetrahydro-2*H*-pyran-2-yl)oxy)hexan-1-ol (S6c)

(^{1}H NMR, 400 MHz, CDCl$_3$)

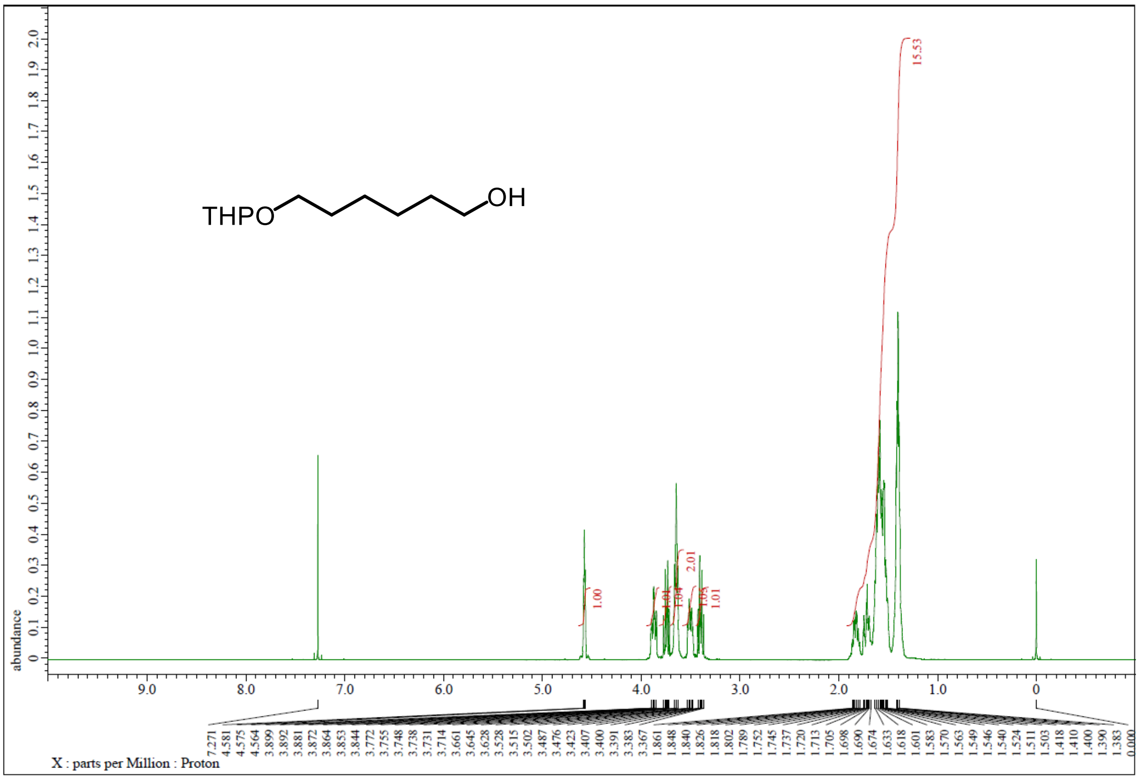

(^{13}C NMR, 100 MHz, CDCl$_3$)

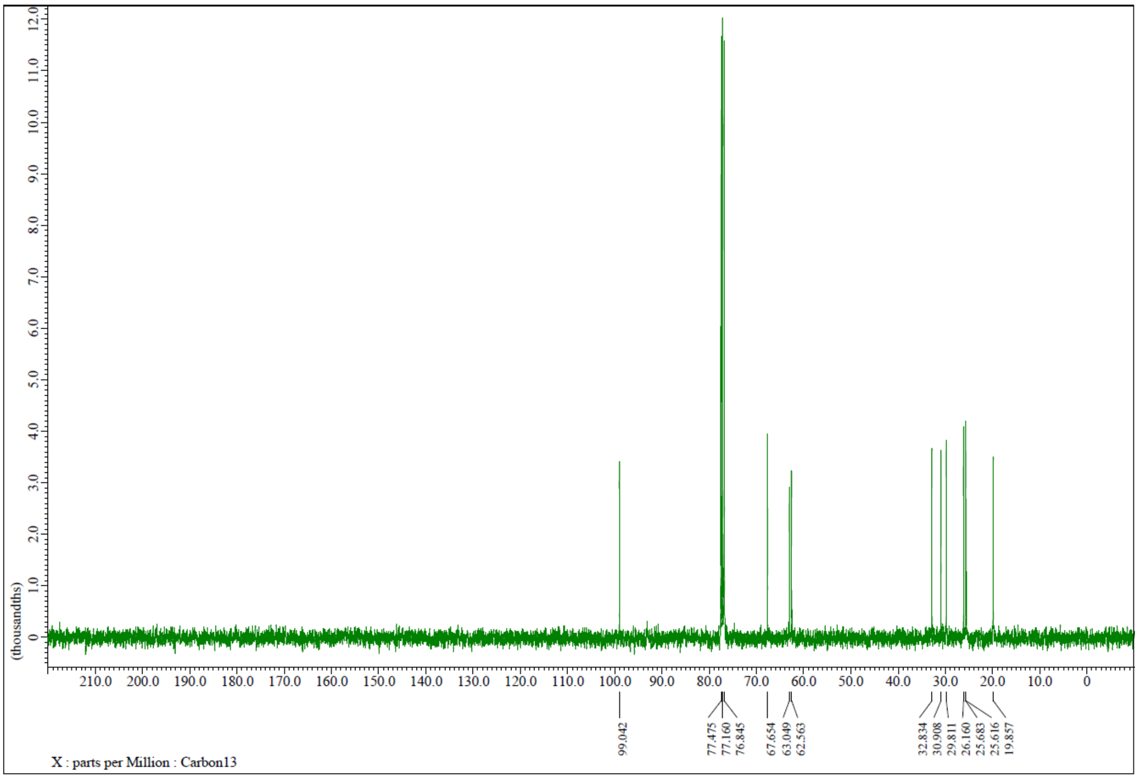

1,9-Bis((tetrahydro-2*H*-pyran-2-yl)oxy)non-5-yn-4-ol (S8a)

(^{1}H NMR, 400 MHz, $CDCl_3$)

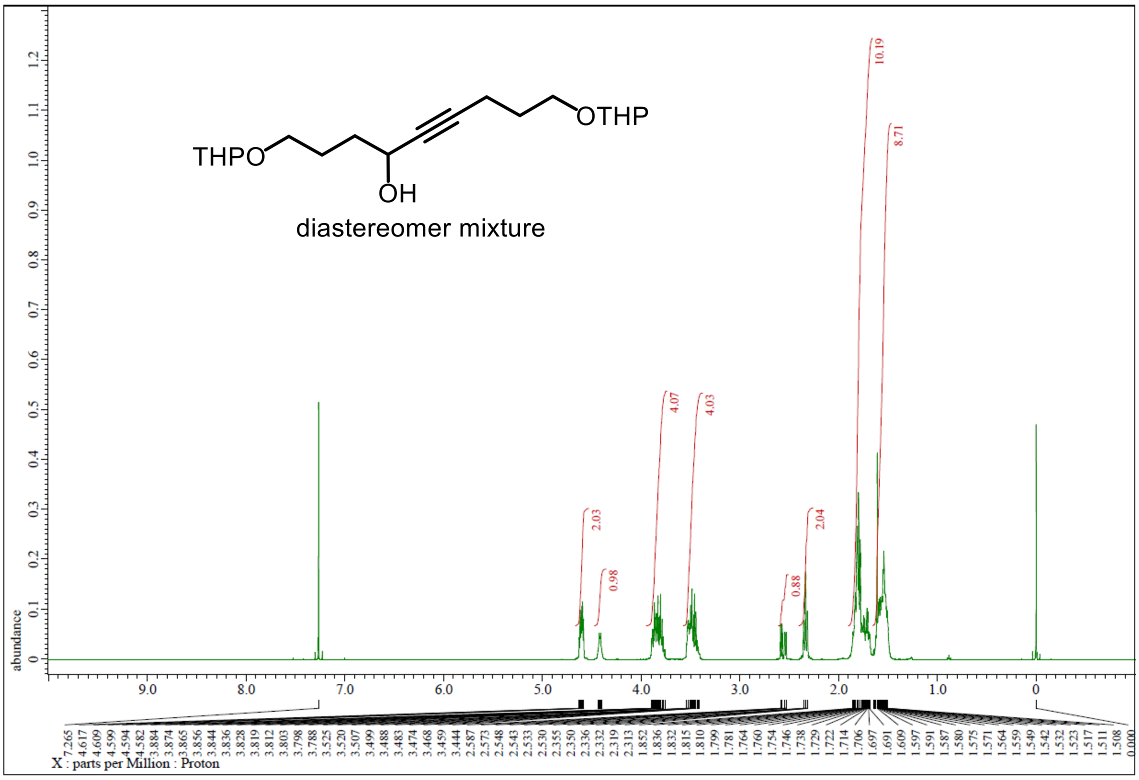

(^{13}C NMR, 100 MHz, $CDCl_3$)

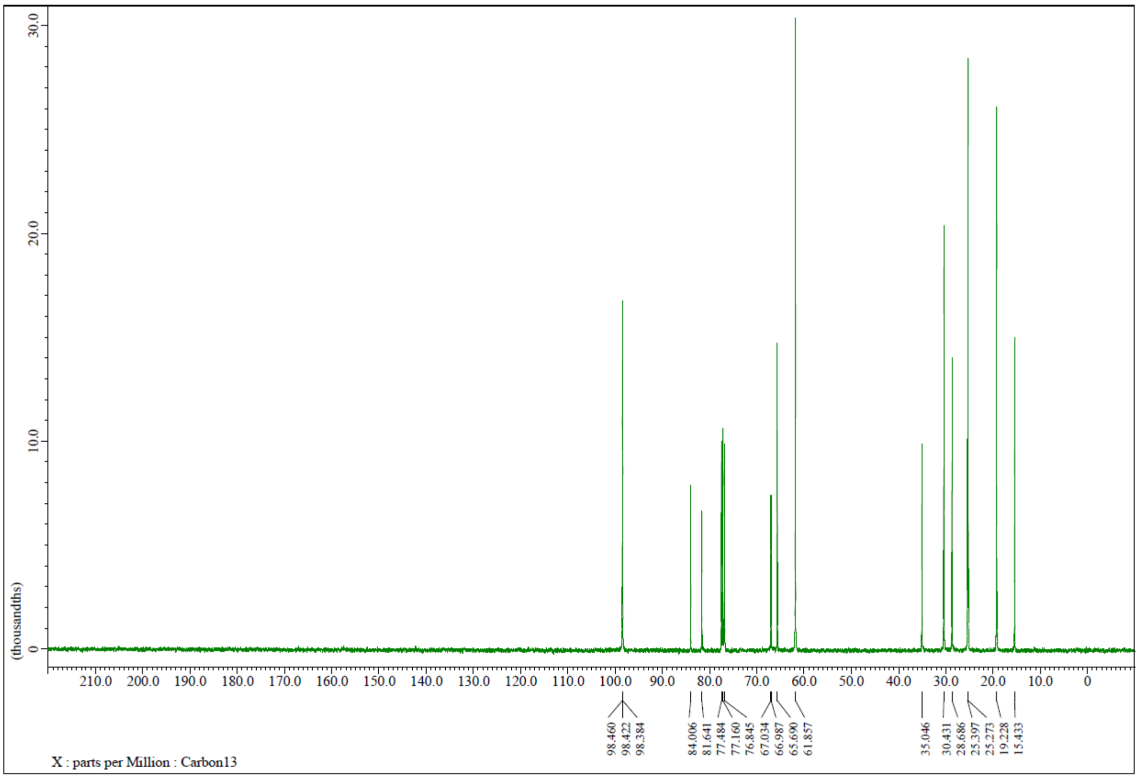

1,9-Bis((tetrahydro-2*H*-pyran-2-yl)oxy)nona-4,5-diene (S9a)

(^{1}H NMR, 400 MHz, $CDCl_3$)

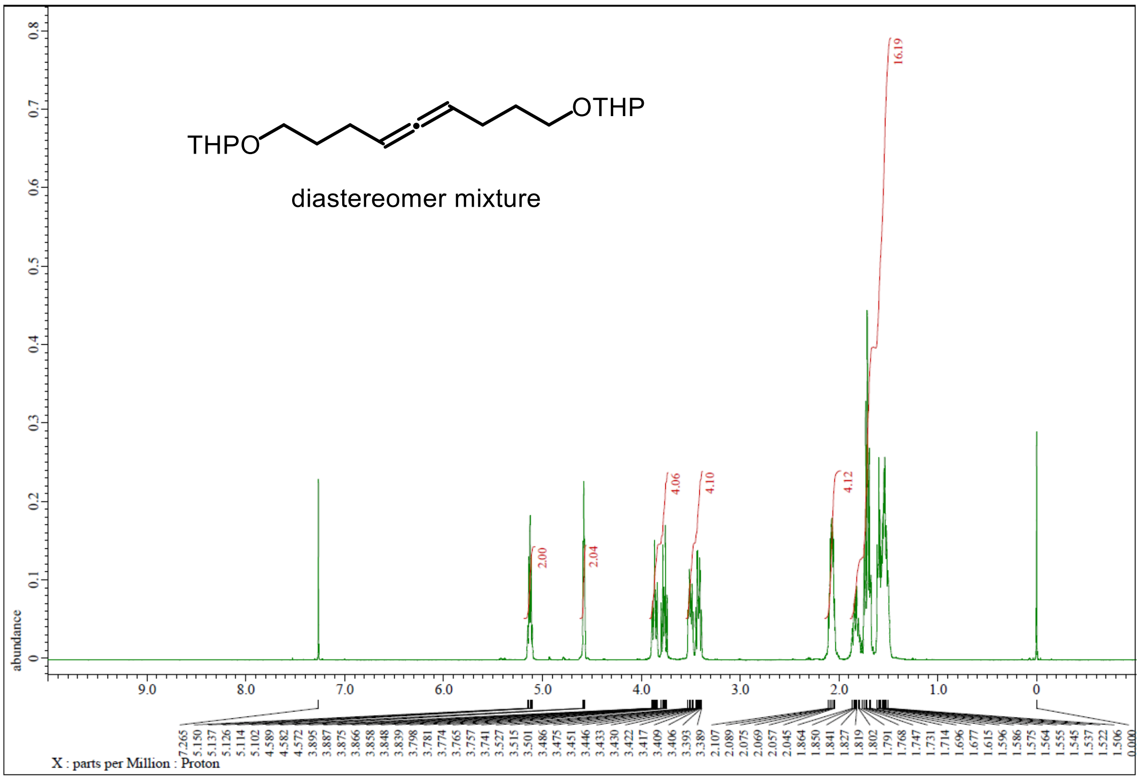

(^{13}C NMR, 100 MHz, $CDCl_3$)

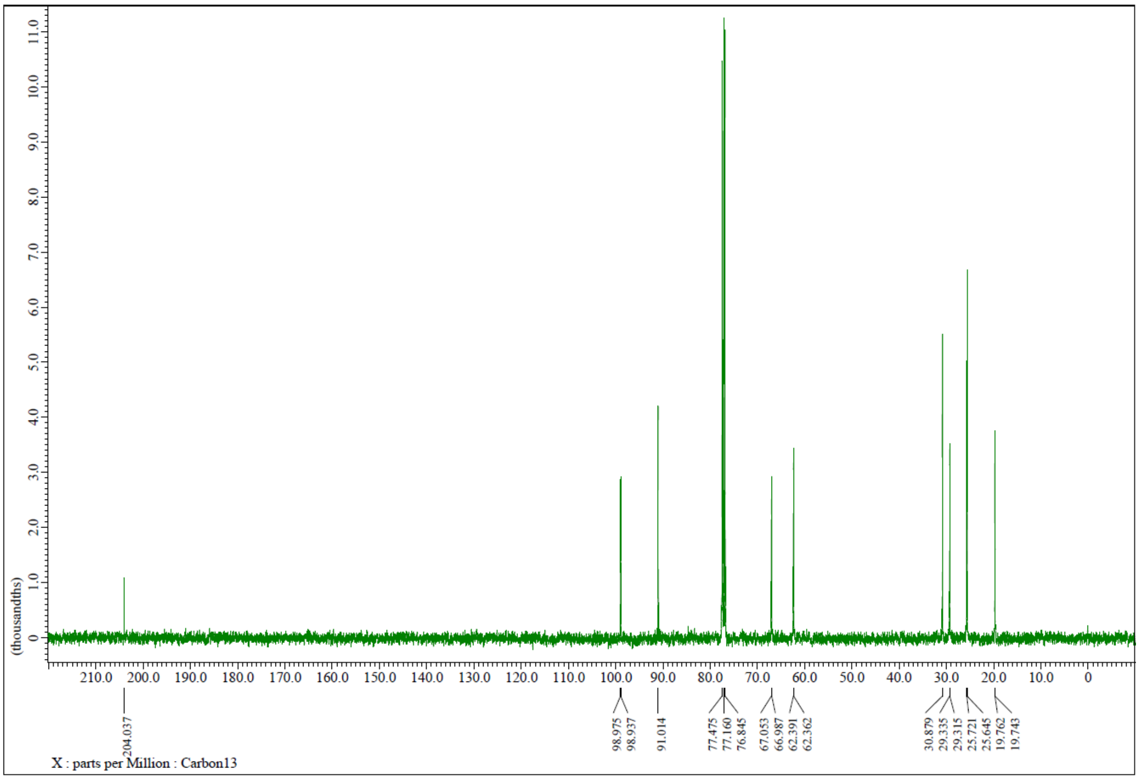

Nona-4,5-diene-1,9-diol (S10a)

(^{1}H NMR, 400 MHz, CDCl$_3$)

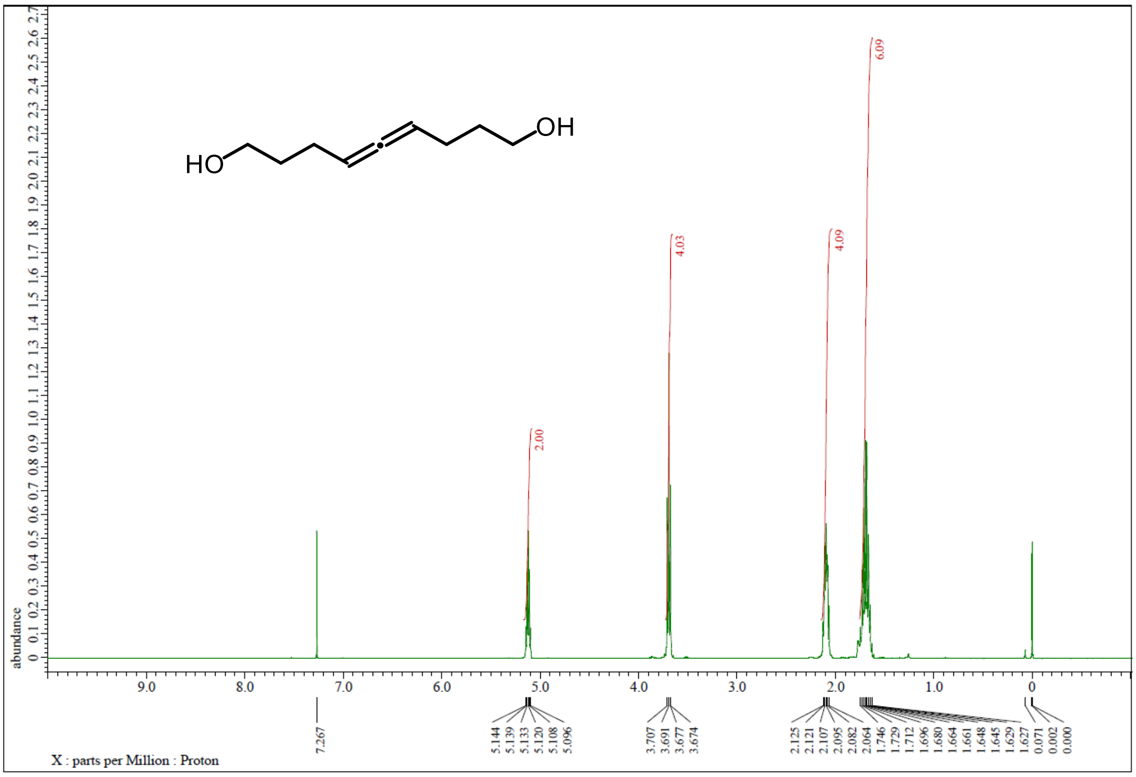

(^{13}C NMR, 100 MHz, CDCl$_3$)

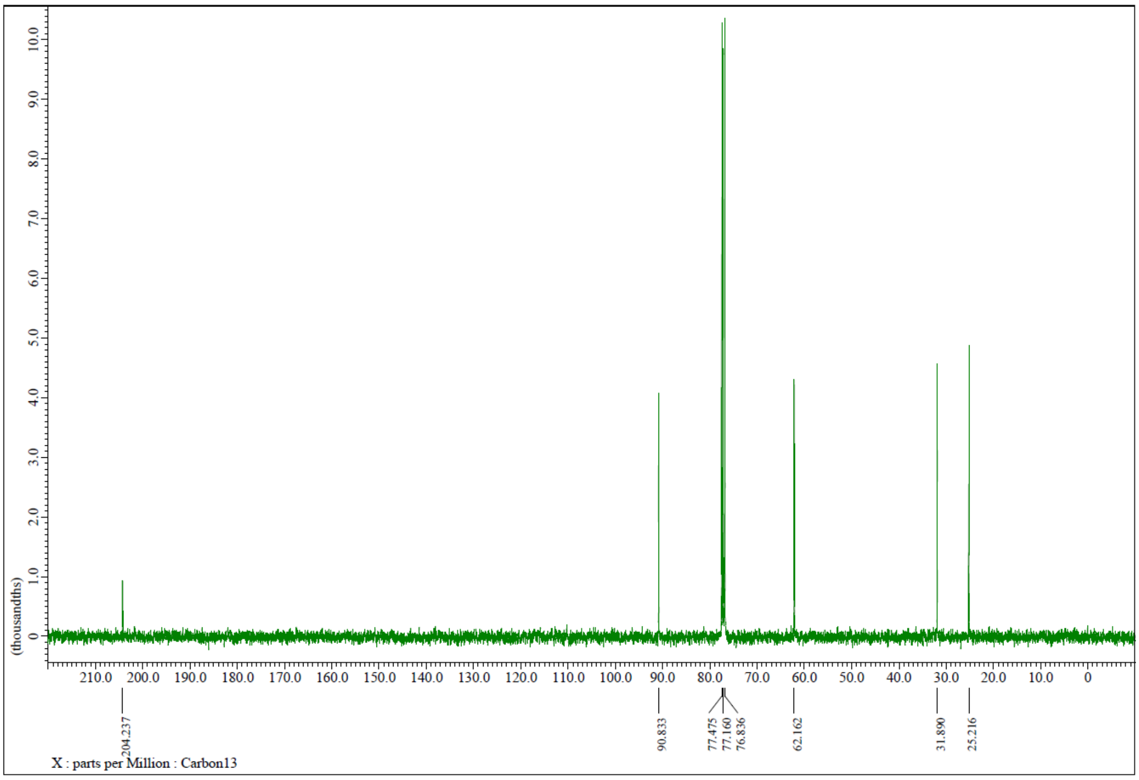

Nona-4,5-dienedial (1a)

(^{1}H NMR, 400 MHz, $CDCl_3$)

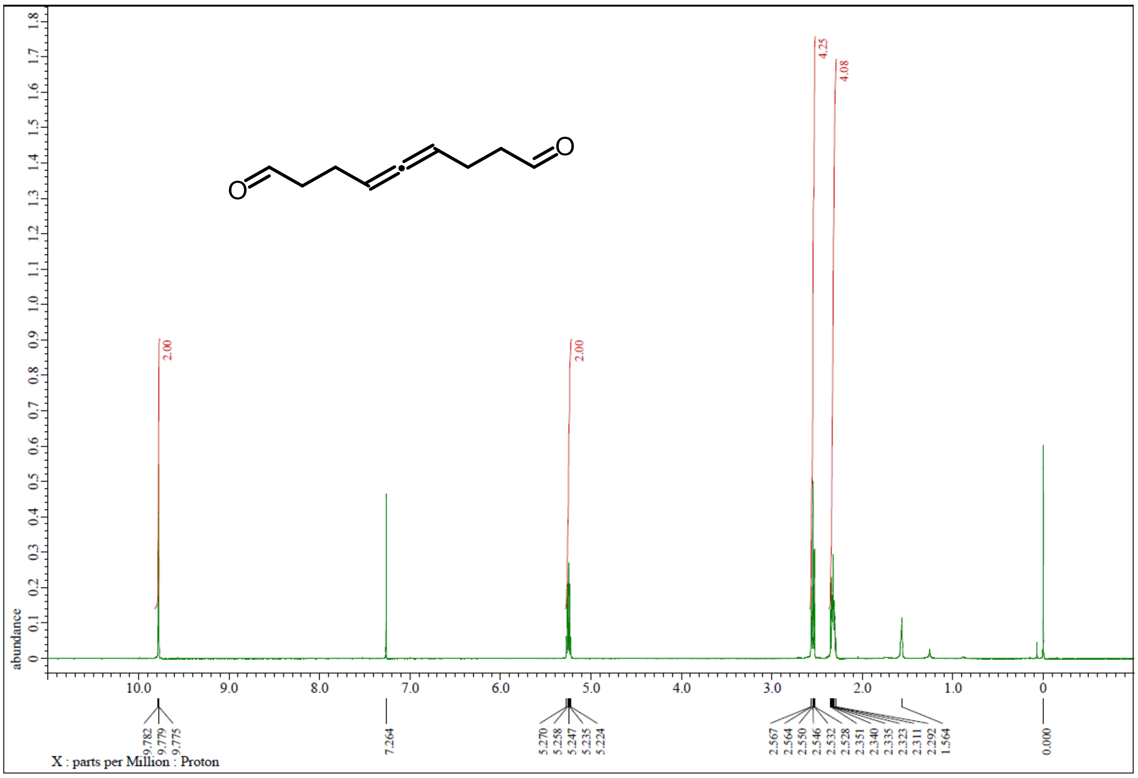

(^{13}C NMR, 100 MHz, $CDCl_3$)

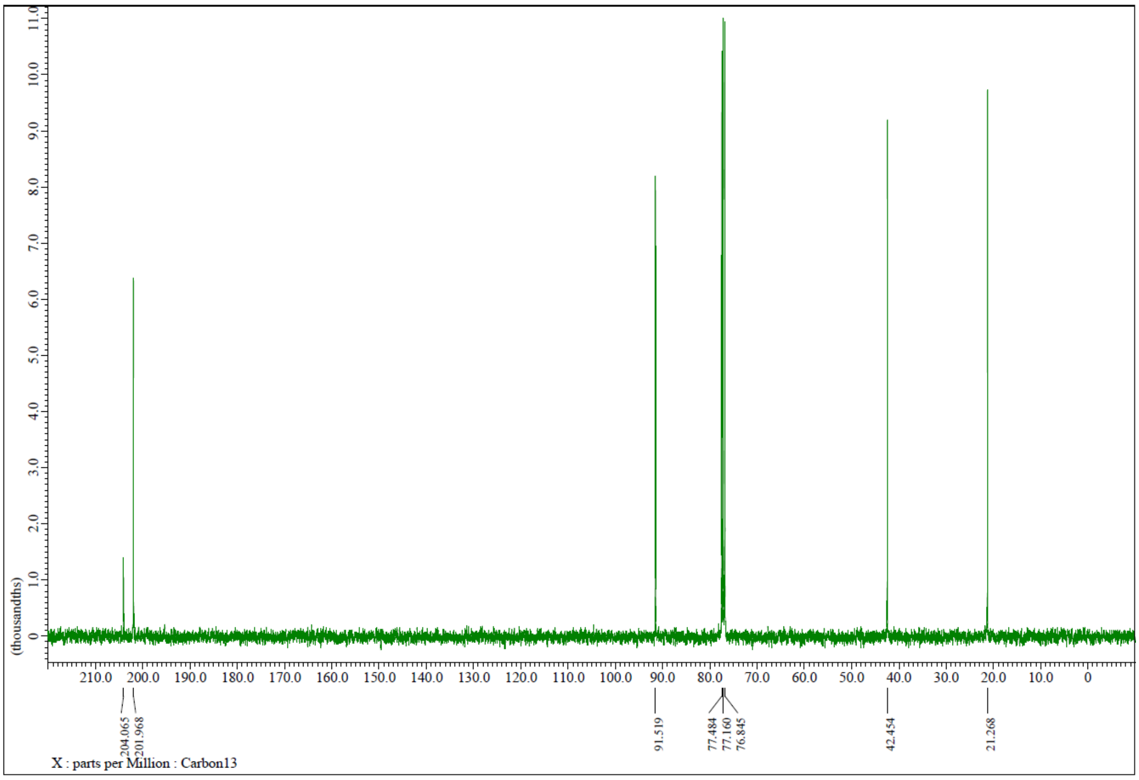

Nona-4,5-dienedial bisoxime (4a)

(^{1}H NMR, 400 MHz, $CDCl_3$)

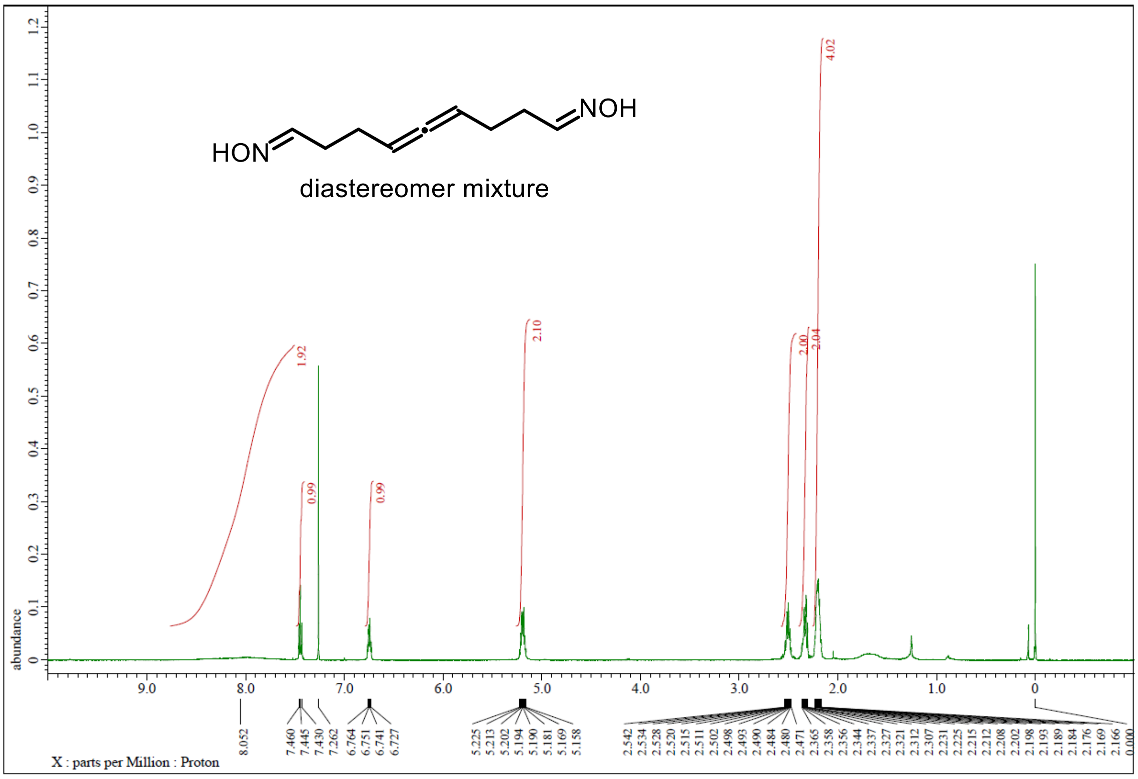

(^{13}C NMR, 100 MHz, $CDCl_3$)

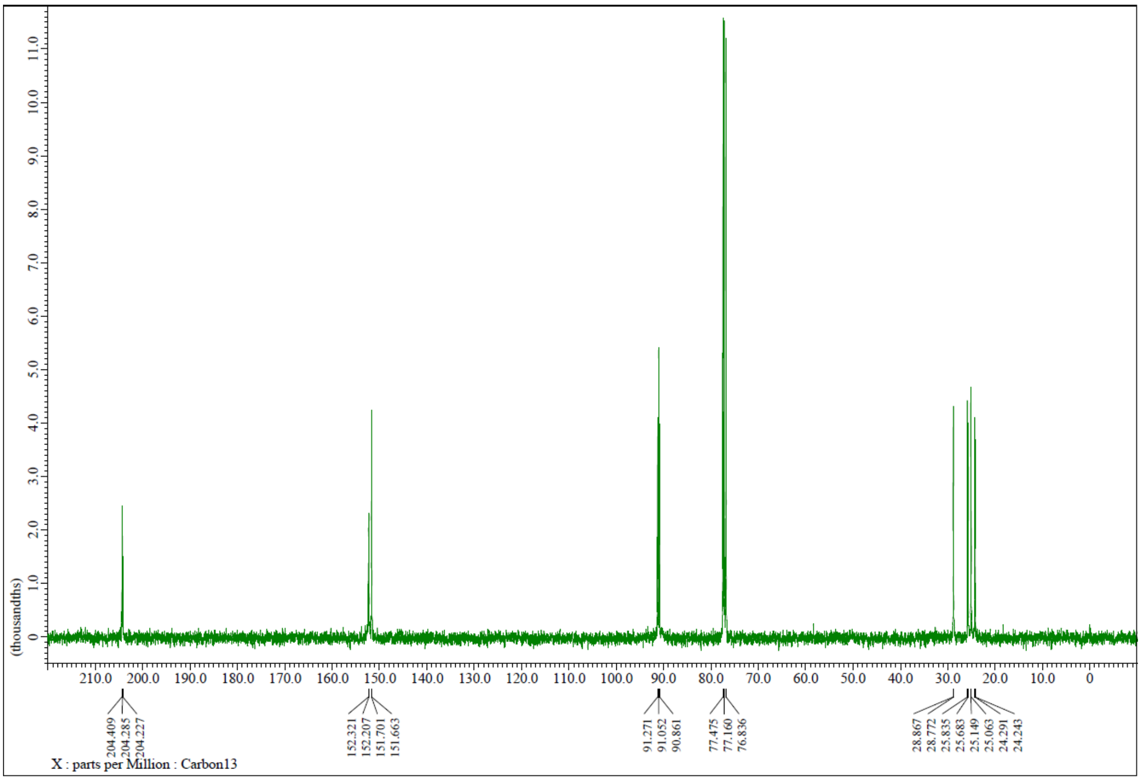

1-((Tetrahydro-2*H*-pyran-2-yl)oxy)-8-(((tetrahydro-2*H*-pyran-2-yl)oxy)methyl)tetradec-5-yn-4-ol (S8b)

(^{1}H NMR, 400 MHz, CDCl$_3$)

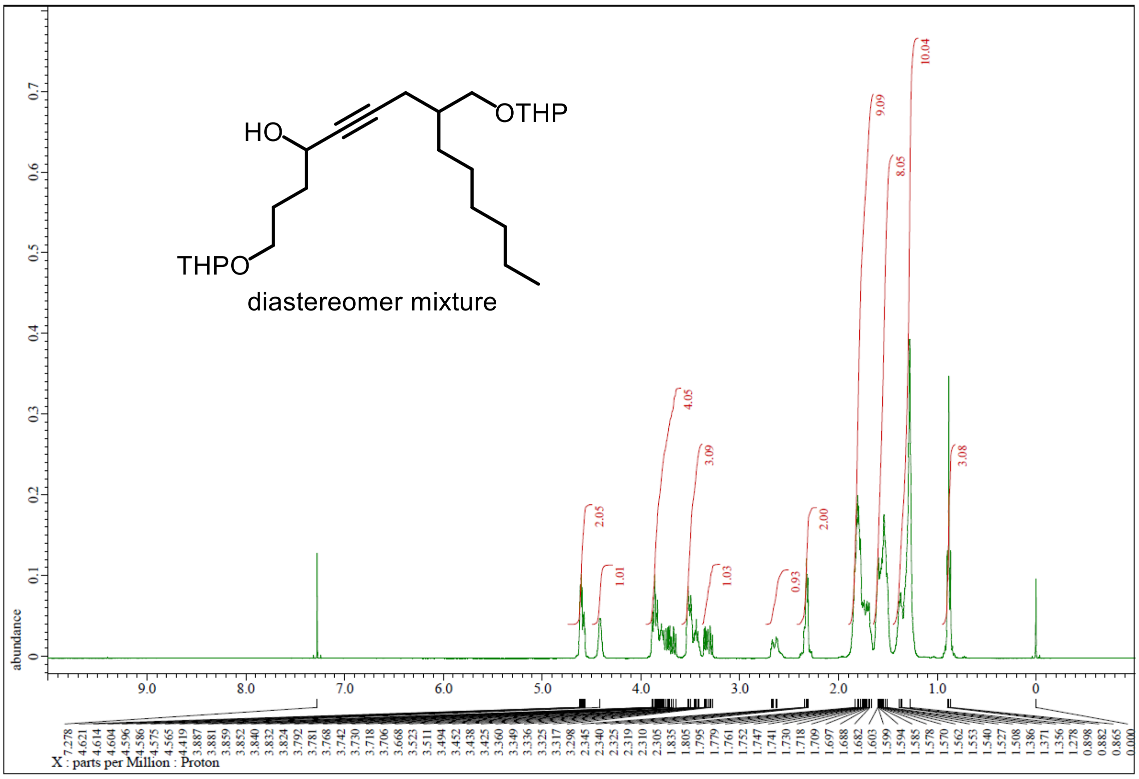

(^{13}C NMR, 100 MHz, CDCl$_3$)

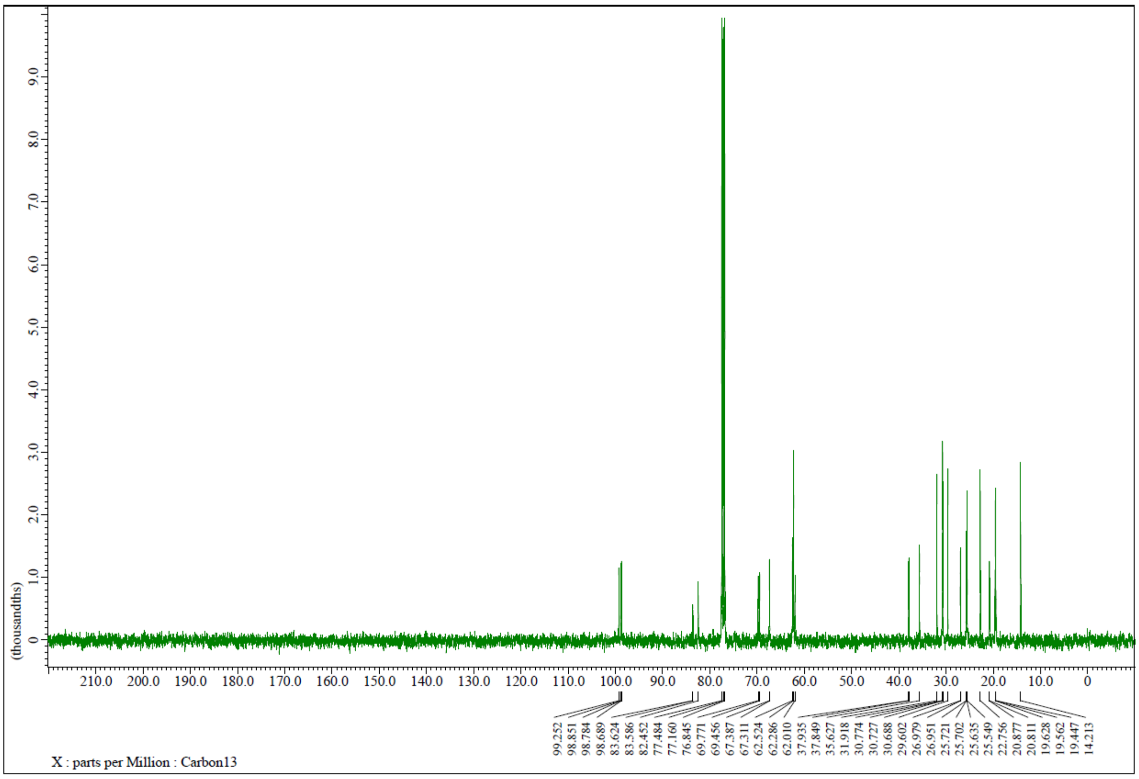

2,2'-((2-Hexylnona-4,5-diene-1,9-diyl)bis(oxy))bis(tetrahydro-2*H*-pyran) (S9b)

(^{1}H NMR, 400 MHz, $CDCl_3$)

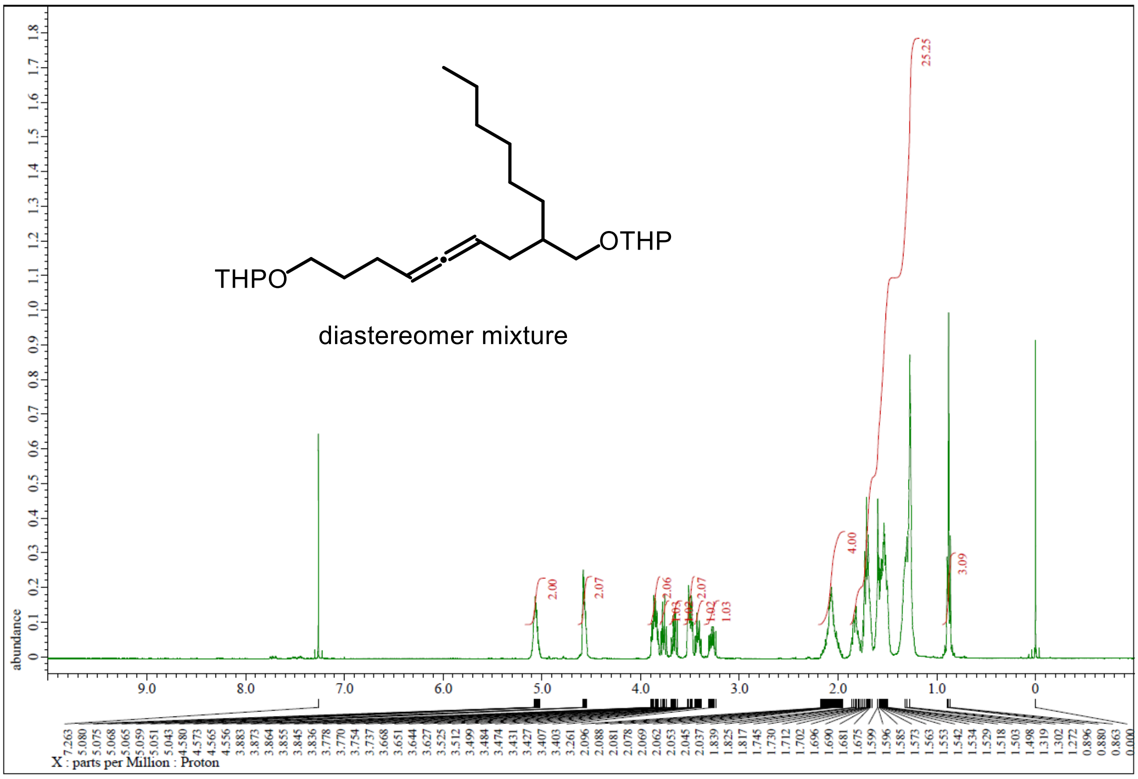

(^{13}C NMR, 100 MHz, $CDCl_3$)

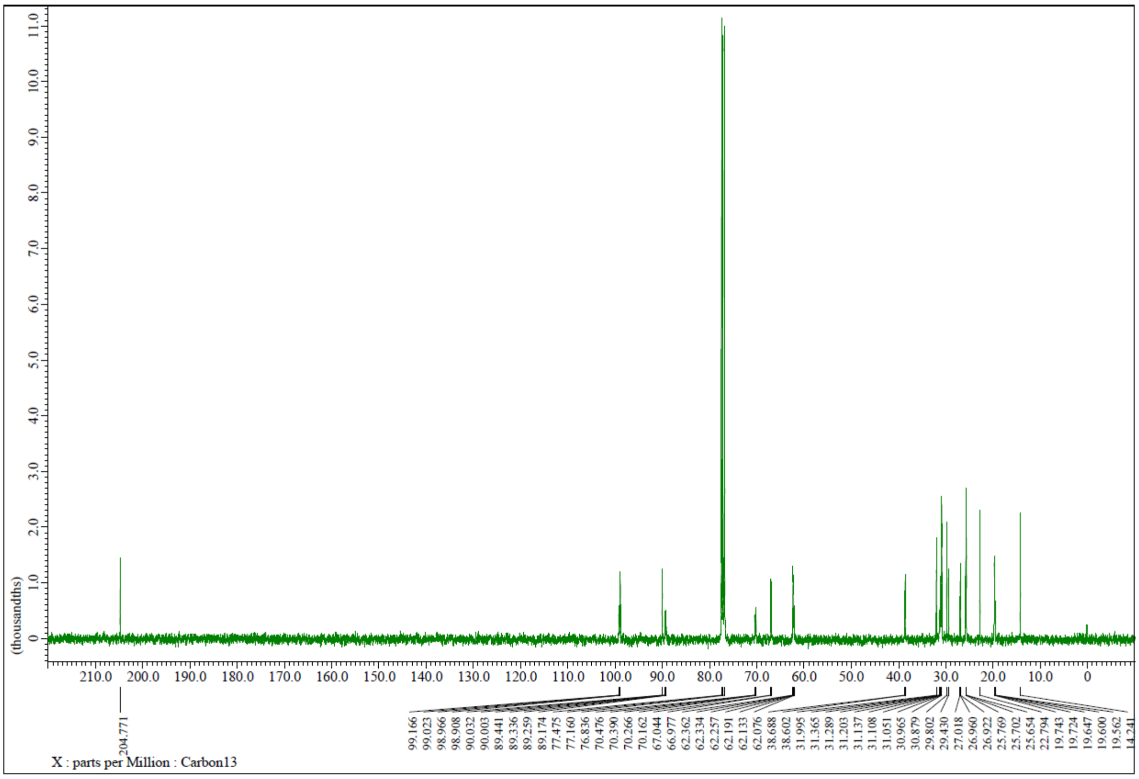

2-Hexylnona-4,5-diene-1,9-diol (S10b)

(^{1}H NMR, 400 MHz, $CDCl_3$)

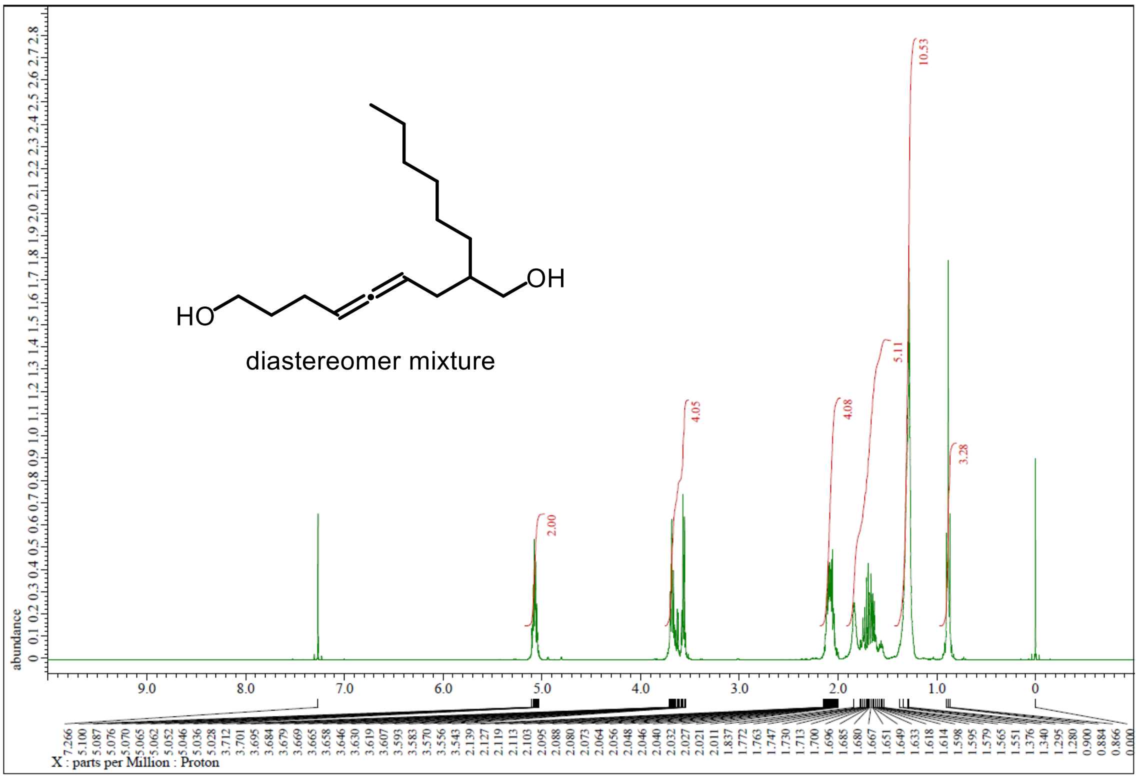

(^{13}C NMR, 100 MHz, $CDCl_3$)

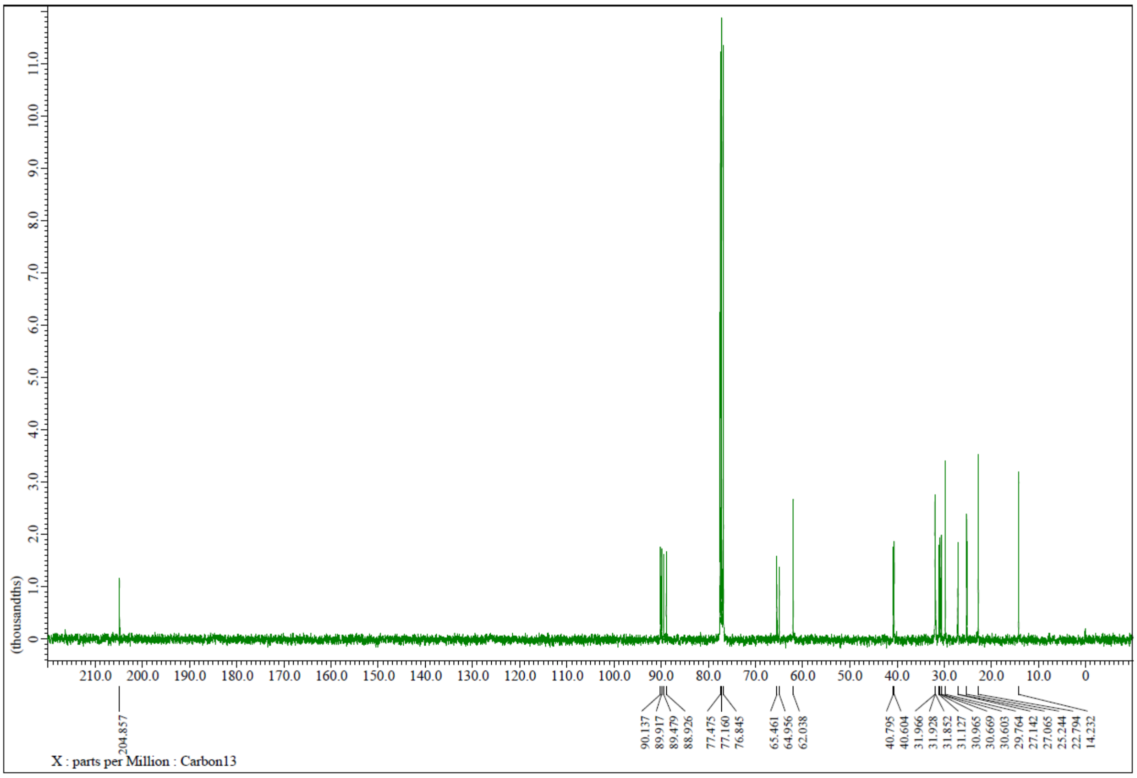

2-Hexylnona-4,5-dienedial (1b)

(^{1}H NMR, 400 MHz, $CDCl_3$)

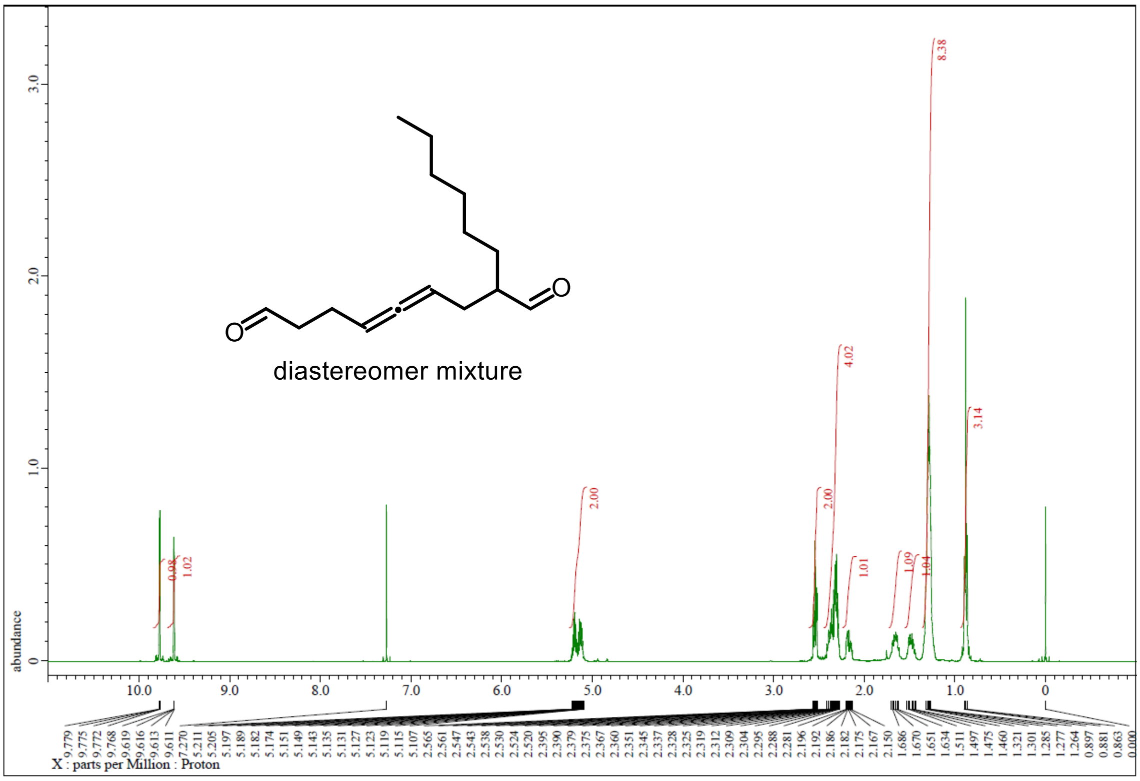

(^{13}C NMR, 100 MHz, $CDCl_3$)

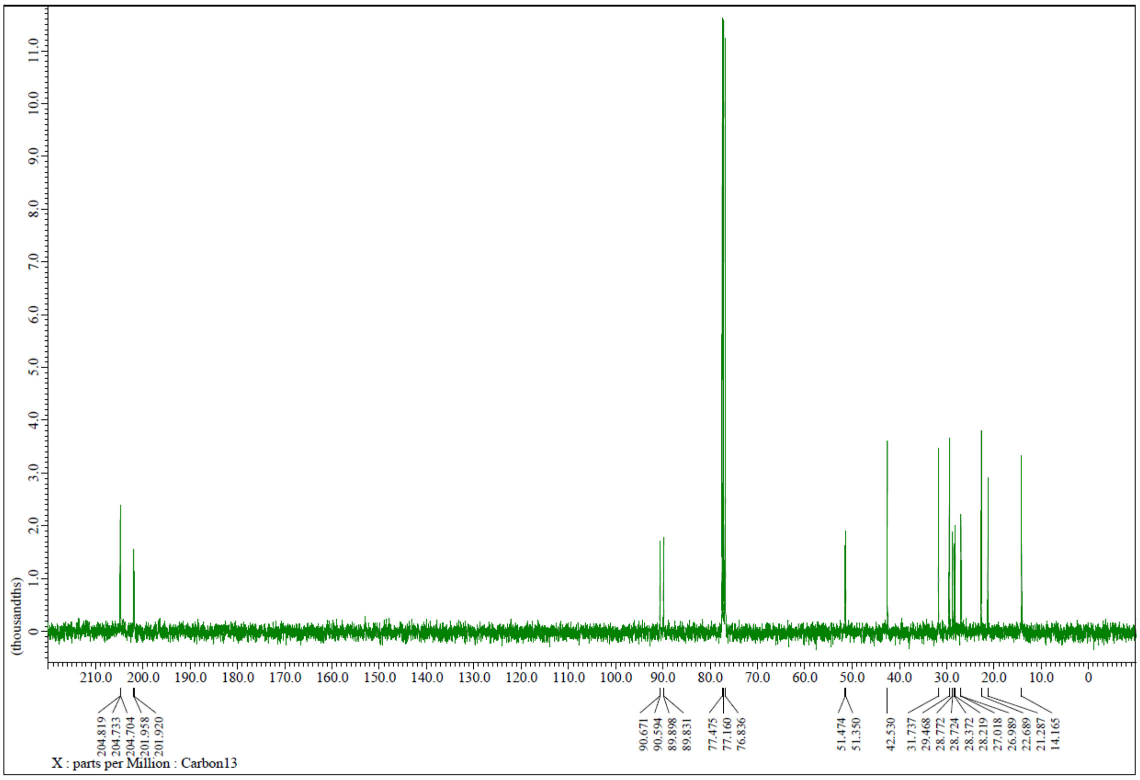

2-Hexylnona-4,5-dienedial bisoxime (4b)

(^{1}H NMR, 400 MHz, $CDCl_3$)

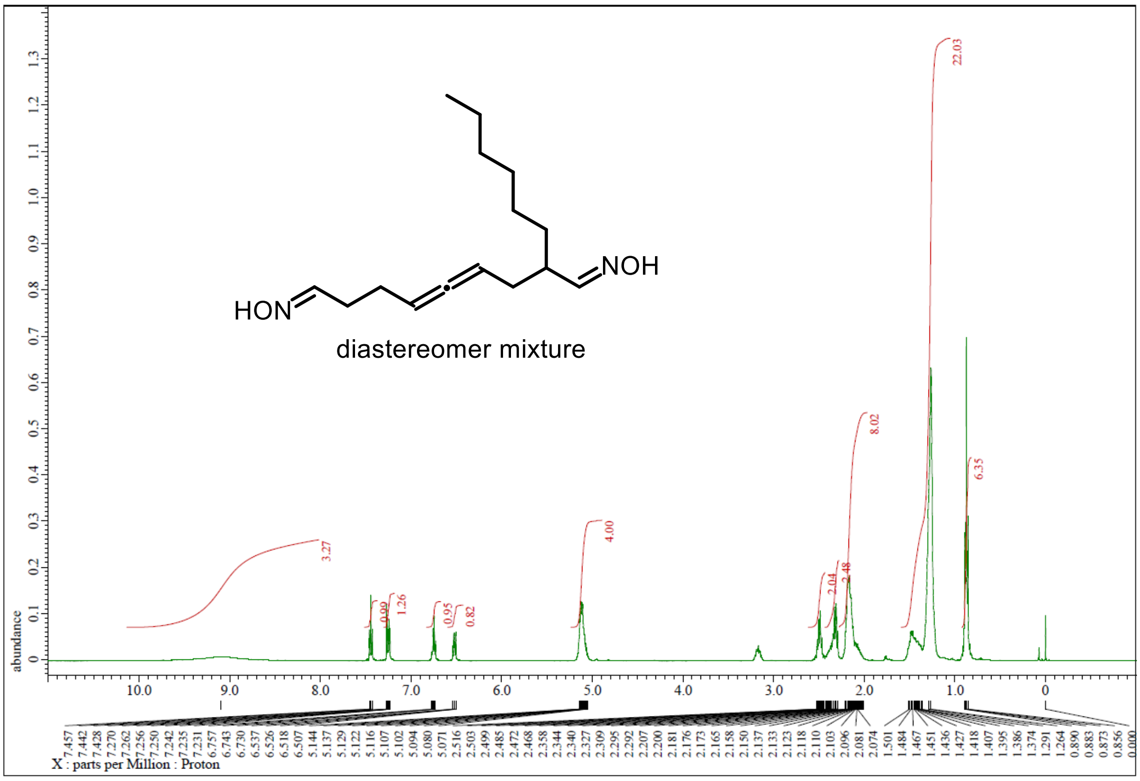

(^{13}C NMR, 100 MHz, $CDCl_3$)

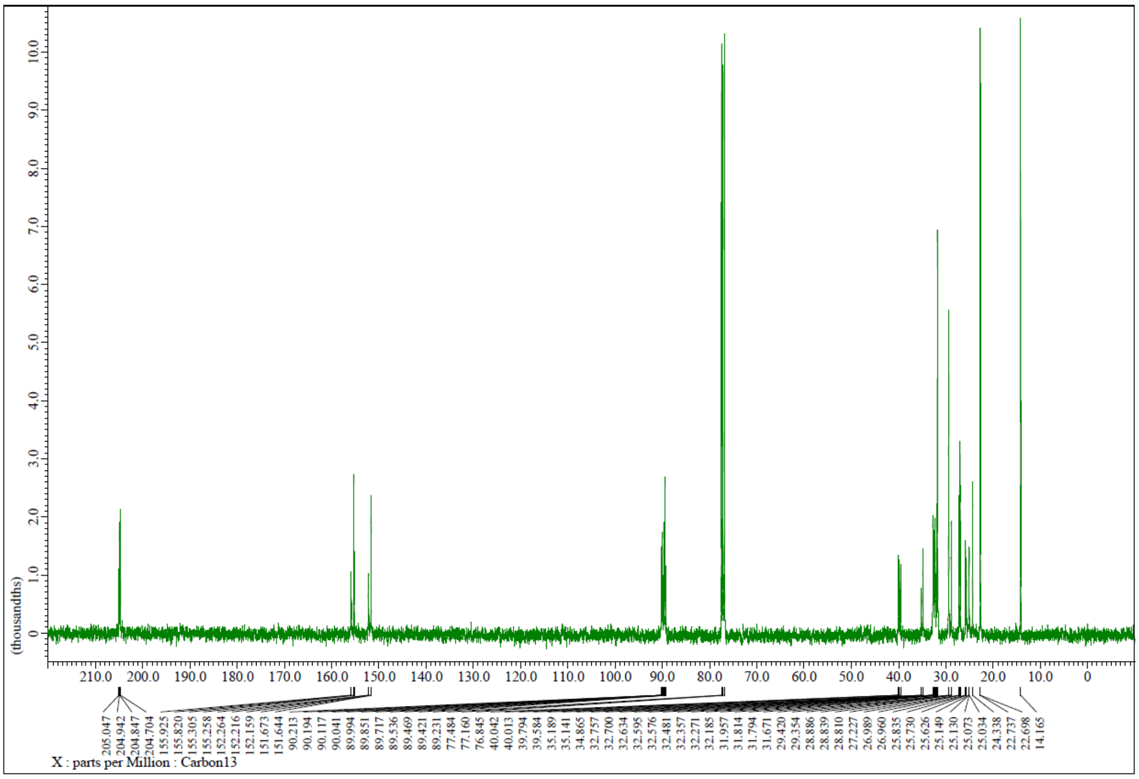

8-(Benzyloxy)-1,9-bis((tetrahydro-2*H*-pyran-2-yl)oxy)non-5-yn-4-ol (S8c)

(^{1}H NMR, 400 MHz, $CDCl_3$)

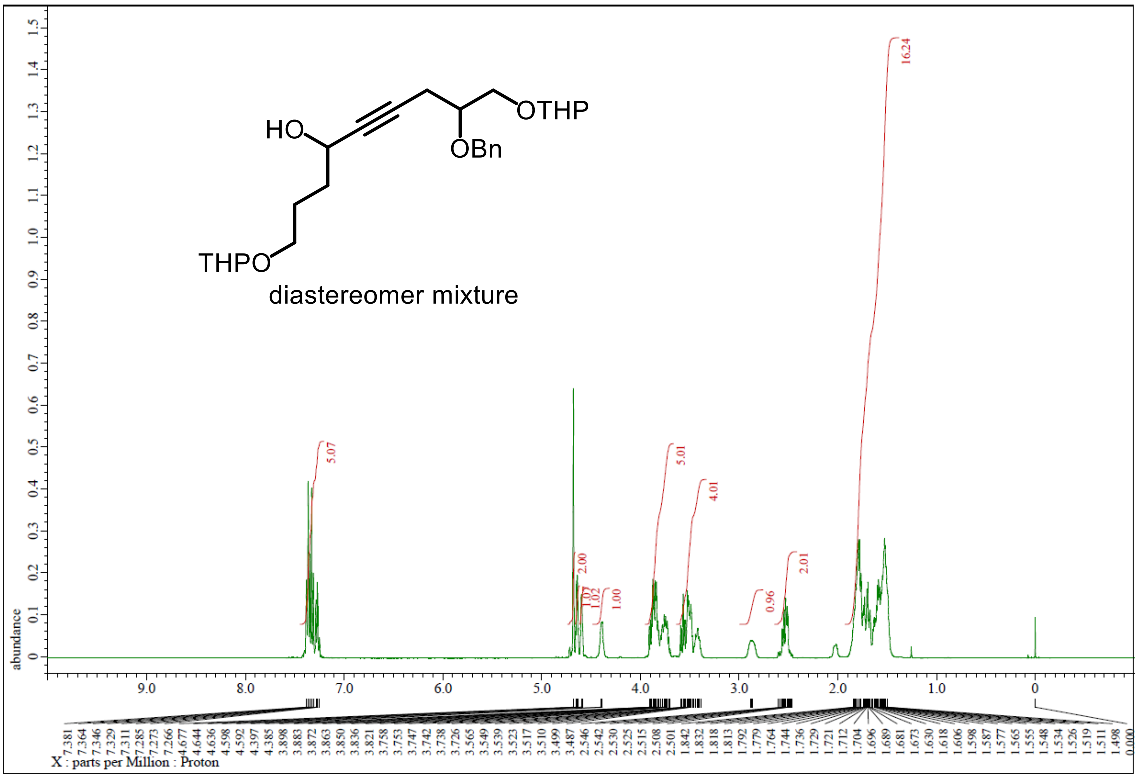

(^{13}C NMR, 100 MHz, $CDCl_3$)

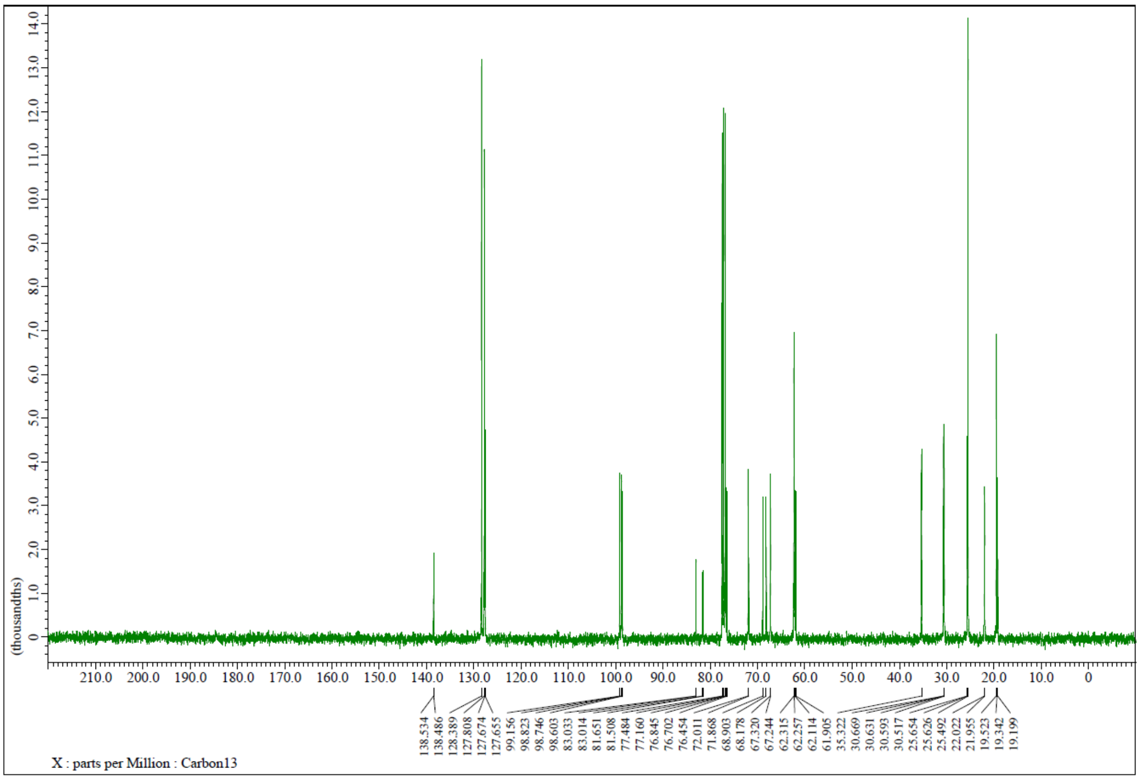

2,2'-((2-(Benzyloxy)nona-4,5-diene-1,9-diyl)bis(oxy))bis(tetrahydro-2*H*-pyran) (S9c)

(^{1}H NMR, 400 MHz, CDCl$_3$)

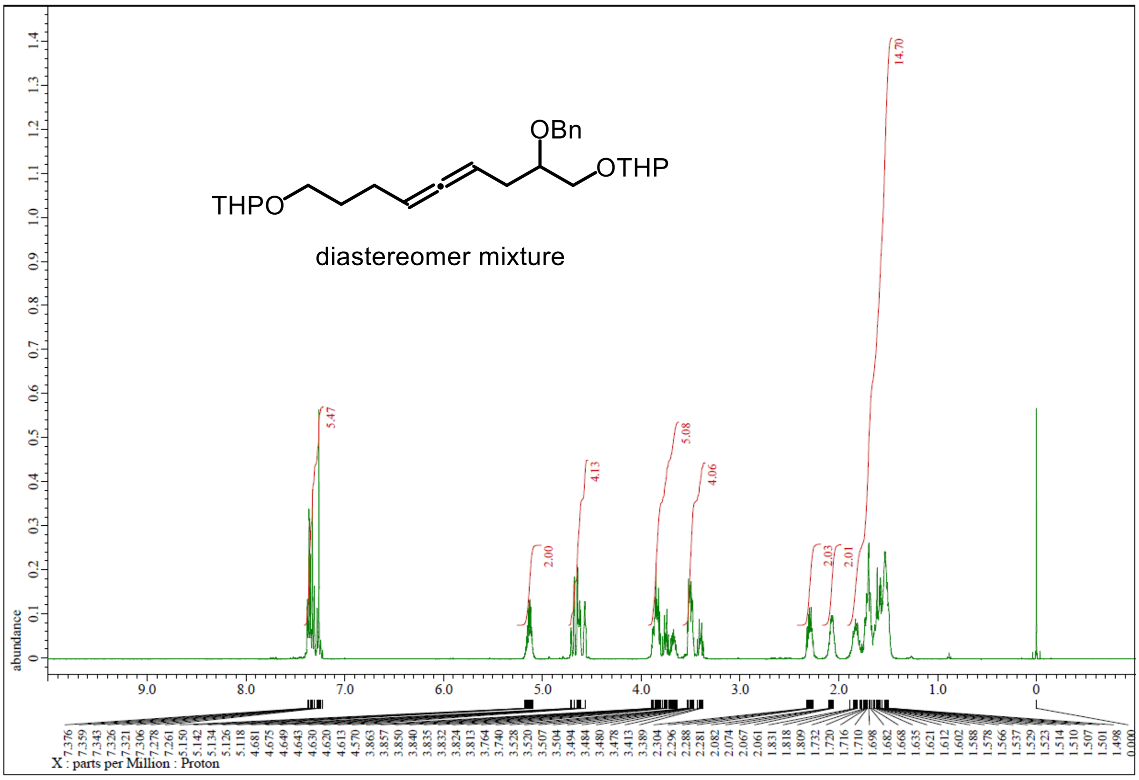

(^{13}C NMR, 100 MHz, CDCl$_3$)

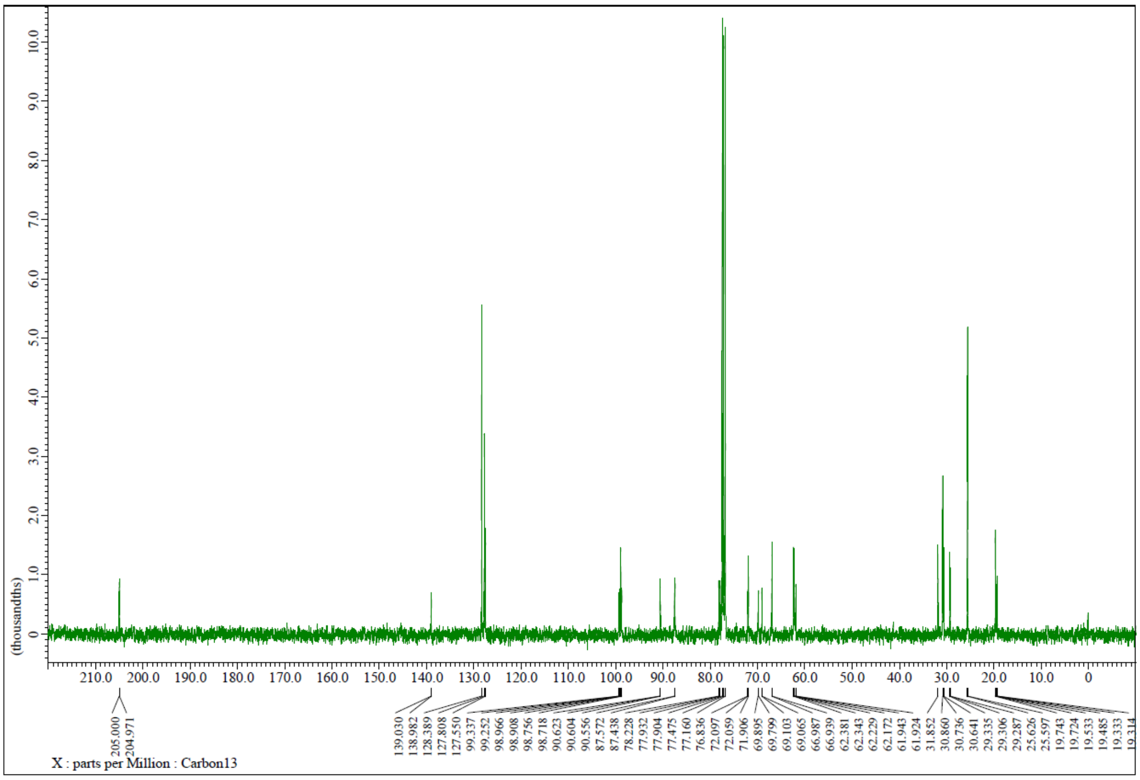

2-(Benzyloxy)nona-4,5-diene-1,9-diol (S10c)

(^{1}H NMR, 400 MHz, $CDCl_3$)

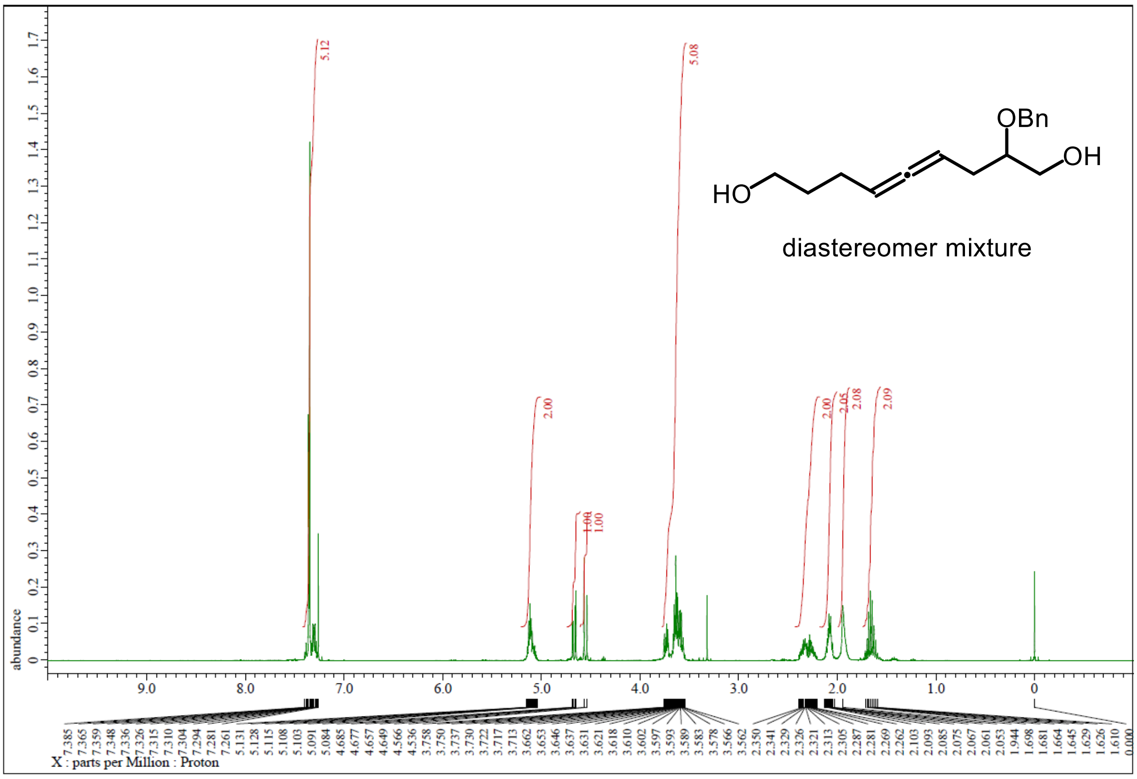

(^{13}C NMR, 100 MHz, $CDCl_3$)

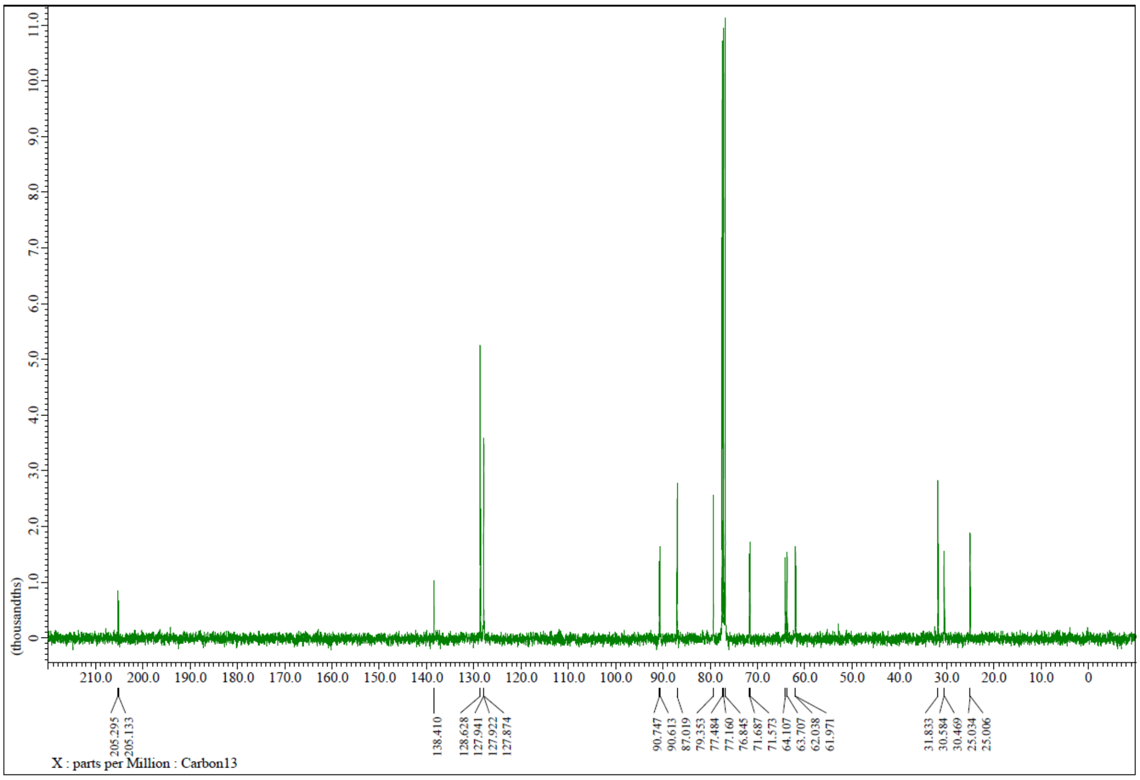

2-(Benzyloxy)nona-4,5-dienedial (1c)

(^{1}H NMR, 400 MHz, $CDCl_3$)

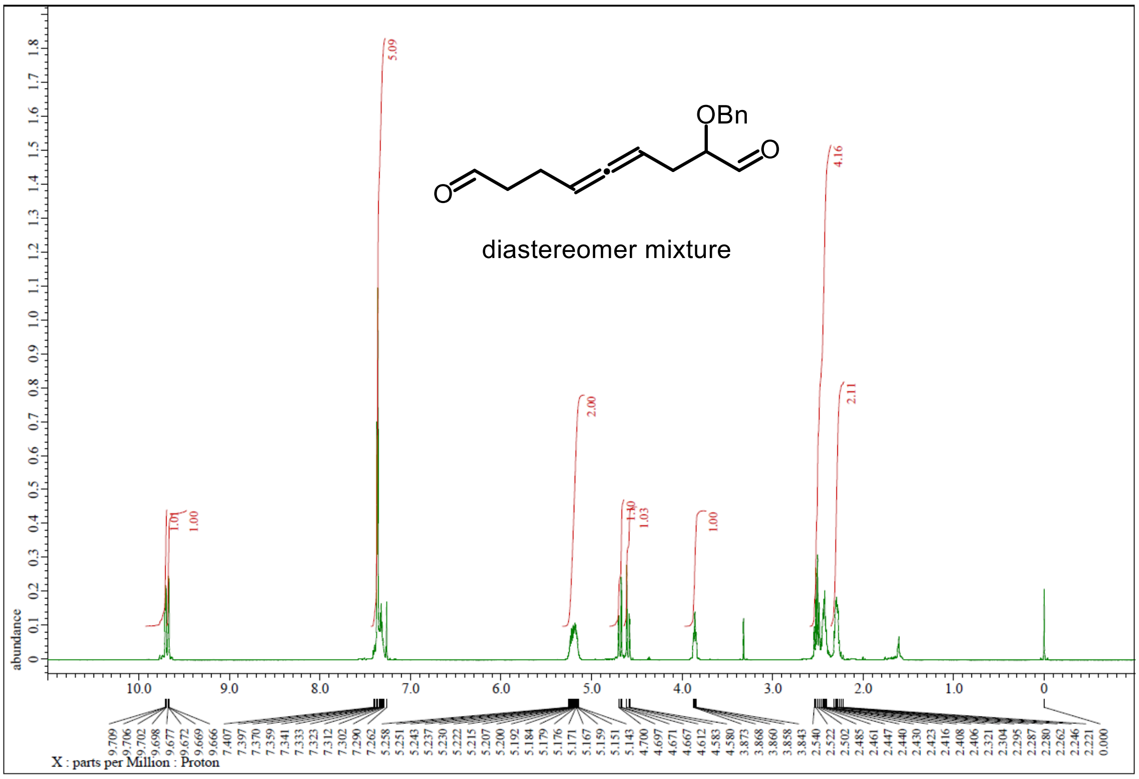

(^{13}C NMR, 100 MHz, $CDCl_3$)

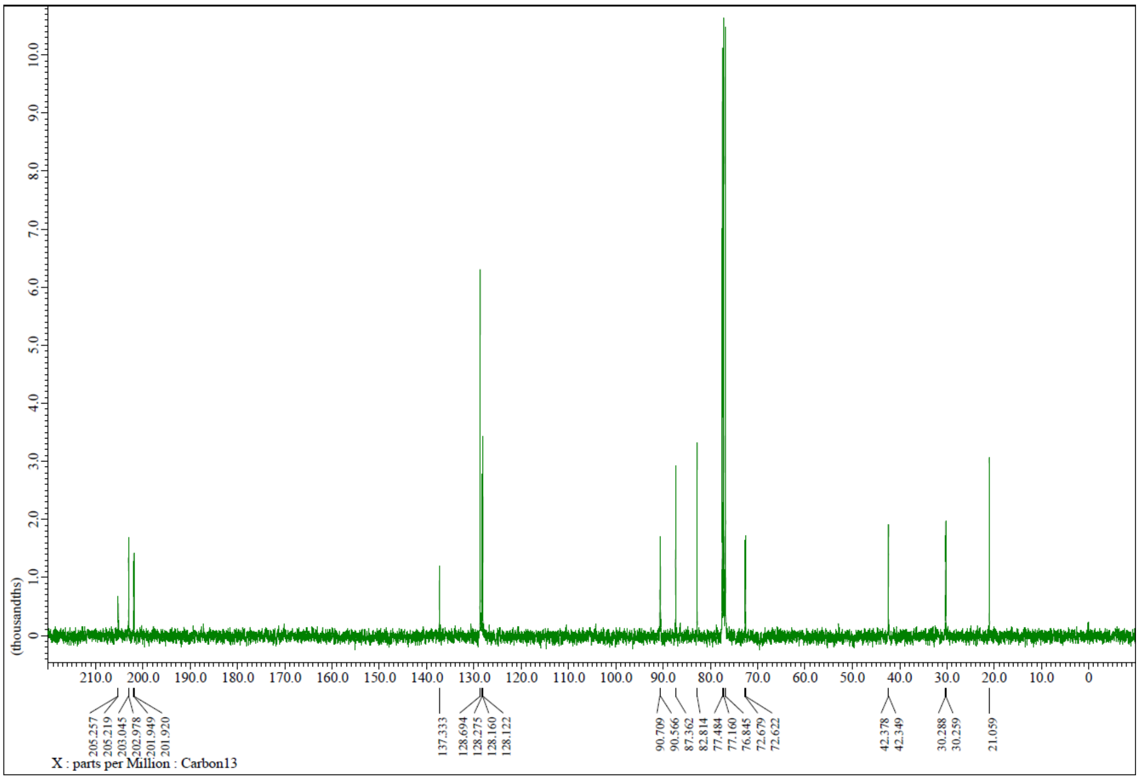

2-(Benzyloxy)nona-4,5-dienedial bisoxime (4c)

(^{1}H NMR, 400 MHz, $CDCl_3$)

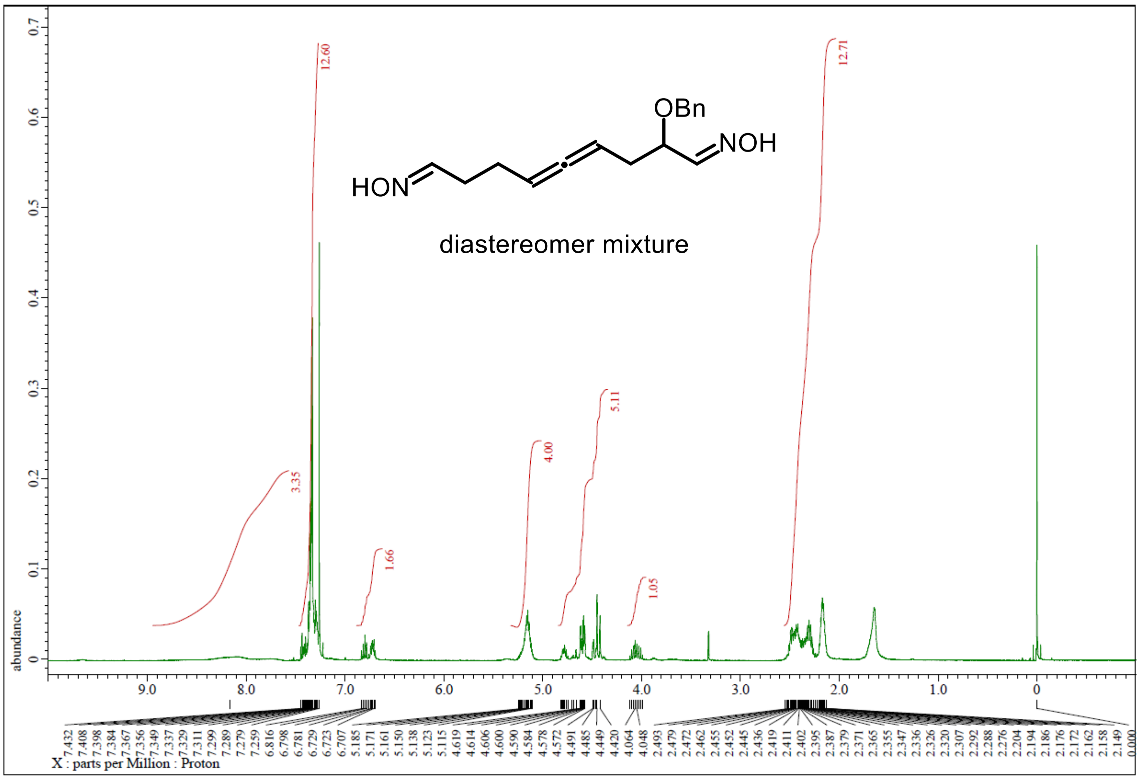

(^{13}C NMR, 100 MHz, $CDCl_3$)

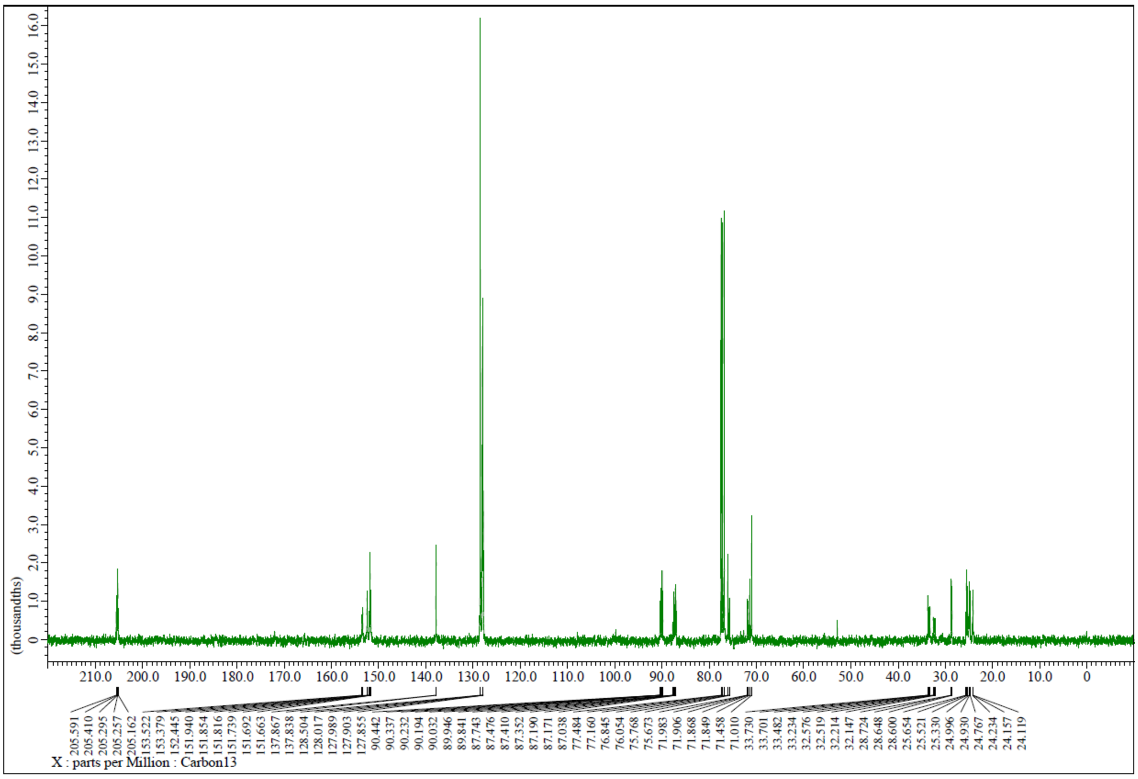

1,10-Bis((tetrahydro-2*H*-pyran-2-yl)oxy)dec-5-yn-4-ol (S8d)

(^{1}H NMR, 400 MHz, $CDCl_3$)

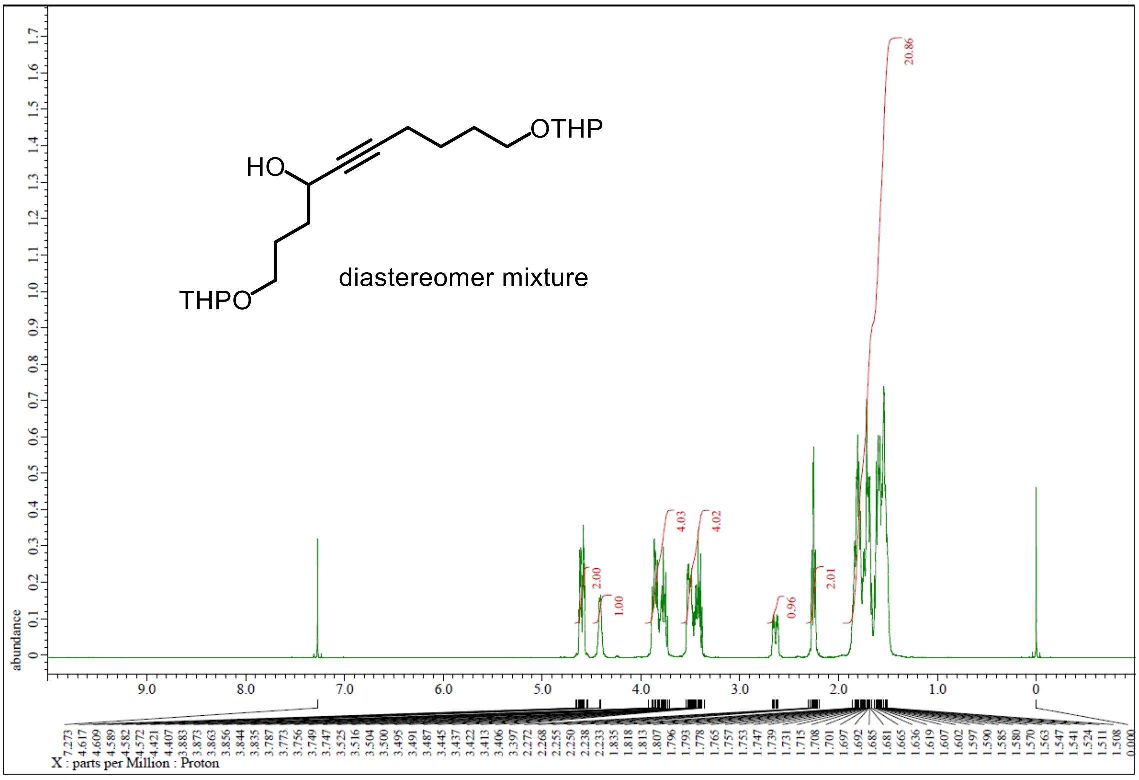

(^{13}C NMR, 100 MHz, $CDCl_3$)

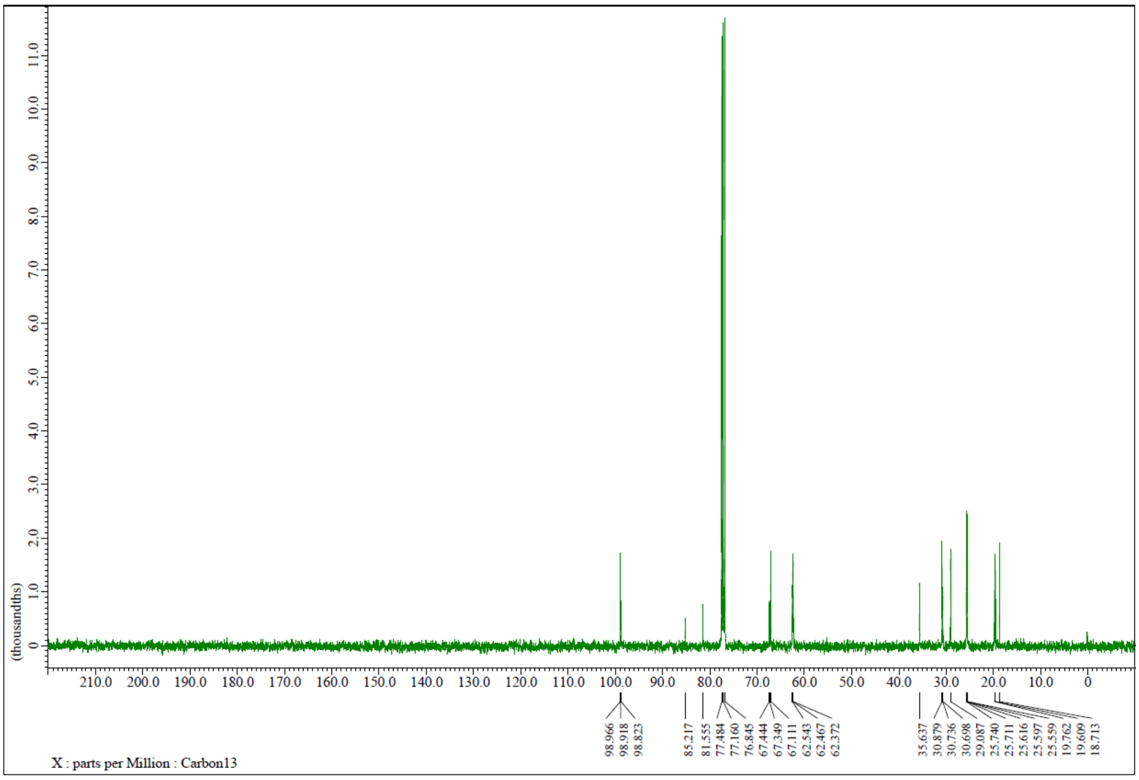

2,2'-(Deca-4,5-diene-1,10-diylbis(oxy))bis(tetrahydro-2*H*-pyran) (S9d)

(^{1}H NMR, 400 MHz, $CDCl_3$)

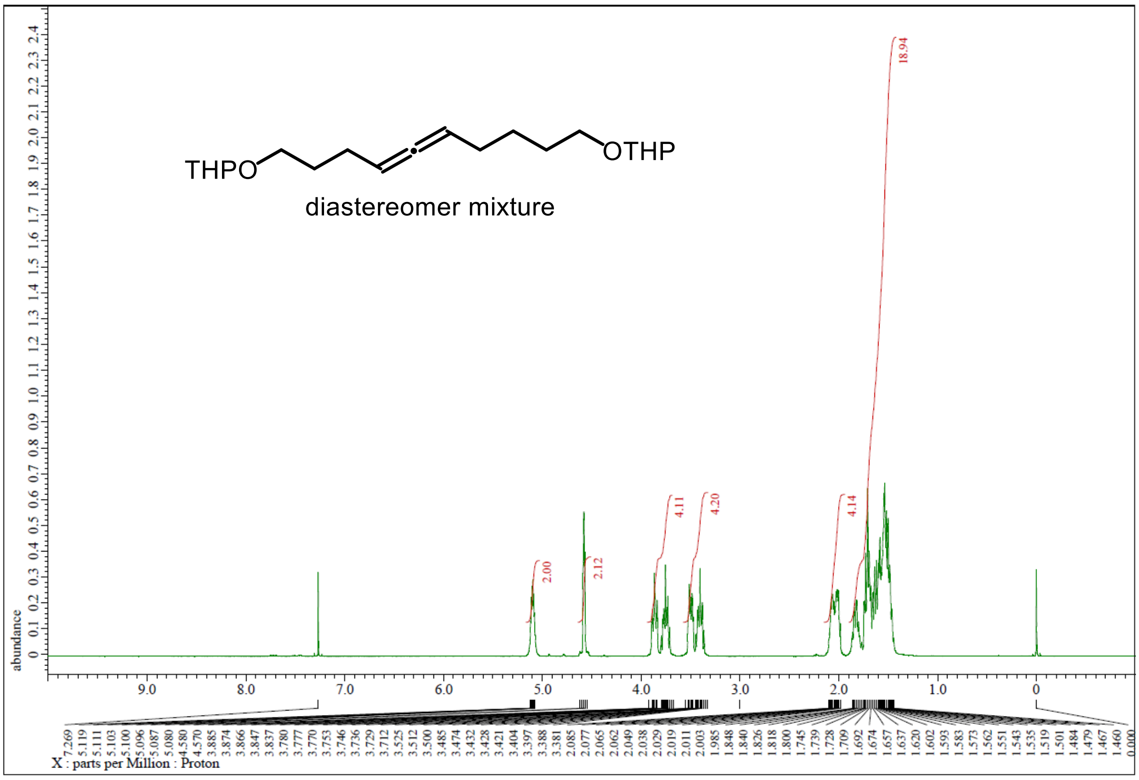

(^{13}C NMR, 100 MHz, $CDCl_3$)

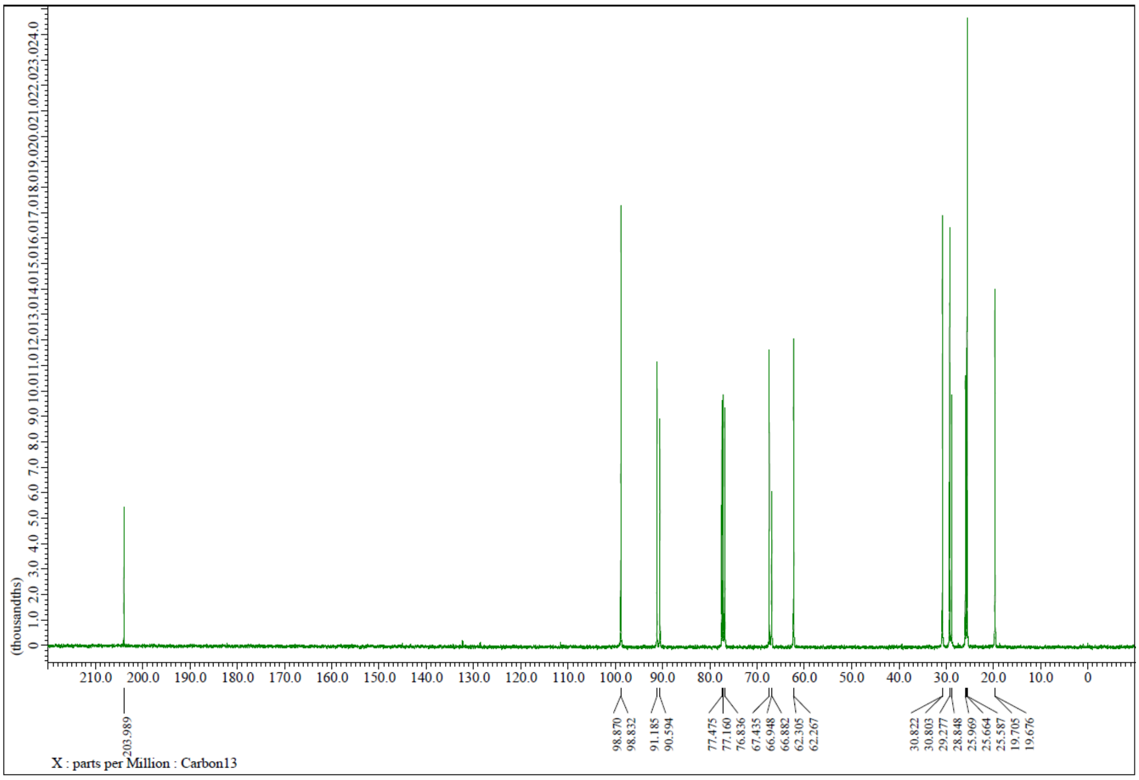

Deca-4,5-diene-1,10-diol (S10d)

(^{1}H NMR, 400 MHz, $CDCl_3$)

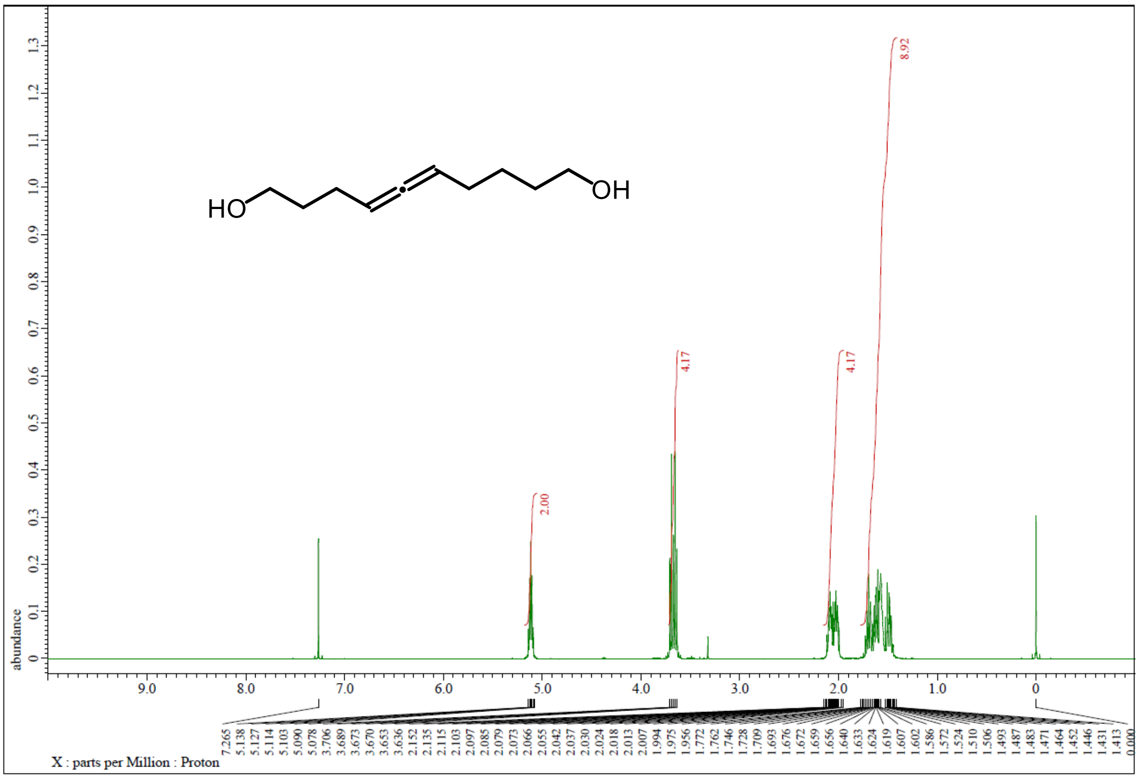

(^{13}C NMR, 100 MHz, $CDCl_3$)

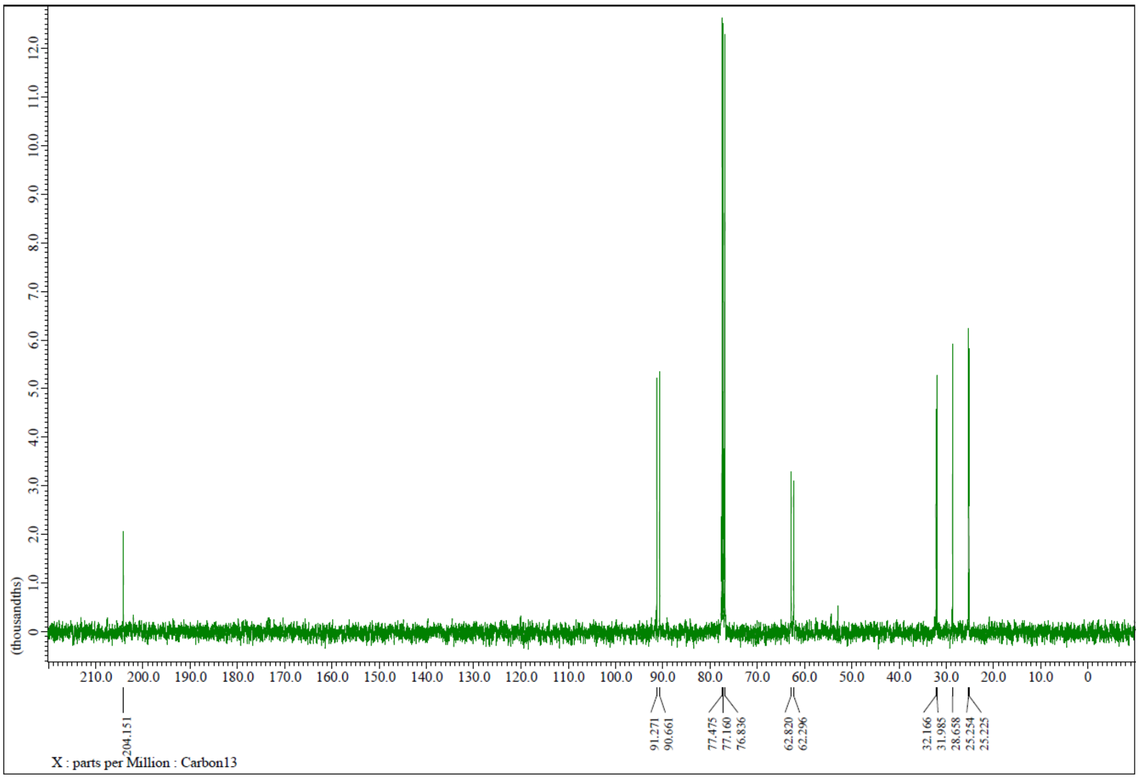

Deca-4,5-dienedial (1d)

(^{1}H NMR, 400 MHz, $CDCl_3$)

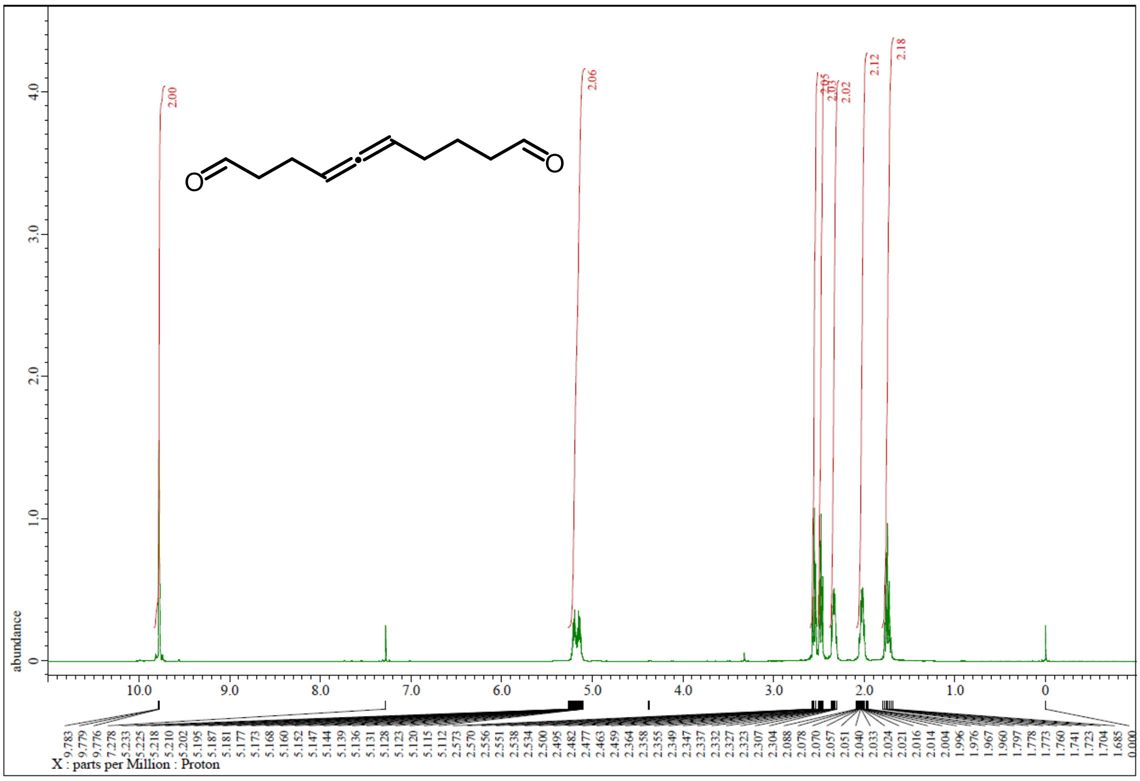

(^{13}C NMR, 100 MHz, $CDCl_3$)

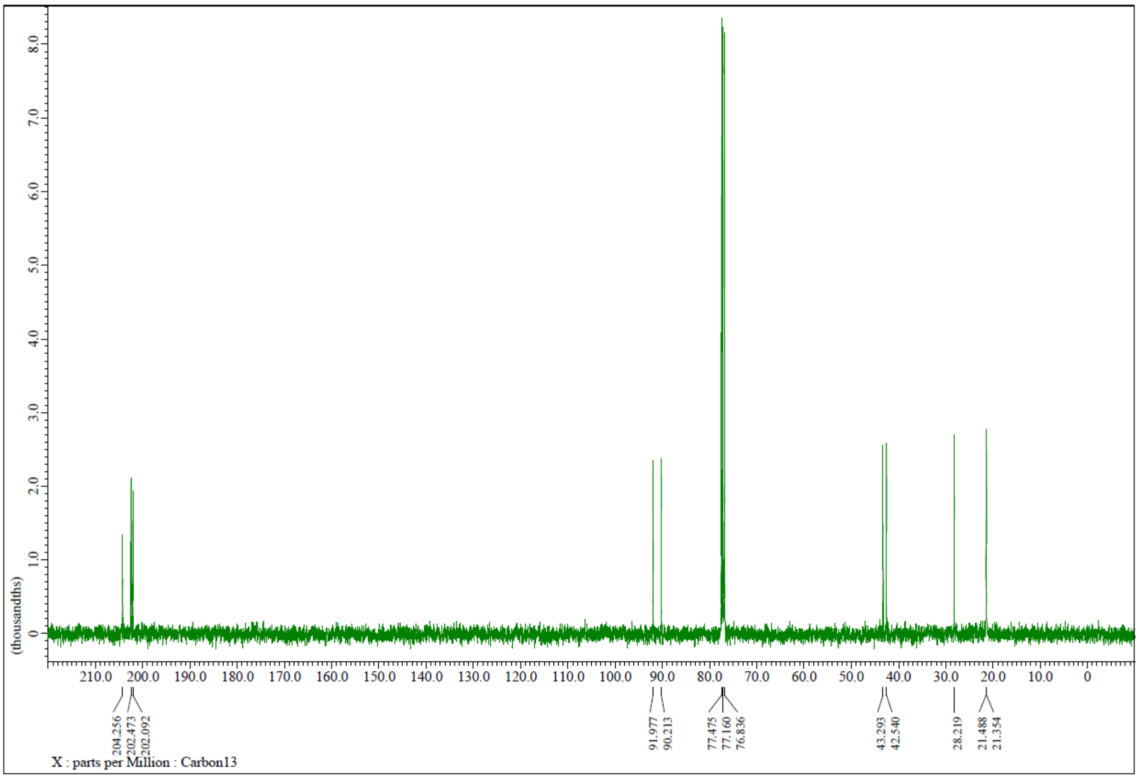

Deca-4,5-dienedial bisoxime (4d)

(^{1}H NMR, 400 MHz, $CDCl_3$)

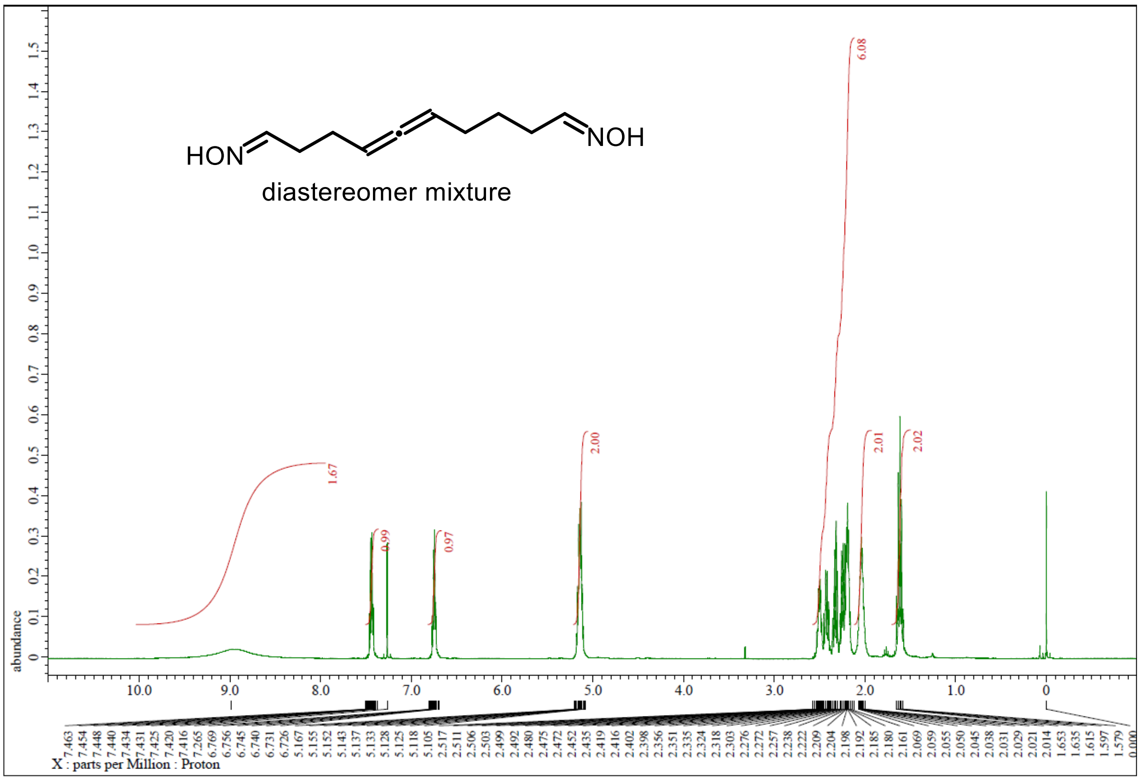

(^{13}C NMR, 100 MHz, $CDCl_3$)

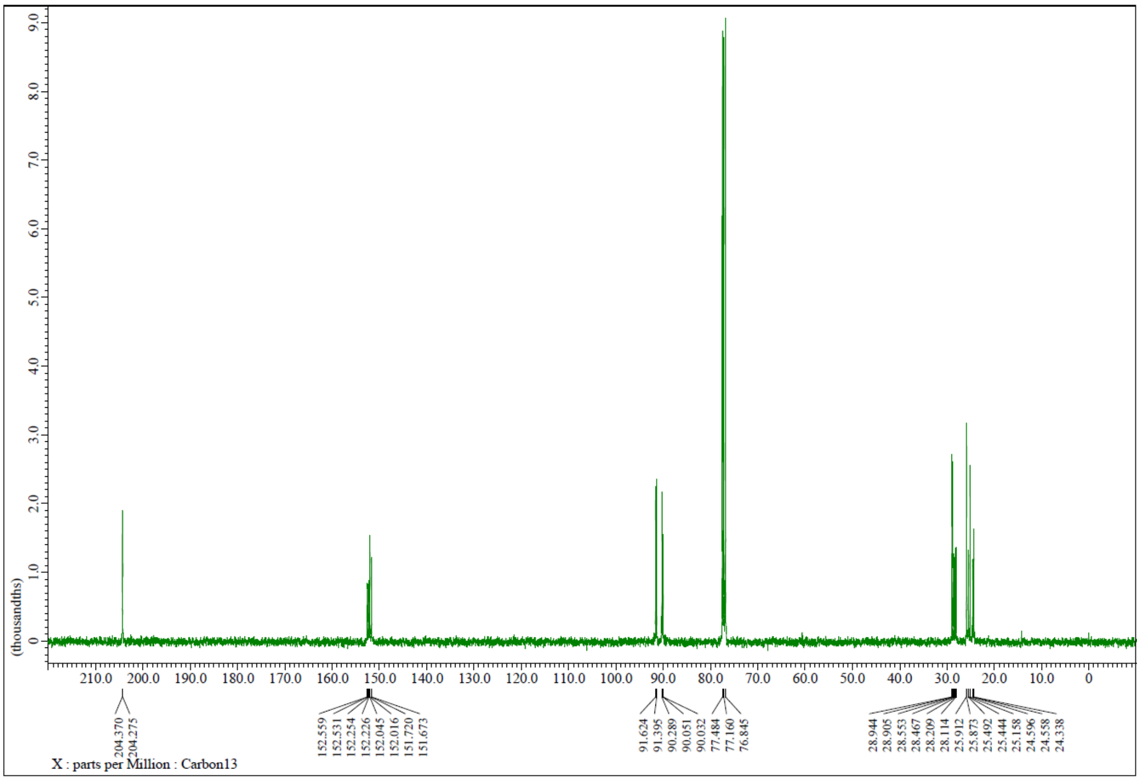

1,11-Bis((tetrahydro-2*H*-pyran-2-yl)oxy)undec-6-yn-5-ol (S8e)

(^{1}H NMR, 400 MHz, $CDCl_3$)

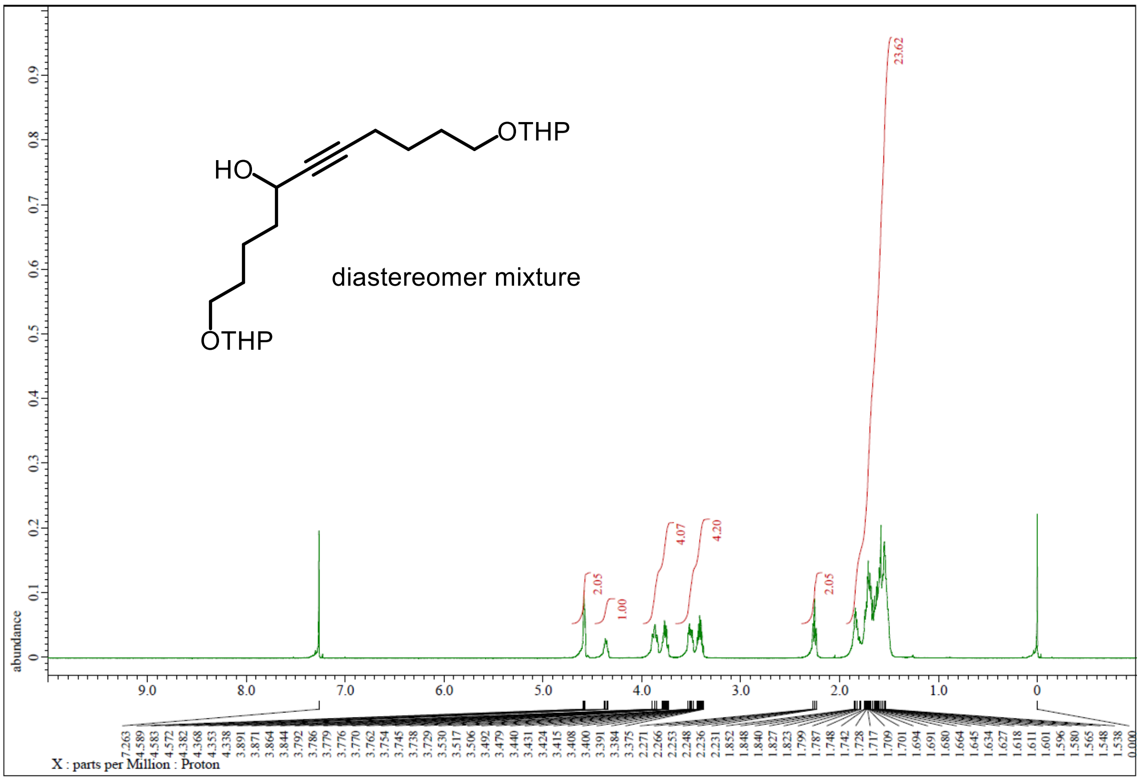

(^{13}C NMR, 100 MHz, $CDCl_3$)

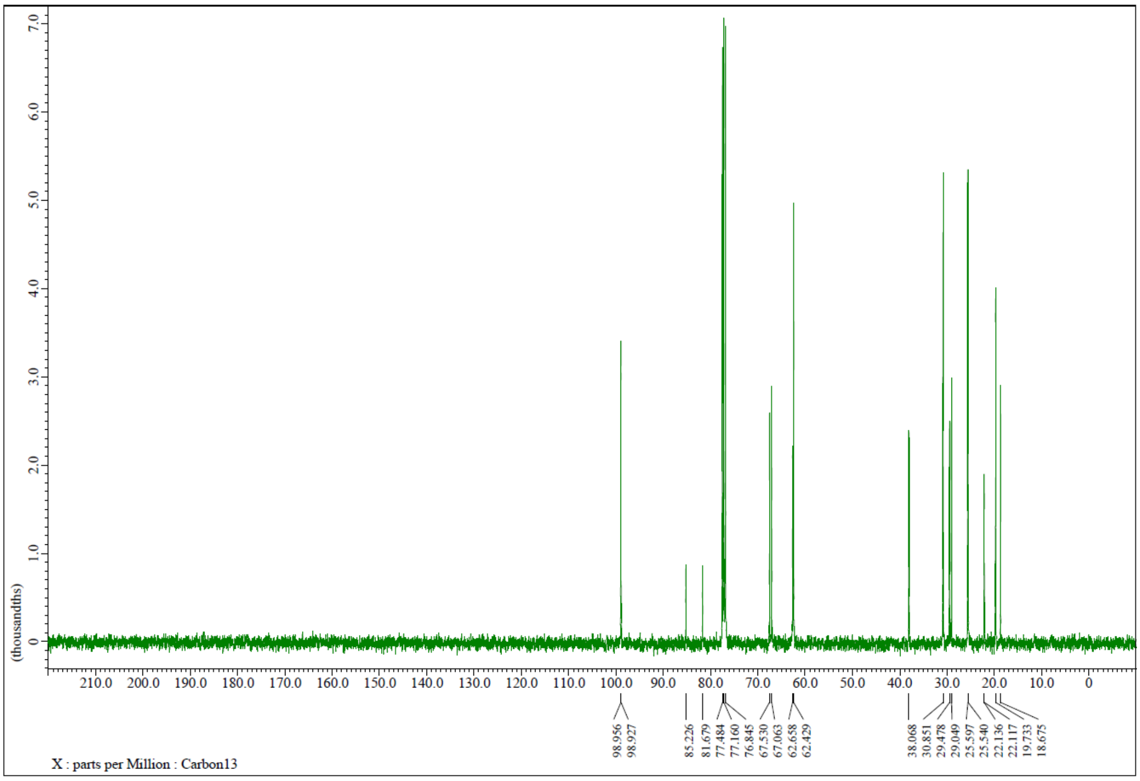

1,11-Bis((tetrahydro-2*H*-pyran-2-yl)oxy)undeca-5,6-diene (S9e)

(^{1}H NMR, 400 MHz, $CDCl_3$)

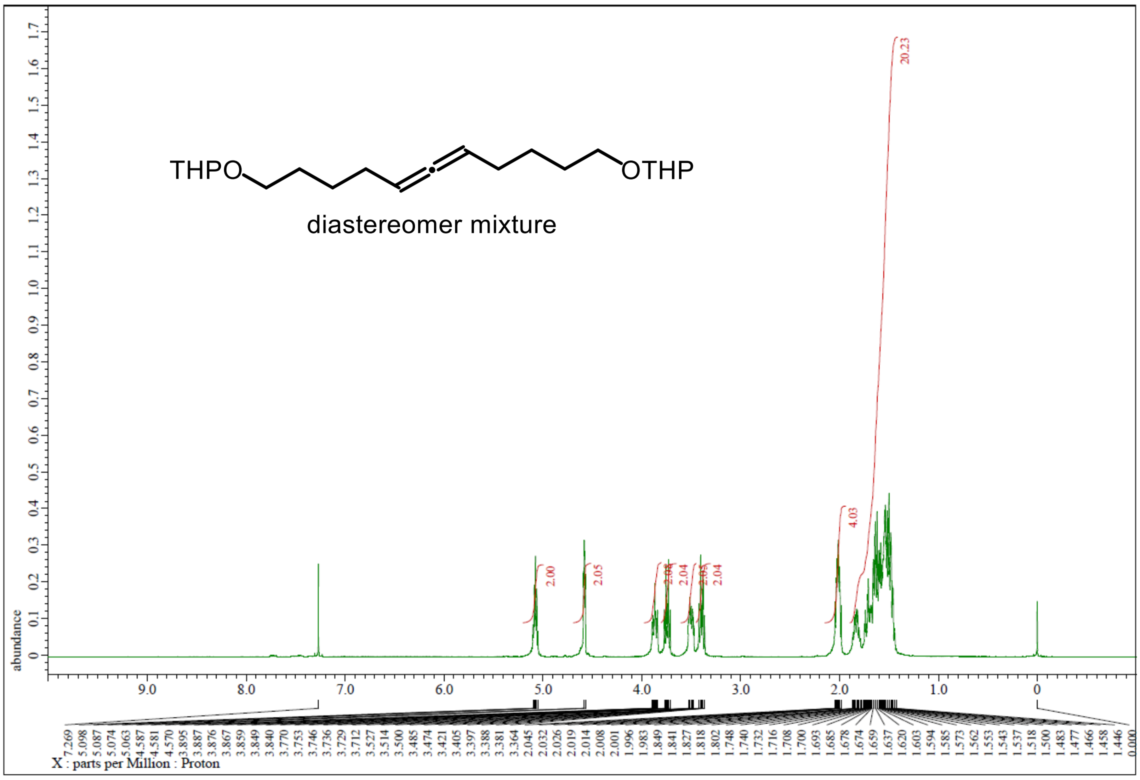

(^{13}C NMR, 100 MHz, $CDCl_3$)

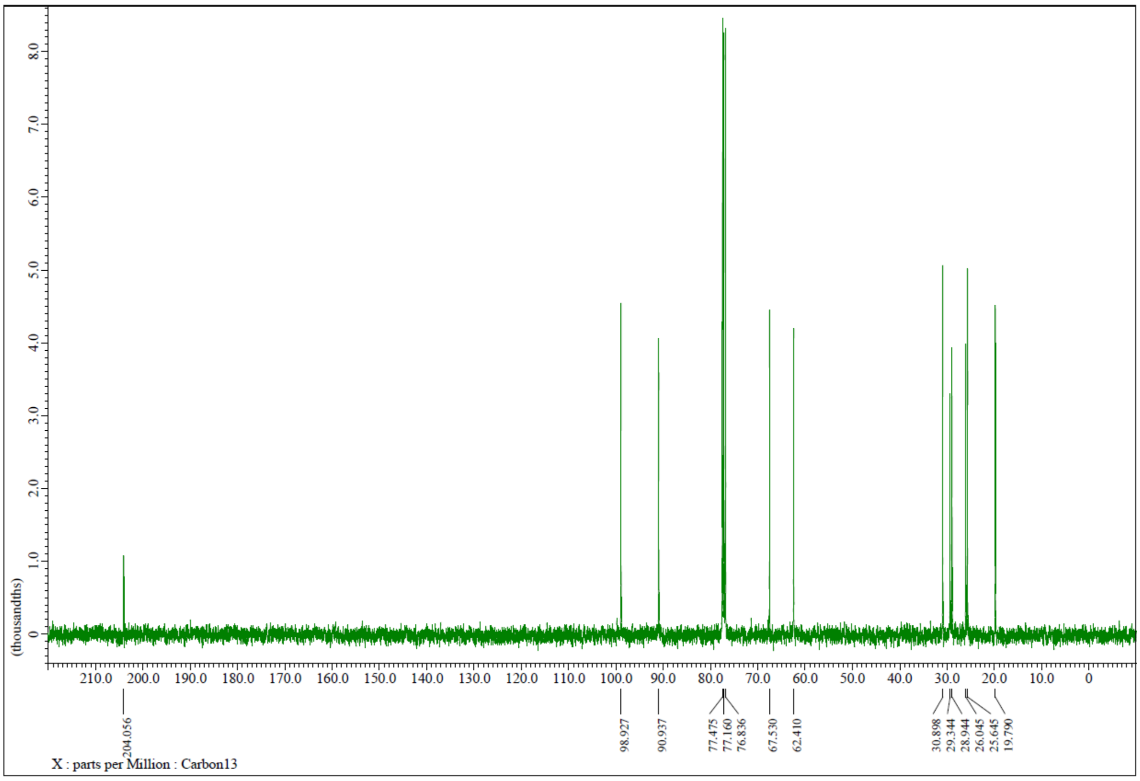

Undeca-5,6-diene-1,11-diol (S10e)

(^{1}H NMR, 400 MHz, $CDCl_3$)

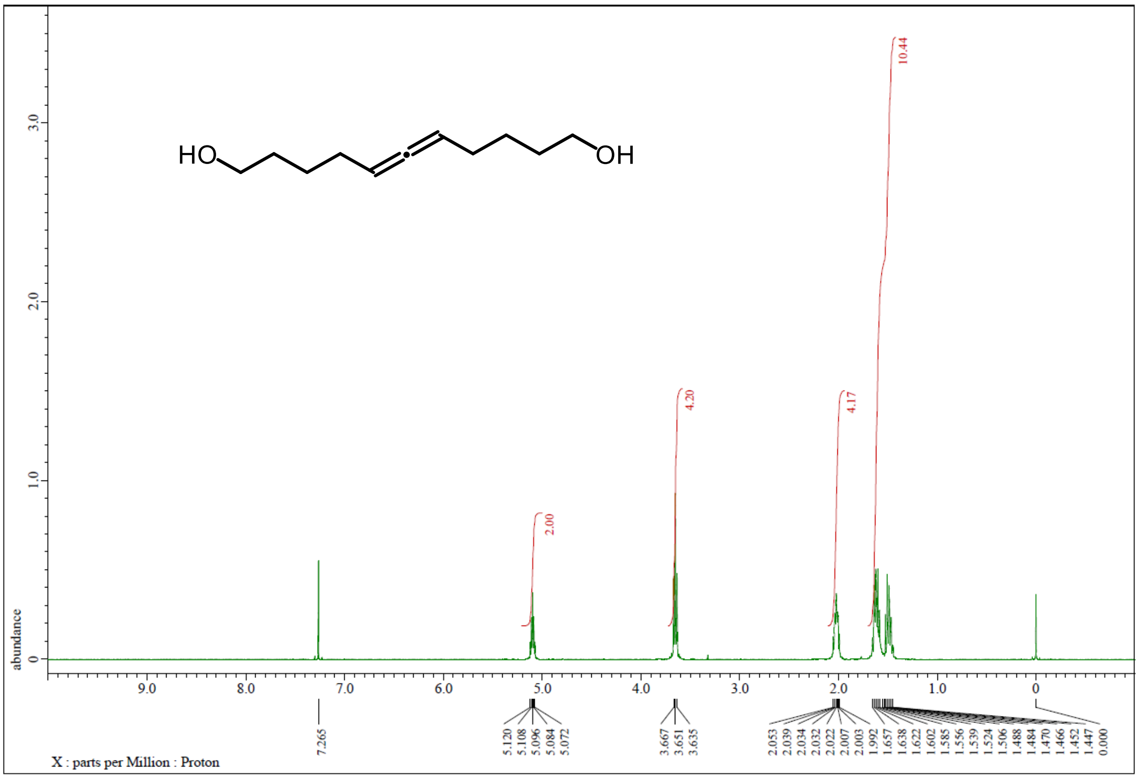

(^{13}C NMR, 100 MHz, $CDCl_3$)

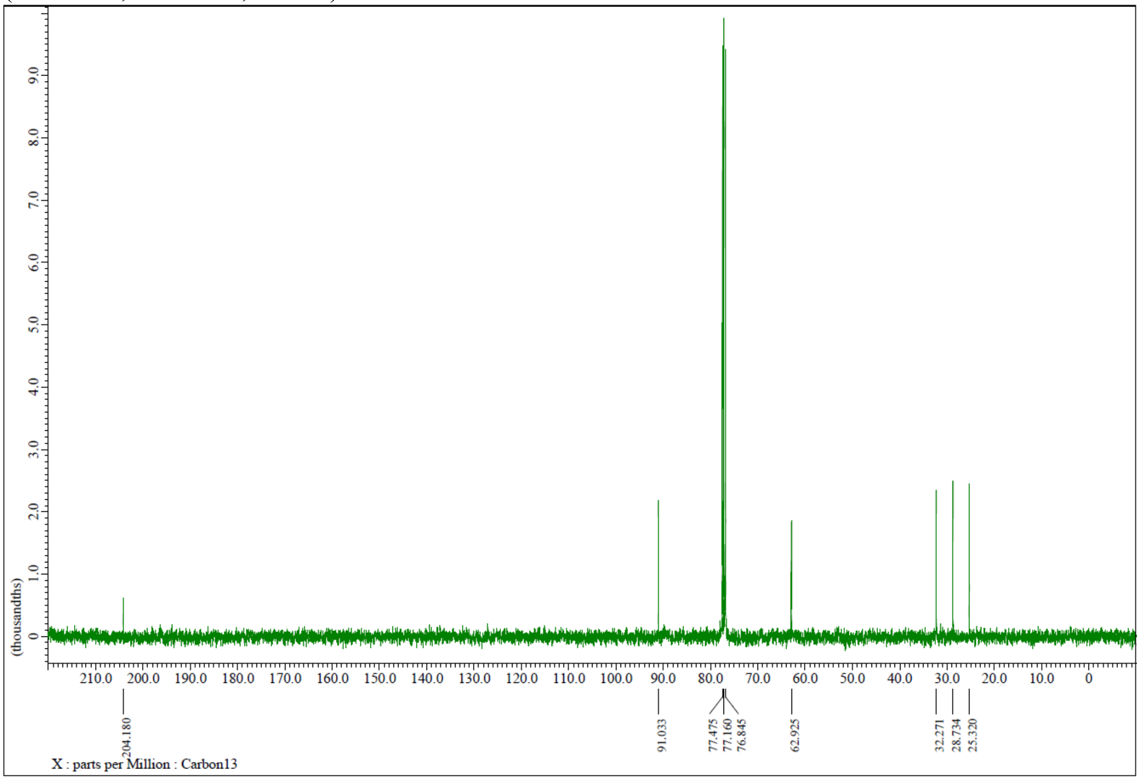

Undeca-5,6-dienedial (1e)

(^{1}H NMR, 400 MHz, $CDCl_3$)

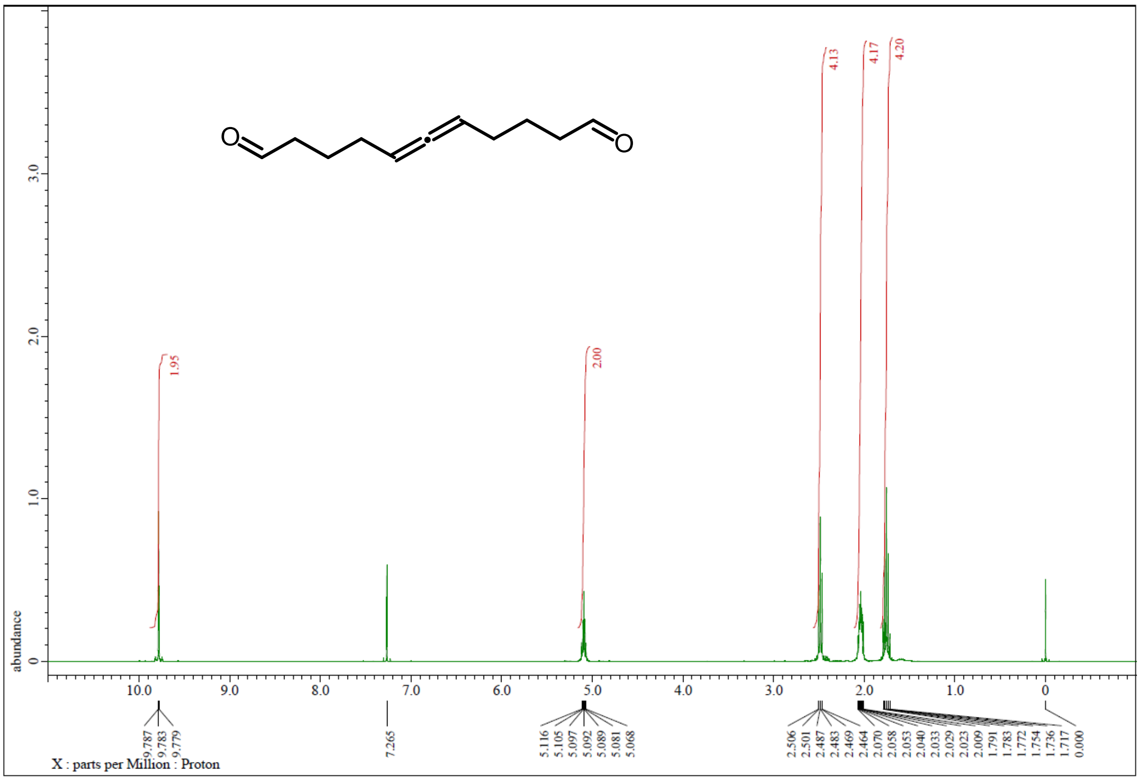

(^{13}C NMR, 100 MHz, $CDCl_3$)

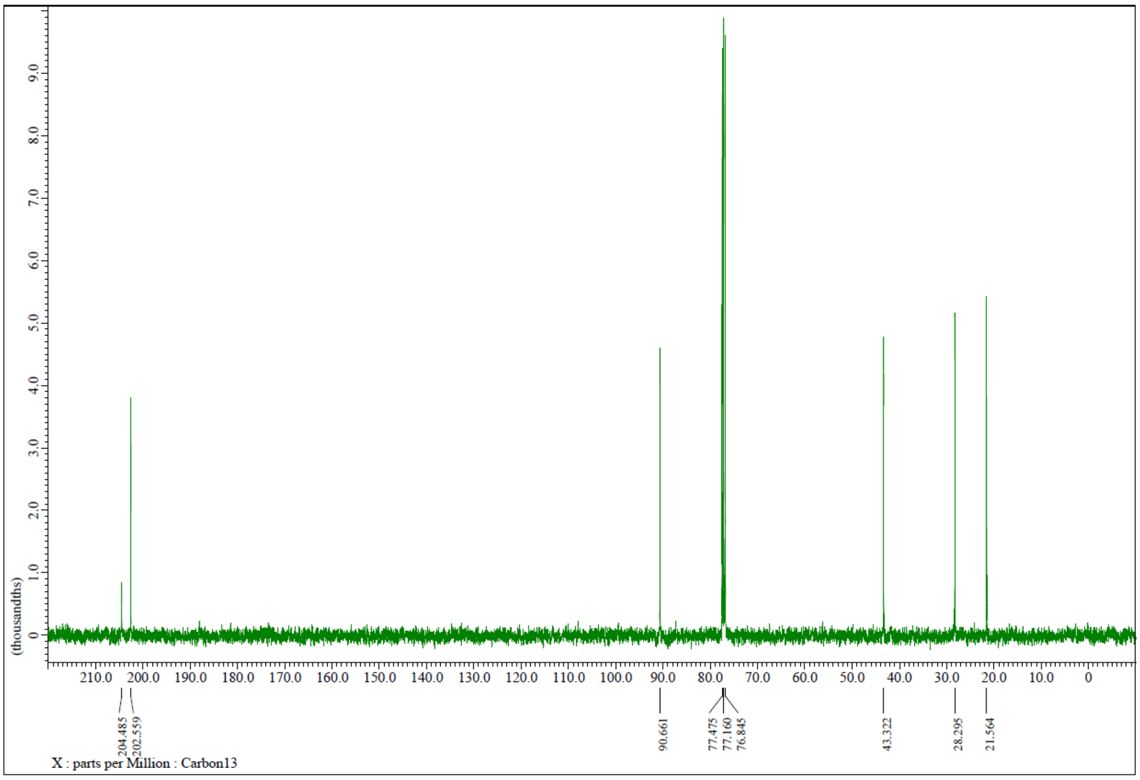

Undeca-5,6-dienedial bisoxime (4e)

(^{1}H NMR, 400 MHz, CD_3OD)

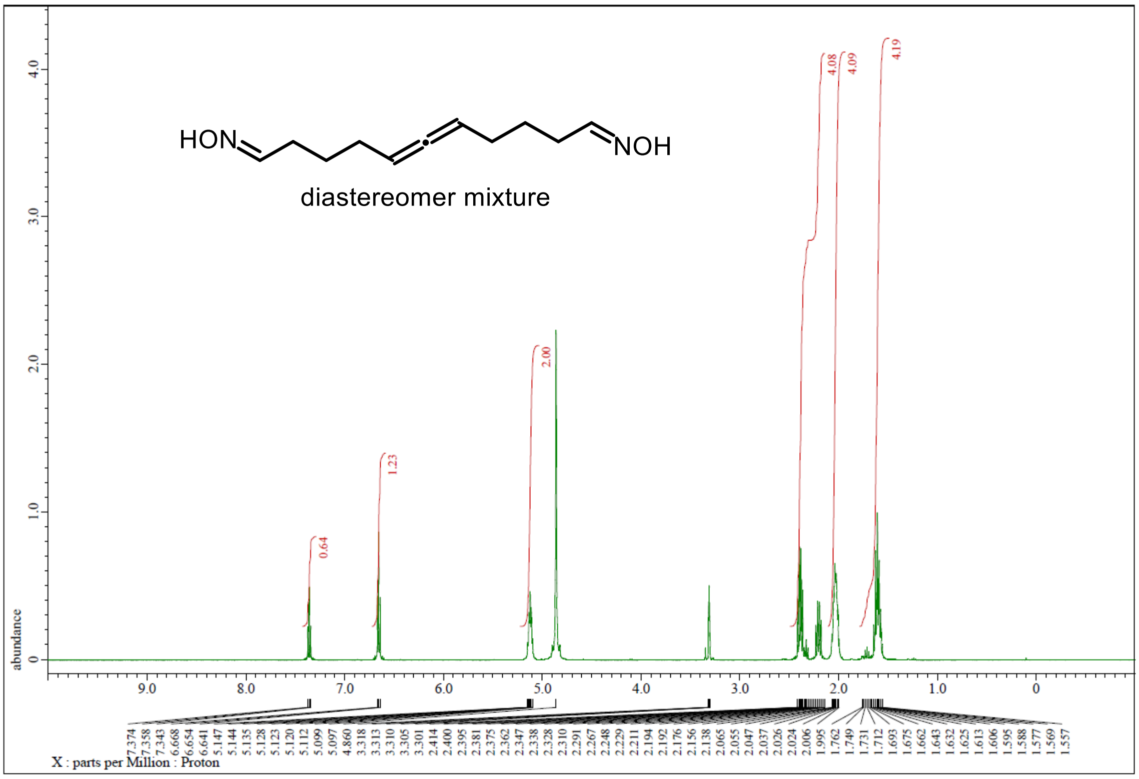

(^{13}C NMR, 100 MHz, CD_3OD)

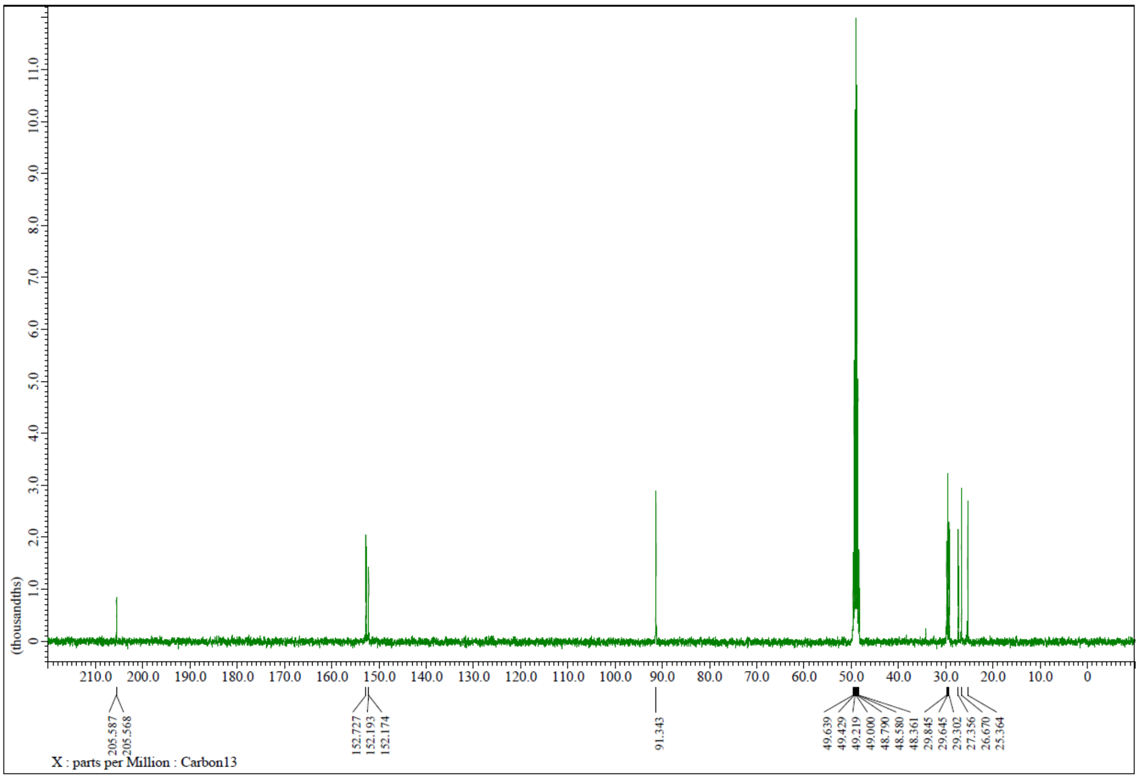

1,13-Bis((tetrahydro-2*H*-pyran-2-yl)oxy)tridec-7-yn-6-ol (S8f)

(^{1}H NMR, 400 MHz, $CDCl_3$)

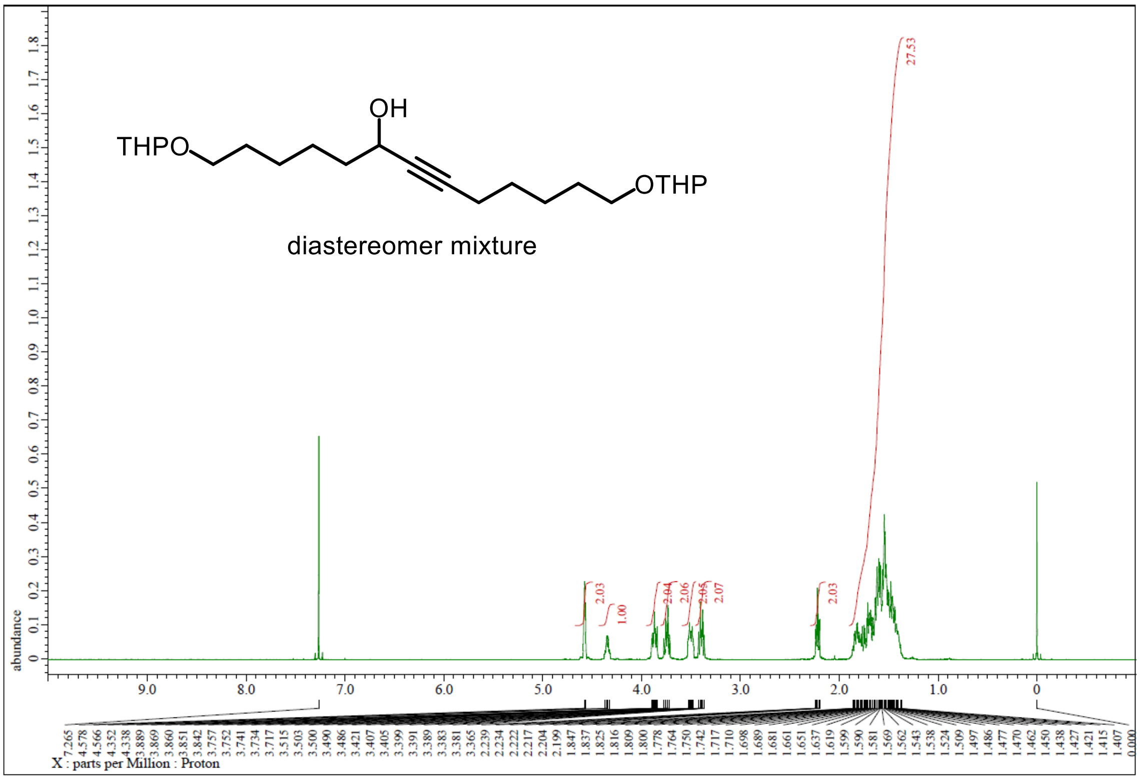

(^{13}C NMR, 100 MHz, $CDCl_3$)

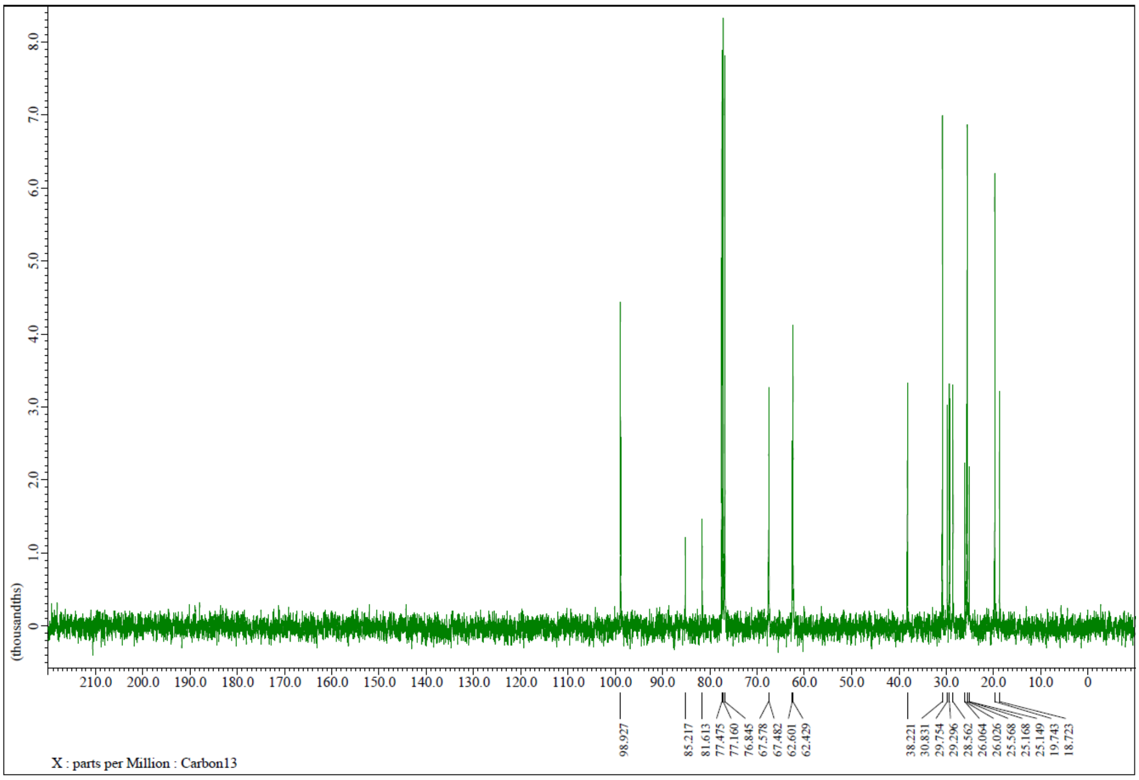

1,13-Bis((tetrahydro-2*H*-pyran-2-yl)oxy)trideca-6,7-diene (S9f)

(^{1}H NMR, 400 MHz, $CDCl_3$)

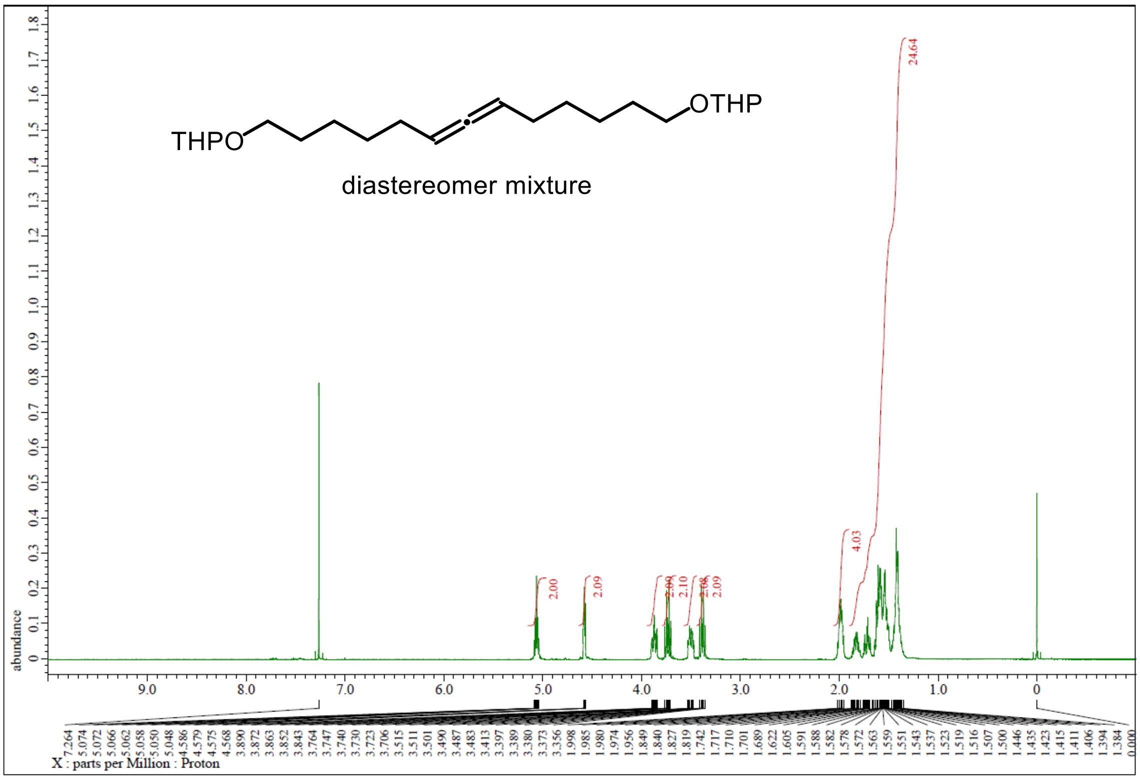

(^{13}C NMR, 100 MHz, $CDCl_3$)

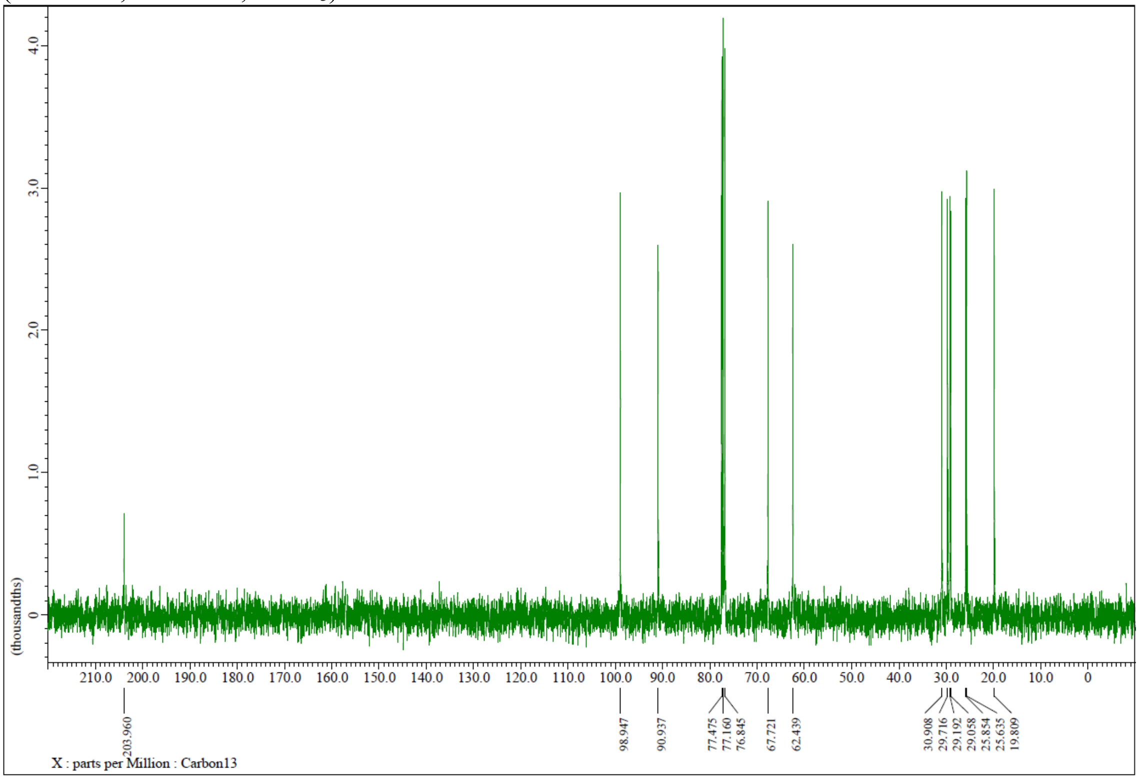

Trideca-6,7-diene-1,13-diol (S10f)

(^{1}H NMR, 400 MHz, $CDCl_3$)

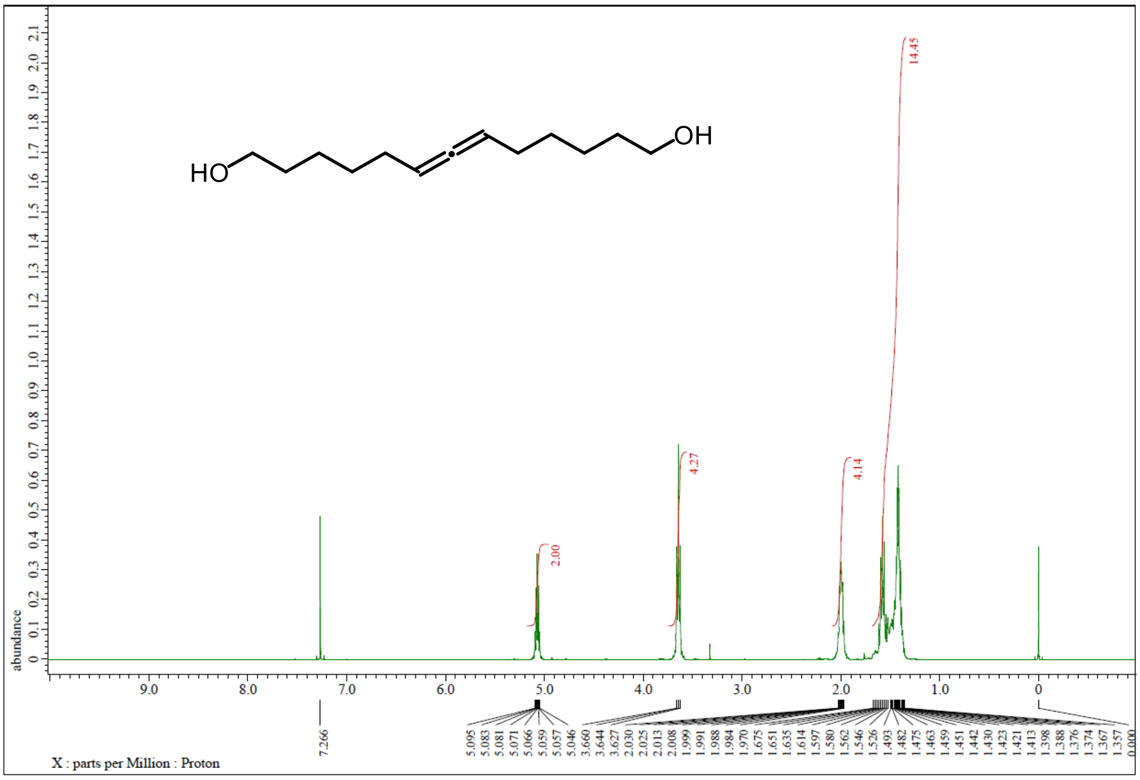

(^{13}C NMR, 100 MHz, $CDCl_3$)

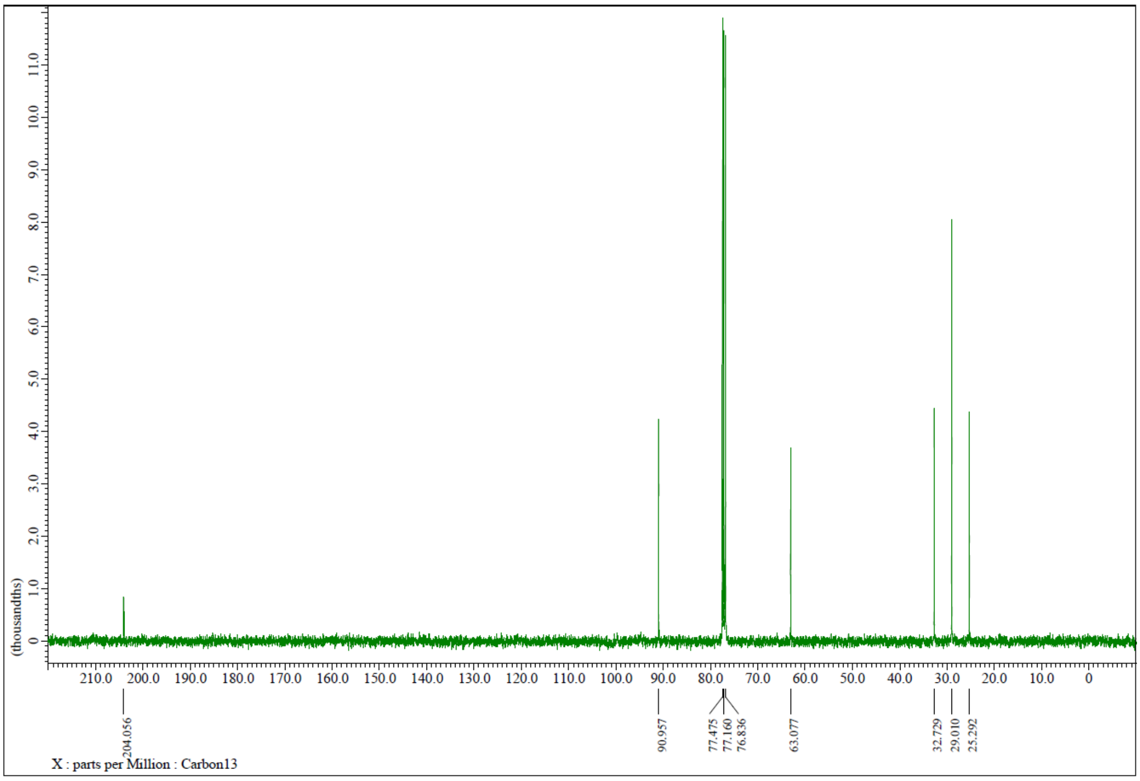

Trideca-6,7-dienedial (1f)

(^{1}H NMR, 400 MHz, $CDCl_3$)

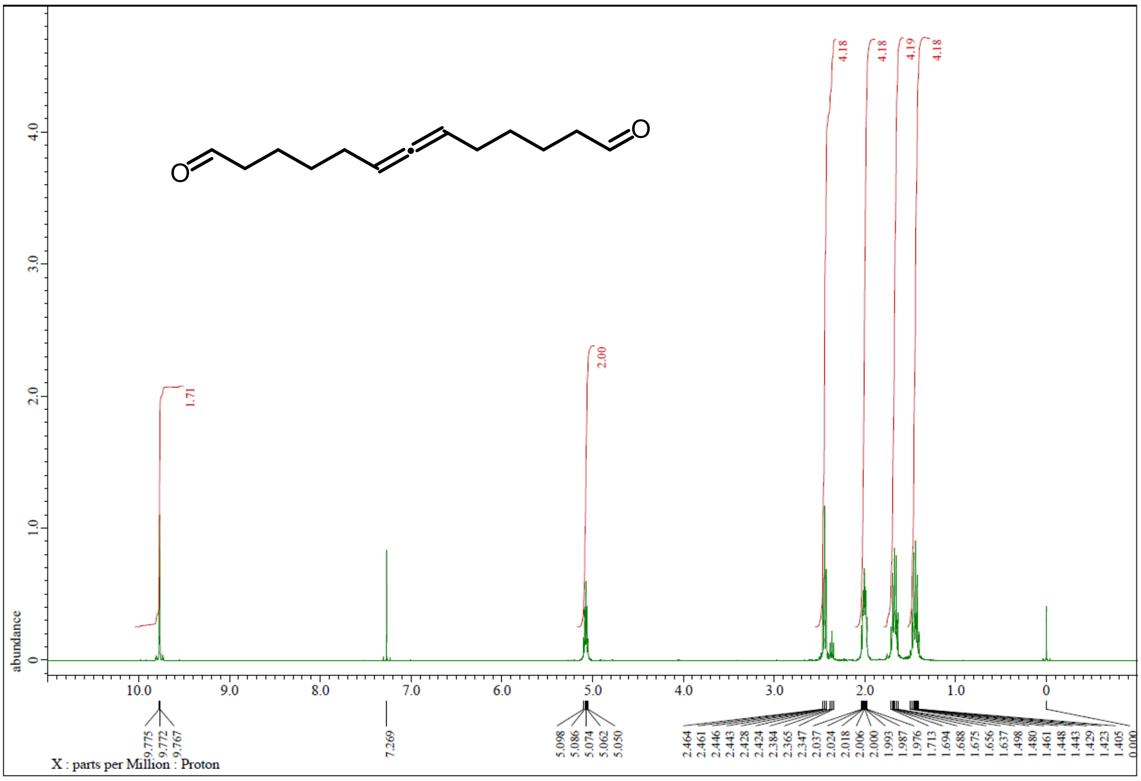

(^{13}C NMR, 100 MHz, $CDCl_3$)

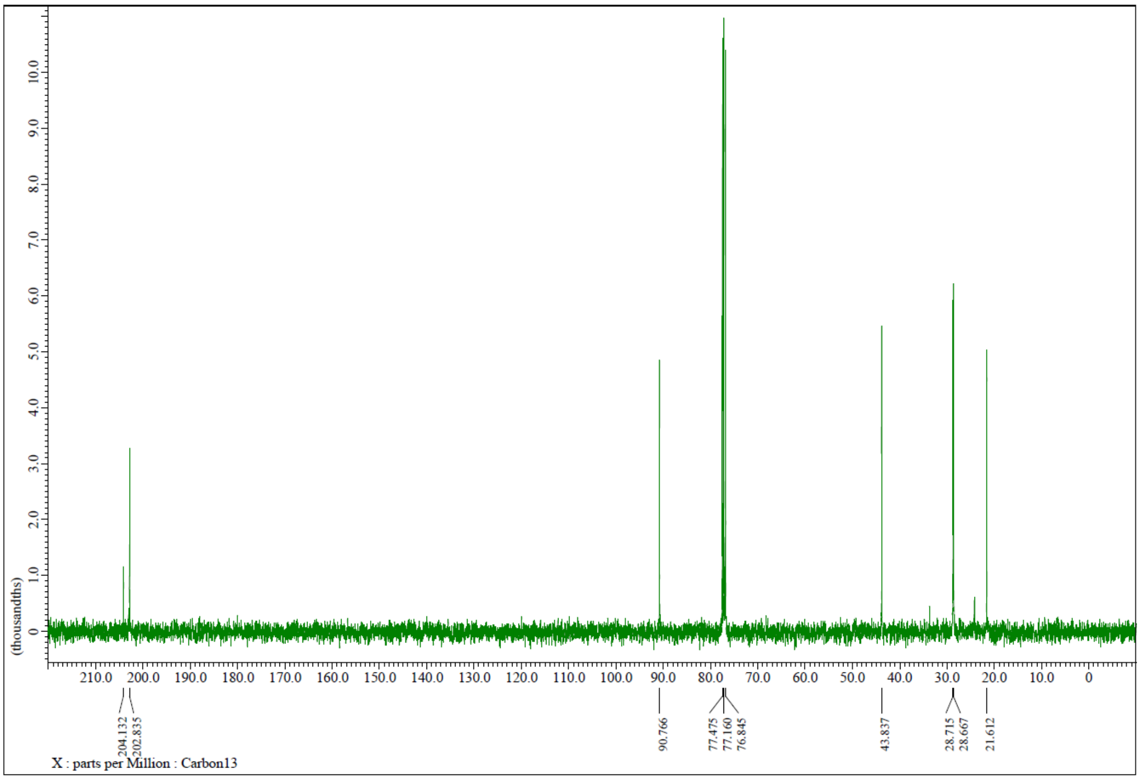

N-(4-Methoxybenzyl)hydroxylamine (S11a)

(^{1}H NMR, 400 MHz, $CDCl_3$)

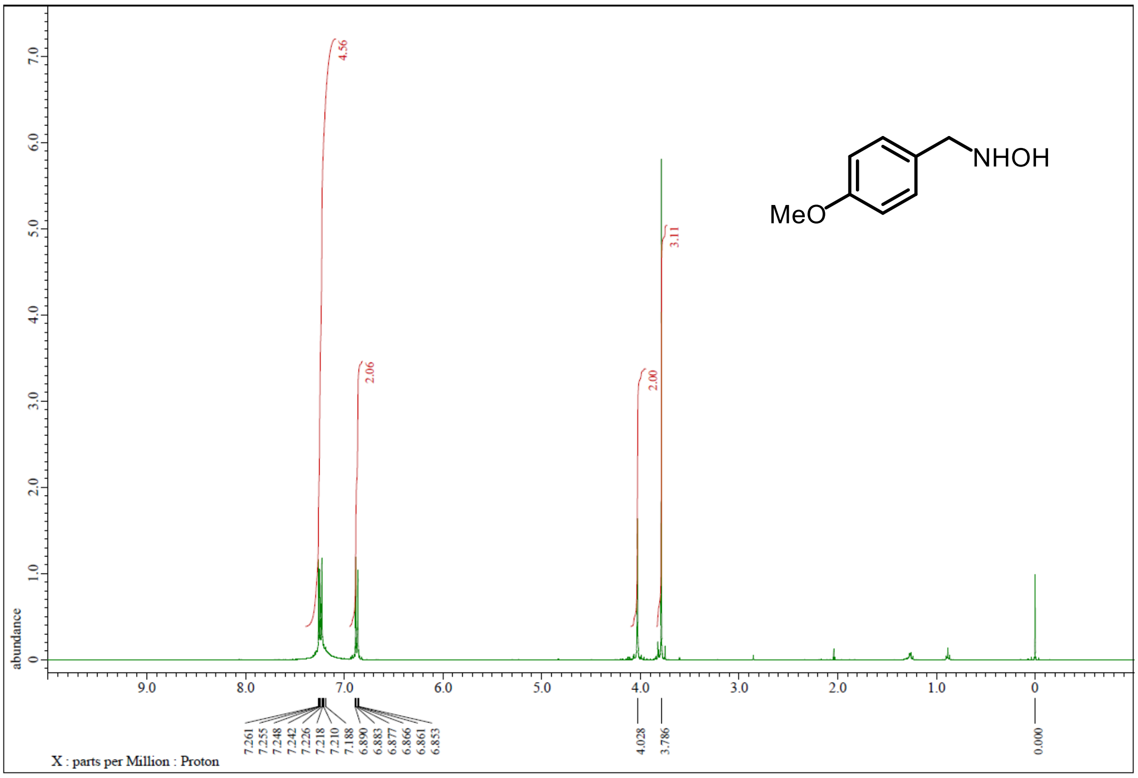

(^{13}C NMR, 100 MHz, $CDCl_3$)

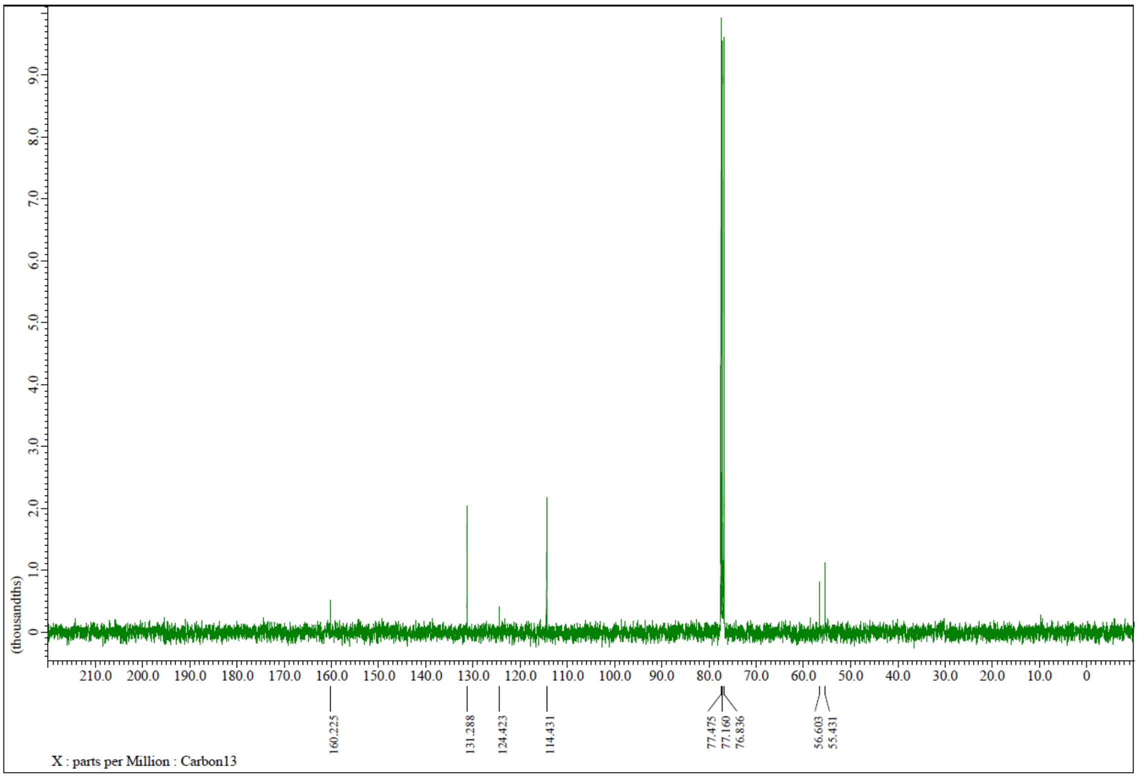

N-(4-Bromobenzyl)hydroxylamine (S11b)

(^{1}H NMR, 400 MHz, $CDCl_3$)

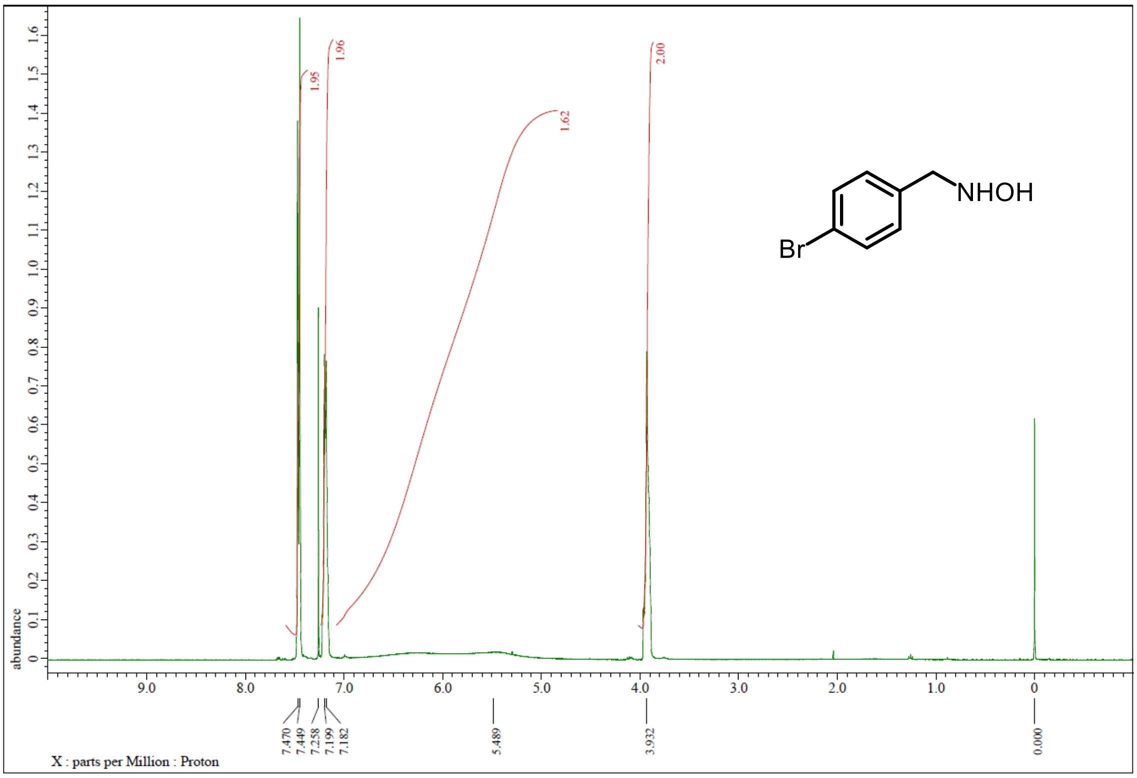

(^{13}C NMR, 100 MHz, $CDCl_3$)

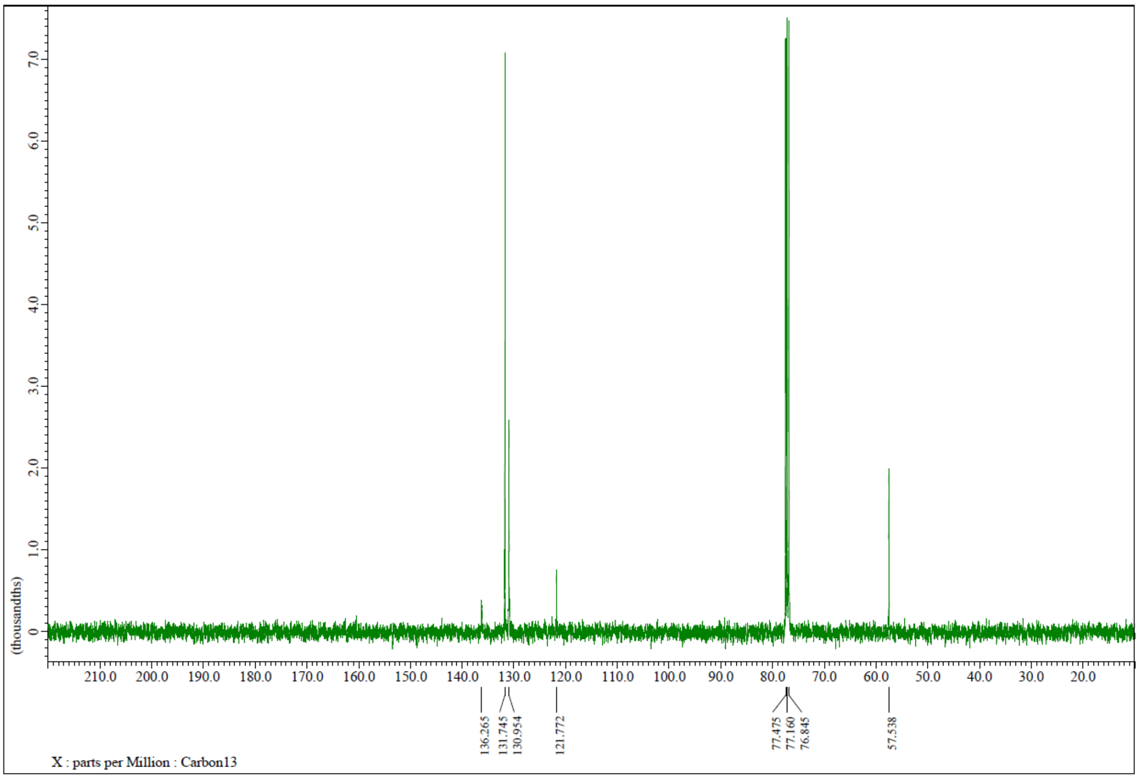

***t*-Butyl(2-nitroethoxy)diphenylsilane (S12a)**

(^{1}H NMR, 400 MHz, $CDCl_3$)

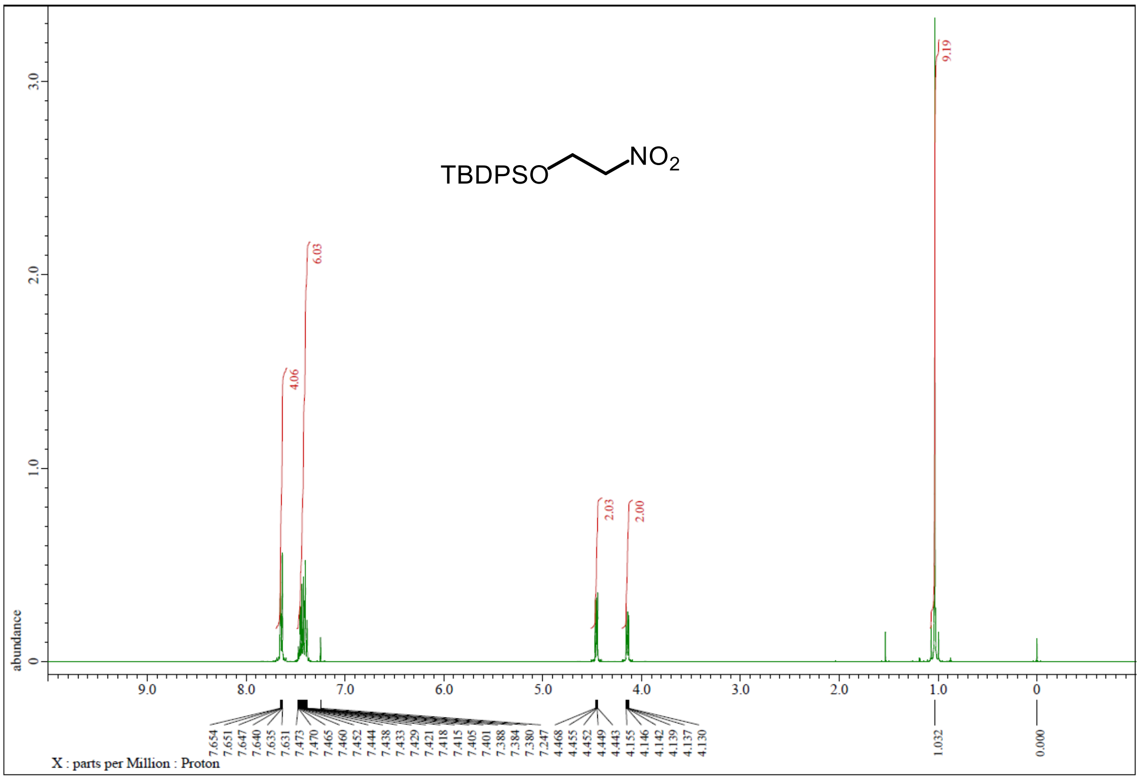

(^{13}C NMR, 100 MHz, $CDCl_3$)

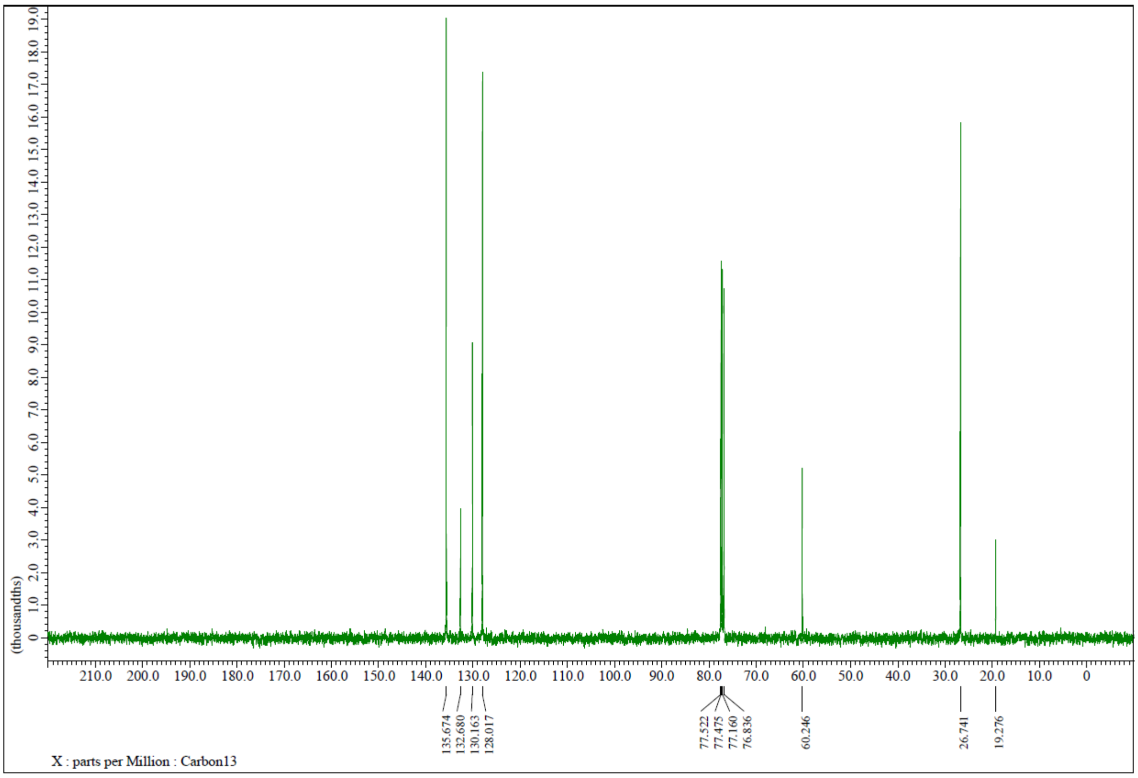

_N_-(2-((_t_-Butyldiphenylsilyl)oxy)ethyl)hydroxylamine (S11c)

(^{1}H NMR, 400 MHz, $CDCl_3$)

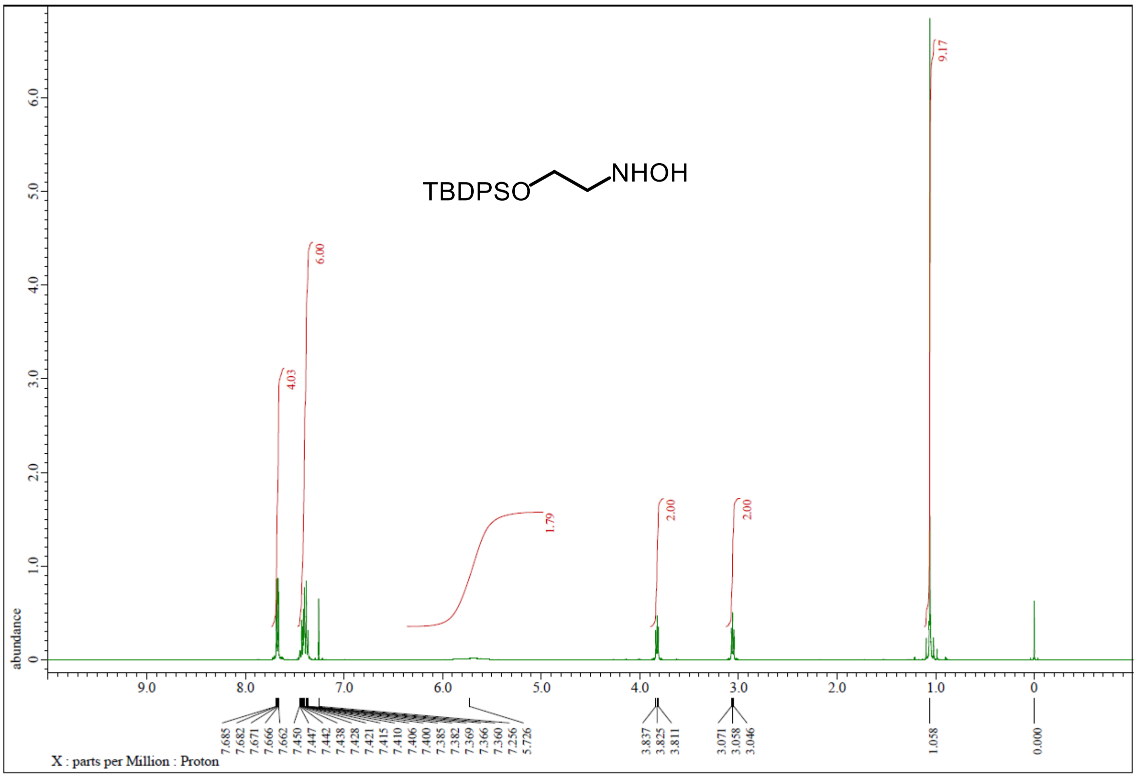

(^{13}C NMR, 100 MHz, $CDCl_3$)

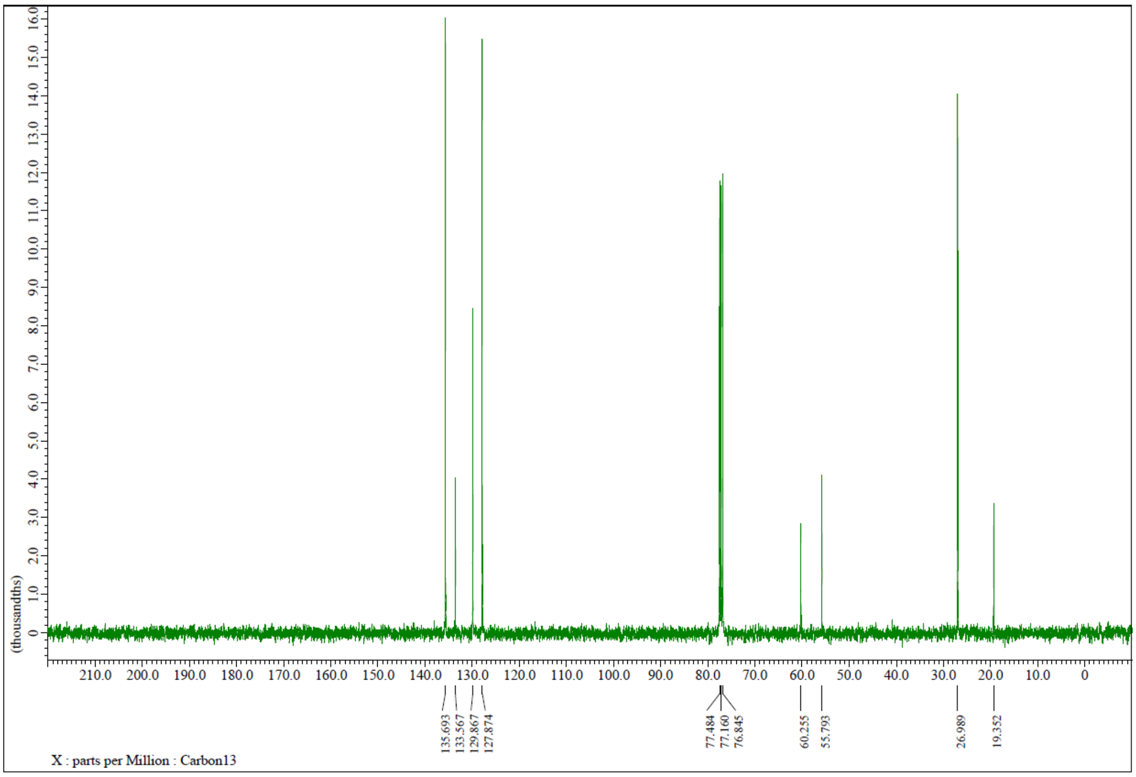

2-(2-Nitroethoxy)tetrahydro-2*H*-pyran (S12b)

(^{1}H NMR, 400 MHz, $CDCl_3$)

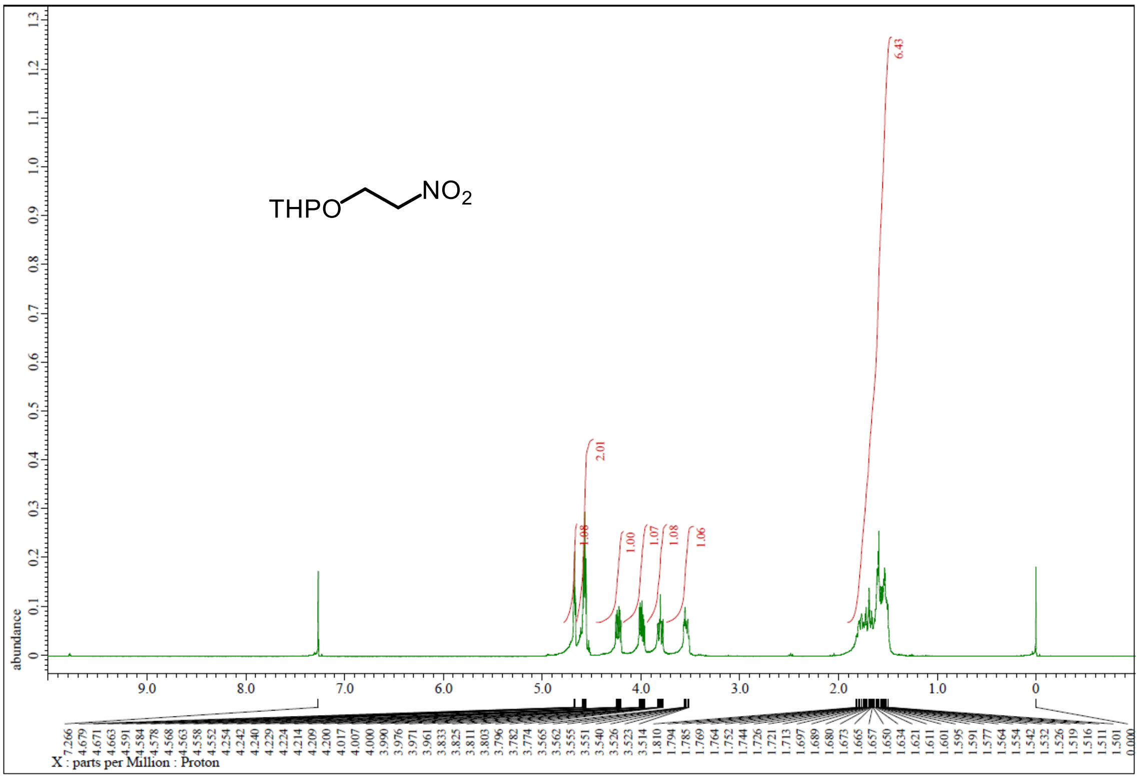

(^{13}C NMR, 100 MHz, $CDCl_3$)

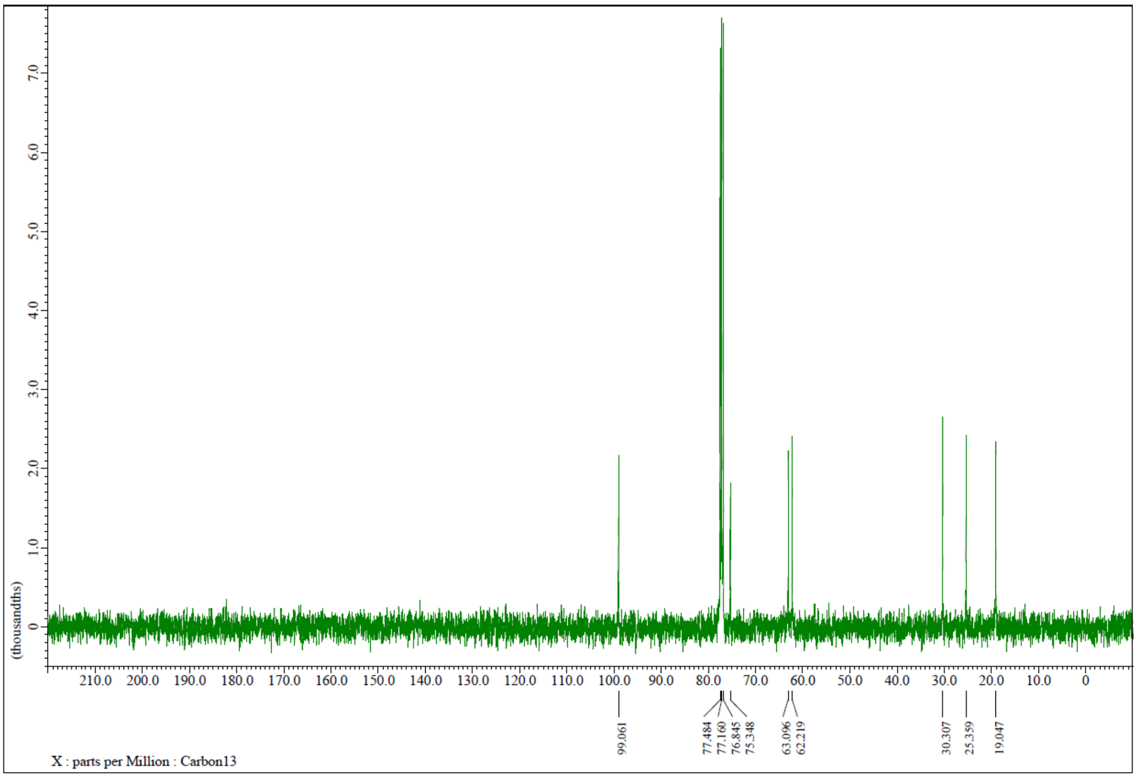

_N_-(2-((Tetrahydro-2_H_-pyran-2-yl)oxy)ethyl)hydroxylamine (S11d)

(^{1}H NMR, 400 MHz, $CDCl_3$)

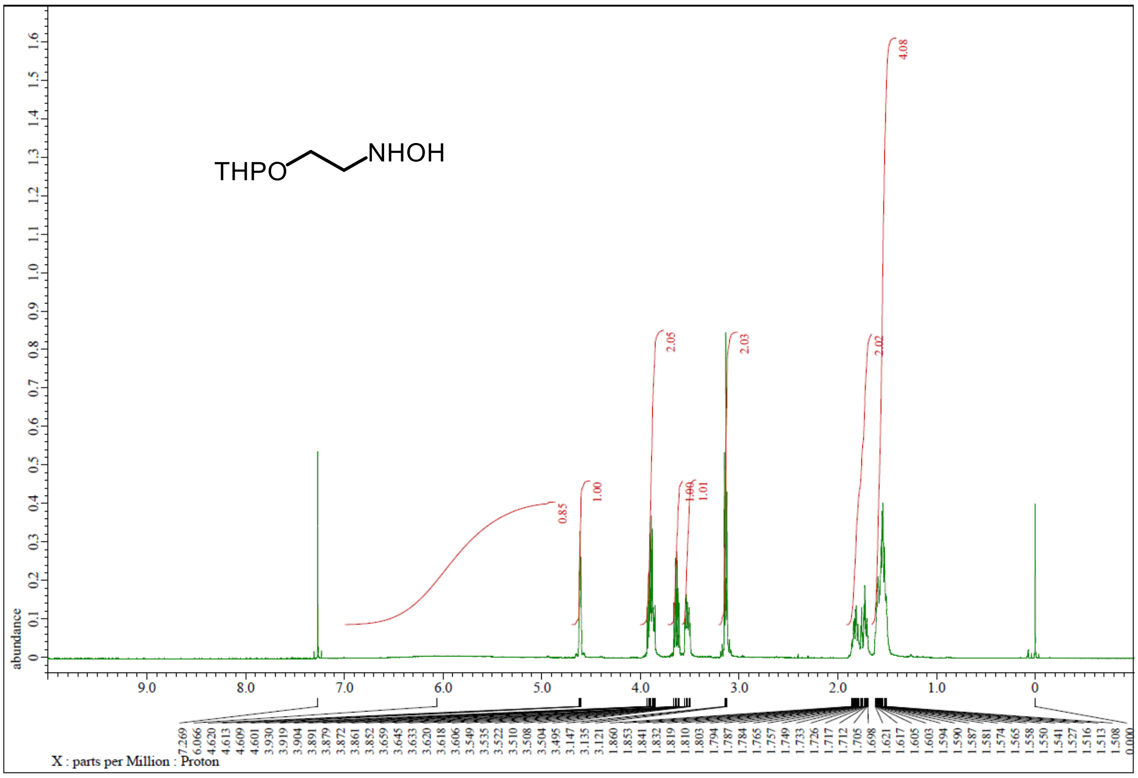

(^{13}C NMR, 100 MHz, $CDCl_3$)

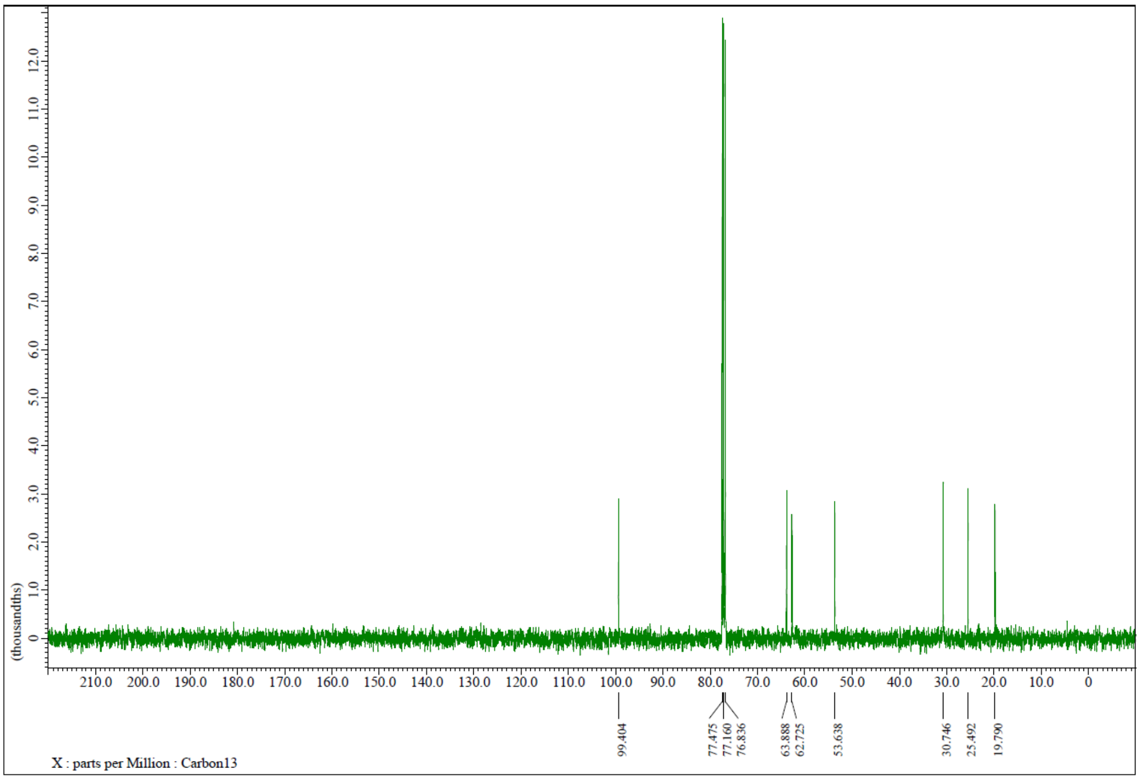

***N*-(Furan-3-ylmethyl)hydroxylamine (S11e)**

(^{1}H NMR, 400 MHz, $CDCl_3$)

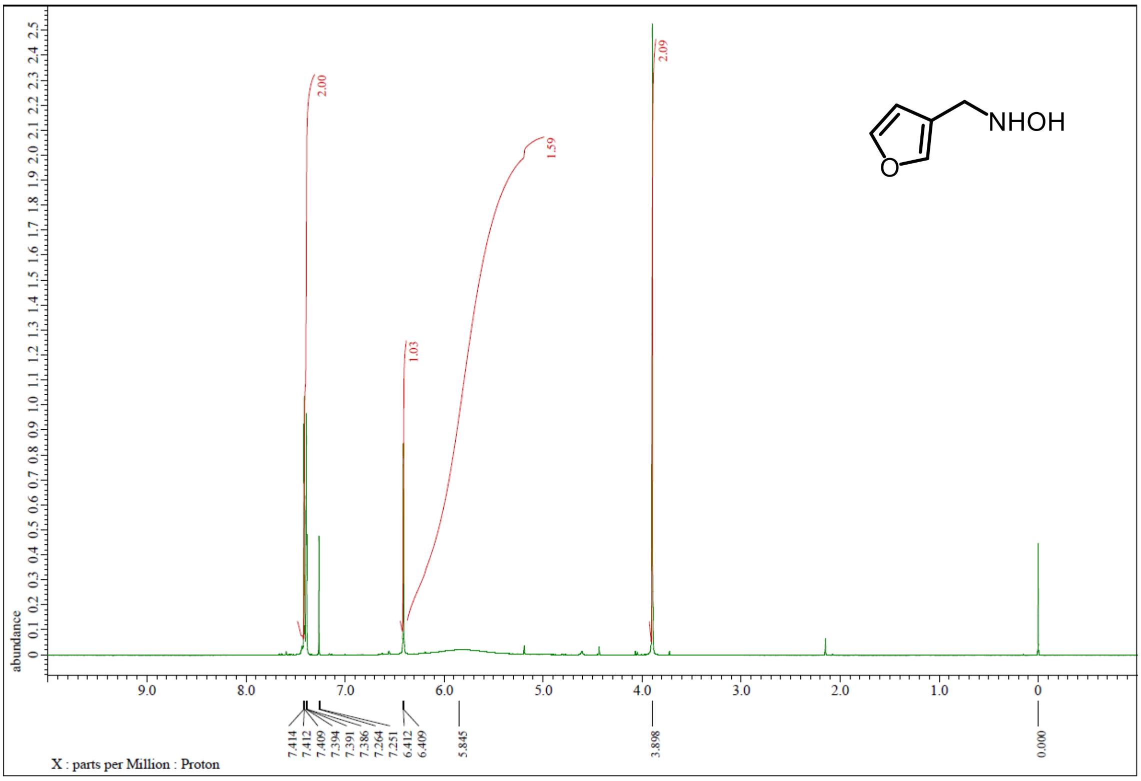

(^{13}C NMR, 100 MHz, $CDCl_3$)

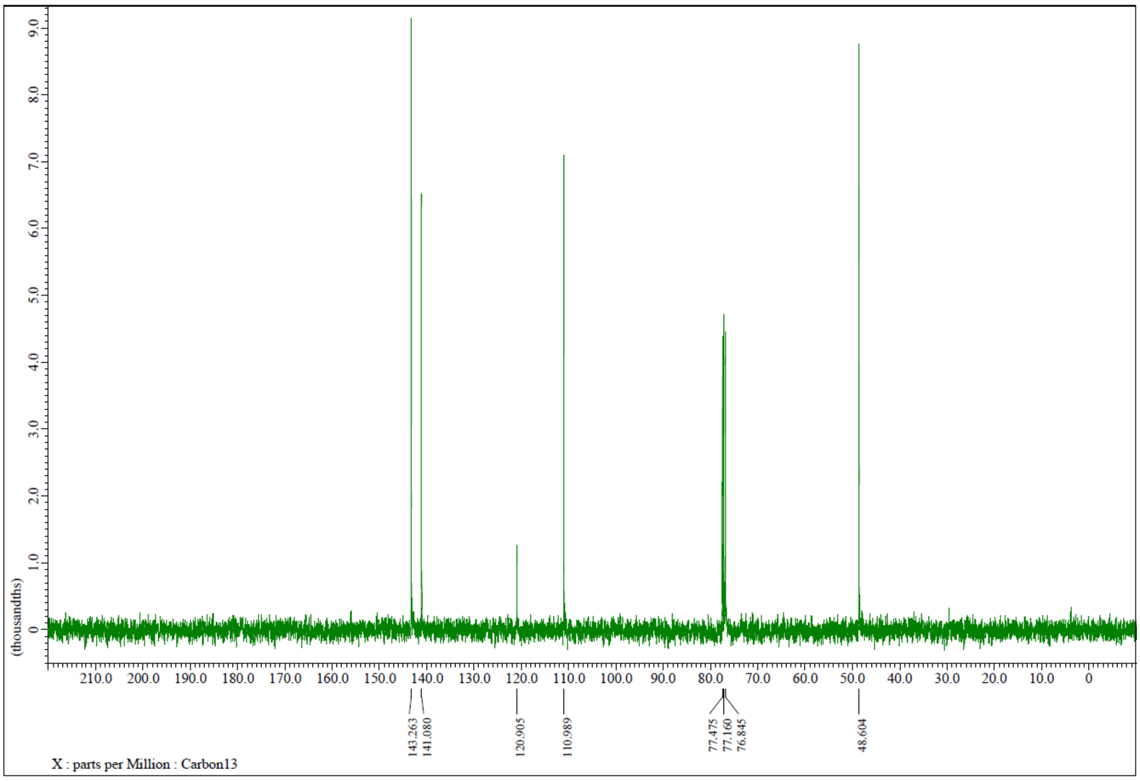

Methyl (*t*-butoxycarbonyl)-L-lysinate (S13a)

(^{1}H NMR, 400 MHz, $CDCl_3$)

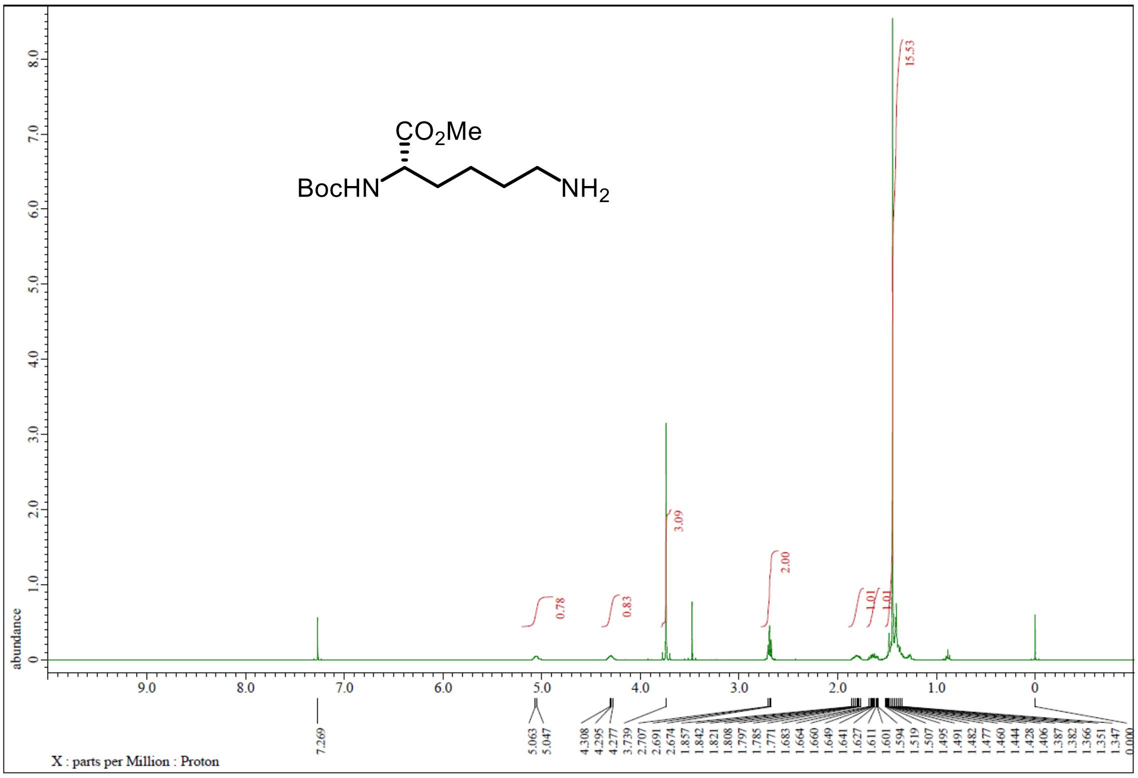

(^{13}C NMR, 100 MHz, $CDCl_3$)

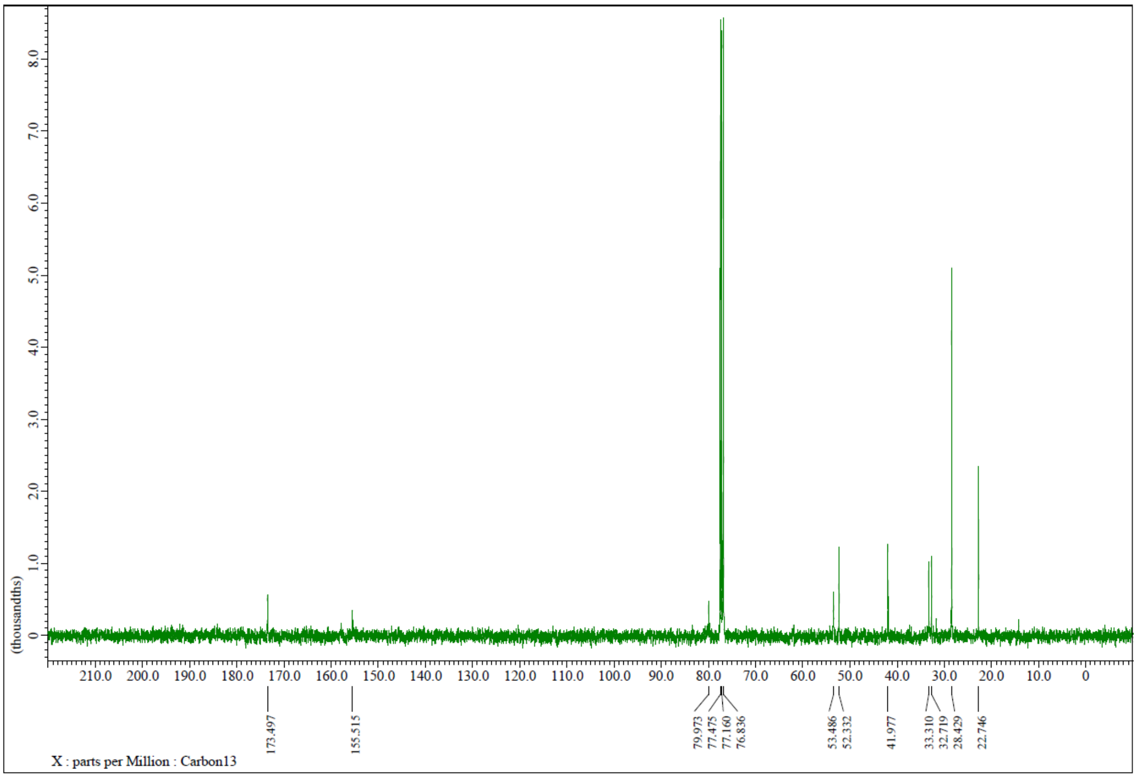

Methyl N^2-(*t*-butoxycarbonyl)-N^6-(cyanomethyl)-L-lysinate (S14a)

(^{1}H NMR, 400 MHz, $CDCl_3$)

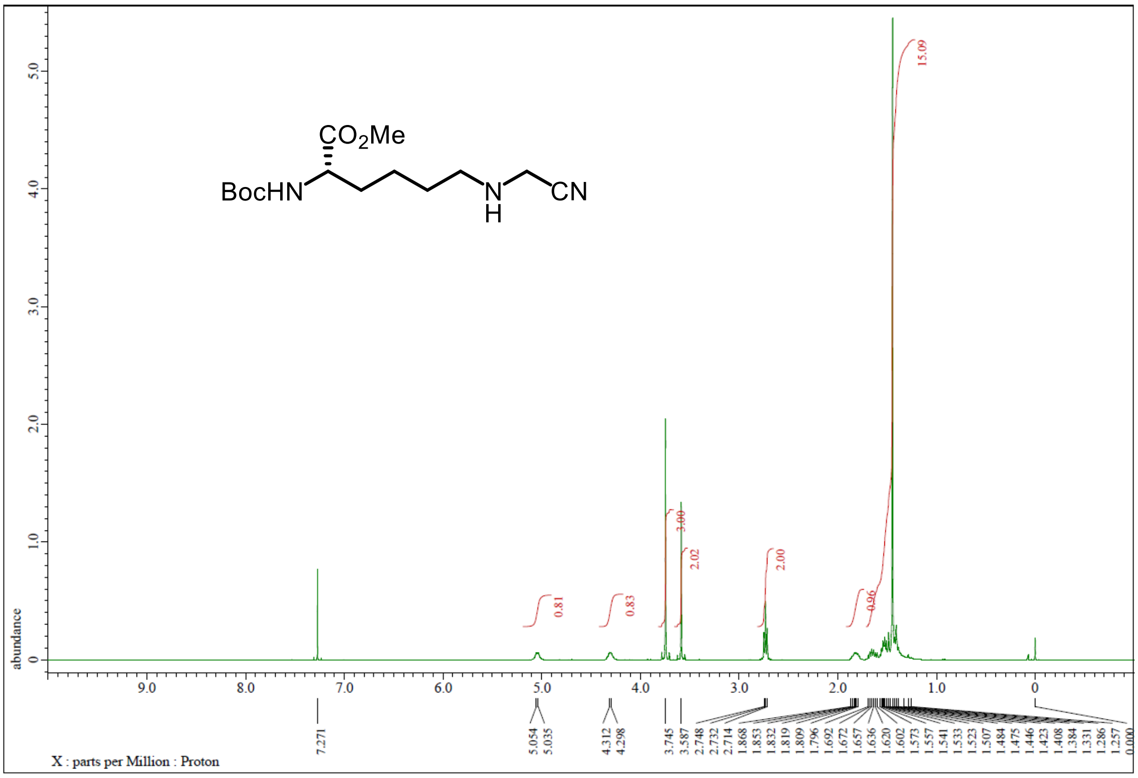

(^{13}C NMR, 100 MHz, $CDCl_3$)

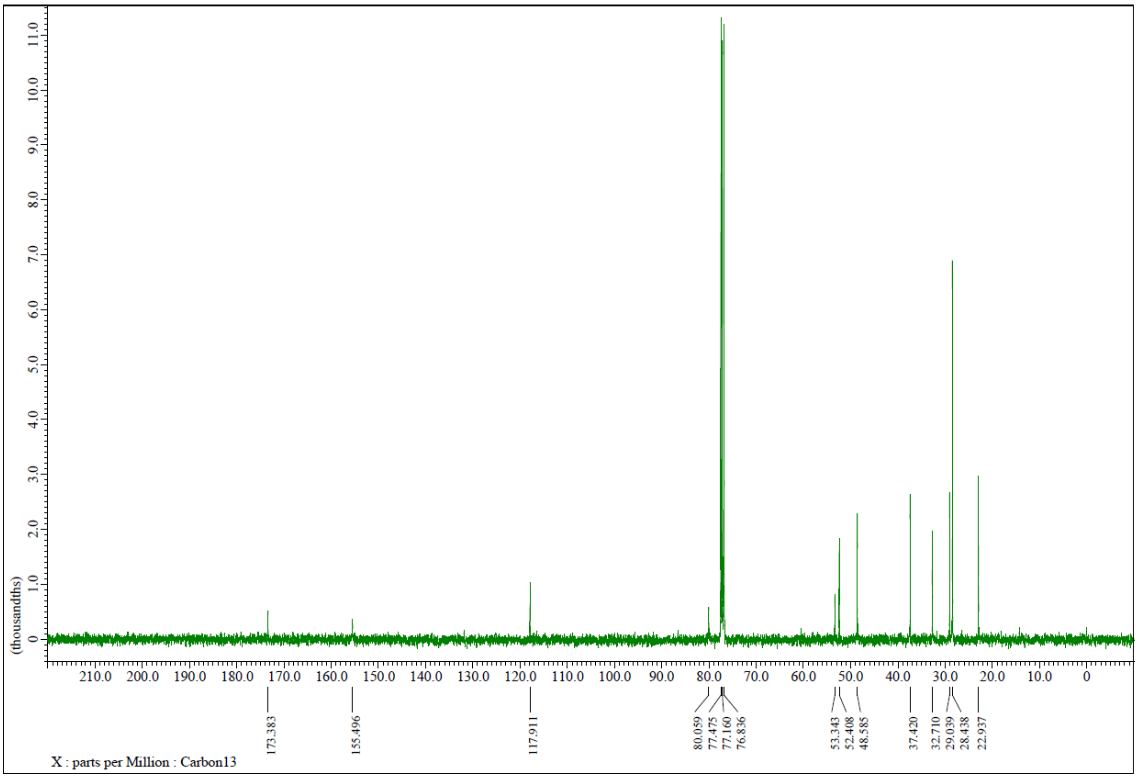

Methyl N^2-(*t*-butoxycarbonyl)-N^6-hydroxy-L-lysinate (S11f)

(^{1}H NMR, 400 MHz, $CDCl_3$)

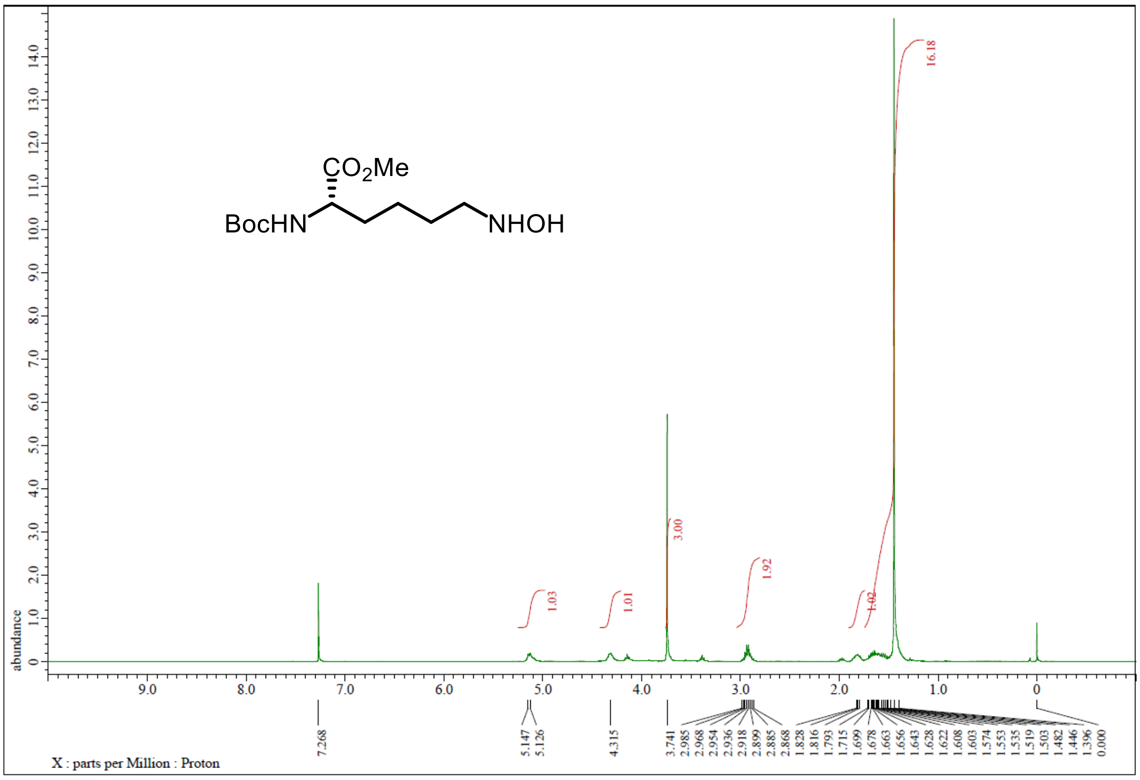

(^{13}C NMR, 100 MHz, $CDCl_3$)

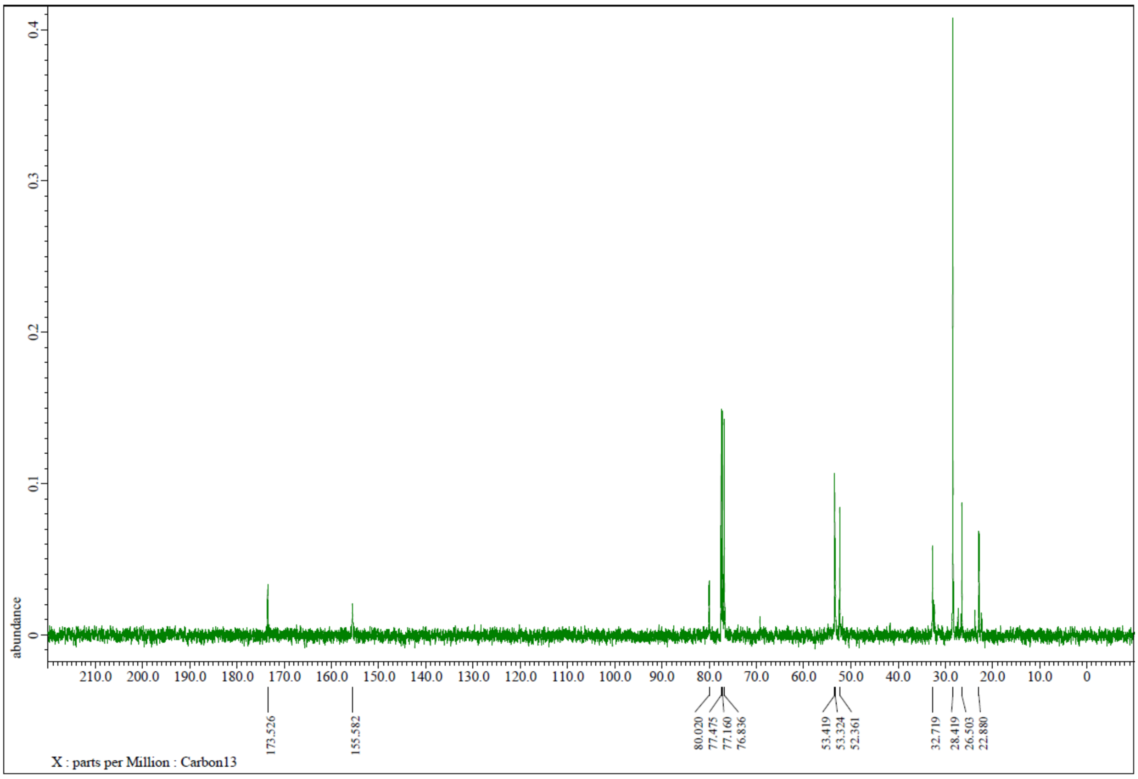

***t*-Butyl N^2-(((9*H*-fluoren-9-yl)methoxy)carbonyl)-N^6-(cyanomethyl)-L-lysinate (S14b)**

(^{1}H NMR, 400 MHz, $CDCl_3$)

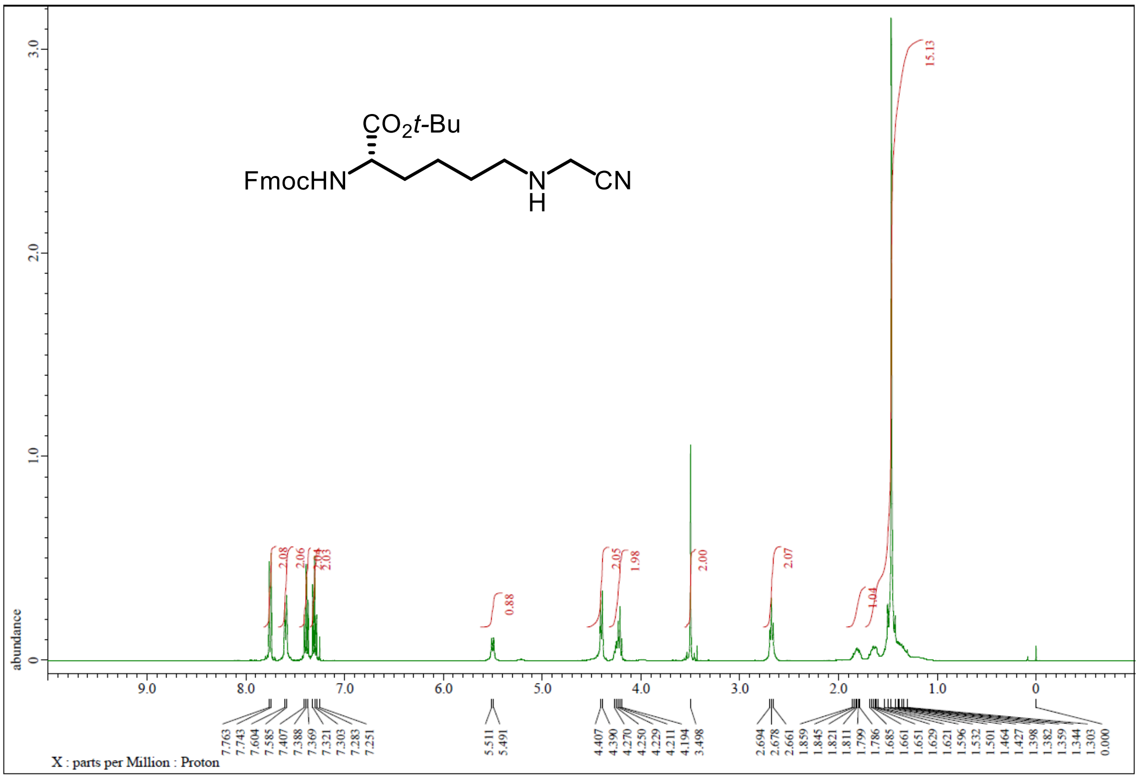

(^{13}C NMR, 100 MHz, $CDCl_3$)

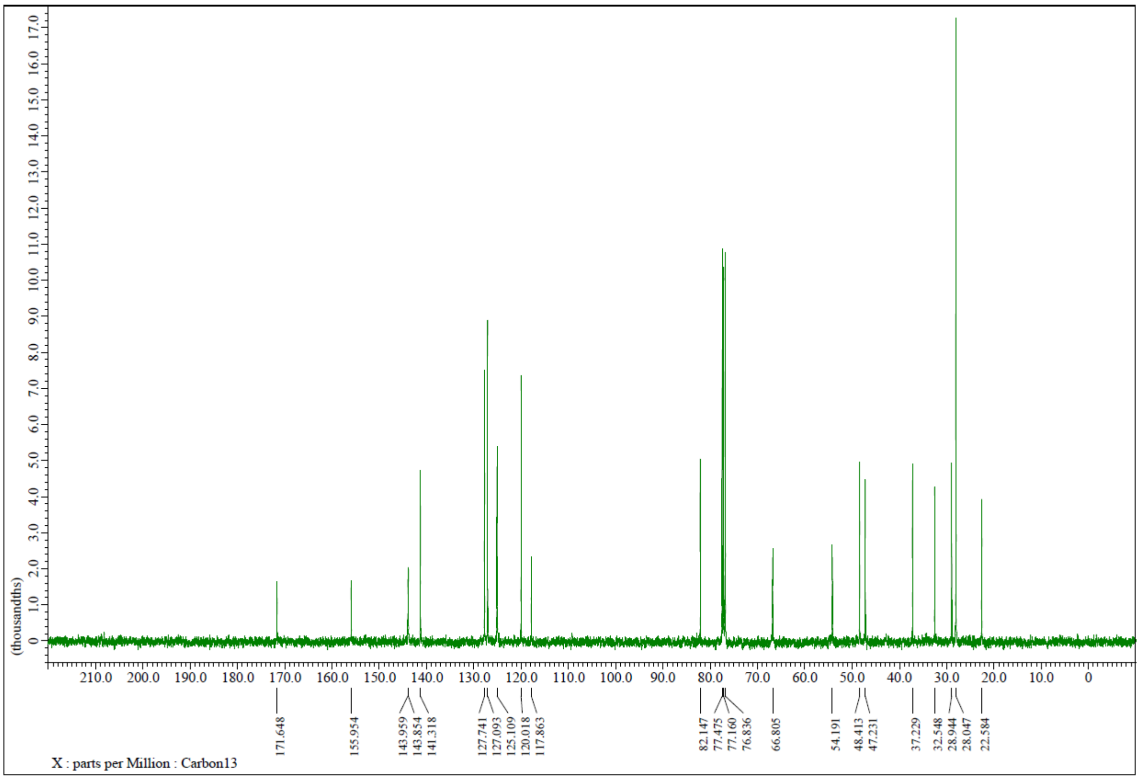

***t*-Butyl *N*²-(((9*H*-fluoren-9-yl)methoxy)carbonyl)-*N*⁶-hydroxy-L-lysinate (S11g)**

(^{1}H NMR, 400 MHz, $CDCl_3$)

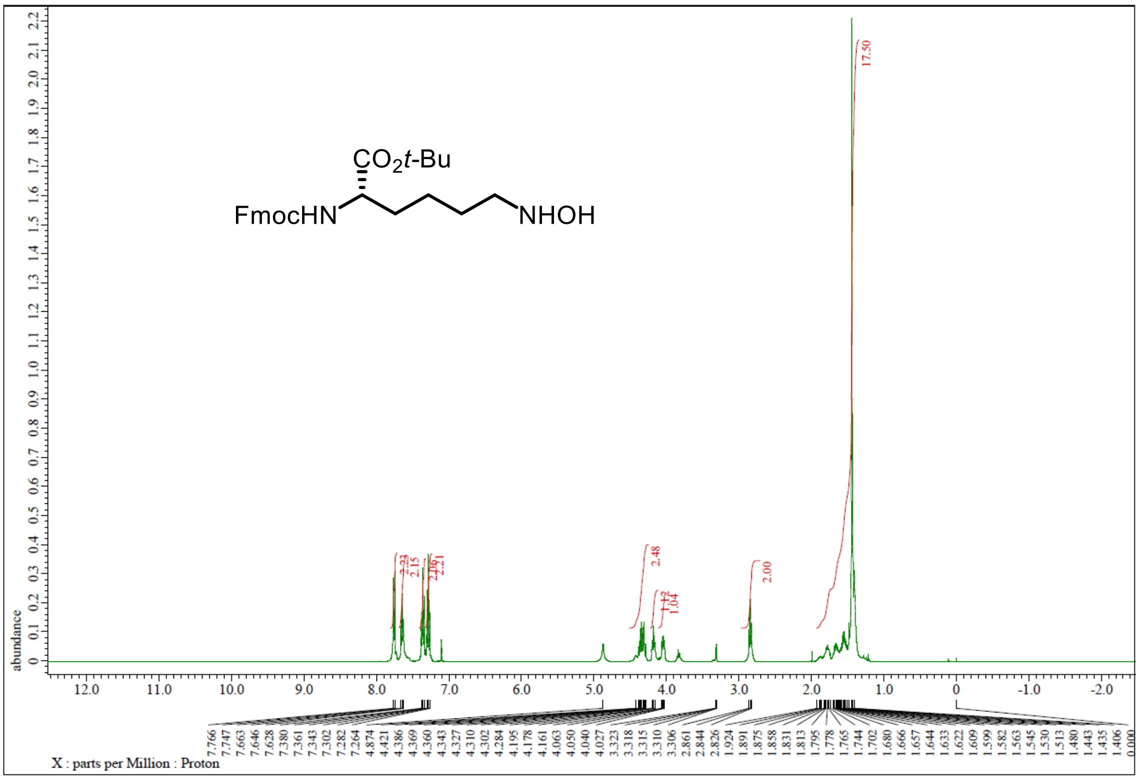

(^{13}C NMR, 100 MHz, $CDCl_3$)

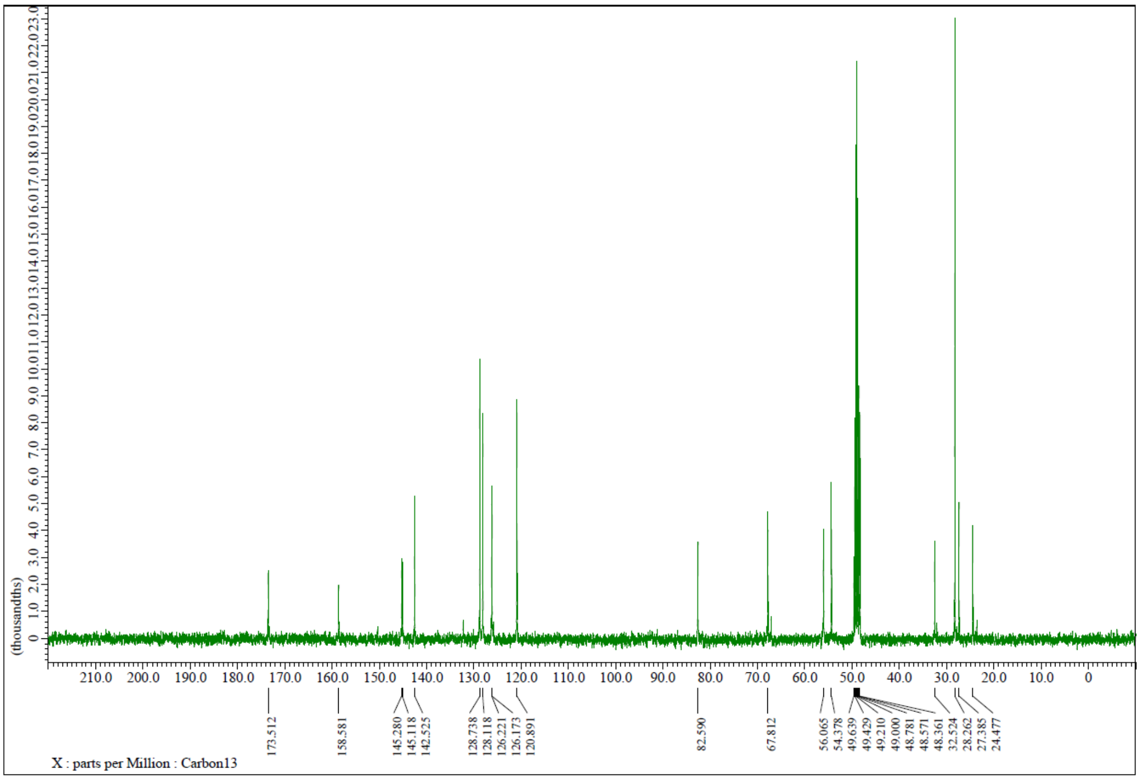

c,c,c,c-[5.5.5.5]-N,N′-Dibenzyl-2,8-dioxa-3,9-diazafenestrane (2a)

(^{1}H NMR, 400 MHz, $CDCl_3$)

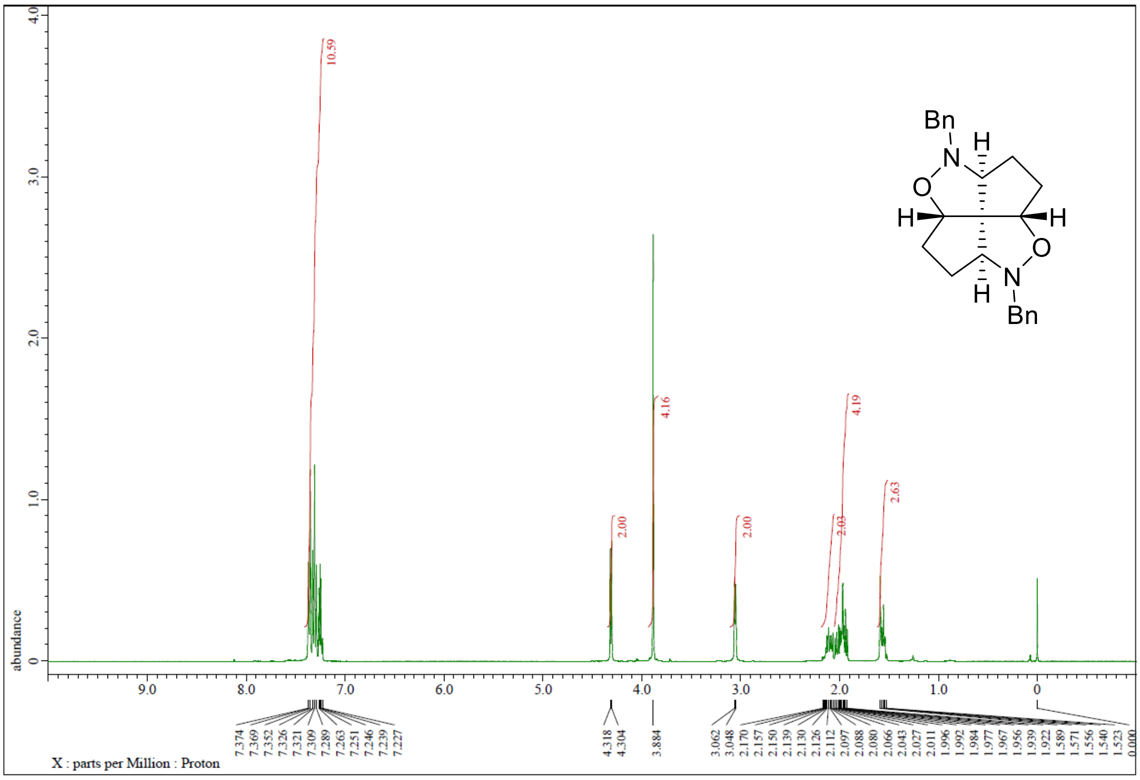

(^{13}C NMR, 100 MHz, $CDCl_3$)

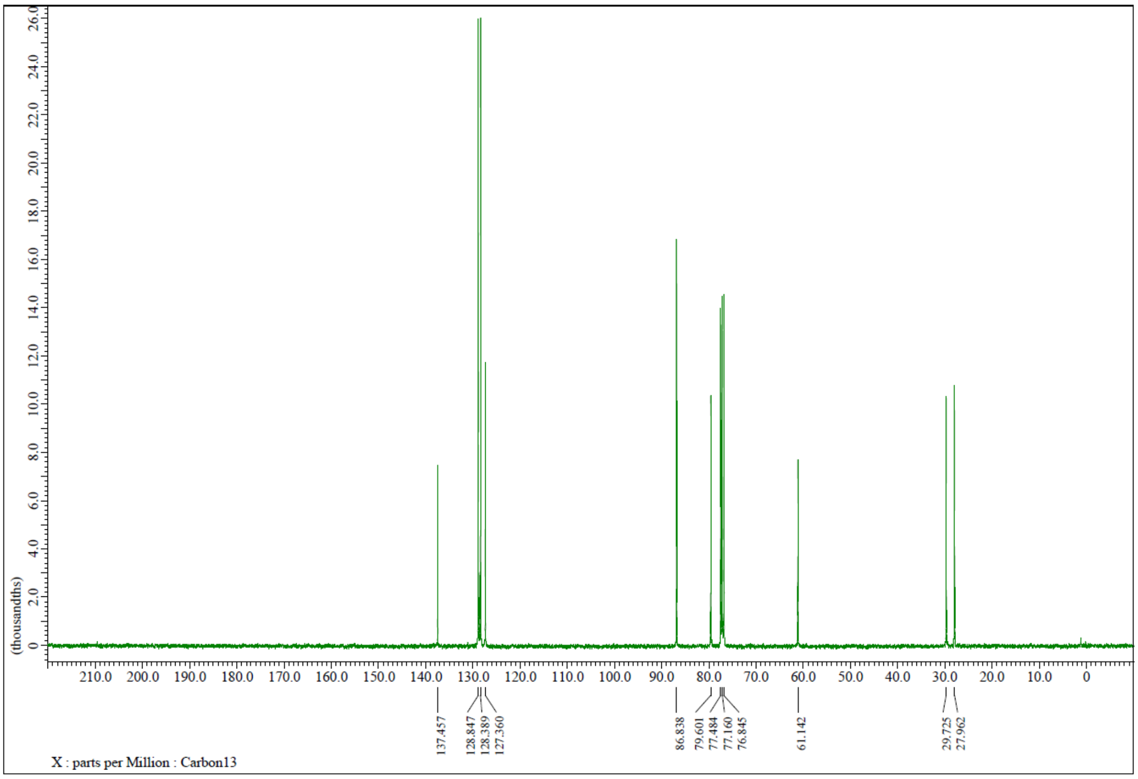

***c,c*-[5.5.5.5]-3,9-Dioxa-1,7-diiminofenestrane (5a)**

(^{1}H NMR, 400 MHz, $CDCl_3$)

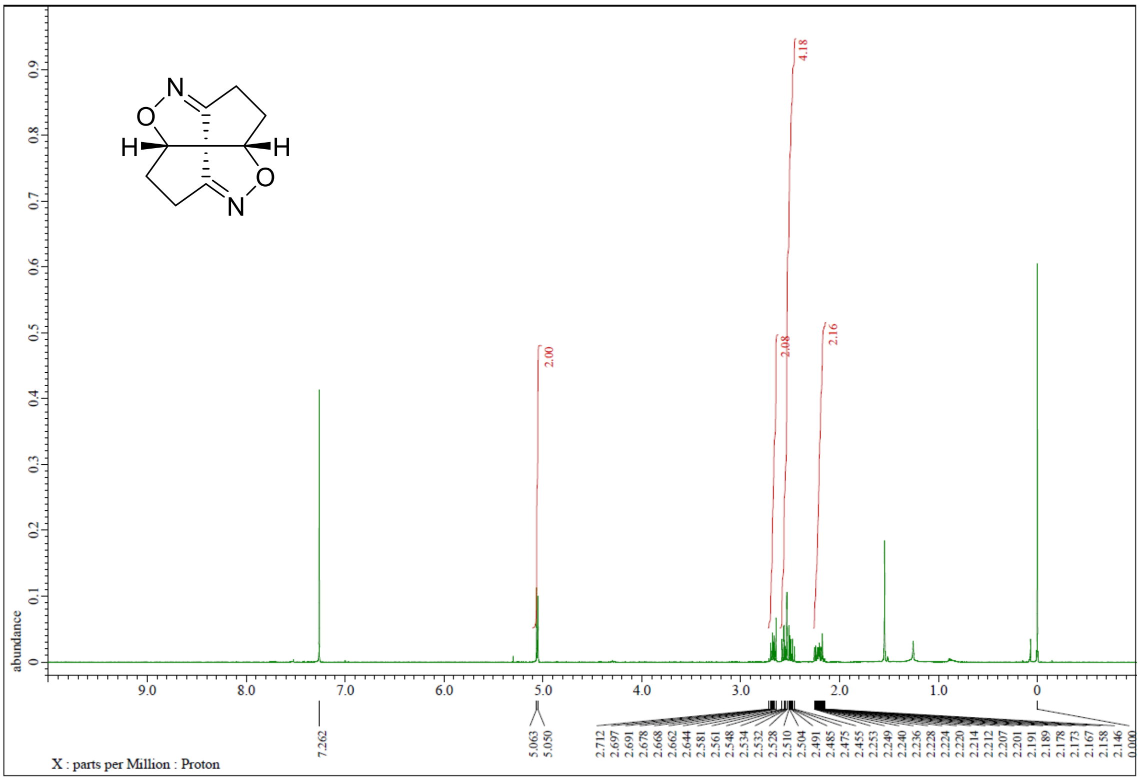

(^{13}C NMR, 100 MHz, $CDCl_3$)

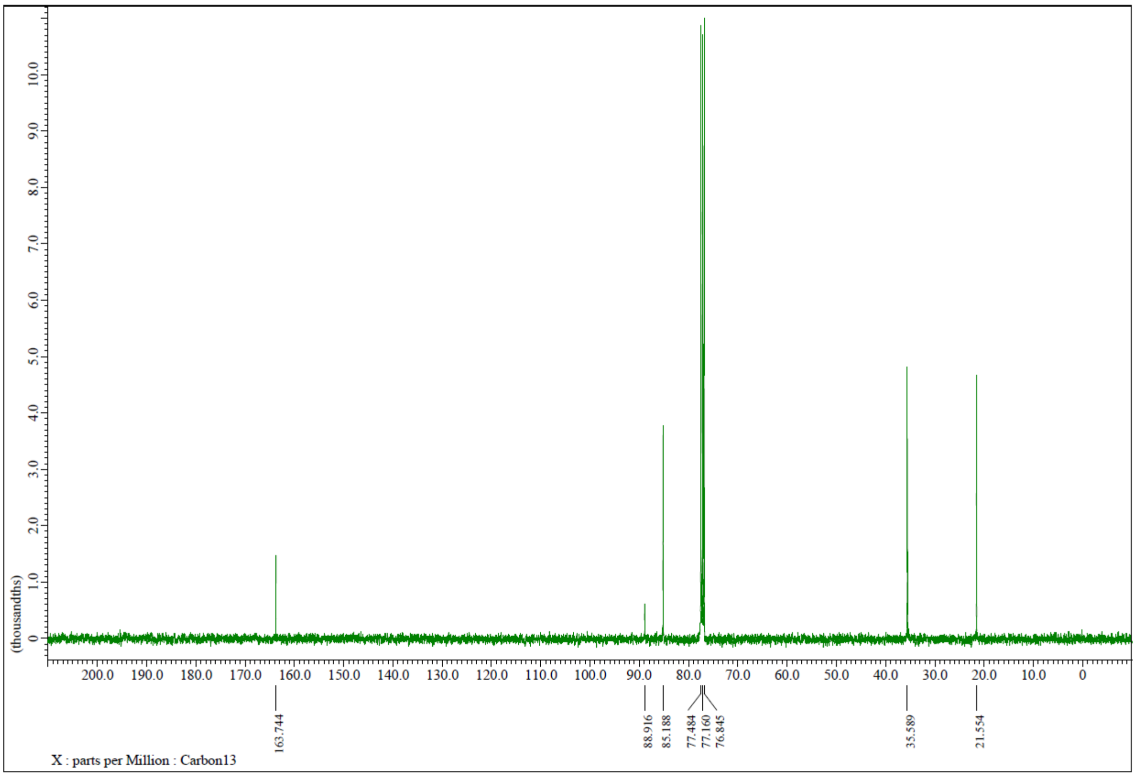

***c,c,c,c*-[5.5.5.5]-*N,N'*-Dimethyl-2,8-dioxa-3,9-diazafenestrane (2b)**

(^{1}H NMR, 400 MHz, $CDCl_3$)

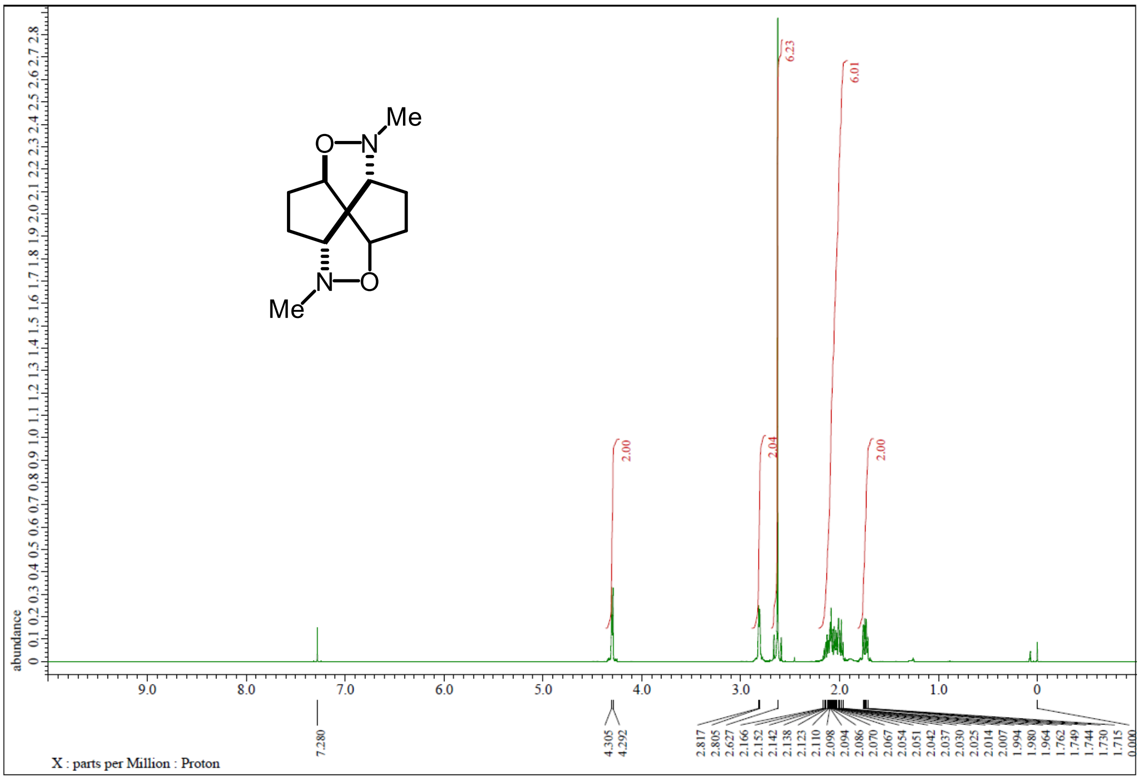

(^{13}C NMR, 100 MHz, $CDCl_3$)

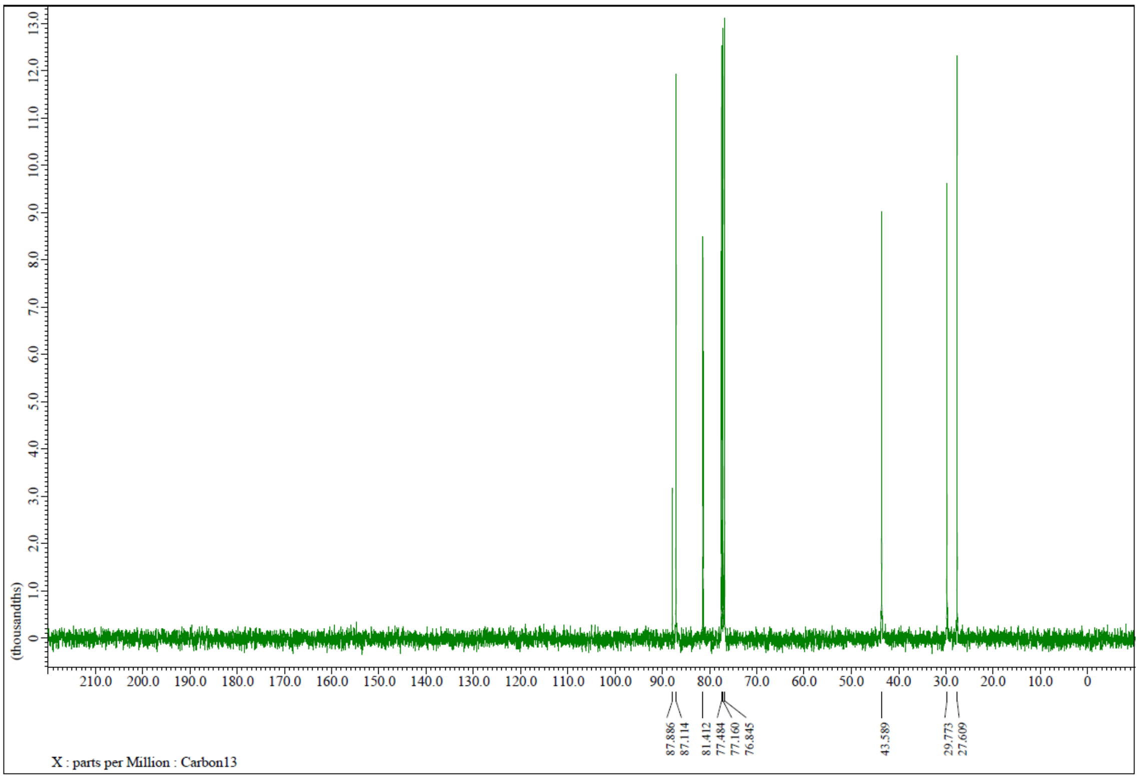

c,c,c,c-[5.5.5.5]-N,N'-Diisopropyl-2,8-dioxa-3,9-diazafenestrane (2c)

(^{1}H NMR, 400 MHz, $CDCl_3$)

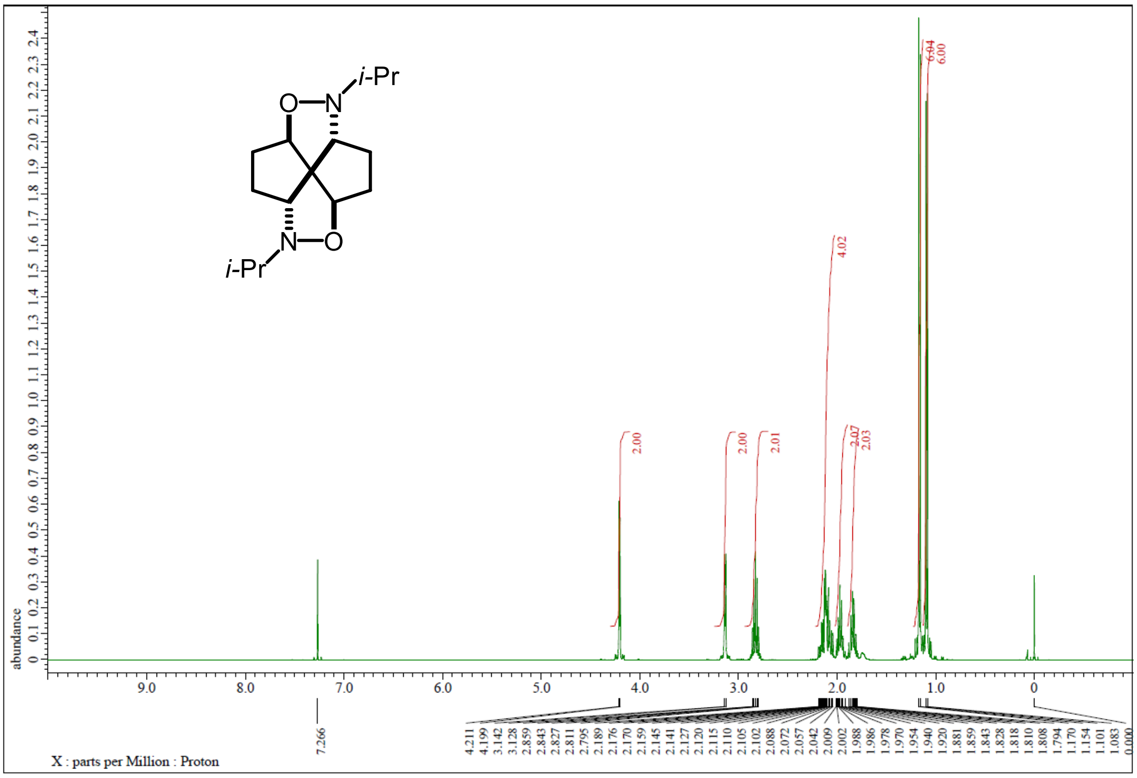

(^{13}C NMR, 100 MHz, $CDCl_3$)

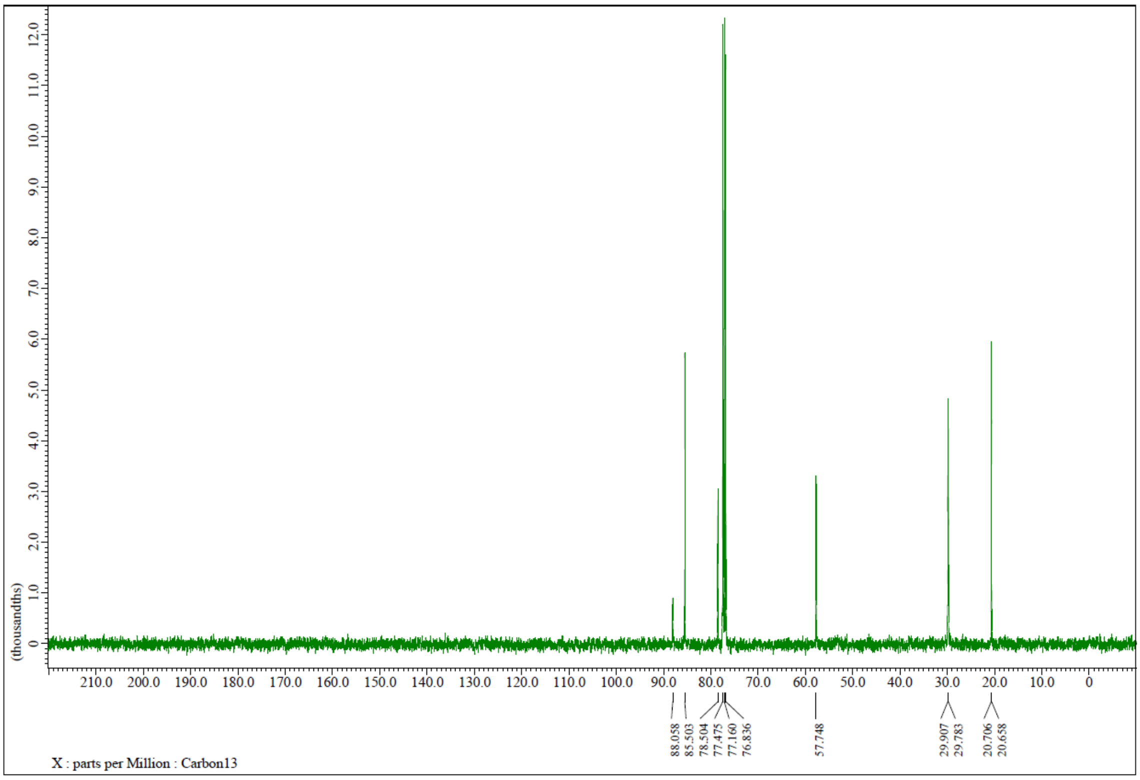

_c,c,c,c_-[5.5.5.5]-_N,N'_-Dicyclohexyl-2,8-dioxa-3,9-diazafenestrane (2d)

(^{1}H NMR, 400 MHz, $CDCl_3$)

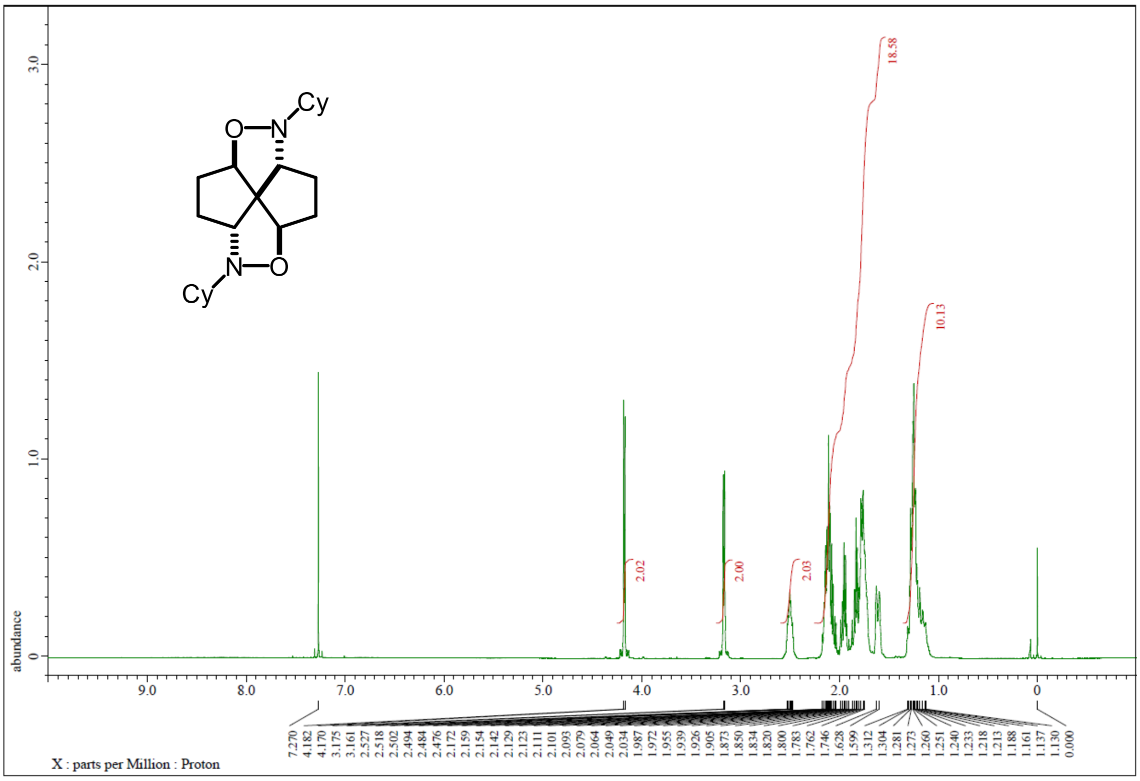

(^{13}C NMR, 100 MHz, $CDCl_3$)

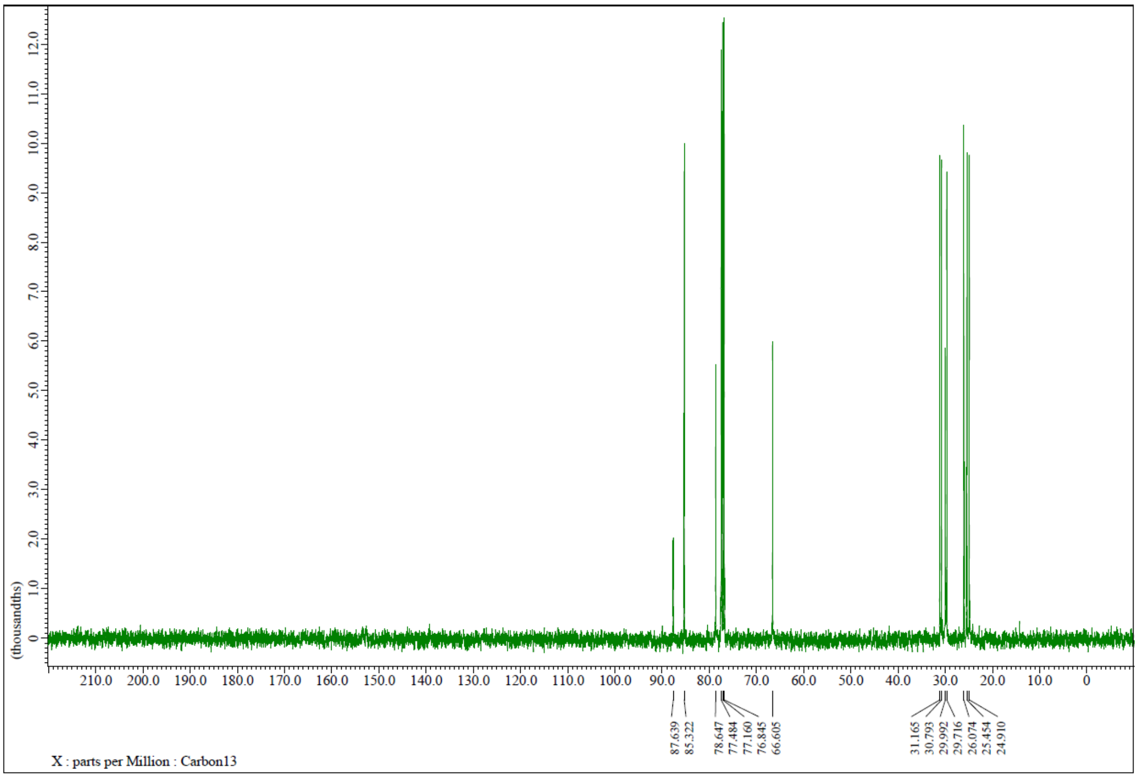

***c,c,c,c*-[5.5.5.5]-*N,N'*-Di(4-methoxybenzyl)-2,8-dioxa-3,9-diazafenestrane (2f)**

(^{1}H NMR, 400 MHz, $CDCl_3$)

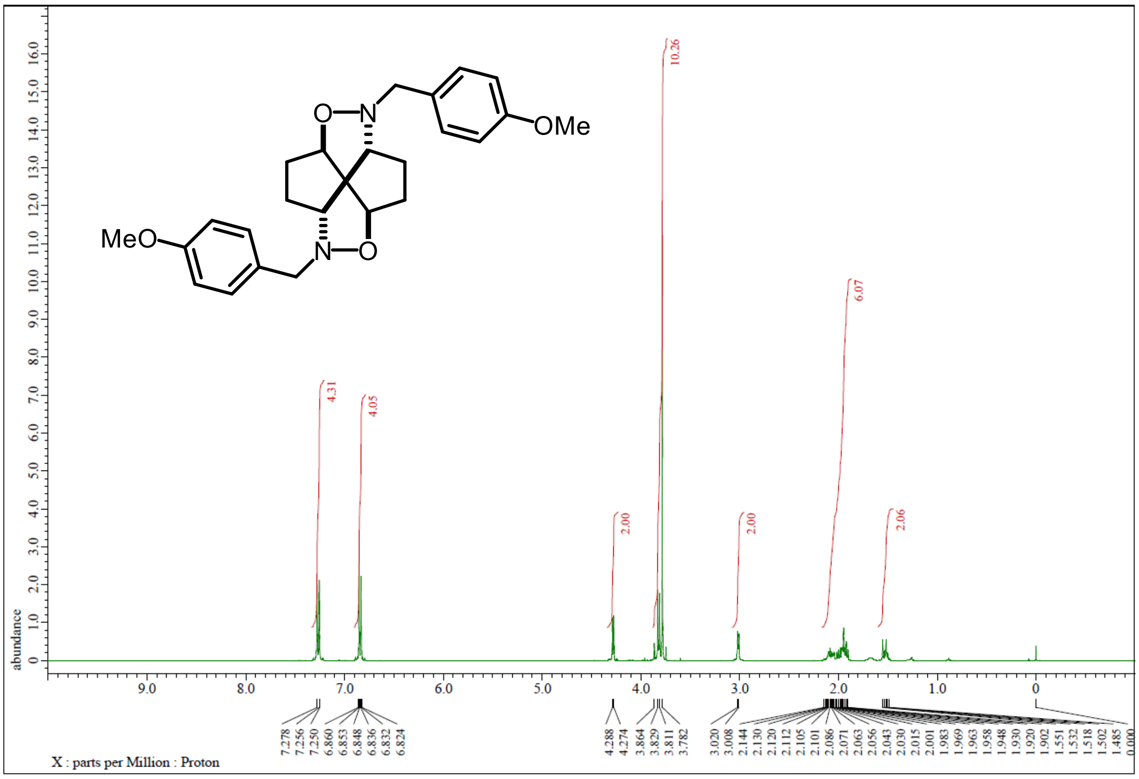

(^{13}C NMR, 100 MHz, $CDCl_3$)

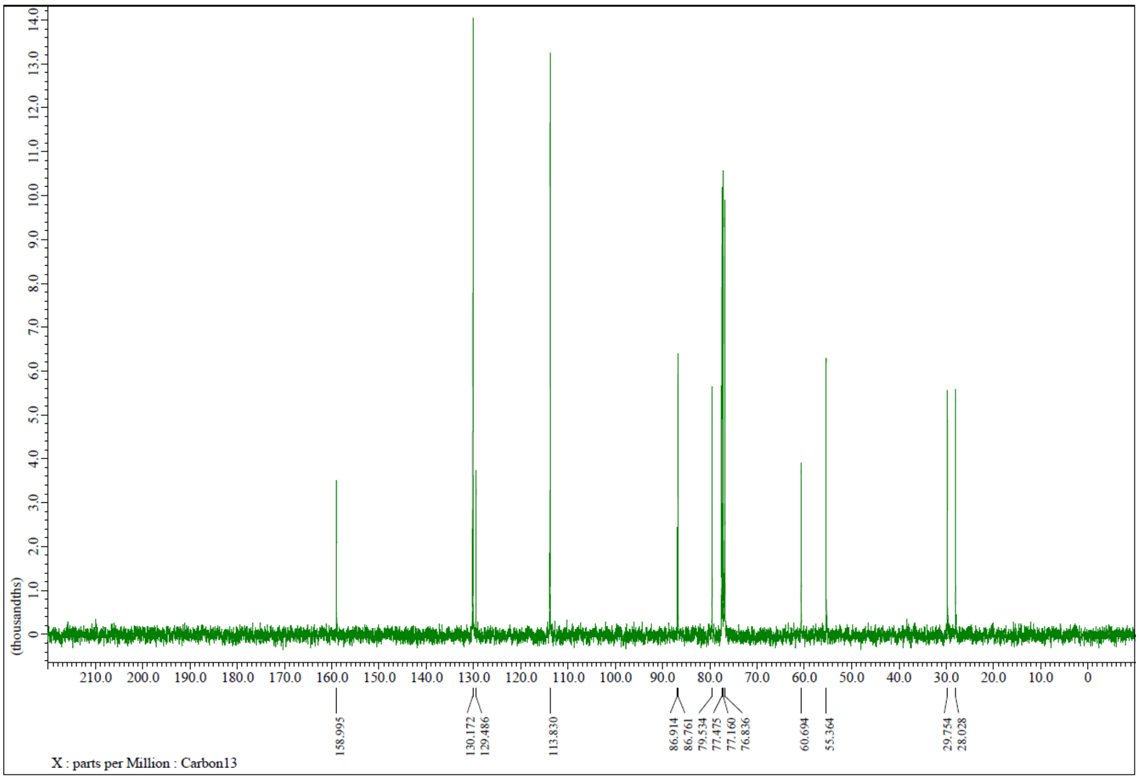

c,c,c,c-[5.5.5.5]-N,N'-Di(4-bromobenzyl)-2,8-dioxa-3,9-diazafenestrane (2g)

(^{1}H NMR, 400 MHz, $CDCl_3$)

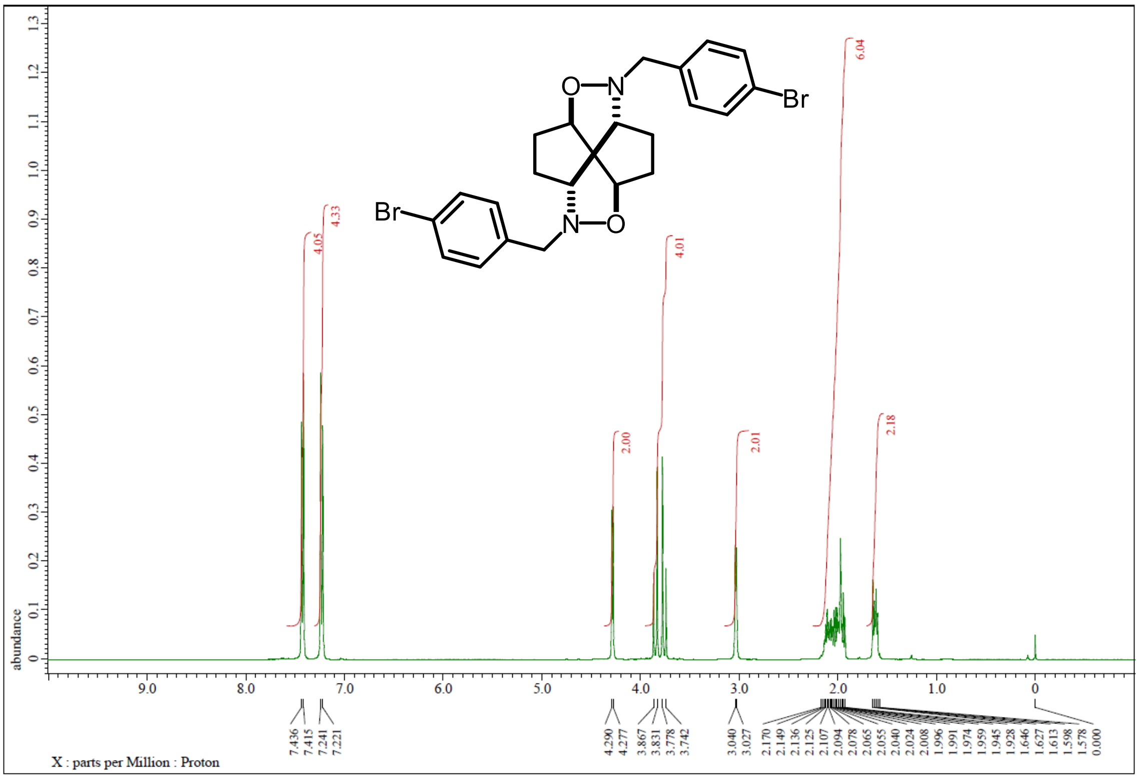

(^{13}C NMR, 100 MHz, $CDCl_3$)

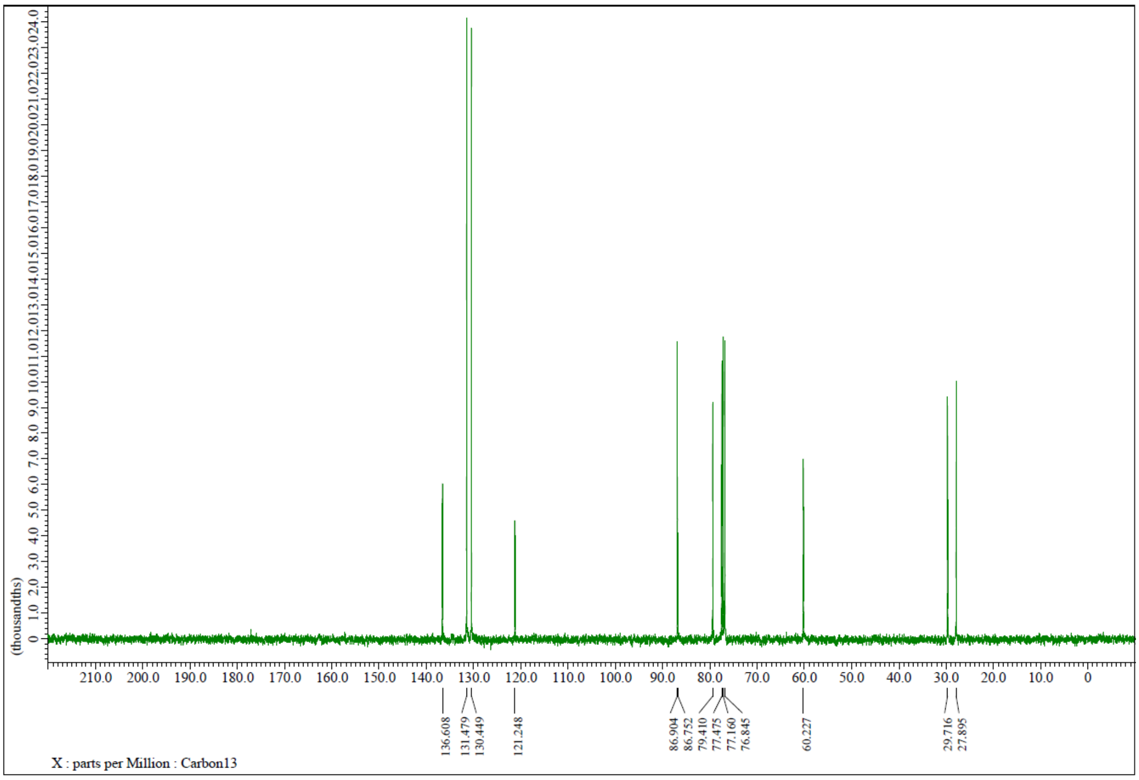

***c,c,c,c*-[5.5.5.5]-*N,N'*-Di(2-((*t*-butyldiphenylsilyl)oxy)ethyl)-2,8-dioxa-3,9-diazafenestrane (2h)**

(^{1}H NMR, 400 MHz, $CDCl_3$)

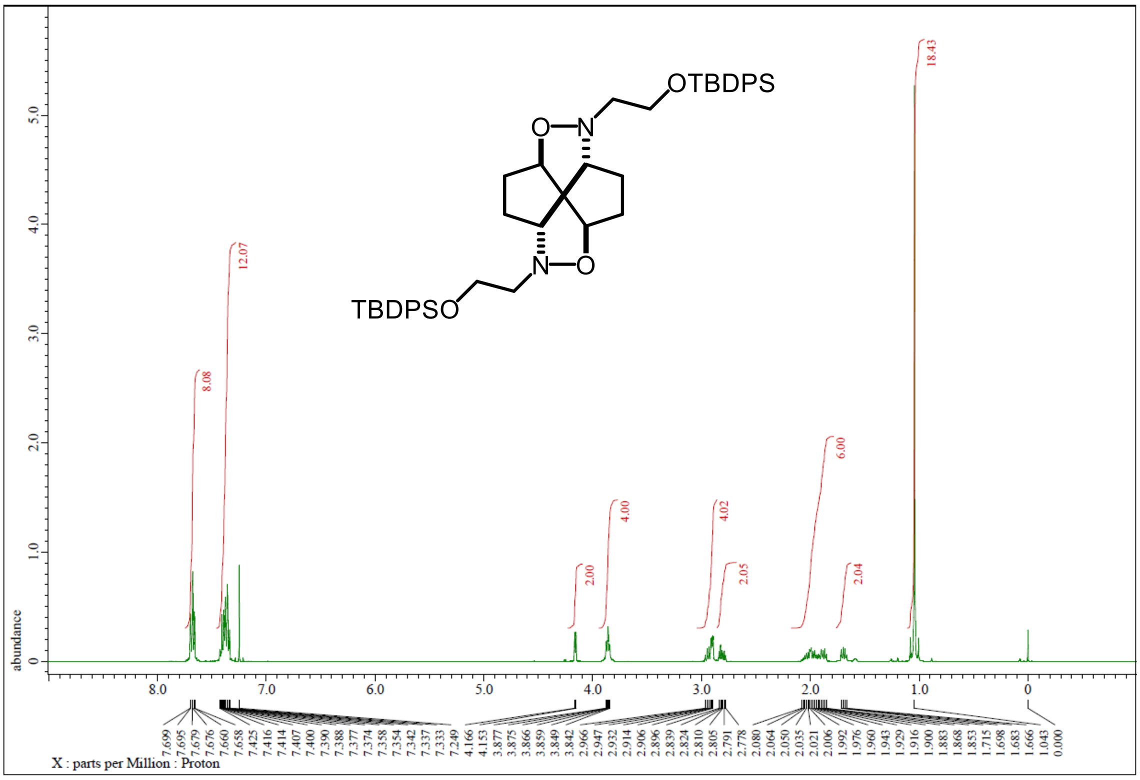

(^{13}C NMR, 100 MHz, $CDCl_3$)

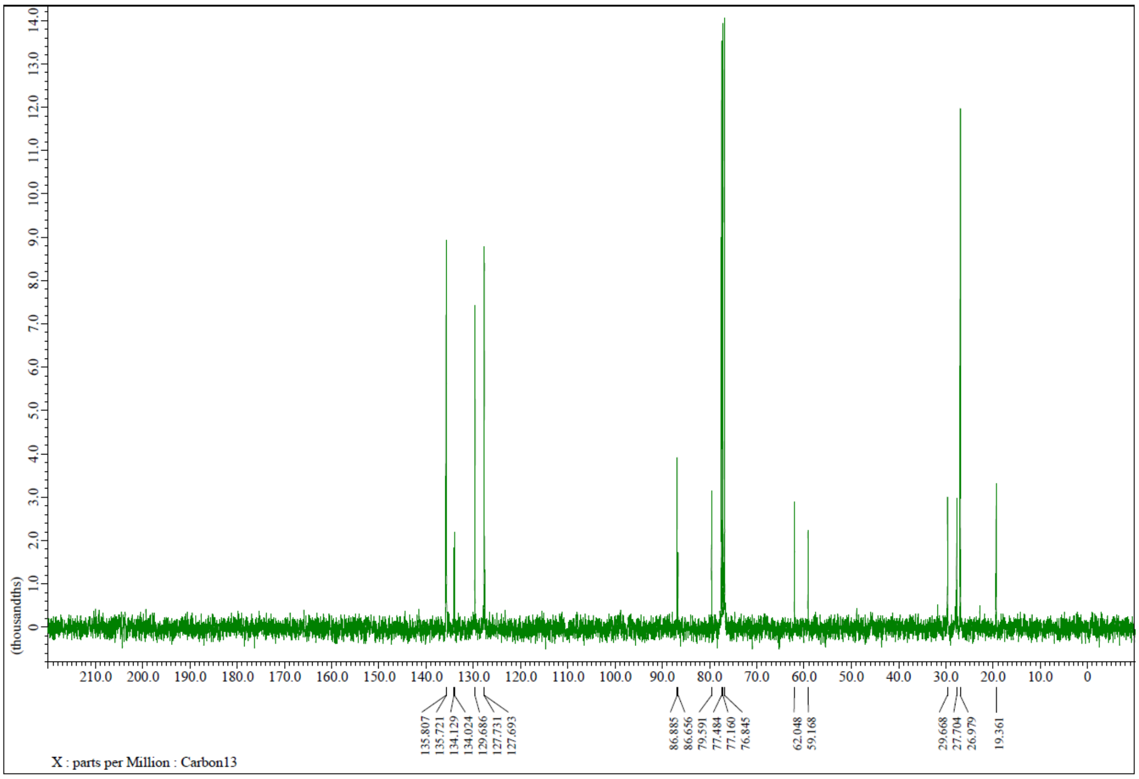

c,c,c,c-[5.5.5.5]-N,N'-Di(2-((tetrahydro-2H-pyran-2-yl)oxy)ethyl)-2,8-dioxa-3,9-diazafenestrane (2i)

(^{1}H NMR, 400 MHz, CDCl$_3$)

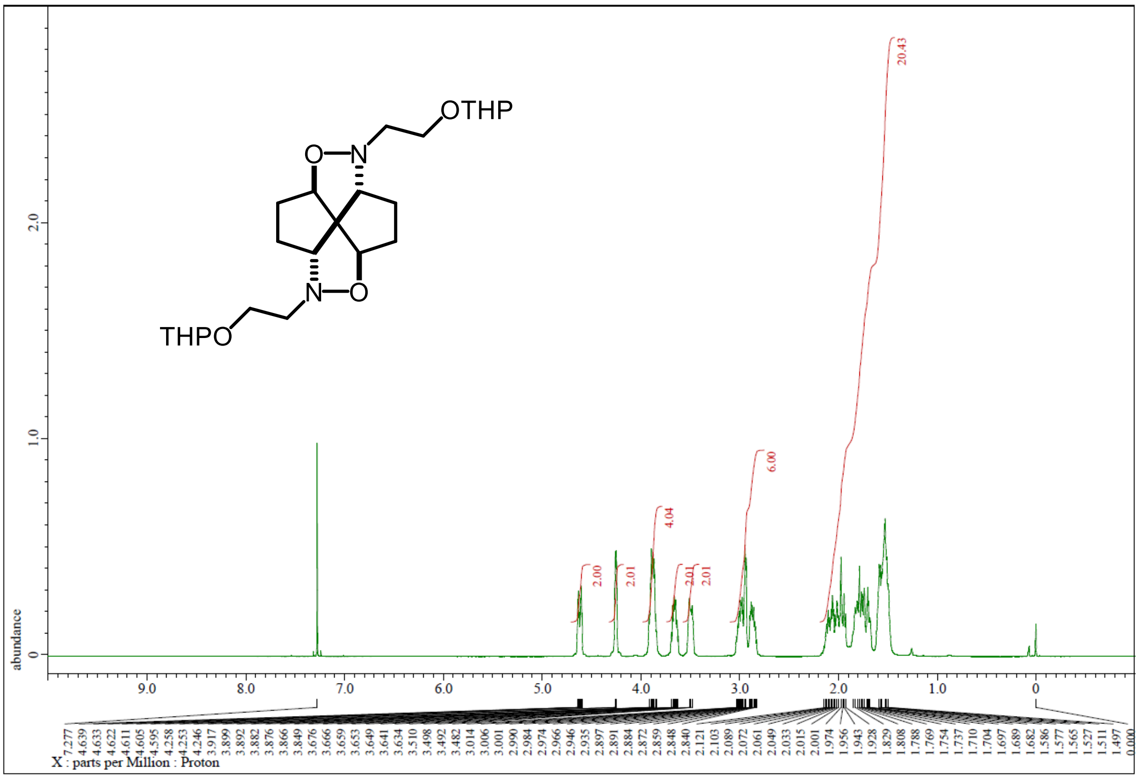

(^{13}C NMR, 100 MHz, CDCl$_3$)

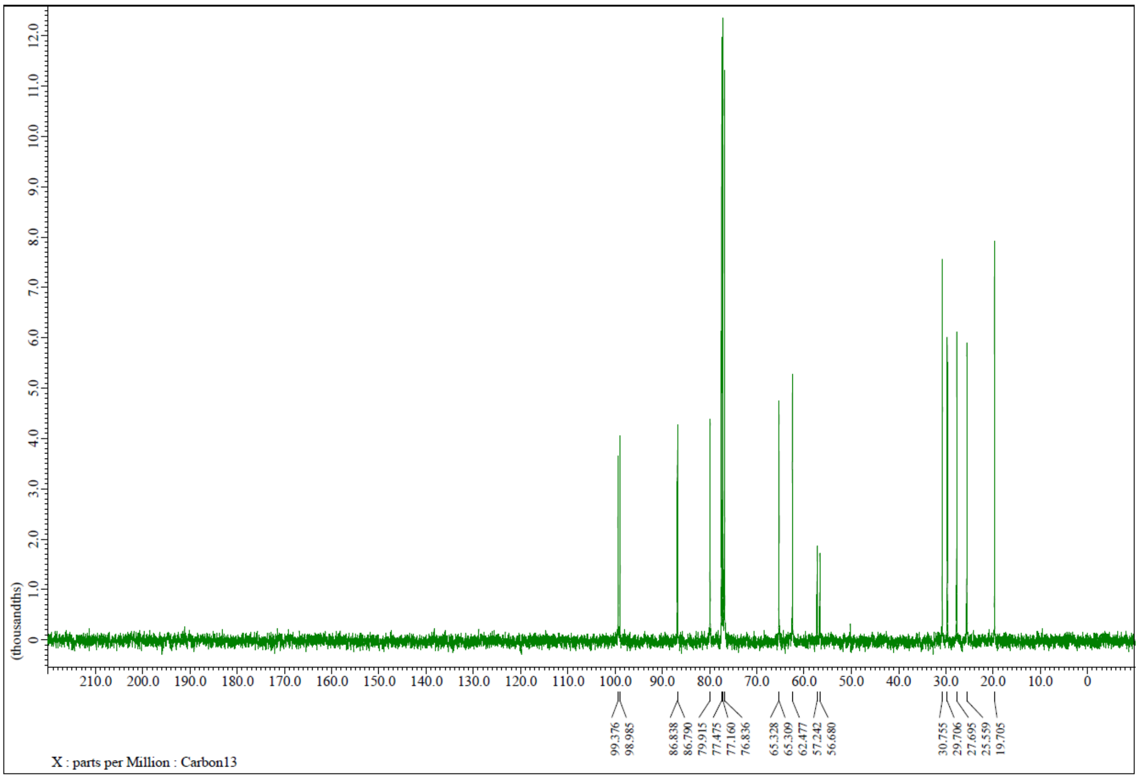

***c*,*c*,*c*,*c*-[5.5.5.5]-*N*,*N'*-Di(furan-3-ylmethyl)-2,8-dioxa-3,9-diazafenestrane (2j)**

(^{1}H NMR, 400 MHz, $CDCl_3$)

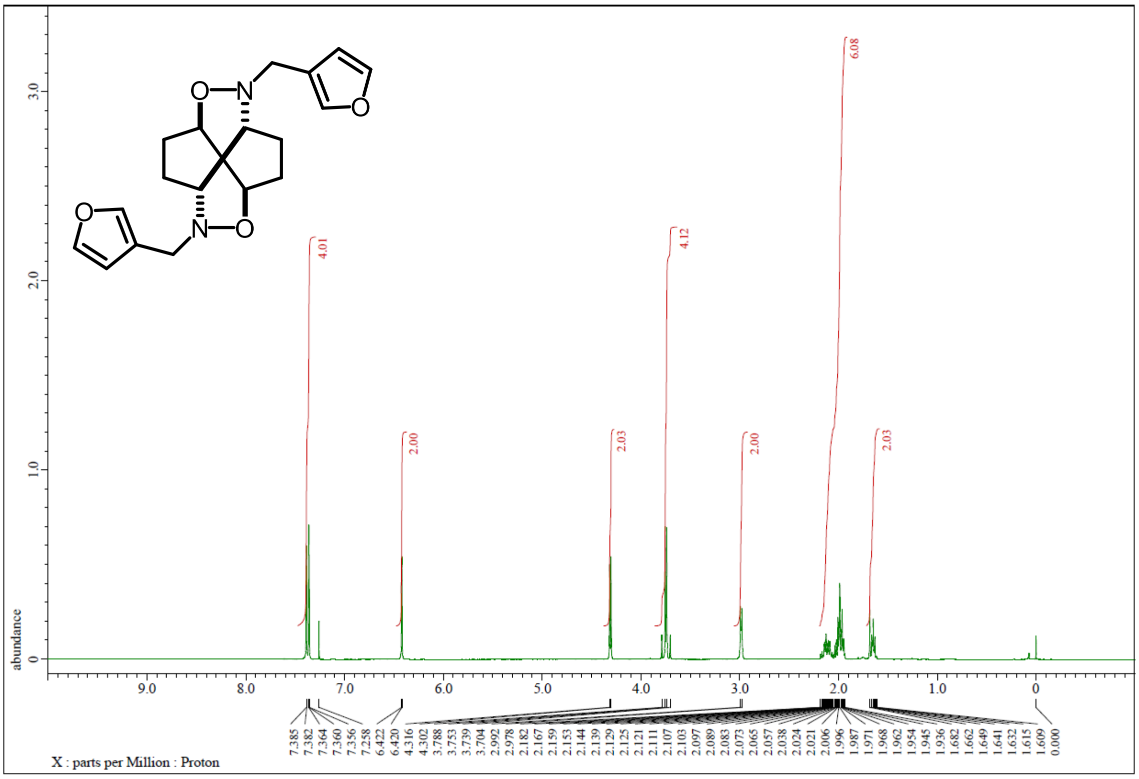

(^{13}C NMR, 100 MHz, $CDCl_3$)

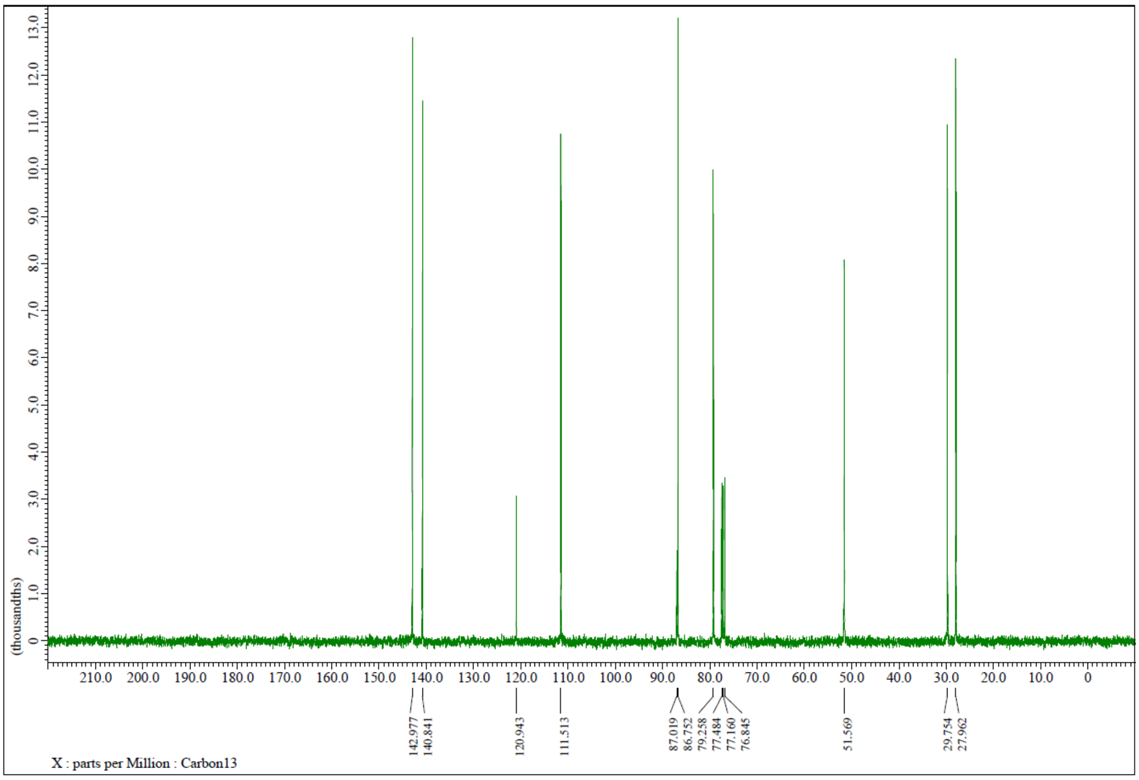

***c,c,c,c*-[5.5.5.5]-*N,N'*-Di((5*S*)-5-((*t*-butoxycarbonyl)amino)-5-(methoxycarbonyl)pentyl)-2,8-dioxa-3,9-diazafenestrane (2k)**

(^{1}H NMR, 400 MHz, $CDCl_3$)

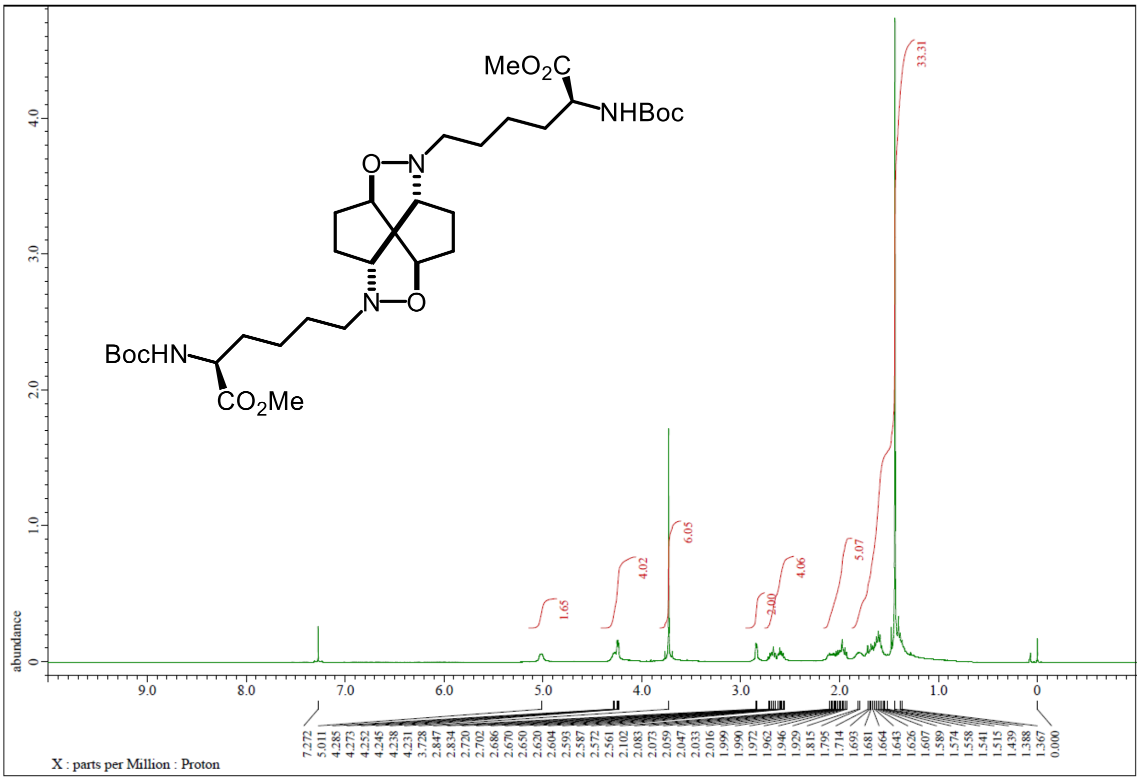

(^{13}C NMR, 100 MHz, $CDCl_3$)

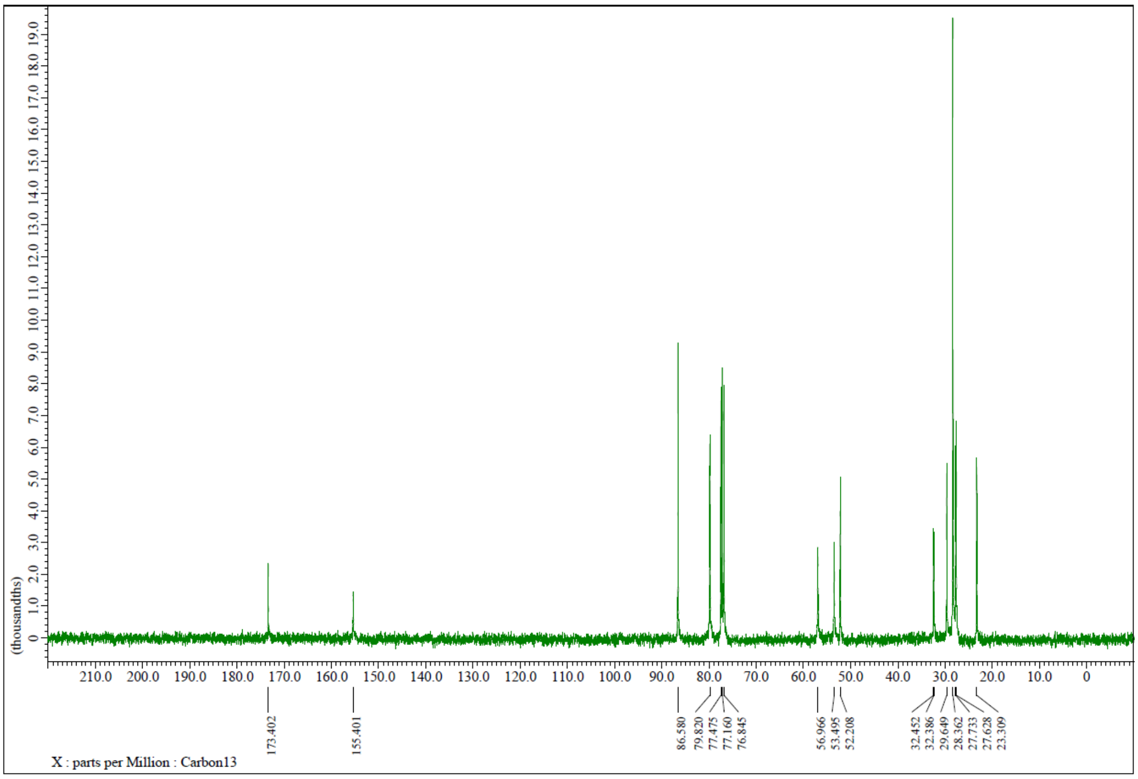

_c,c,c,c_-[5.5.5.5]-_N,N'_-Di((5_S_)-5-(_t_-butoxycarbonyl)-5-((9-fluorenylmethyloxycarbonyl)amino)pentyl)-2,8-dioxa-3,9-diazafenestrane (2l)

(^{1}H NMR, 400 MHz, $CDCl_3$)

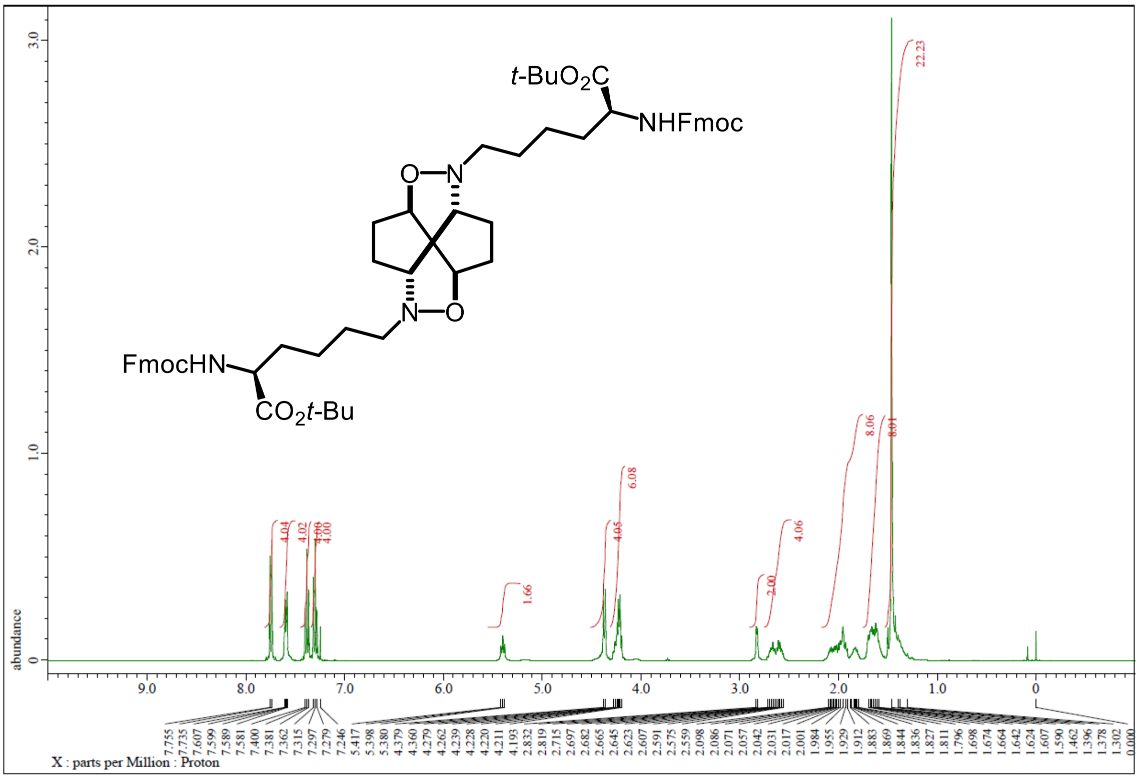

(^{13}C NMR, 100 MHz, $CDCl_3$)

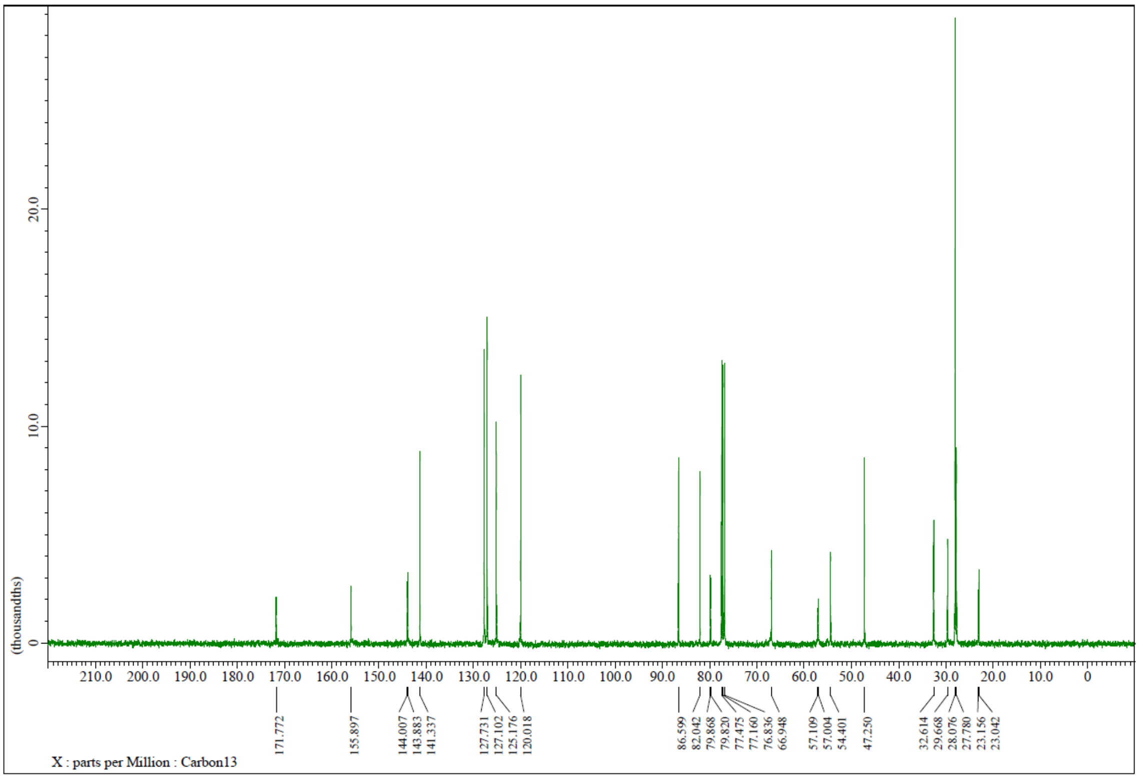

***c,c,c,c*-[5.5.5.5]-*N,N'*-Diphenyl-2,8-dioxa-3,9-diazafenestrane (2m)**

(^{1}H NMR, 400 MHz, $CDCl_3$)

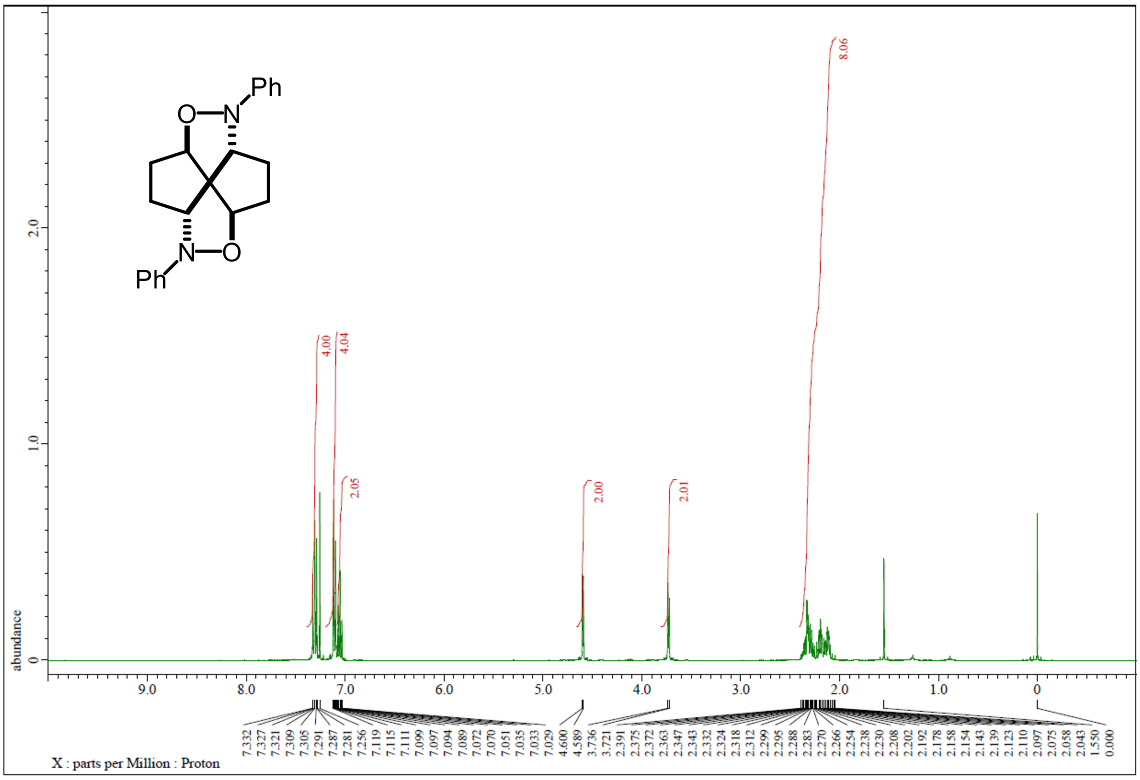

(^{13}C NMR, 100 MHz, $CDCl_3$)

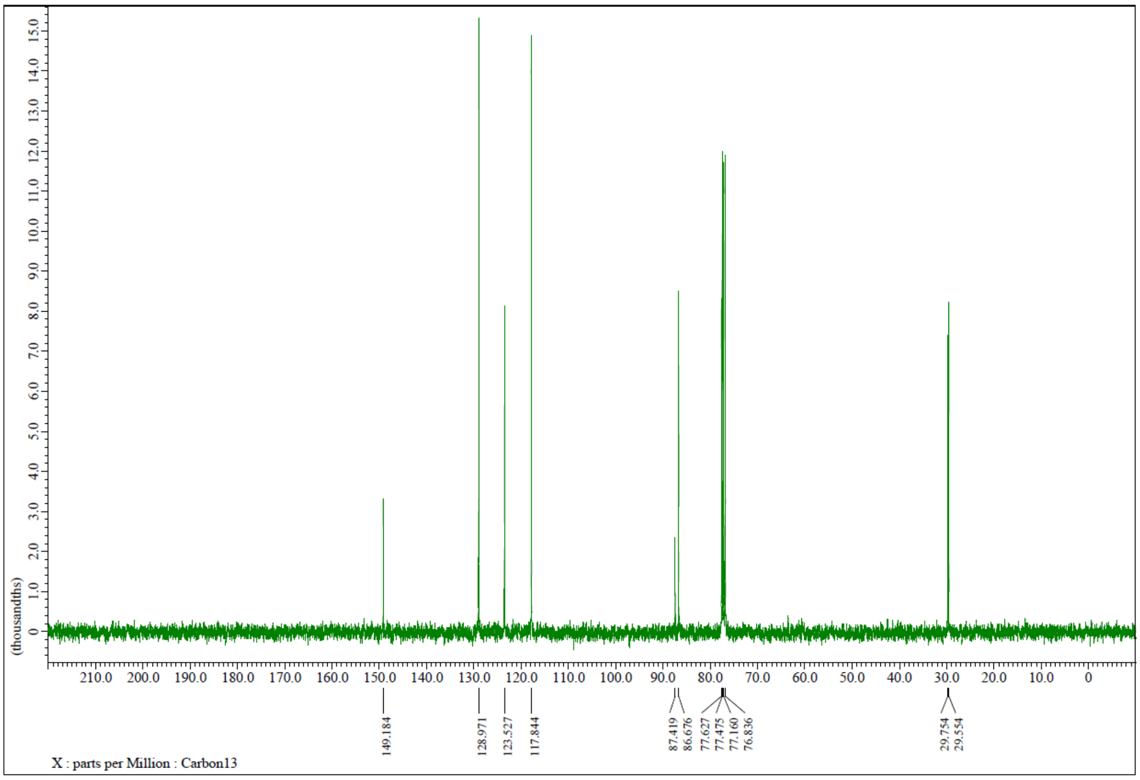

c,c,c,c-[5.5.5.5]-5R-N,N′-Dibenzyl-5-hexyl-2,8-dioxa-3,9-diazafenestrane (2n)

(^{1}H NMR, 400 MHz, $CDCl_3$)

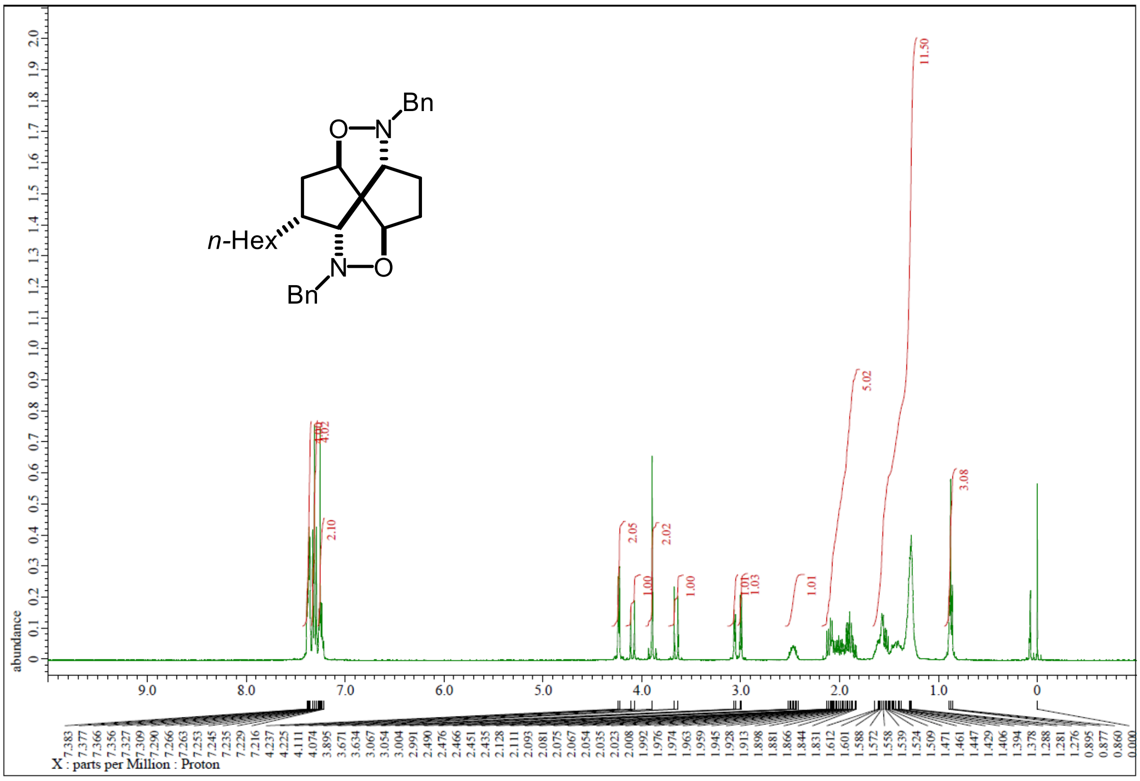

(^{13}C NMR, 100 MHz, $CDCl_3$)

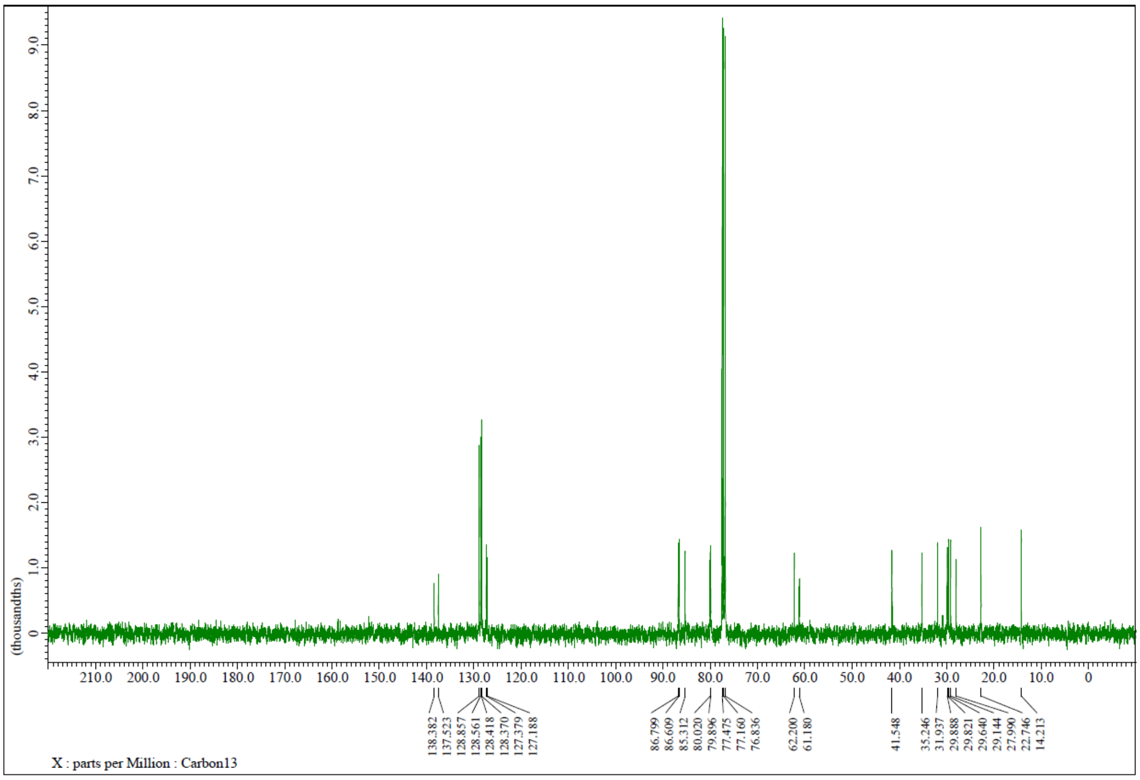

(COSY, 400 MHz, $CDCl_3$)

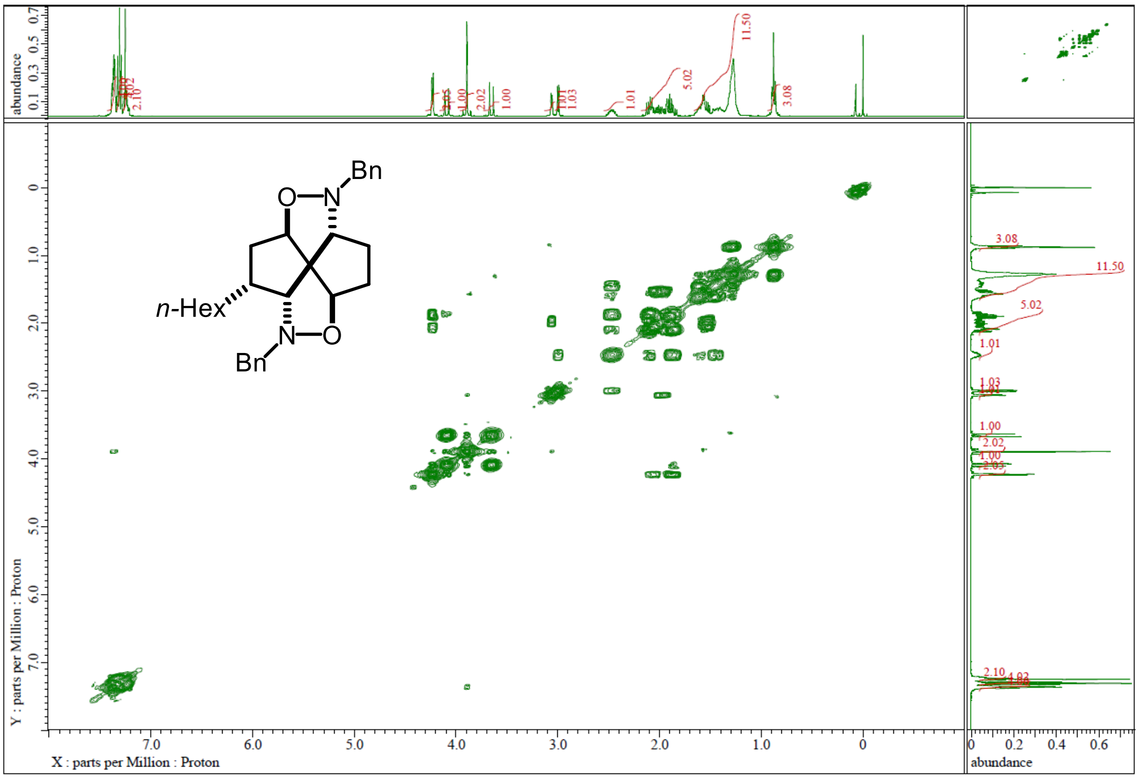

(NOESY, 400 MHz, $CDCl_3$)

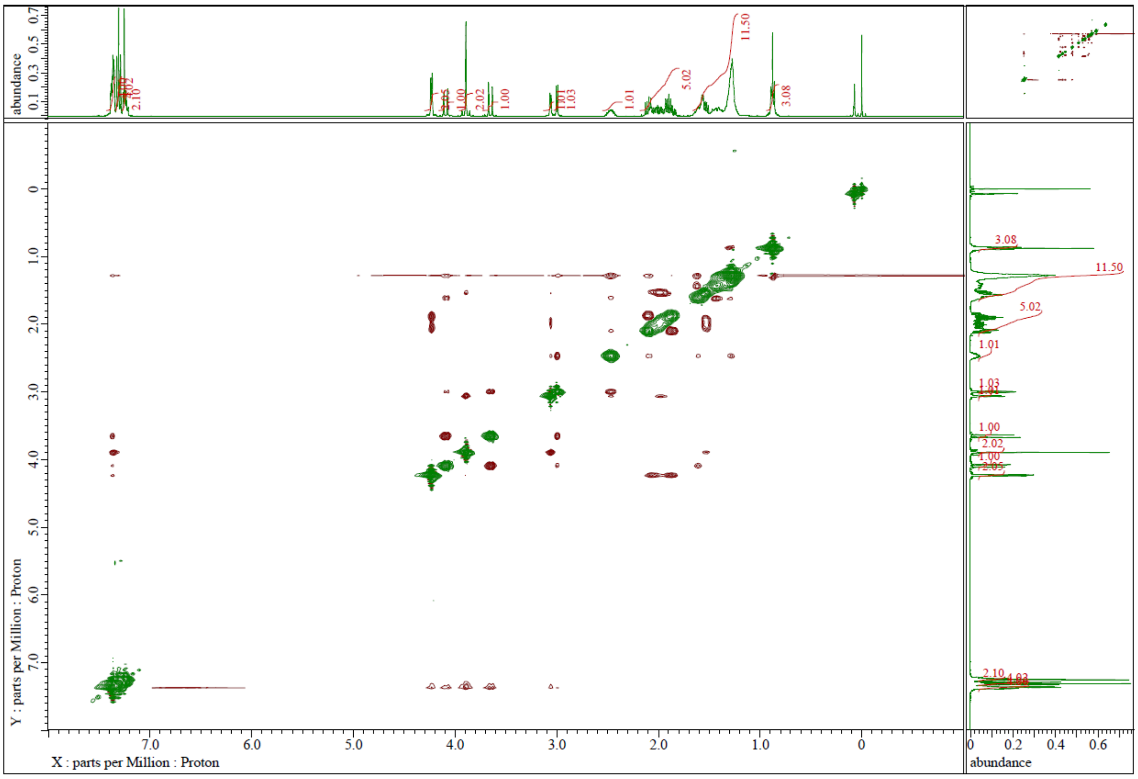

c,c,c,c-[5.5.5.5]-5S-N,N'-Dibenzyl-5-hexyl-2,8-dioxa-3,9-diazafenestrane (2n')

(^{1}H NMR, 400 MHz, $CDCl_3$)

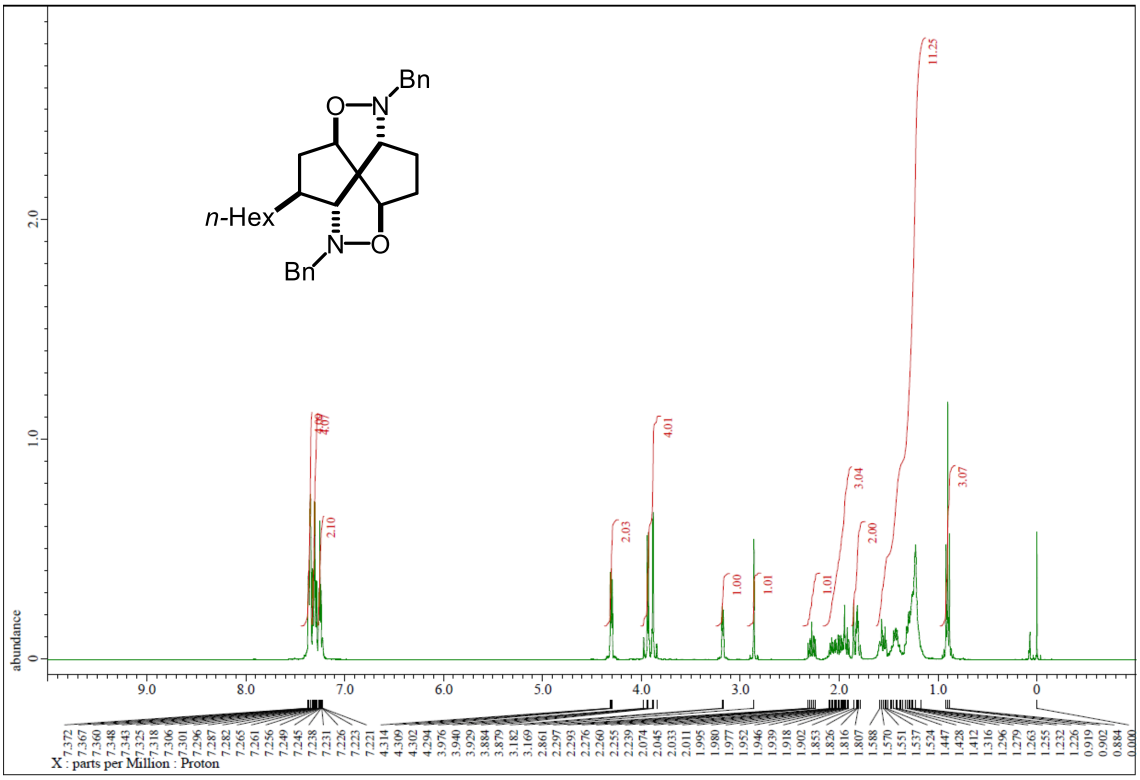

(^{13}C NMR, 100 MHz, $CDCl_3$)

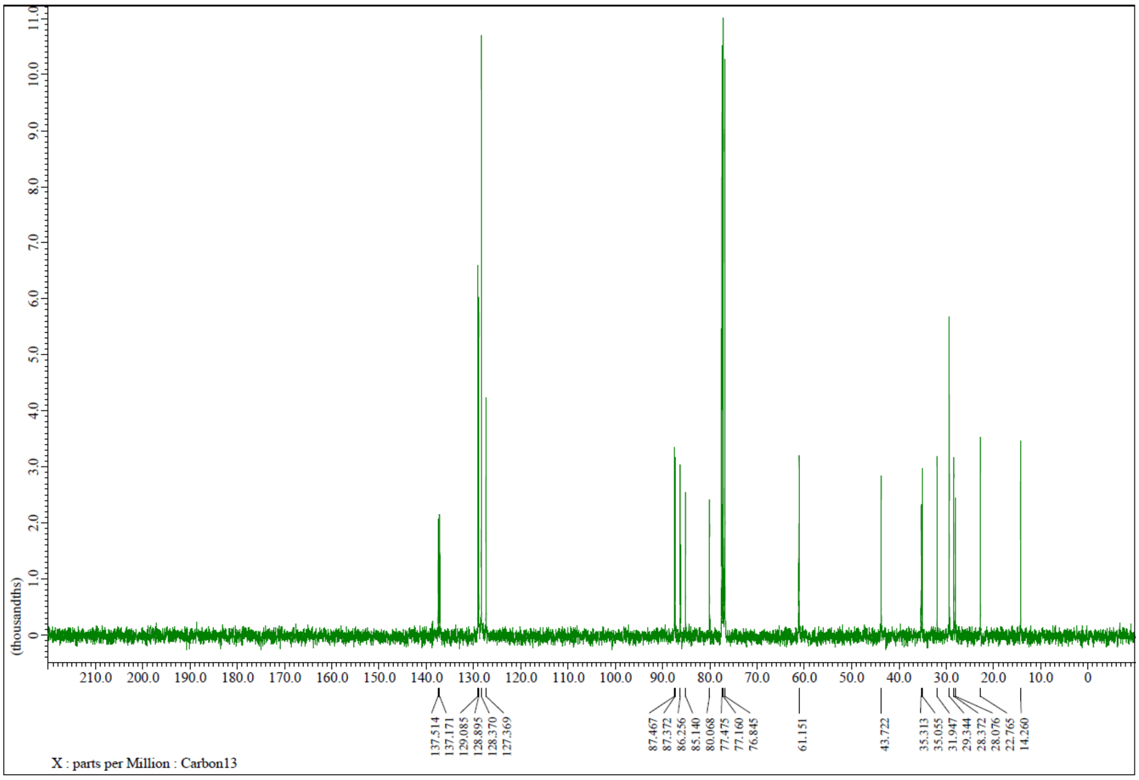

(COSY, 400 MHz, $CDCl_3$)

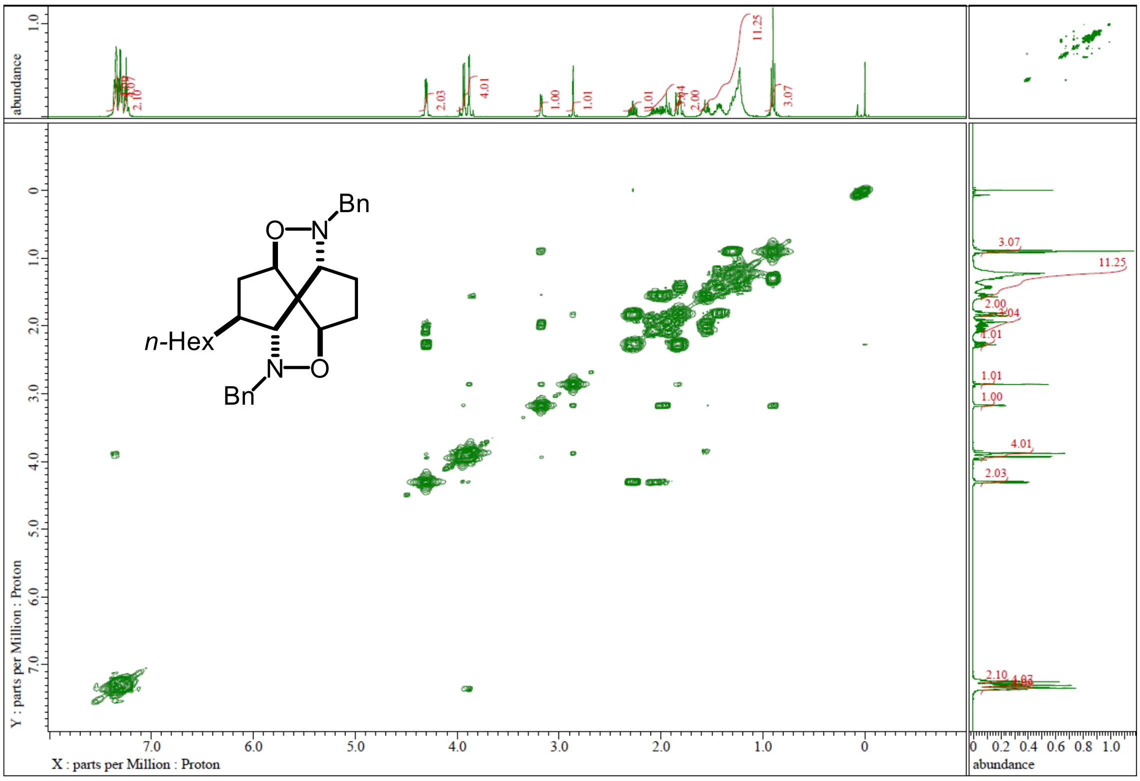

(NOESY, 400 MHz, $CDCl_3$)

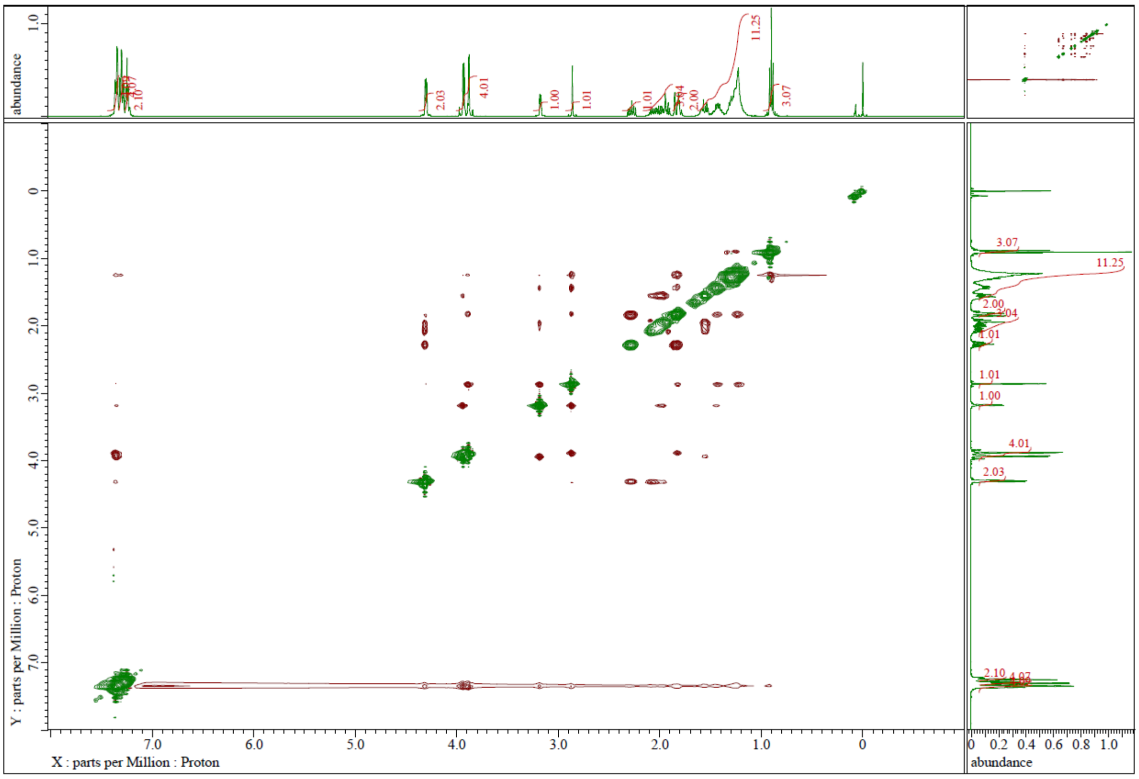

***c,c,c,c*-[5.5.5.5]-*N,N′*-Dimethyl-5-hexyl-2,8-dioxa-3,9-diazafenestrane (2o)**

(^{1}H NMR, 400 MHz, CDCl$_3$)

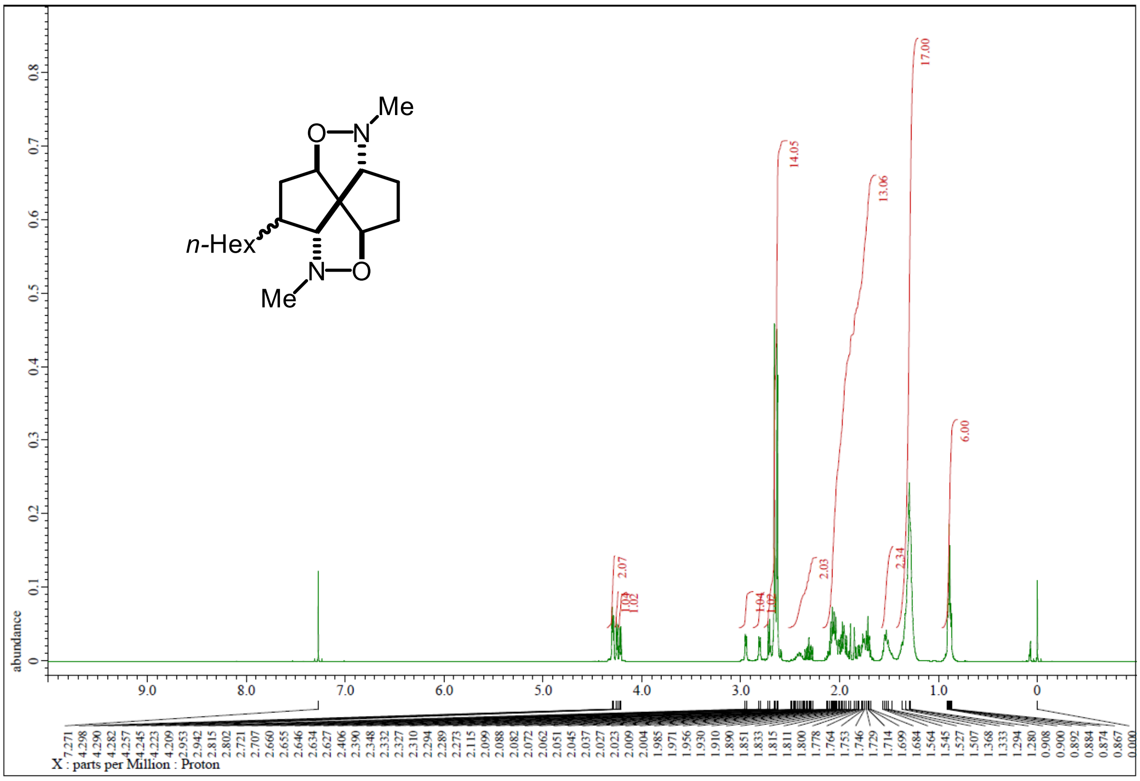

(^{13}C NMR, 100 MHz, CDCl$_3$)

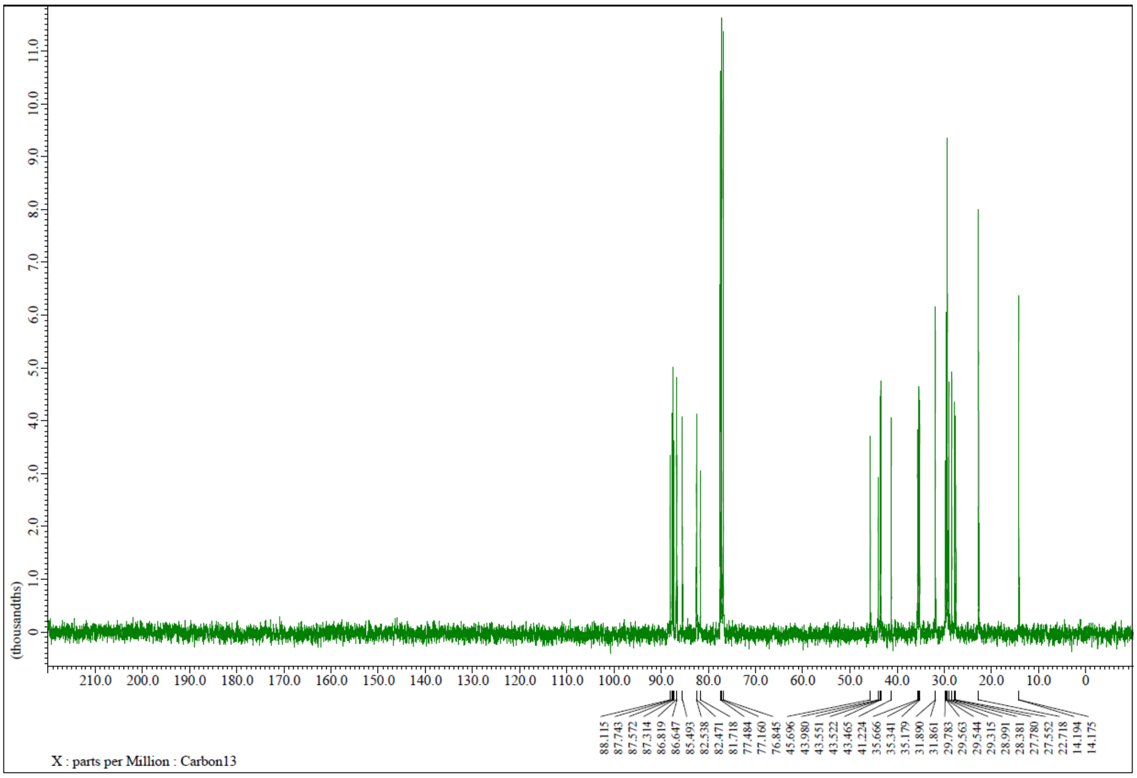

***c,c,c,c*-[5.5.5.5]-5*R*-*N*,*N′*-Dibenzyl-5-benzyloxyl-2,8-dioxa-3,9-diazafenestrane (2p)**

(^{1}H NMR, 400 MHz, $CDCl_3$)

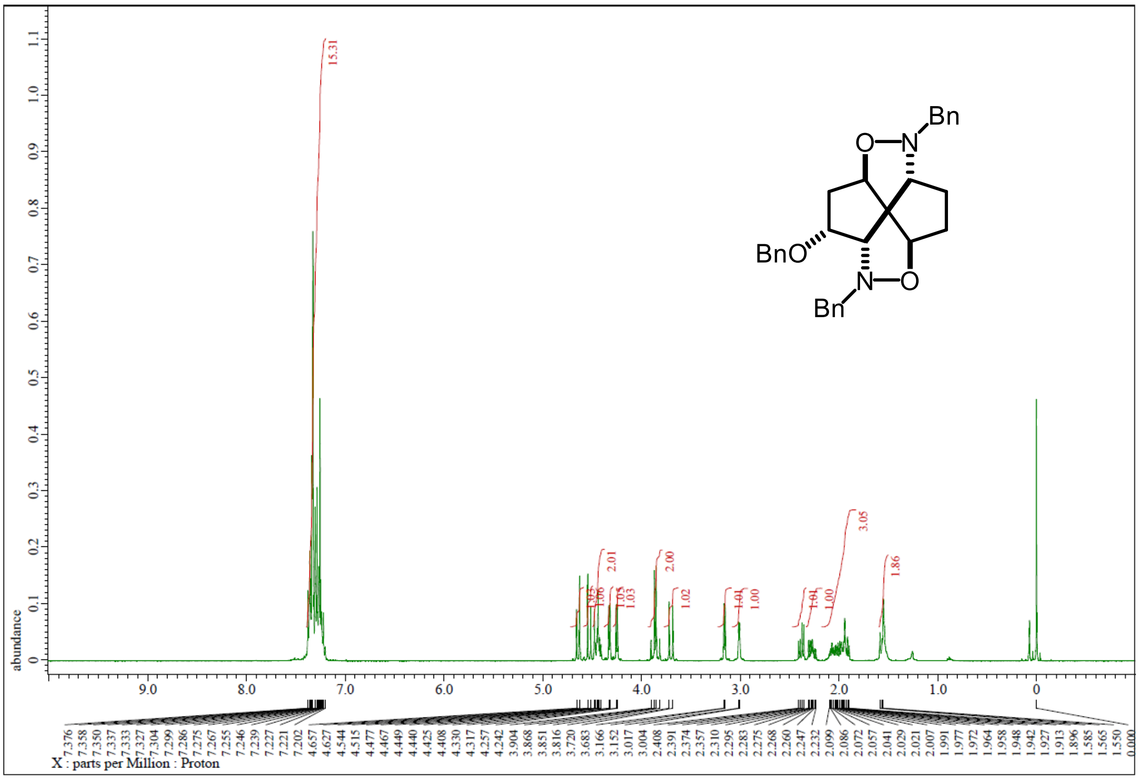

(^{13}C NMR, 100 MHz, $CDCl_3$)

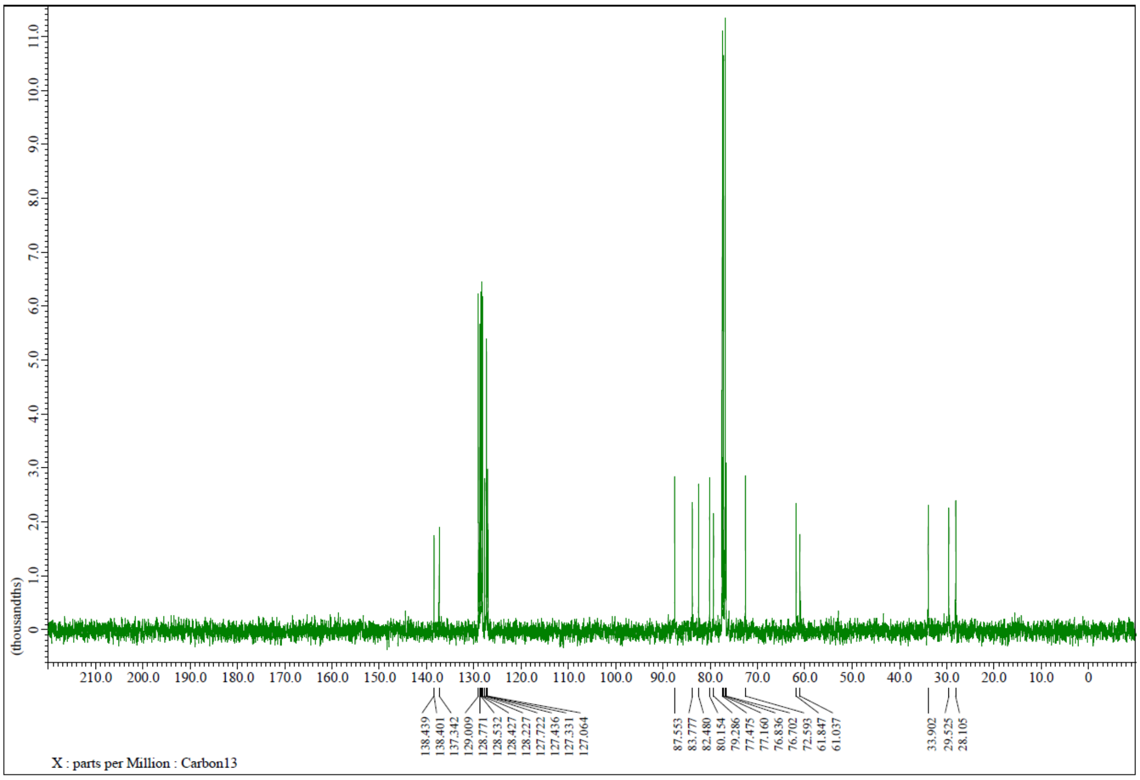

(COSY, 400 MHz, $CDCl_3$)

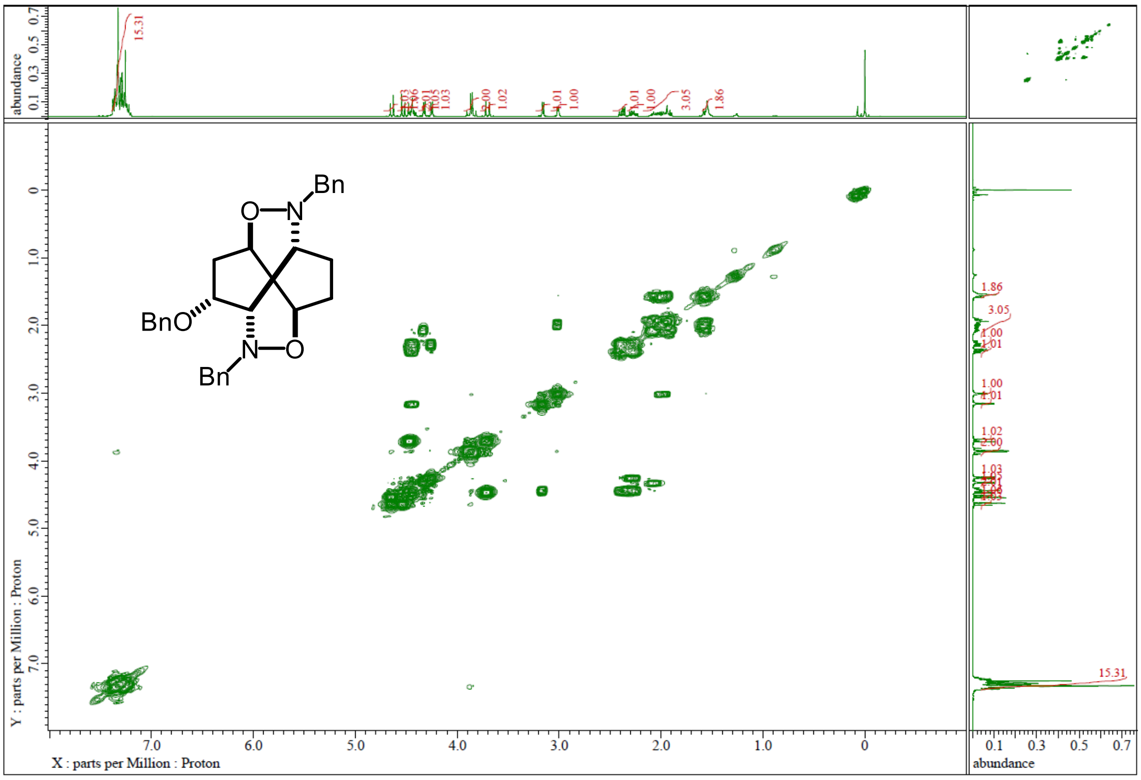

c,c,c,c-[5.5.5.5]-5S-N,N'-Dibenzyl-5-benzyloxyl-2,8-dioxa-3,9-diazafenestrane (2p')

(^1H NMR, 400 MHz, $CDCl_3$)

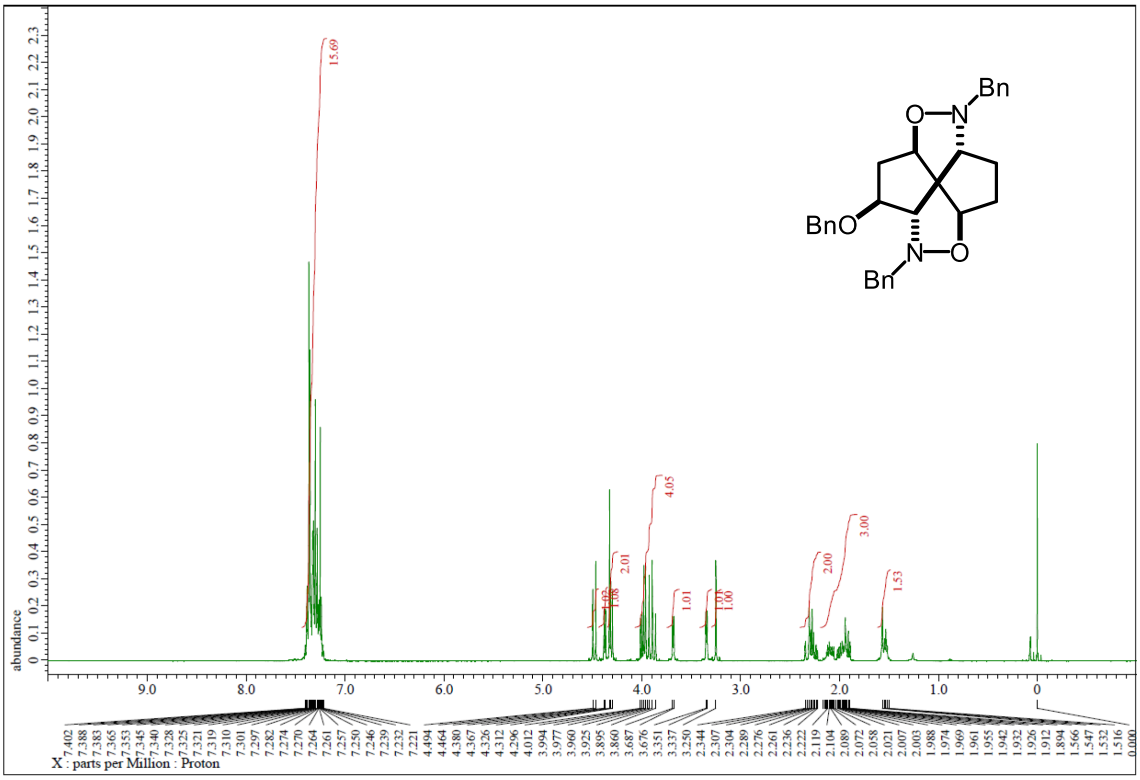

(^{13}C NMR, 100 MHz, $CDCl_3$)

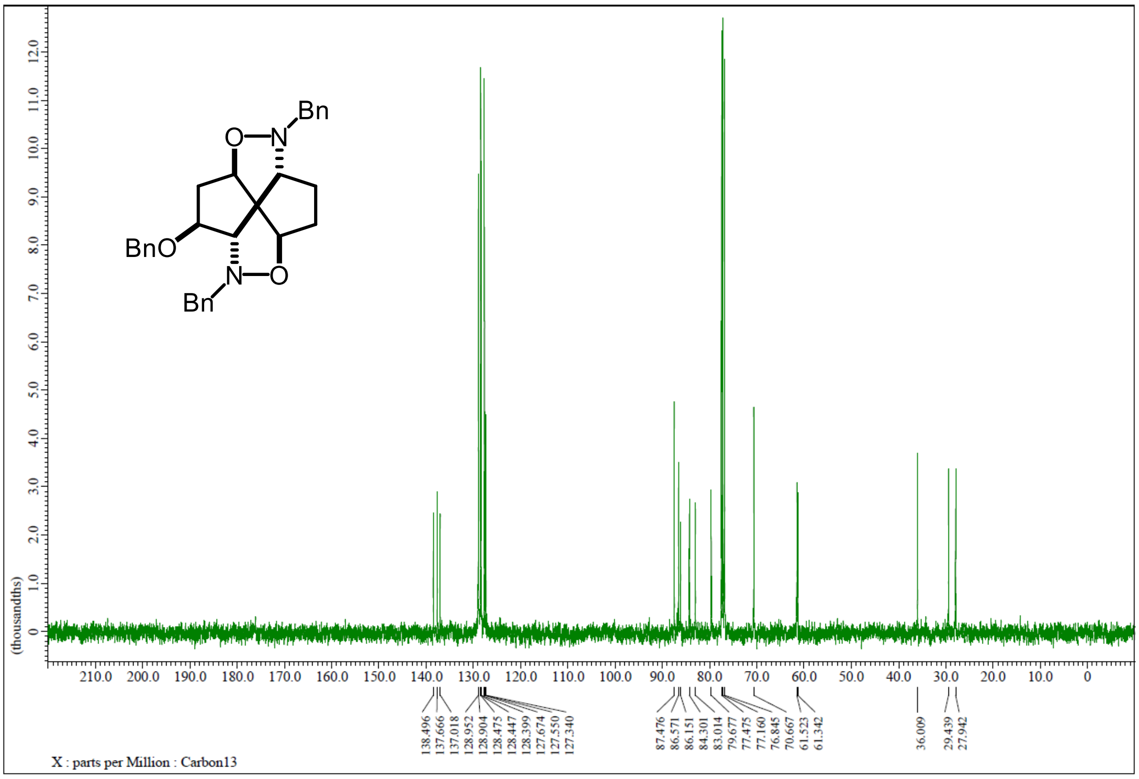

(COSY, 400 MHz, $CDCl_3$)

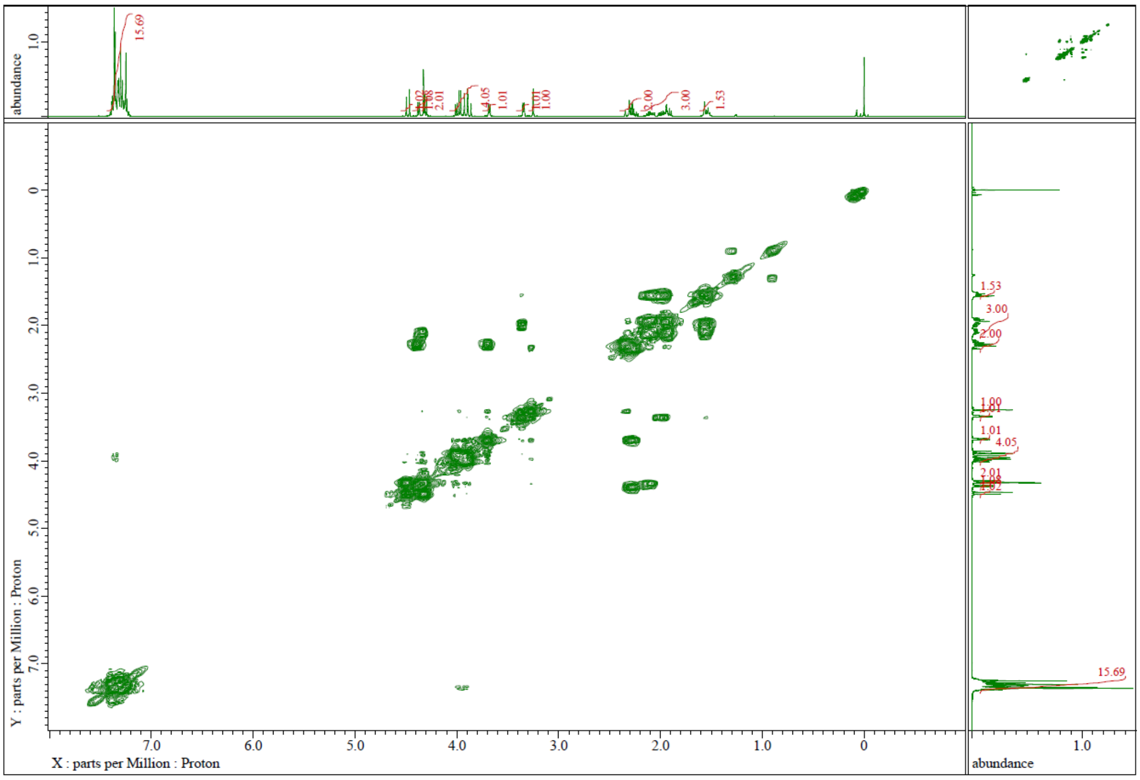

c,c,c,c-[5.5.5.5]-5R-N,N'-Dimethyl-5-benzyloxyl-2,8-dioxa-3,9-diazafenestrane (2q)

(^{1}H NMR, 400 MHz, $CDCl_3$)

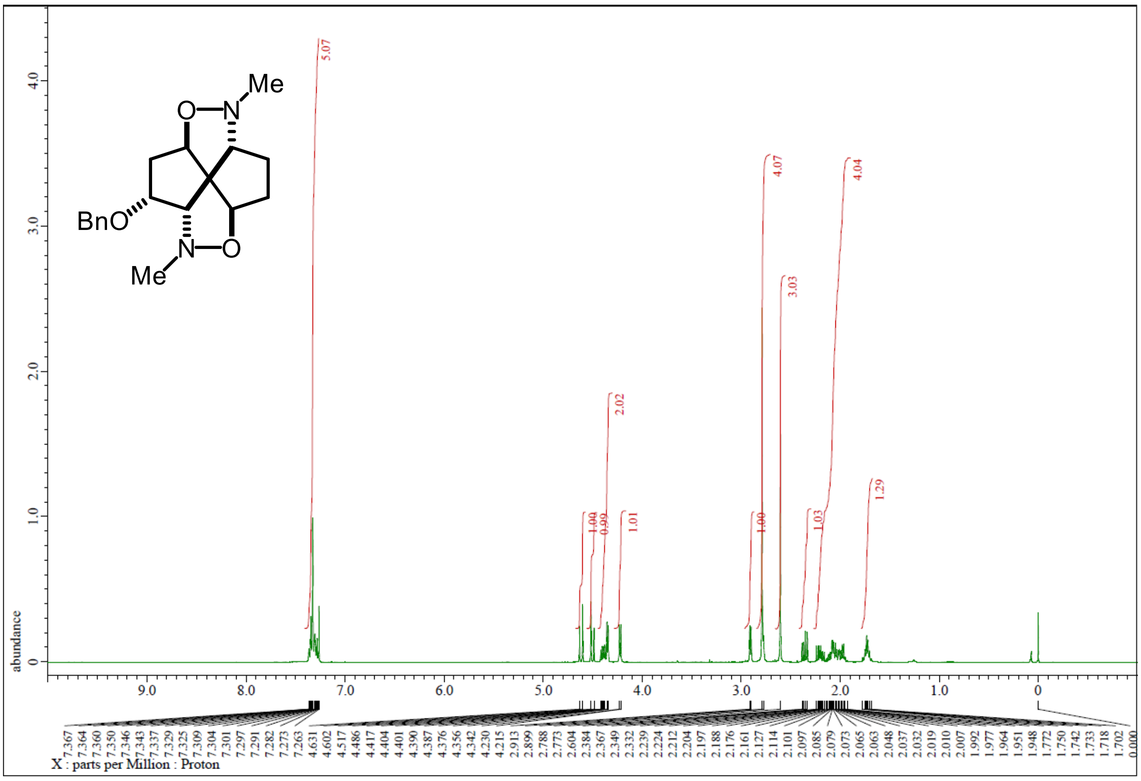

(^{13}C NMR, 100 MHz, $CDCl_3$)

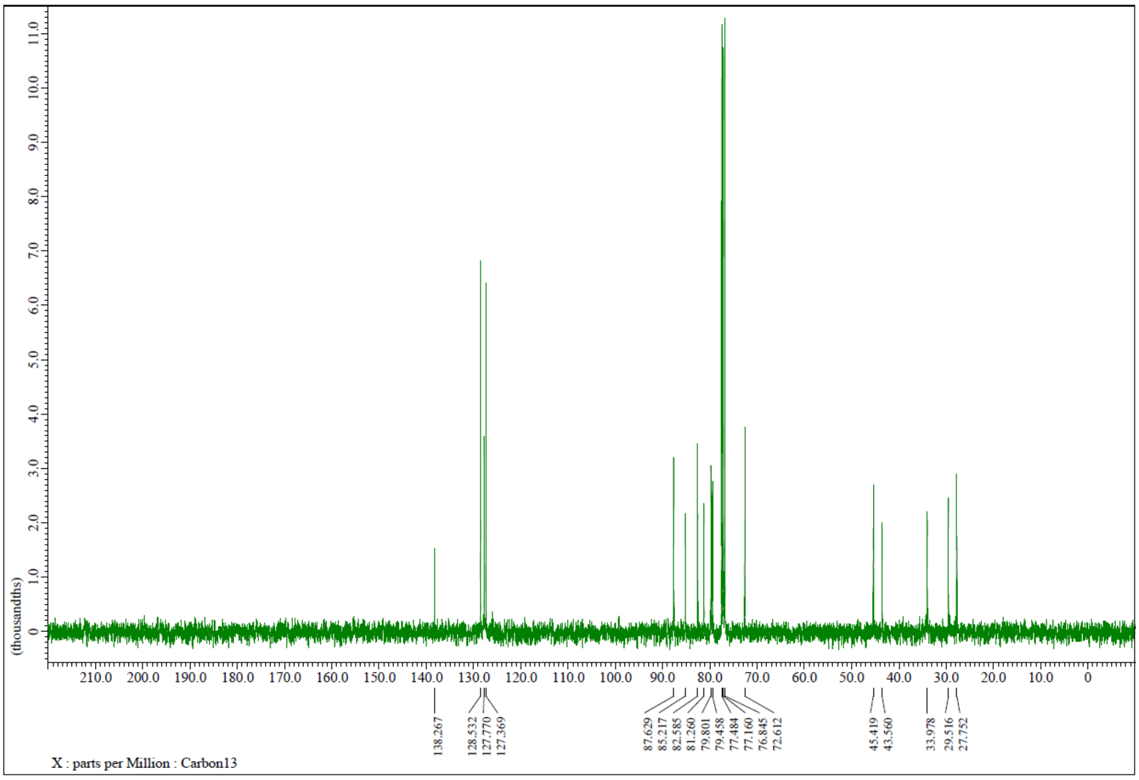

(COSY, 400 MHz, $CDCl_3$)

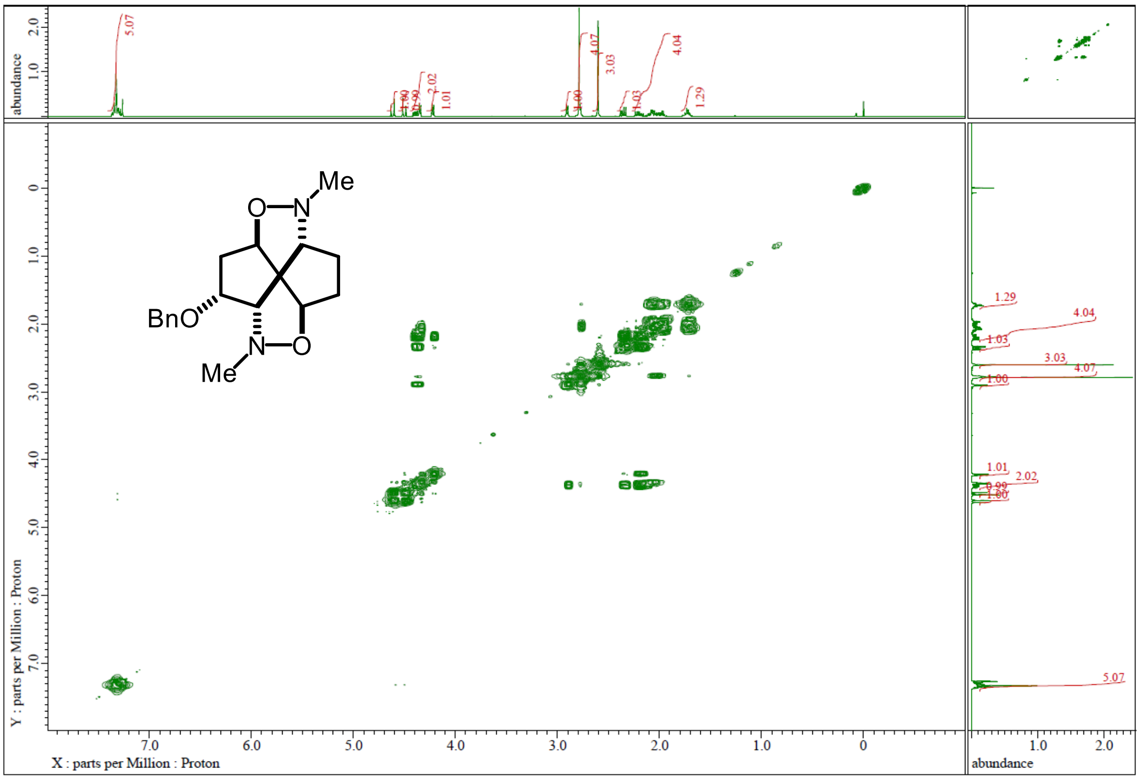

***c,c,c,c*-[5.5.5.5]-5*S*-*N*,*N'*-Dimethyl-5-benzyloxyl-2,8-dioxa-3,9-diazafenestrane (2q')**

(^{1}H NMR, 400 MHz, $CDCl_3$)

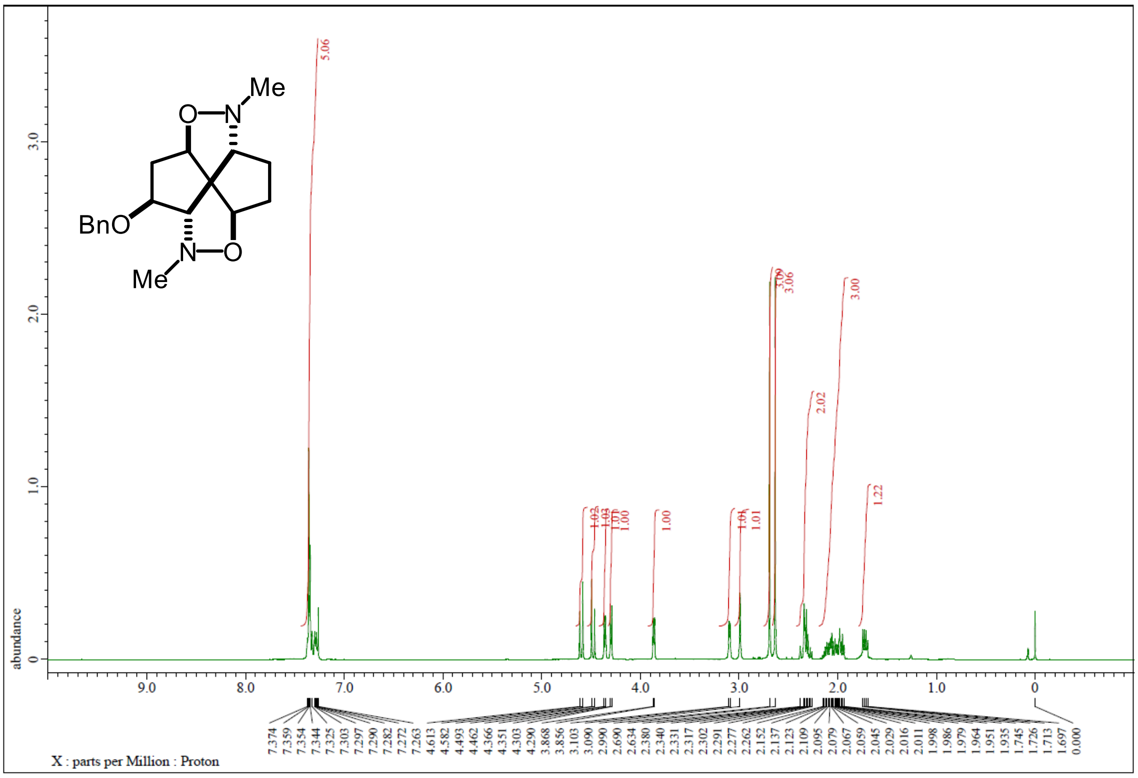

(^{13}C NMR, 100 MHz, $CDCl_3$)

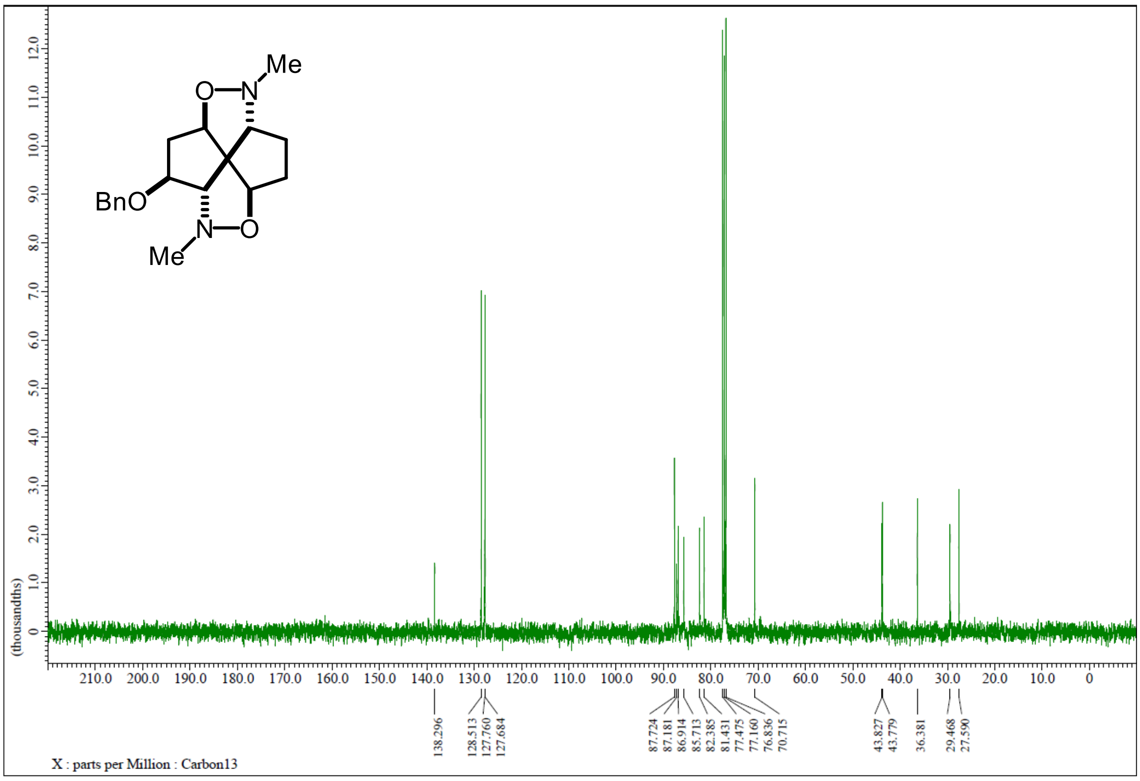

(COSY, 400 MHz, $CDCl_3$)

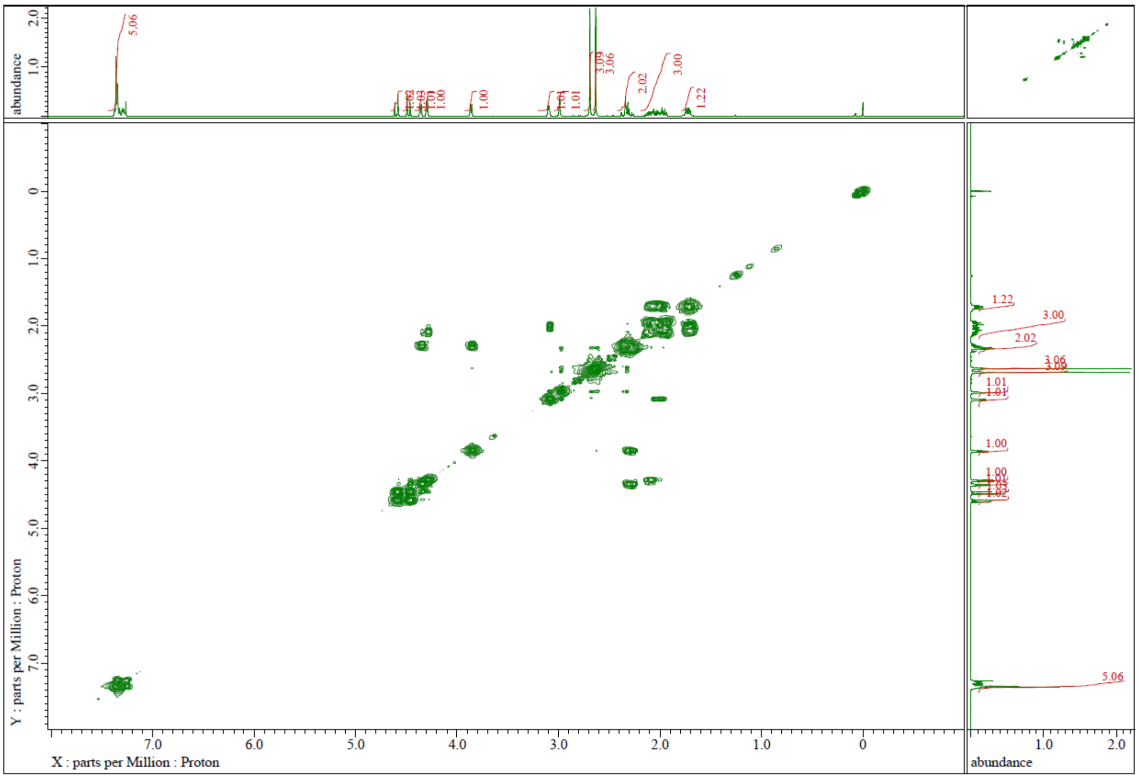

***c,c,c,c*-[5.5.5.6]-*N,N'*-Dibenzyl-2,8-dioxa-3,9-diazafenestrane (2r)**

(^{1}H NMR, 400 MHz, CDCl$_3$)

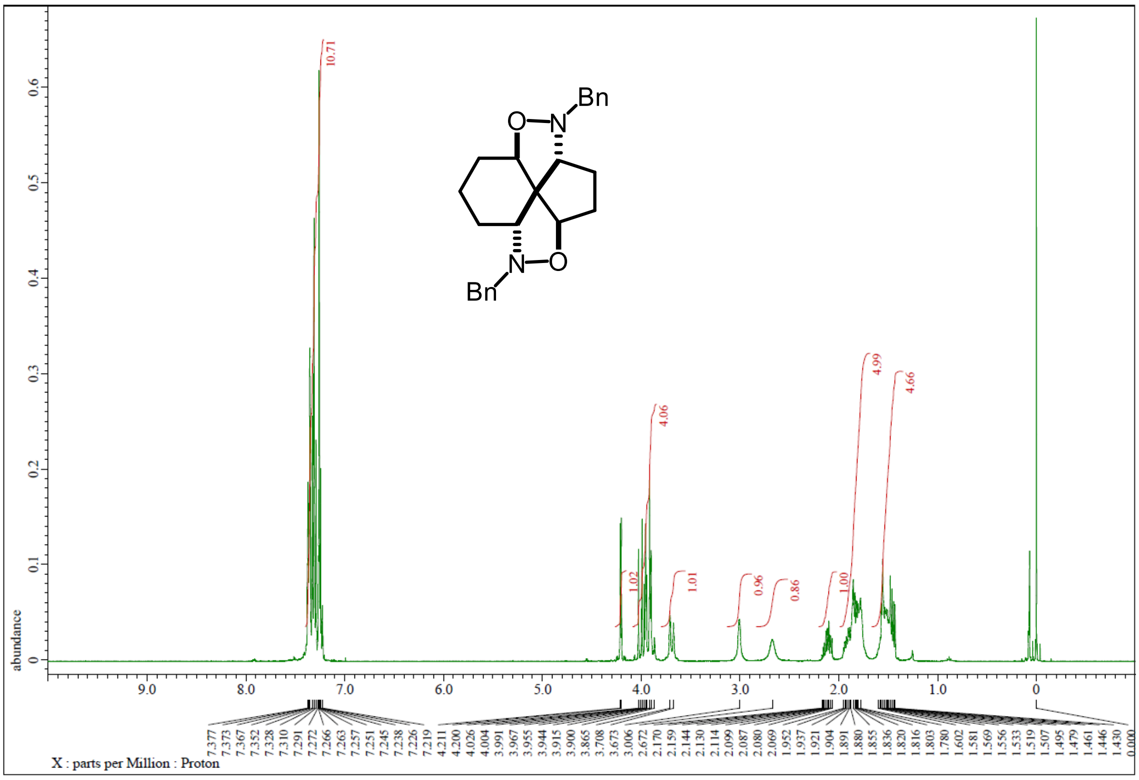

(^{13}C NMR, 100 MHz, CDCl$_3$)

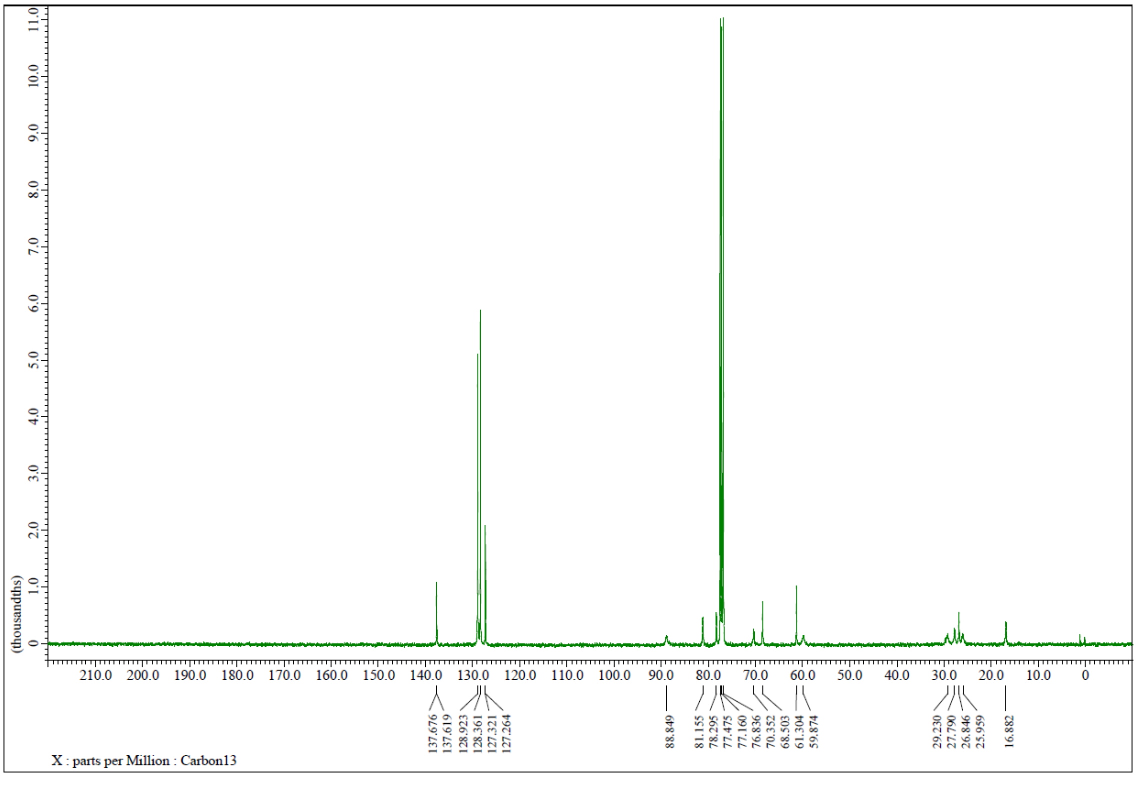

***c,c,c,c*-[5.6.5.6]-*N,N'*-Dibenzyl-2,9-dioxa-3,10-diazafenestrane (2s)**

(^{1}H NMR, 400 MHz, $CDCl_3$)

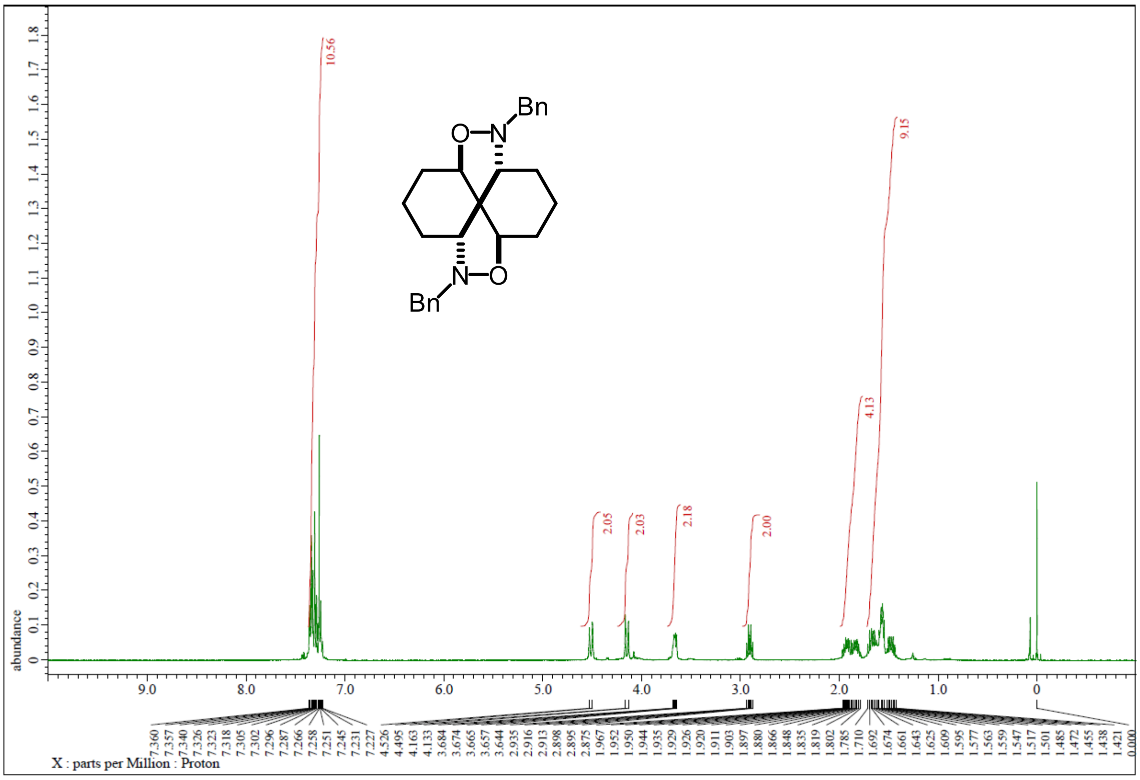

(^{13}C NMR, 100 MHz, $CDCl_3$)

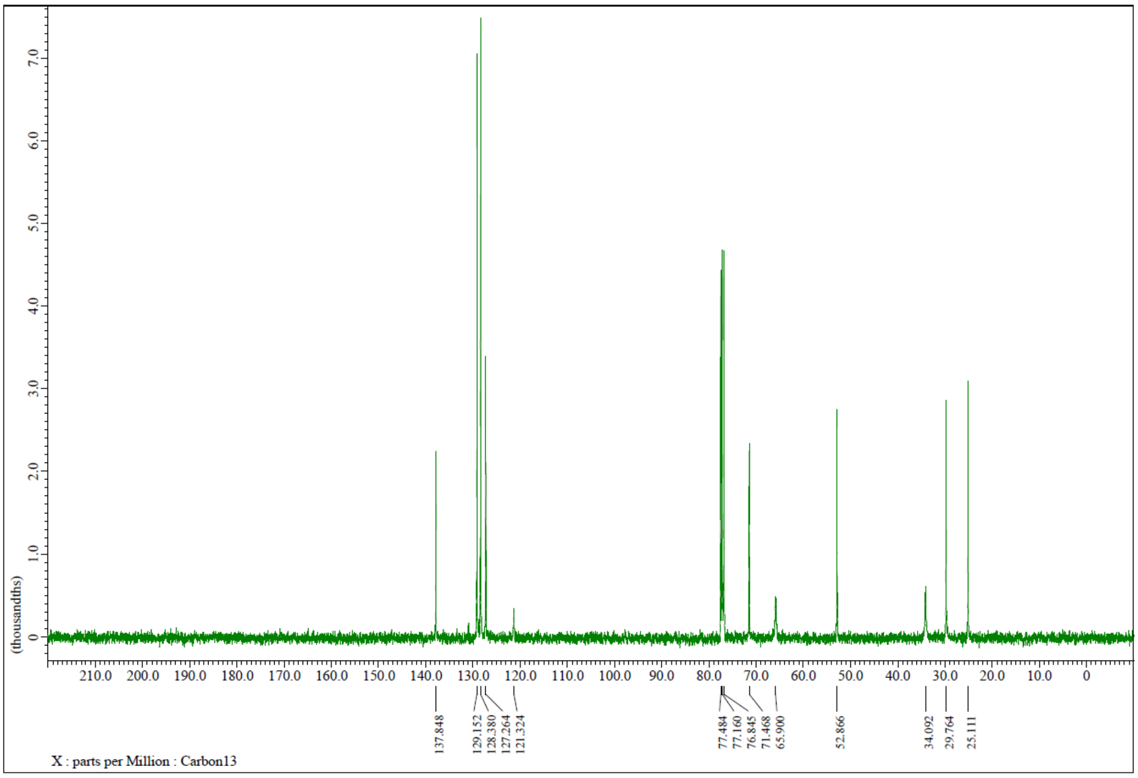

***c,c*-[5.5.5.5]-6-Hexyl-3,9-dioxa-1,7-diiminofenestrane (5b)**

(^{1}H NMR, 400 MHz, $CDCl_3$)

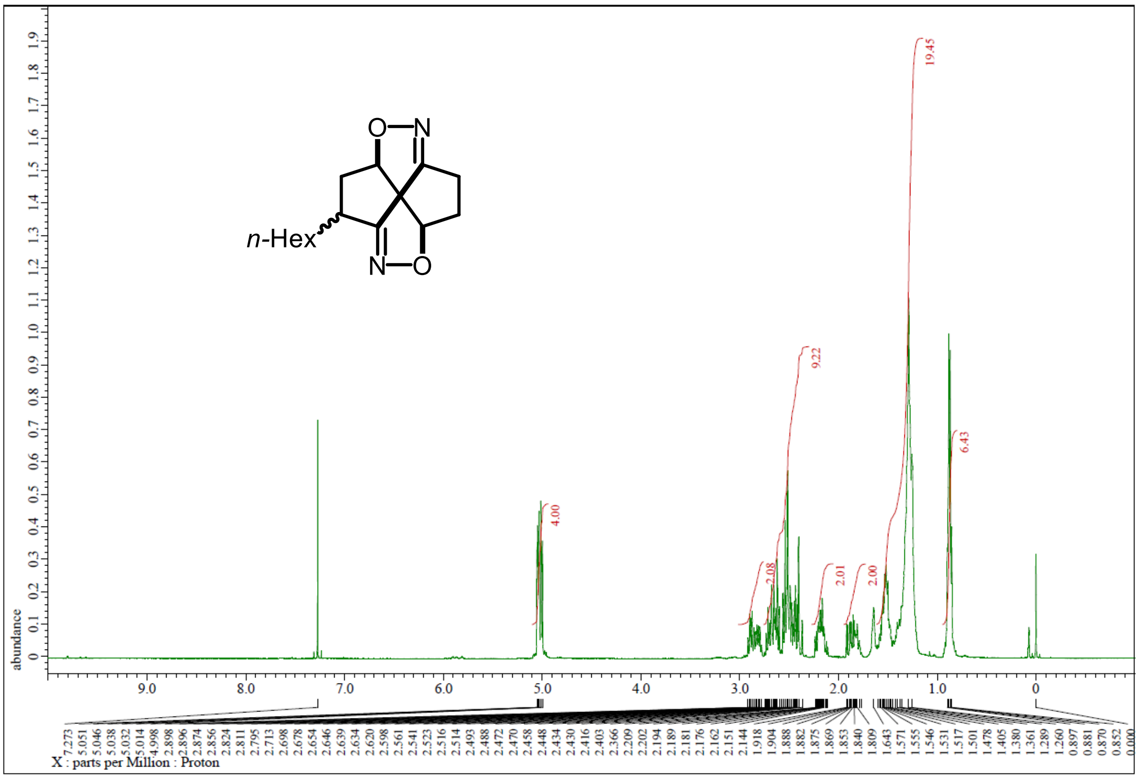

(^{13}C NMR, 100 MHz, $CDCl_3$)

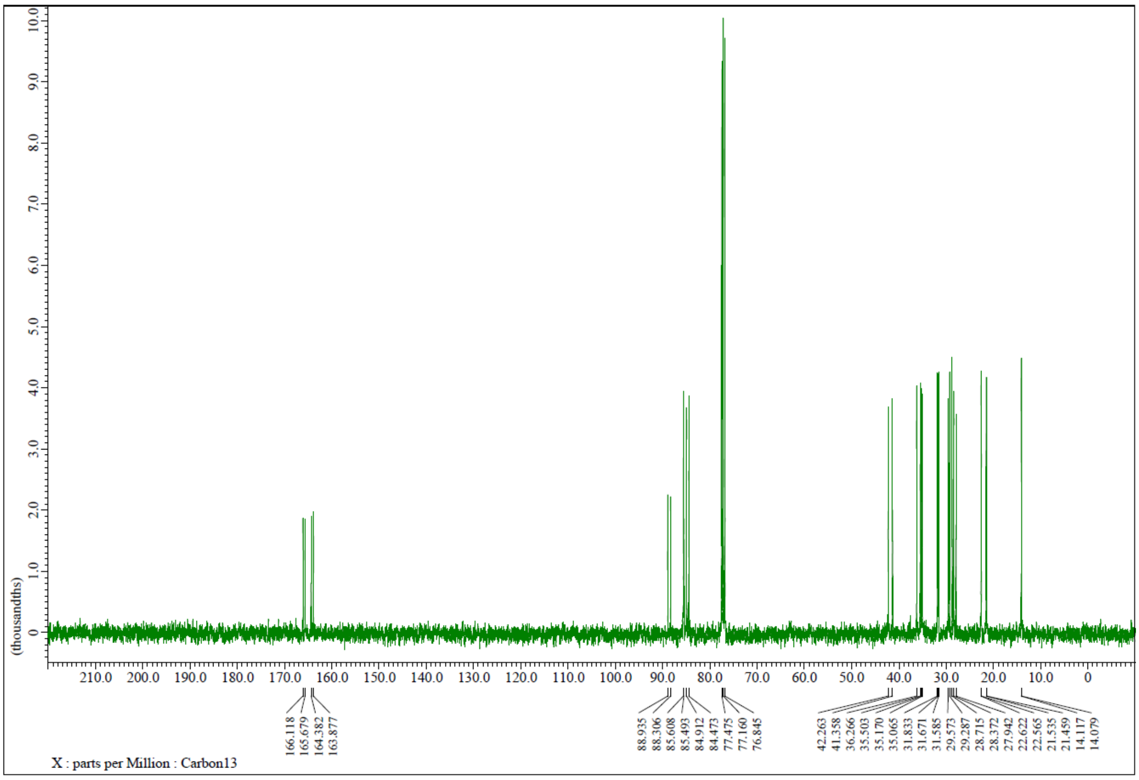

***c,c*-[5.5.5.5]-6*R*-6-Benzyloxyl -3,9-dioxa-1,7-diiminofenestrane (5c)**

(^{1}H NMR, 400 MHz, $CDCl_3$)

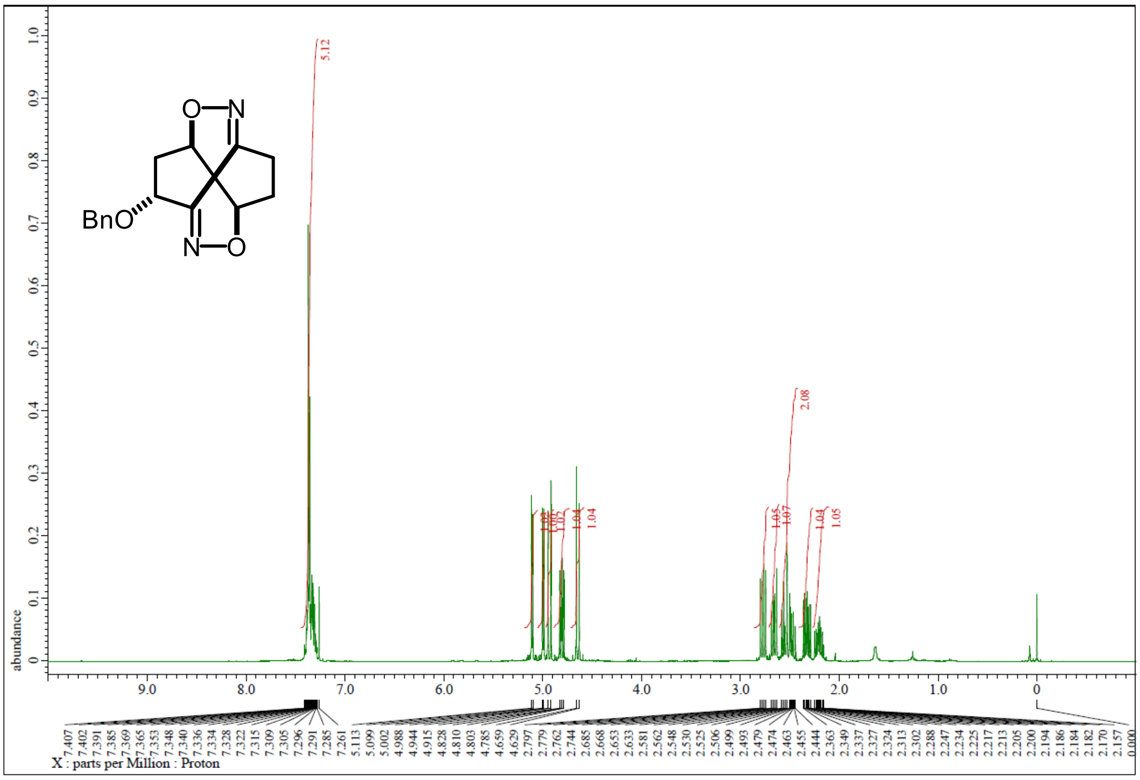

(^{13}C NMR, 100 MHz, $CDCl_3$)

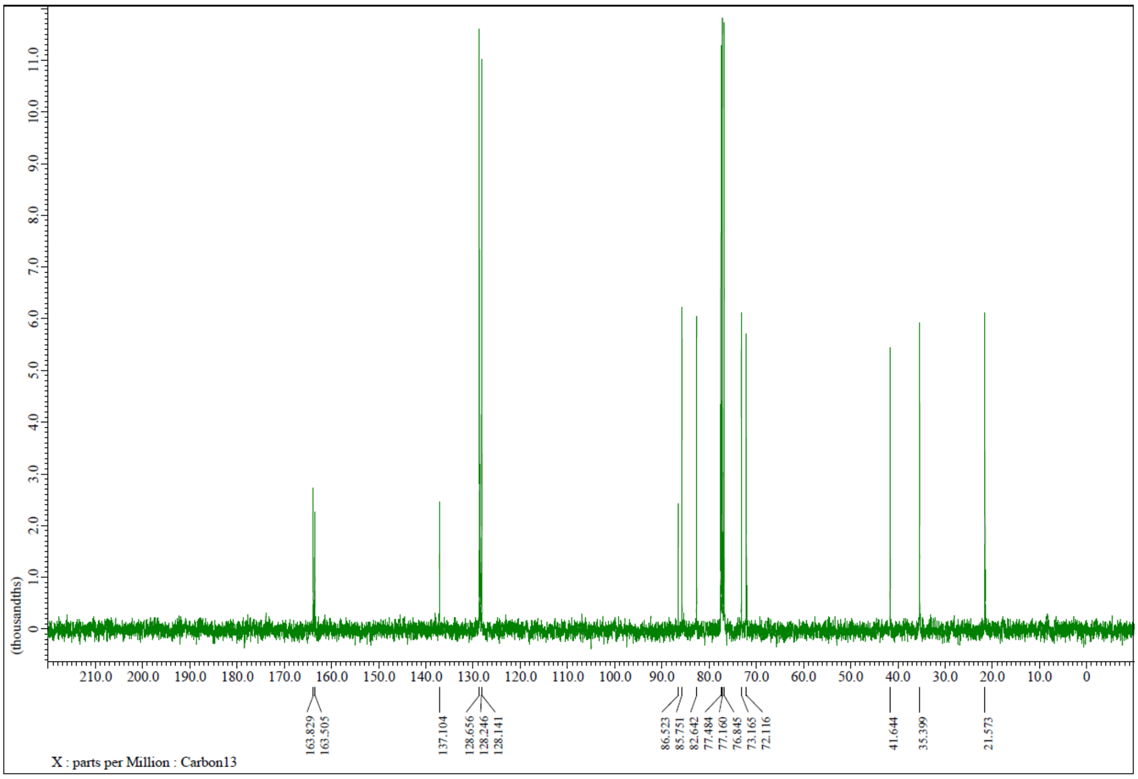

(COSY, 400 MHz, $CDCl_3$)

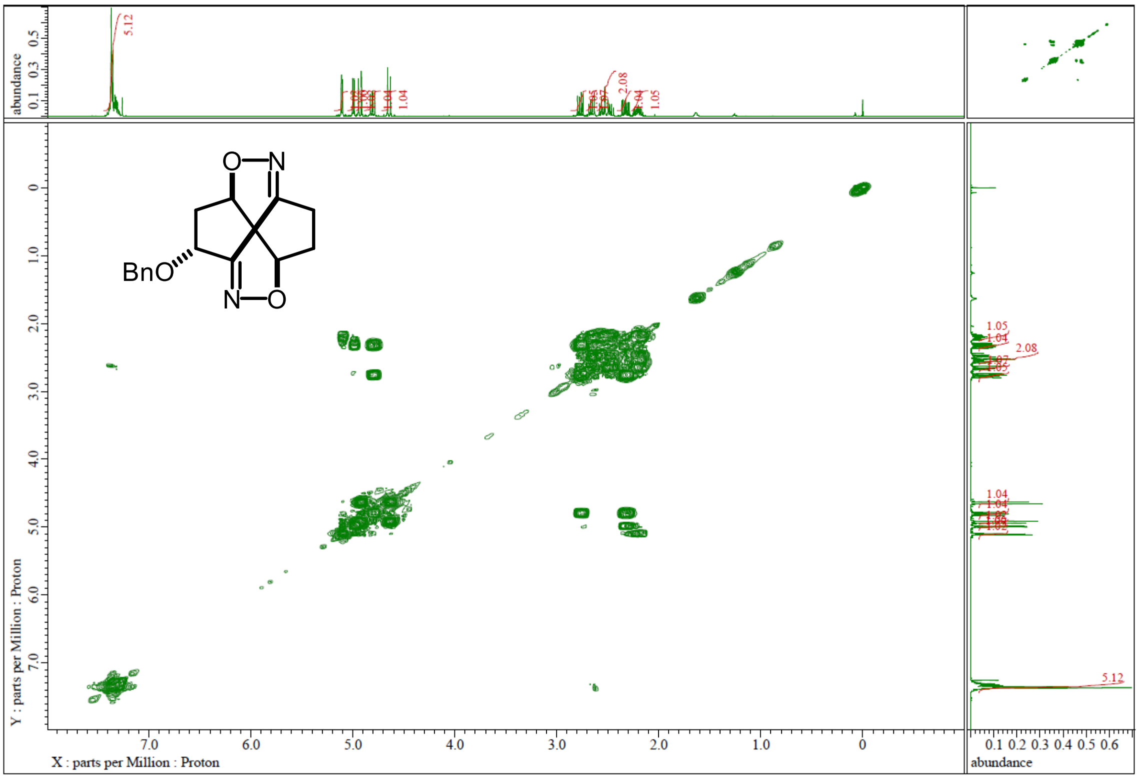

c,c-[5.5.5.5]-6*S*-6-Benzyloxyl -3,9-dioxa-1,7-diiminofenestrane (5c')

(^1H NMR, 400 MHz, $CDCl_3$)

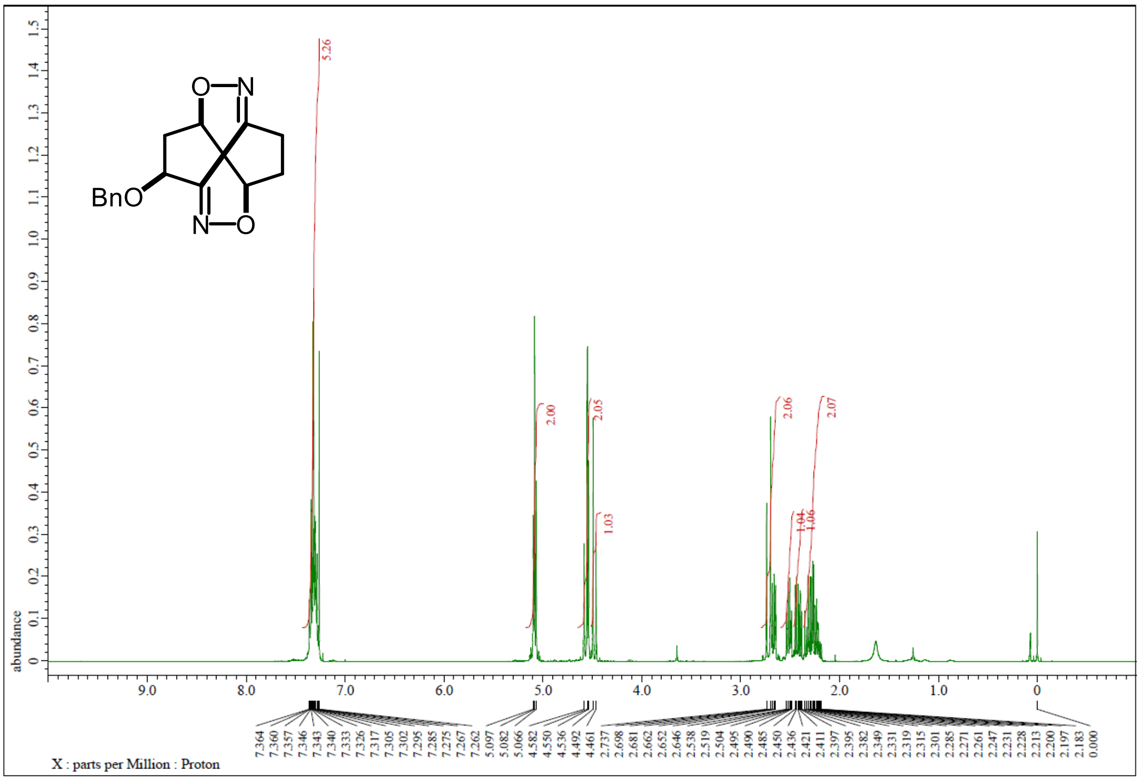

(^{13}C NMR, 100 MHz, $CDCl_3$)

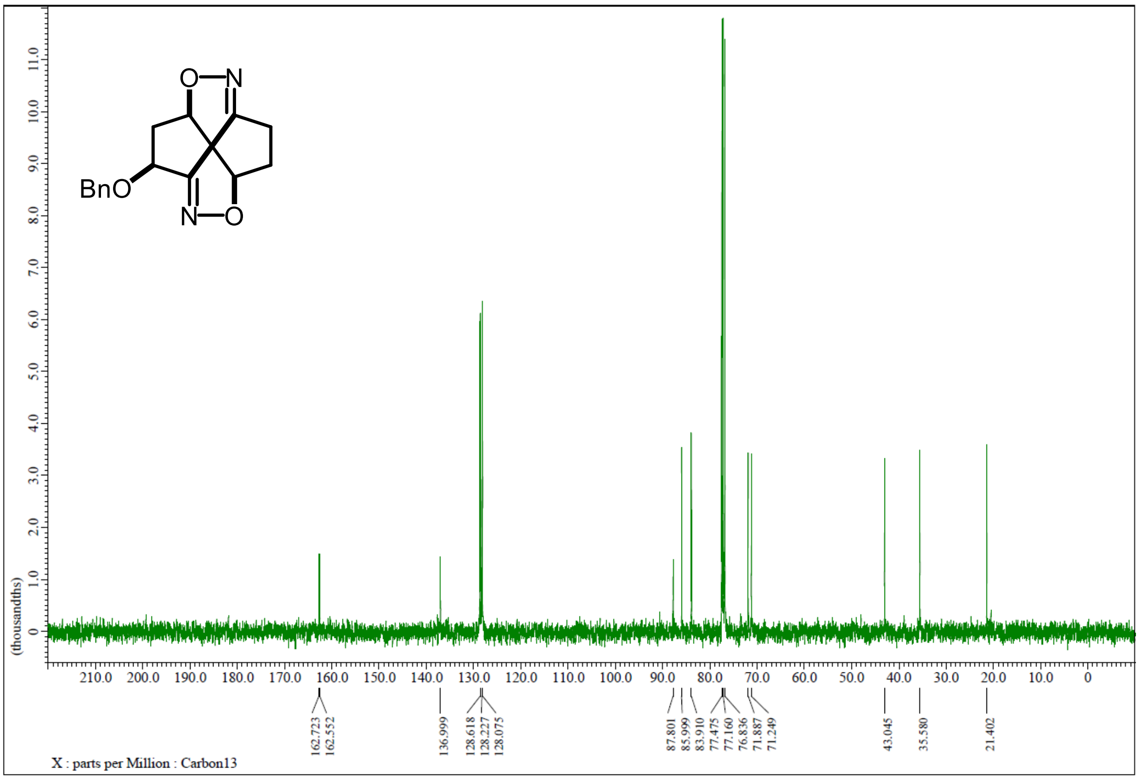

(COSY, 400 MHz, $CDCl_3$)

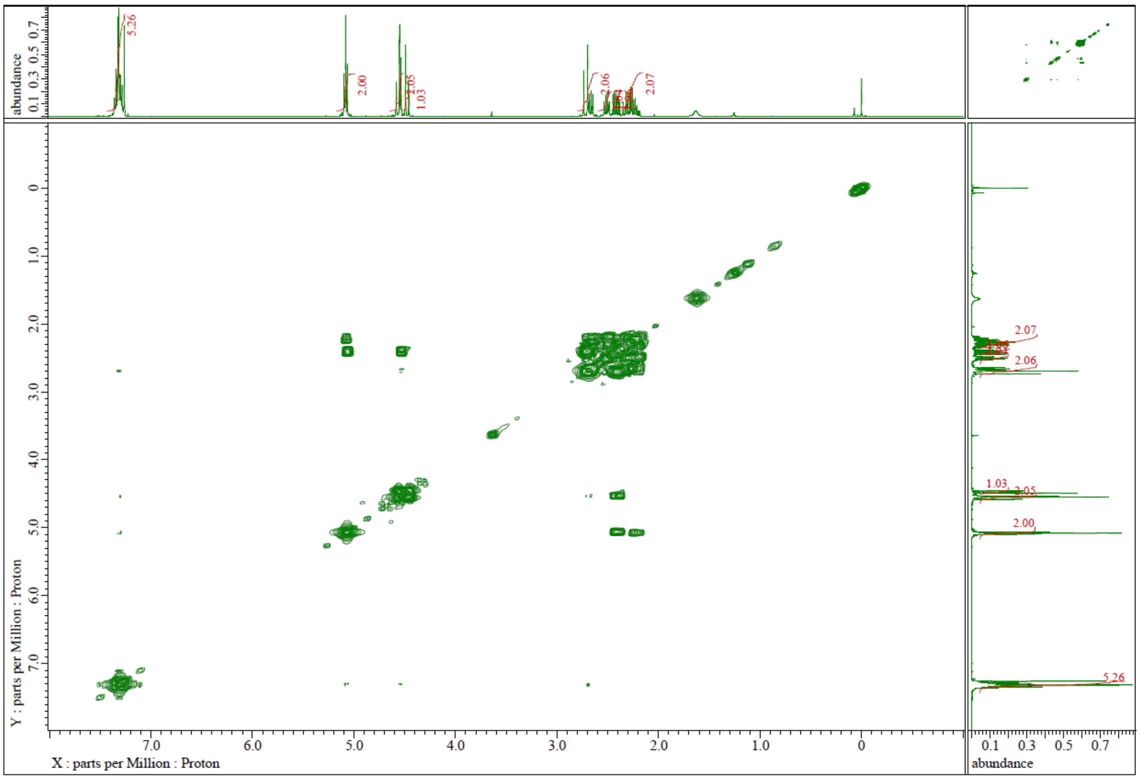

***c,c*-[5.5.5.6]-3,9-Dioxa-1,7-diiminofenestrane (5d)**

(^{1}H NMR, 400 MHz, $CDCl_3$)

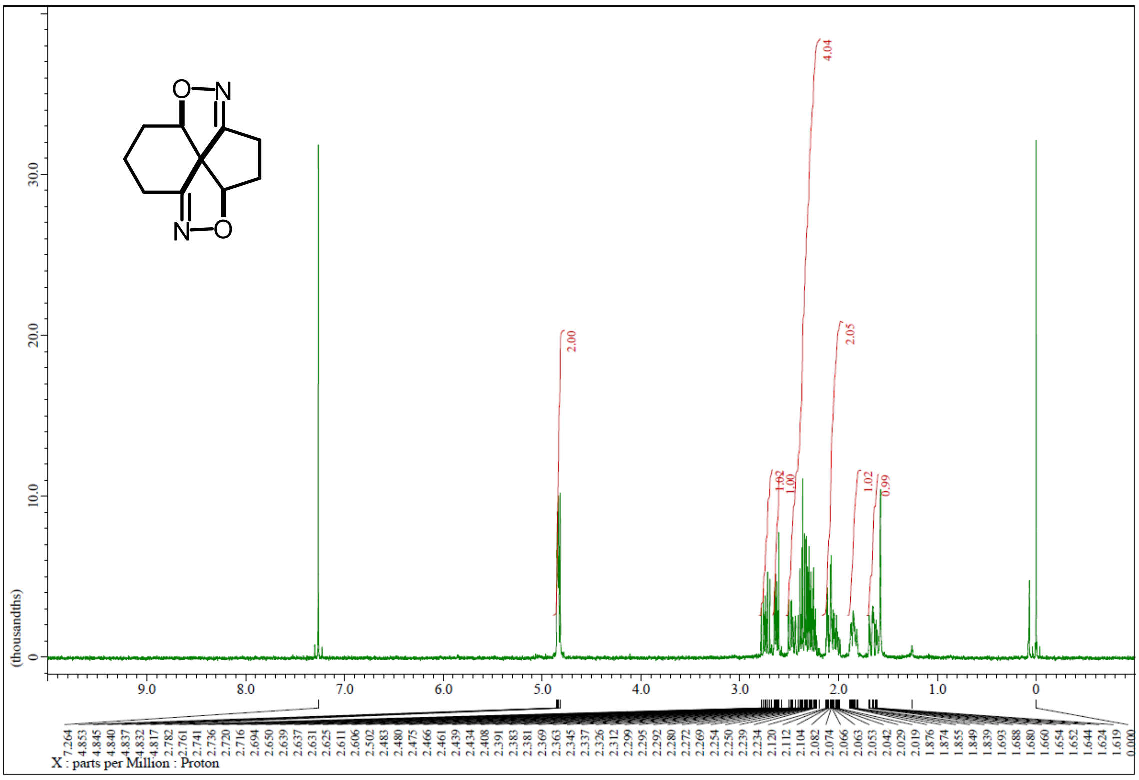

(^{13}C NMR, 100 MHz, $CDCl_3$)

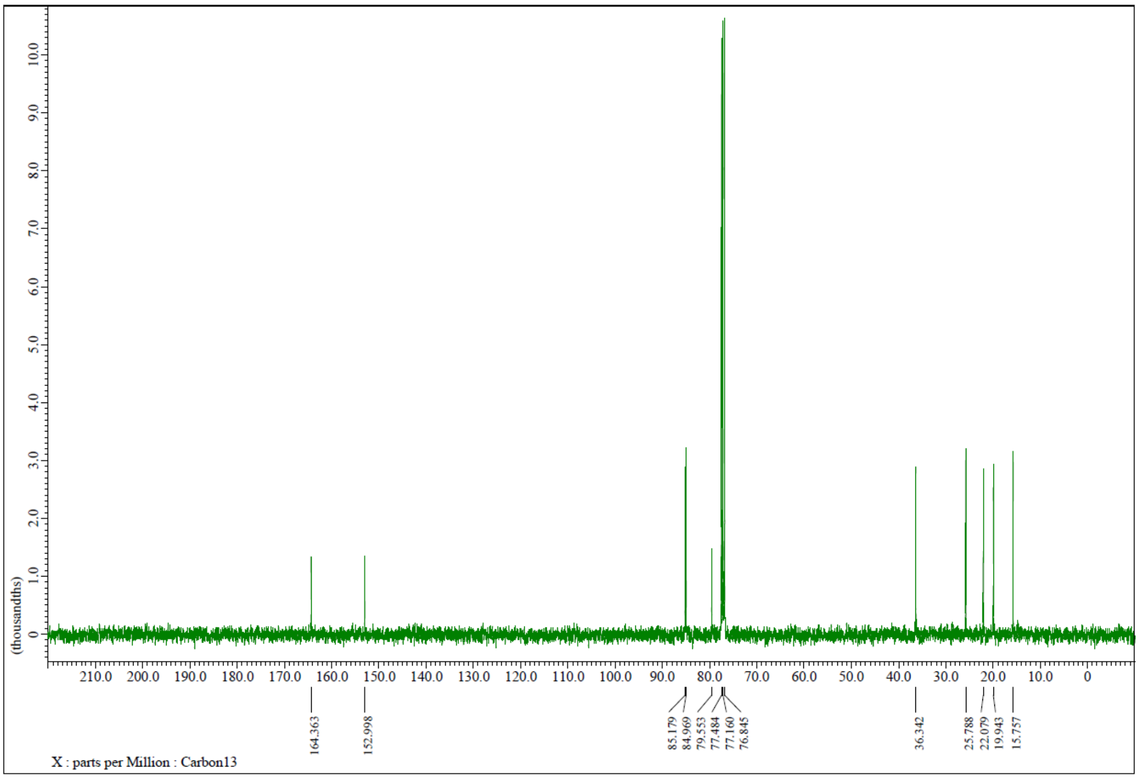

***c,c,c,c*-[5.5.5.5]-*N,N'*-Di((4'-methoxy-[1,1'-biphenyl]-4-yl)methyl)-2,8-dioxa-3,9-diazafenestrane (7a)**

(^{1}H NMR, 400 MHz, $CDCl_3$)

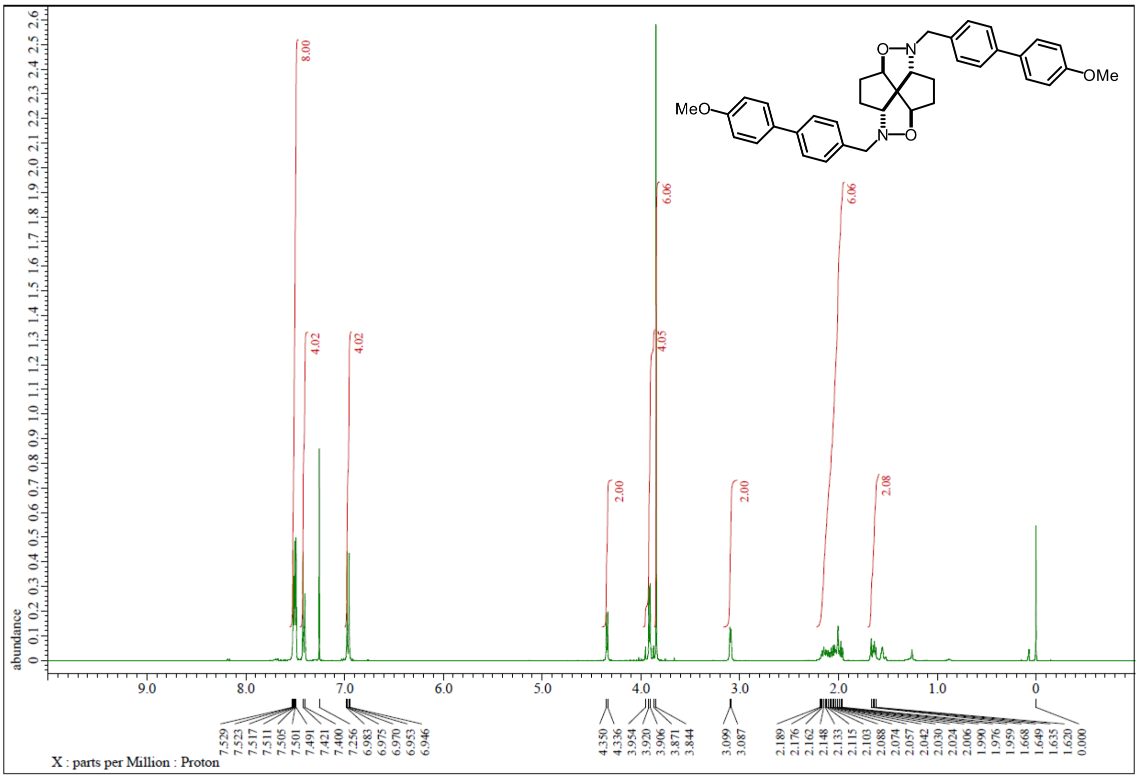

(^{13}C NMR, 100 MHz, $CDCl_3$)

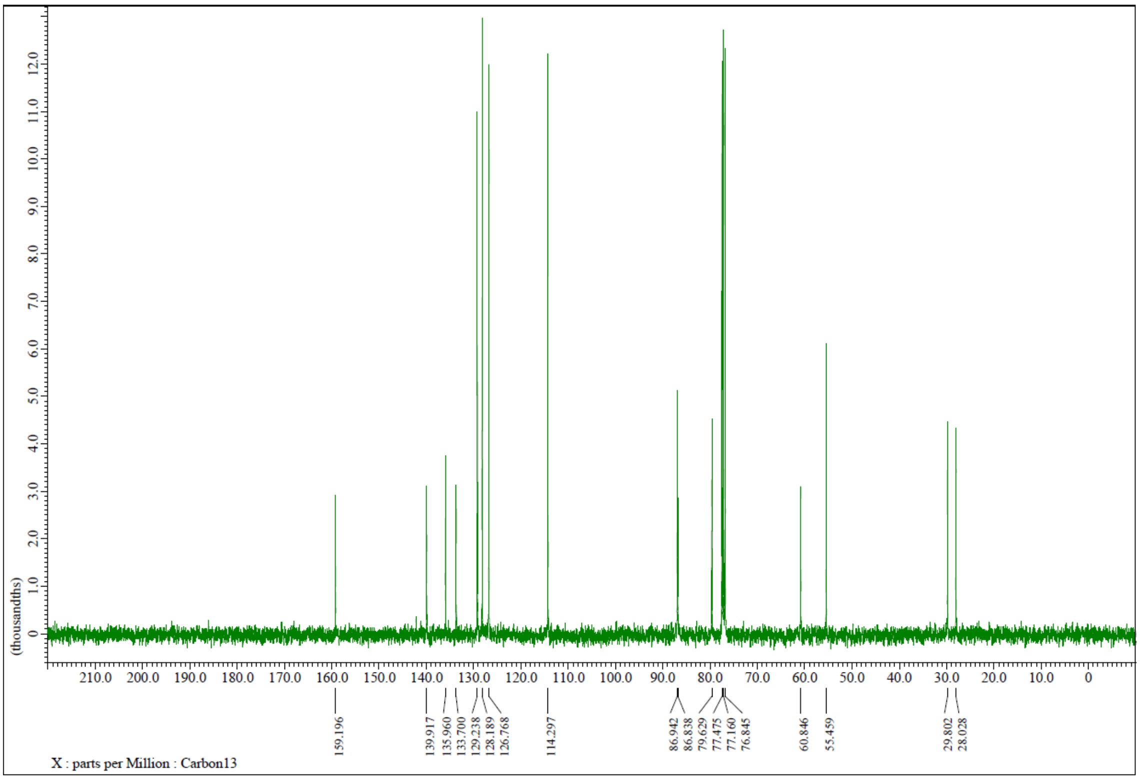

***c,c,c,c*-[5.5.5.5]-*N,N'*-Di((4'-(trifluoromethyl)-[1,1'-biphenyl]-4-yl)methyl)-2,8-dioxa-3,9-diazafenestrane (7b)**

(^{1}H NMR, 400 MHz, $CDCl_3$)

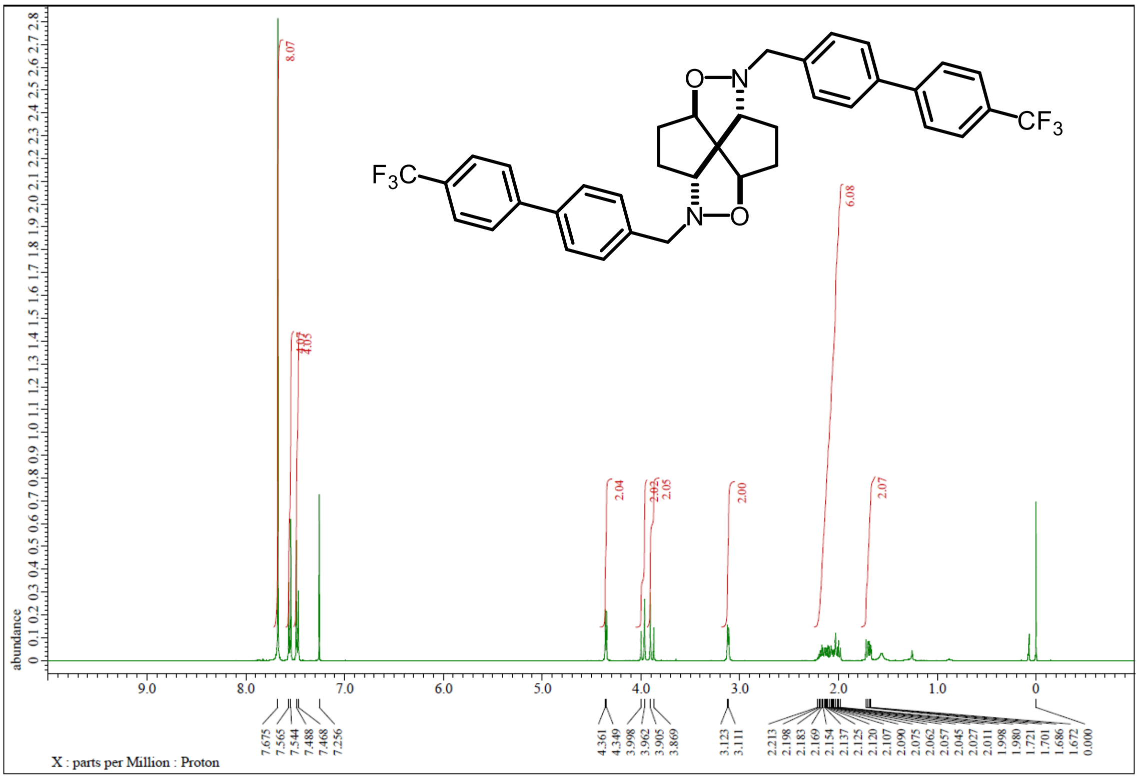

(^{13}C NMR, 100 MHz, $CDCl_3$)

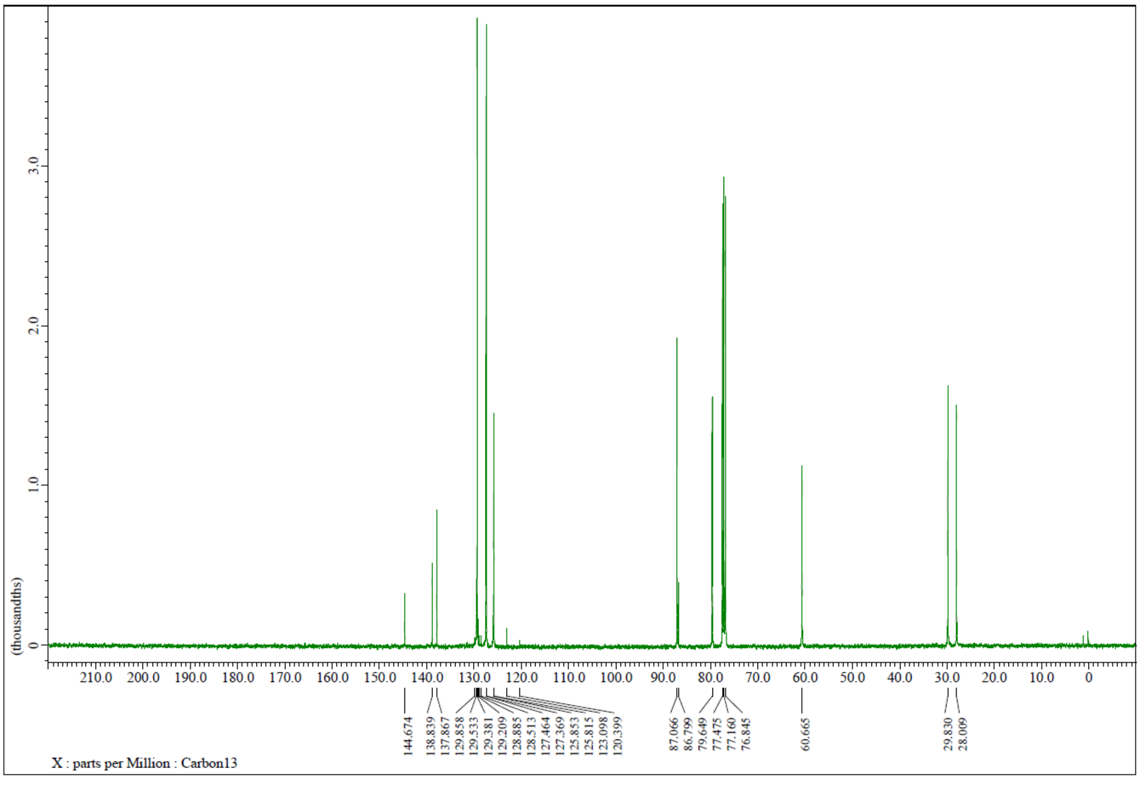

(19F NMR, 376 MHz, $CDCl_3$)

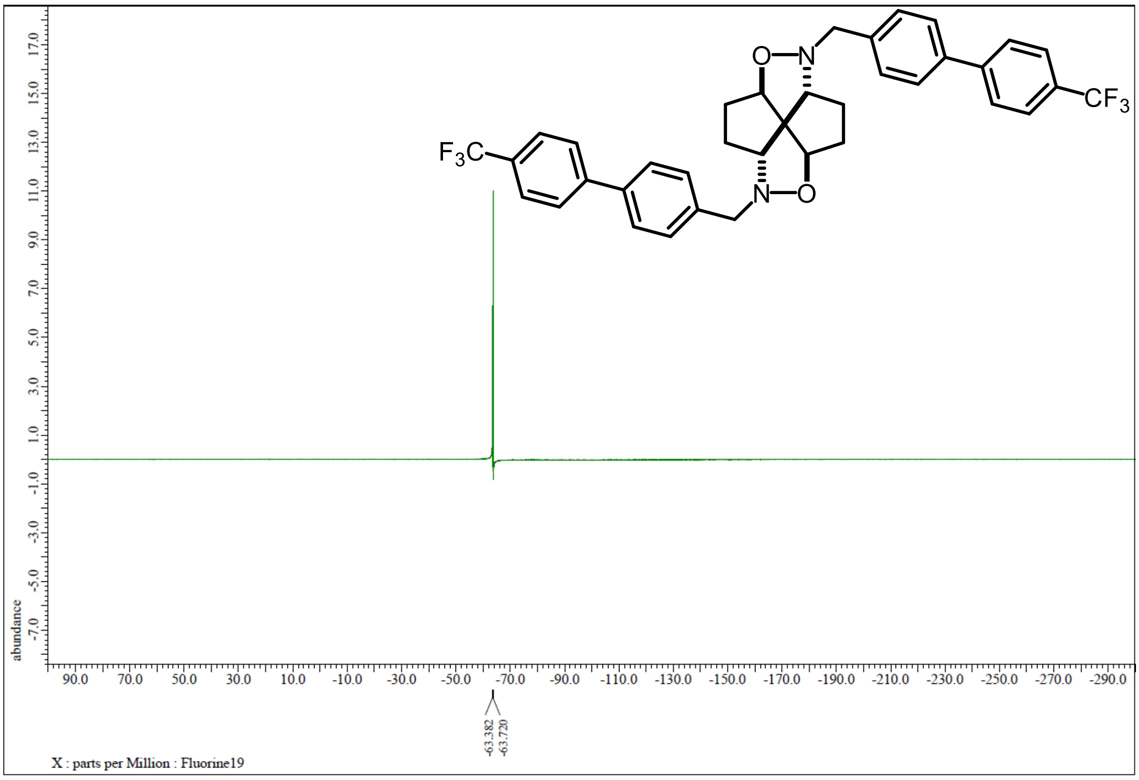

***c,c,c,c*-[5.5.5.5]-*N,N'*-Di((4-(3-hydroxy-3-methylbut-1-yn-1-yl)phenyl)methyl)-2,8-dioxa-3,9-diazafenestrane (7c)**

(^{1}H NMR, 400 MHz, $CDCl_3$)

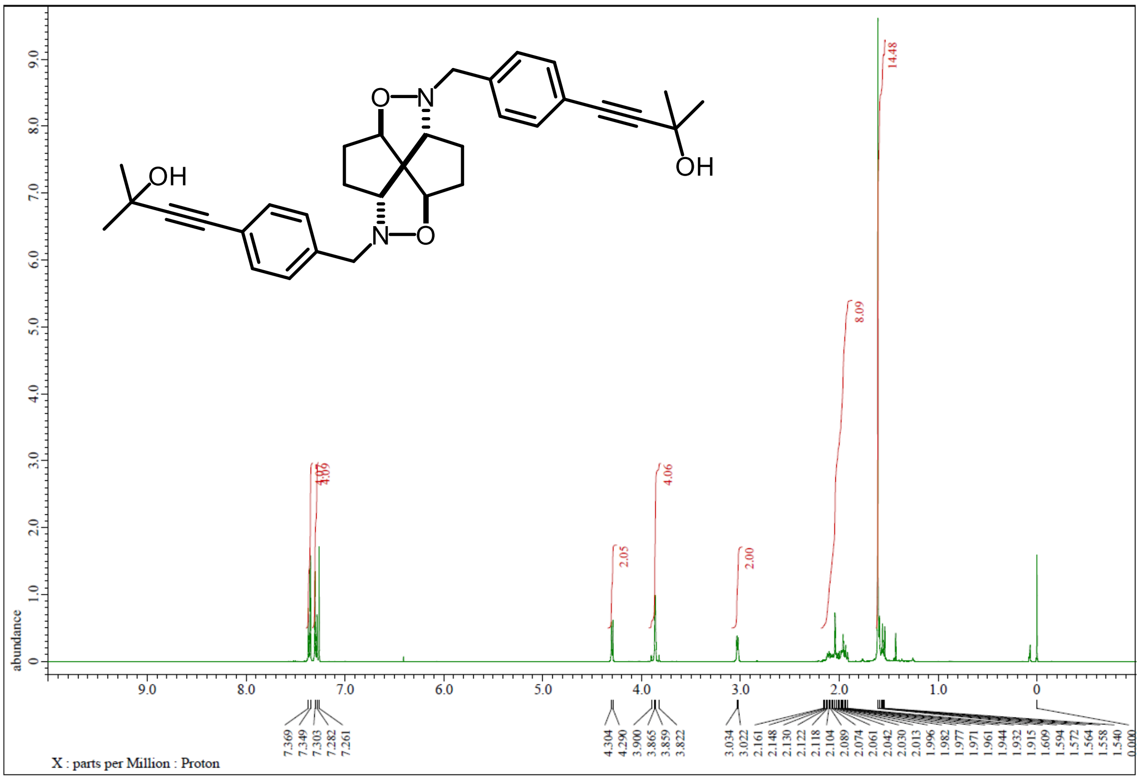

(^{13}C NMR, 100 MHz, $CDCl_3$)

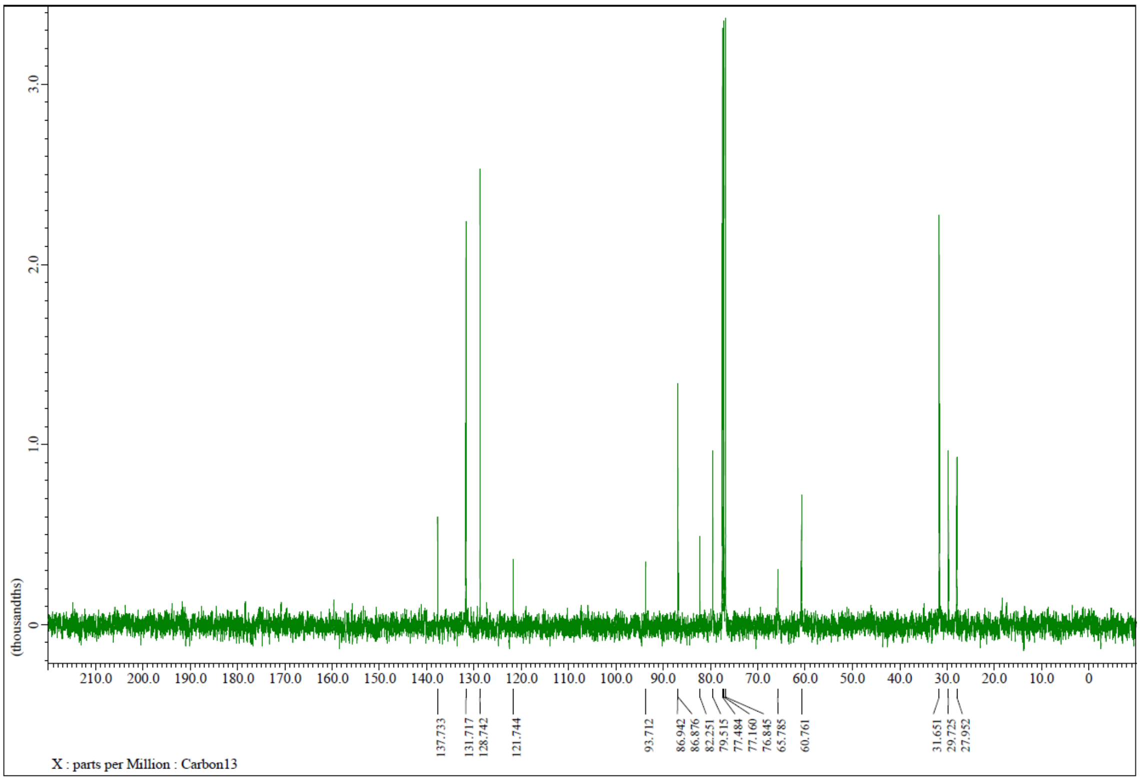

***c*,*c*,*c*,*c*-[5.5.5.5]-*N*,*N'*-Di((*E*)-(4-(3-butoxy-3-oxoprop-1-en-1-yl)phenyl)methyl)-2,8-dioxa-3,9-diazafenestrane (7d)**

(^{1}H NMR, 400 MHz, CDCl$_3$)

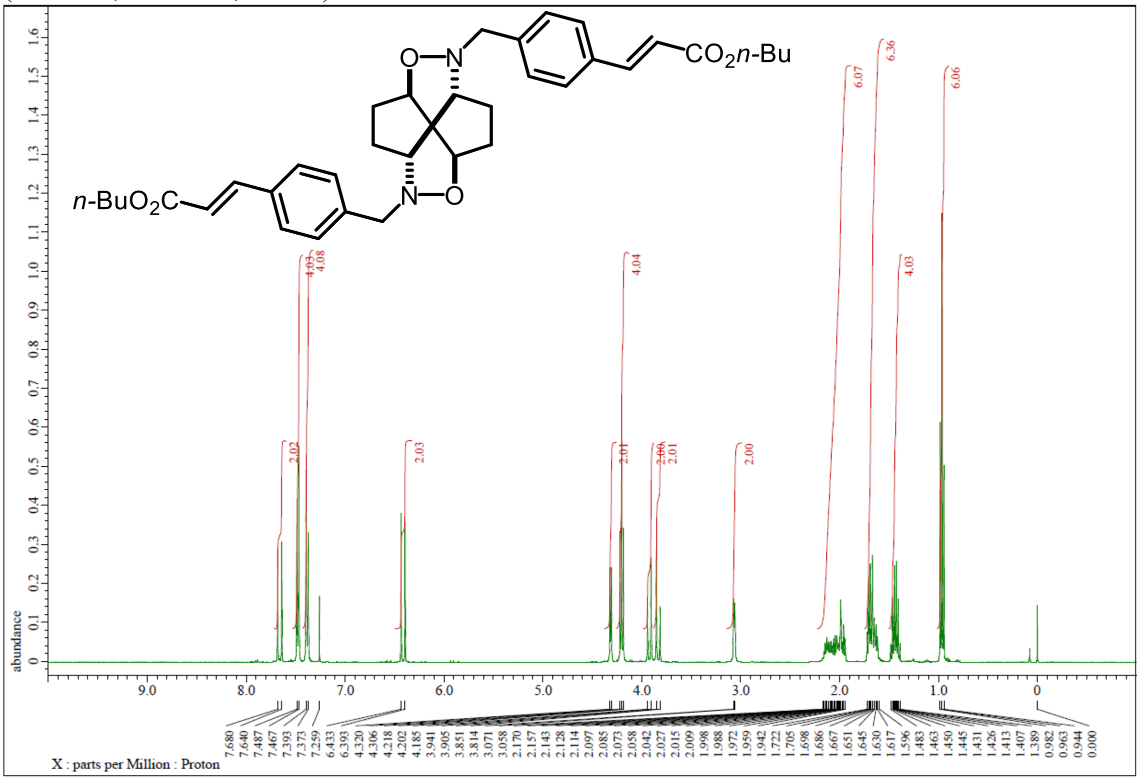

(^{13}C NMR, 100 MHz, CDCl$_3$)

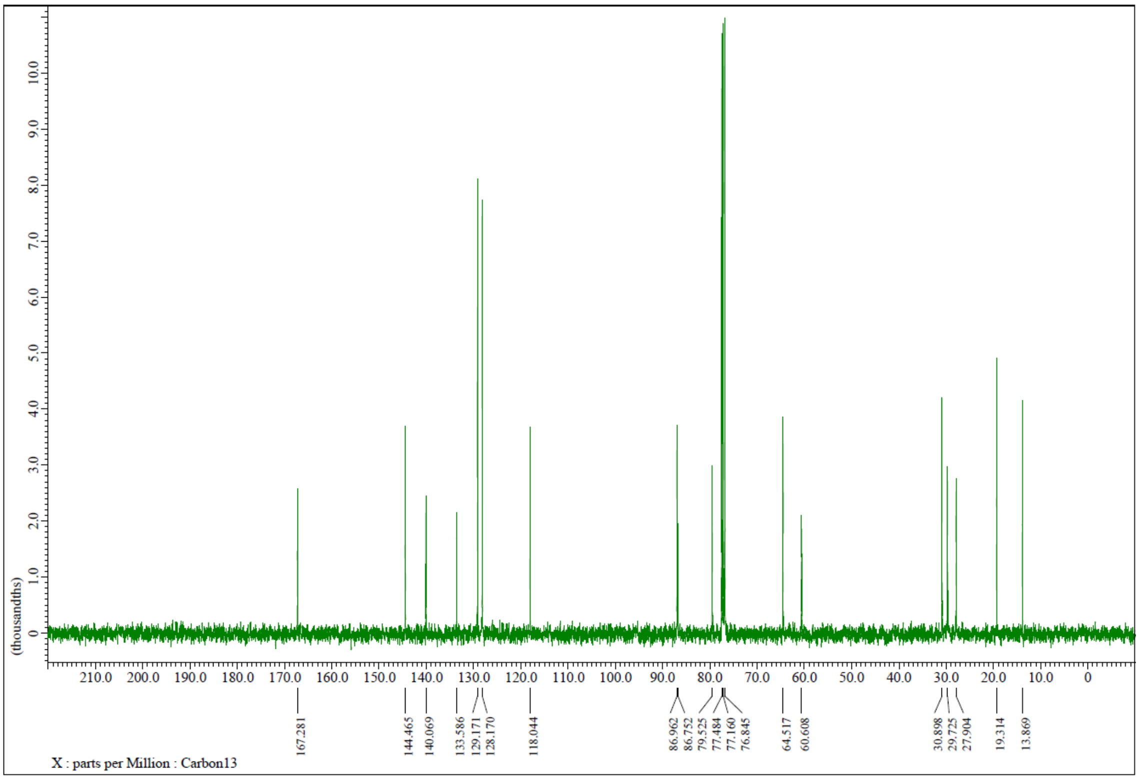

(1*R*,4*R*,5*S*,6*R*,9*R*)-4,9-Bis(benzylamino)spiro[4.4]nonane-1,6-diol (8)

(^{1}H NMR, 400 MHz, CD_3OD)

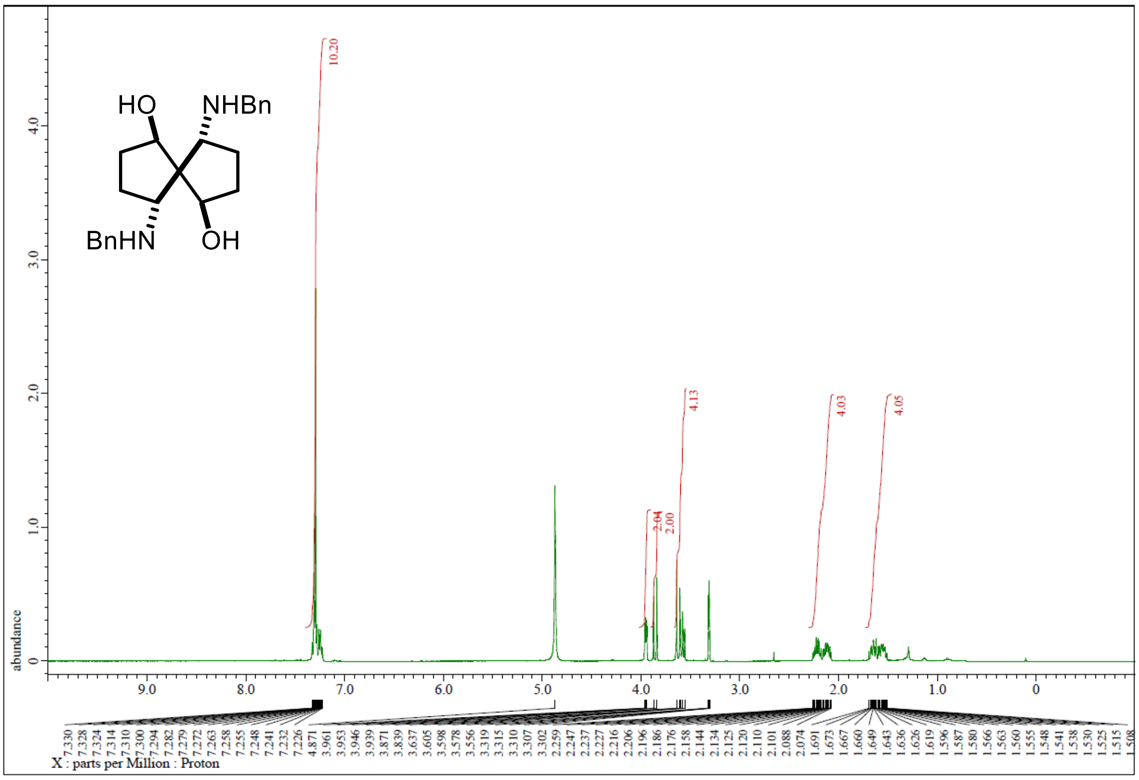

(^{13}C NMR, 100 MHz, CD_3OD)

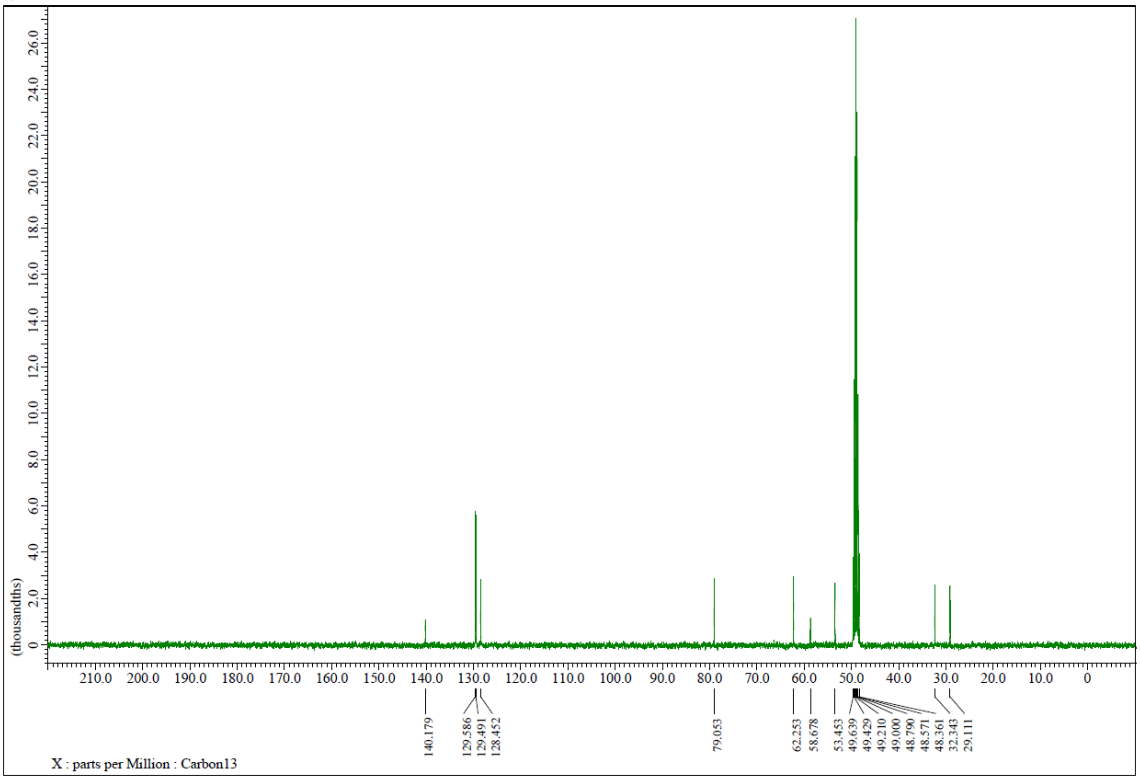

c,c,c-[5.5.5.5]-3,9-Dioxa-2-aza-1-(2-propenyl)-7-iminofenestrane (9a)

(^{1}H NMR, 400 MHz, $CDCl_3$)

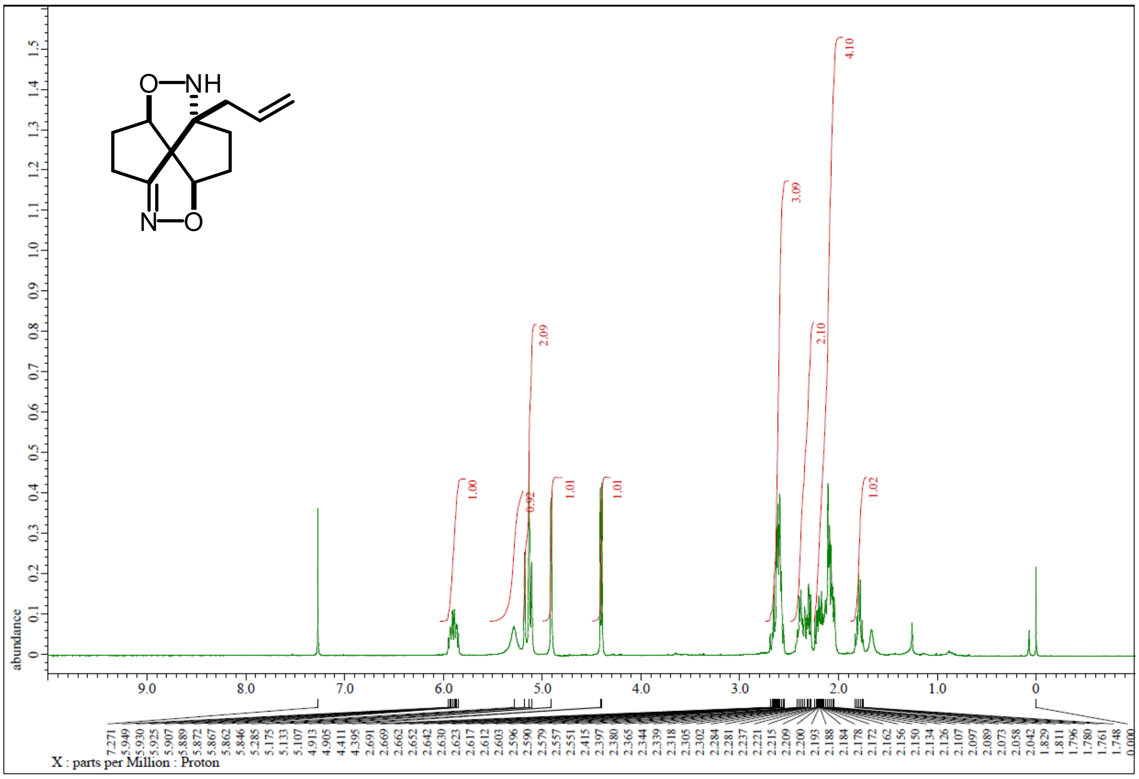

(^{13}C NMR, 100 MHz, $CDCl_3$)

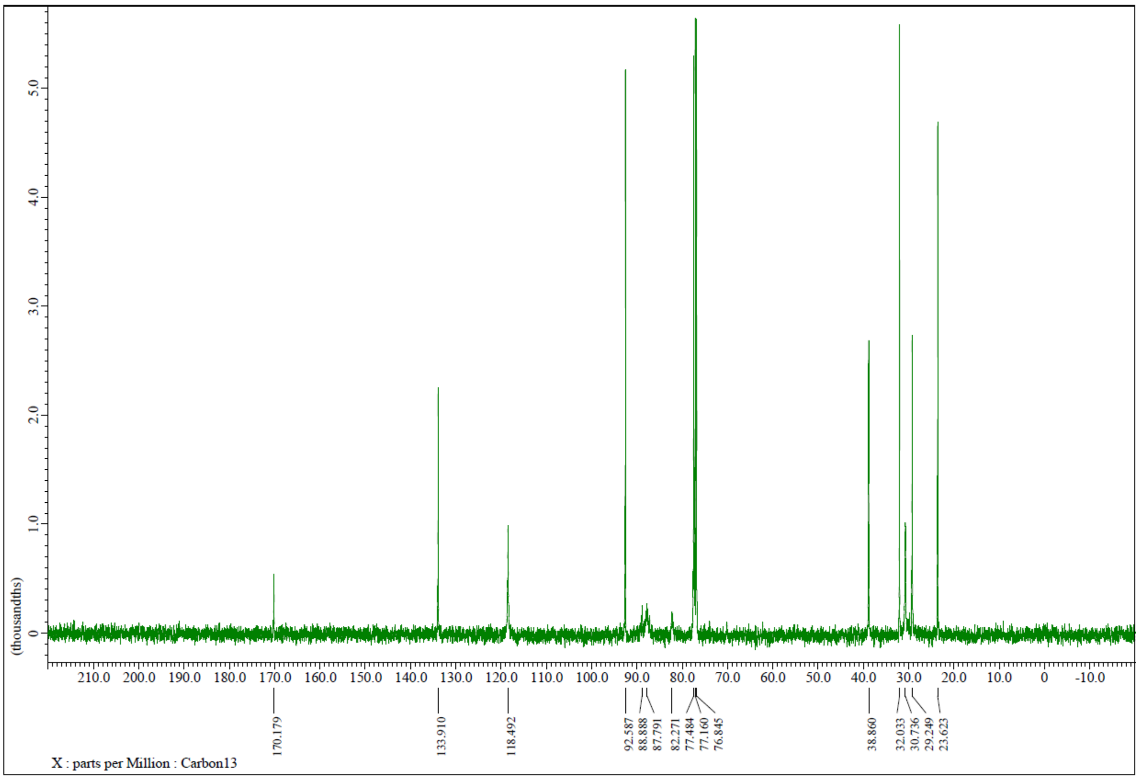

(COSY, 400 MHz, $CDCl_3$)

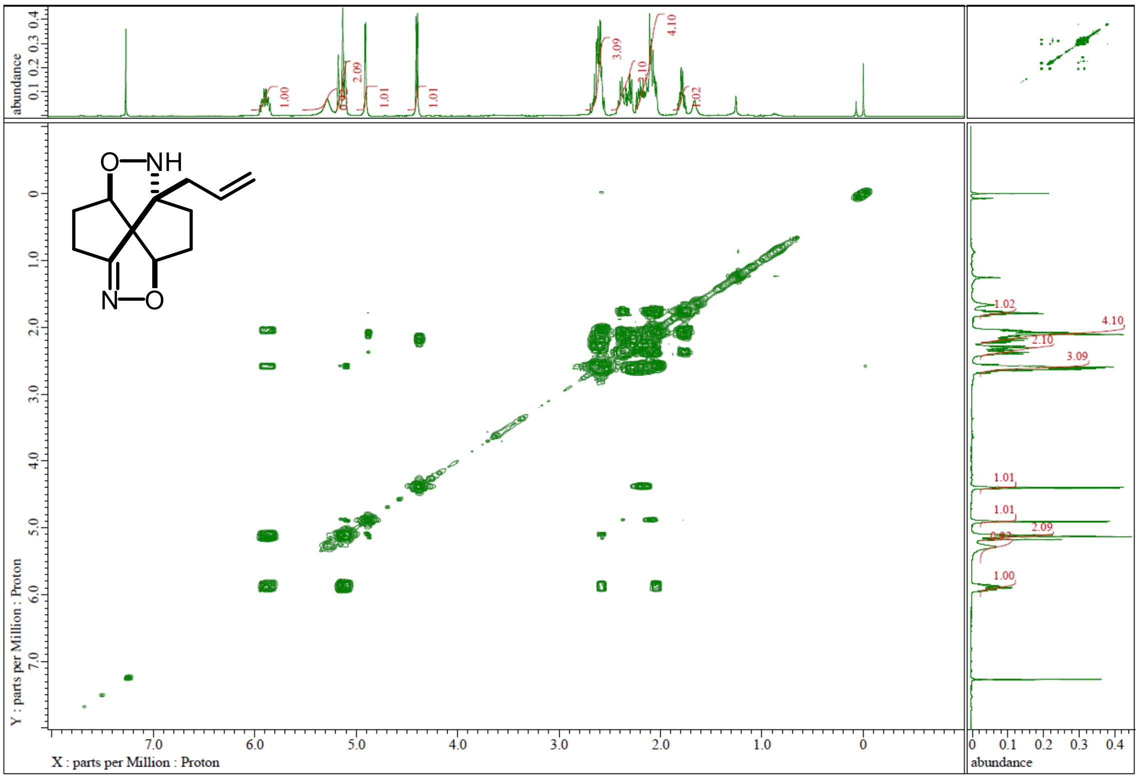

(NOESY, 400 MHz, $CDCl_3$)

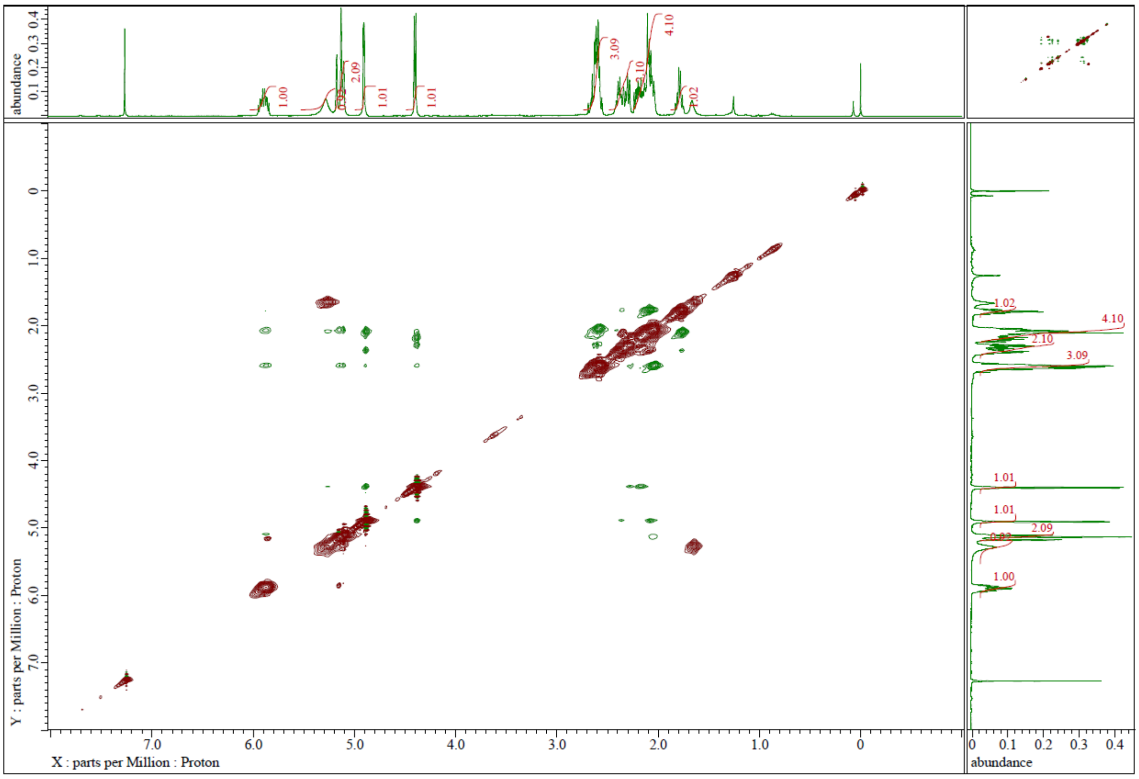

***c,c,c,c*-[5.5.5.5]-3,9-Dioxa-2,8-diaza-1,7-di(2-propenyl)fenestrane (9b)**

(^{1}H NMR, 400 MHz, CDCl$_3$)

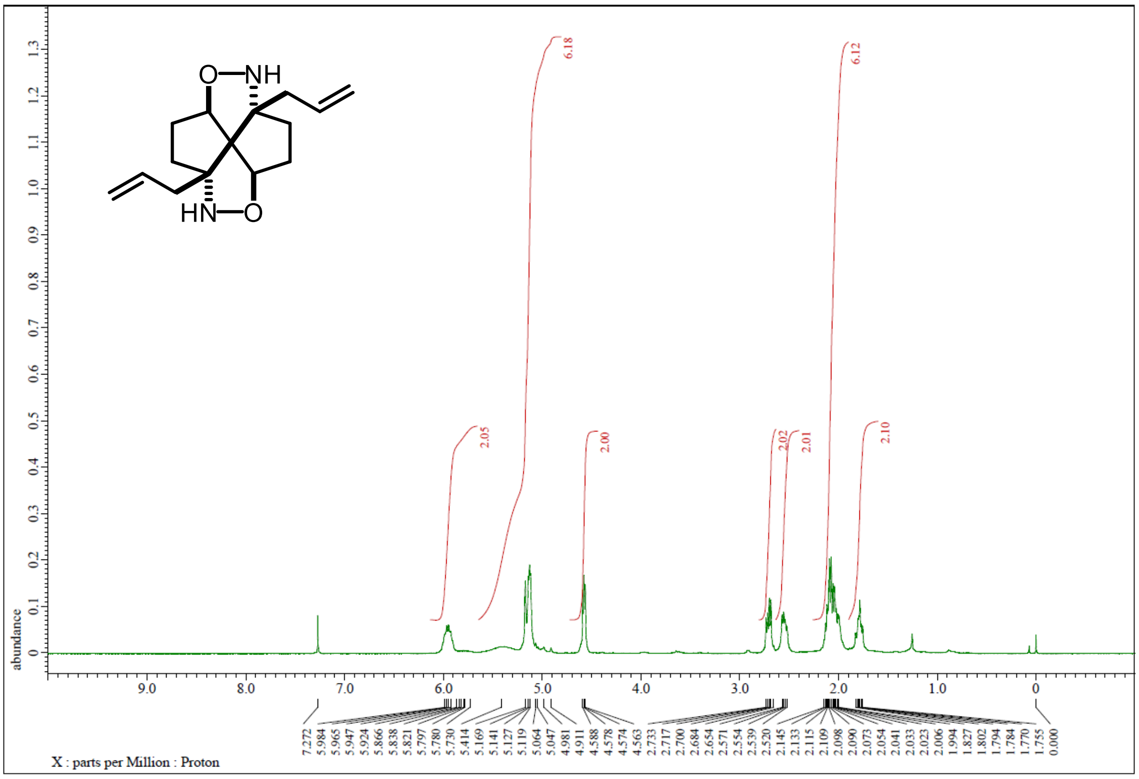

(^{13}C NMR, 100 MHz, CDCl$_3$)

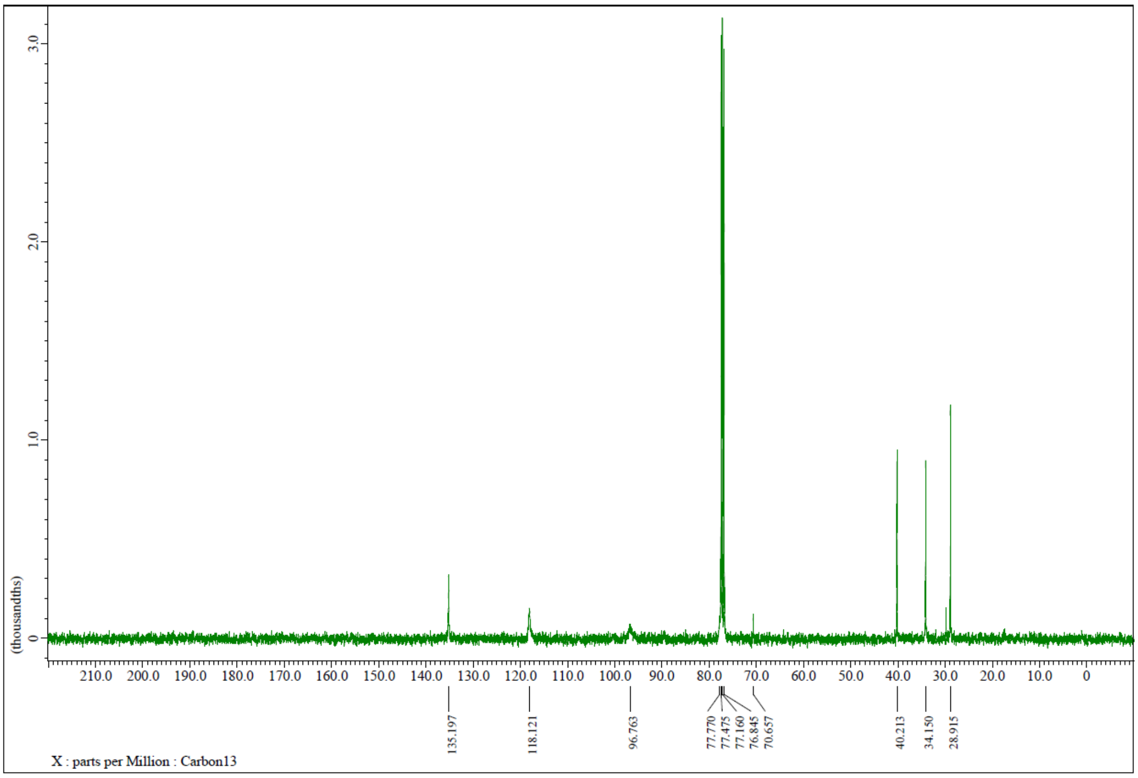

(COSY, 400 MHz, $CDCl_3$)

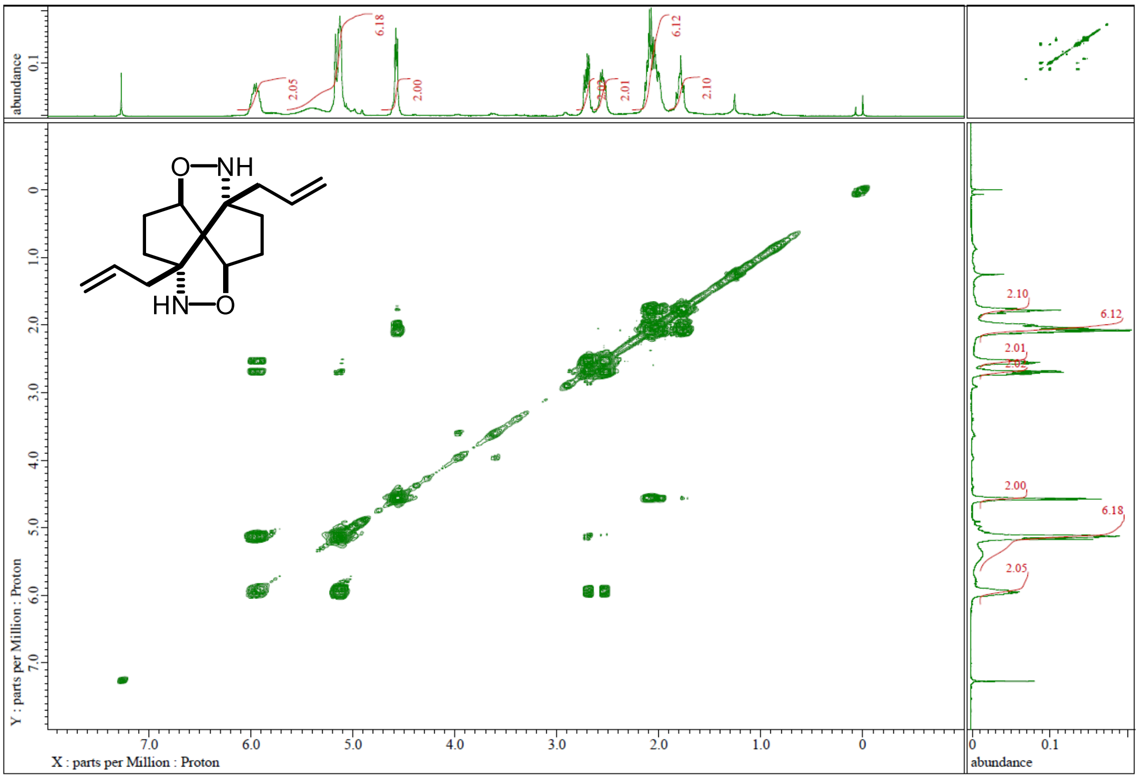

(NOESY, 400 MHz, $CDCl_3$)

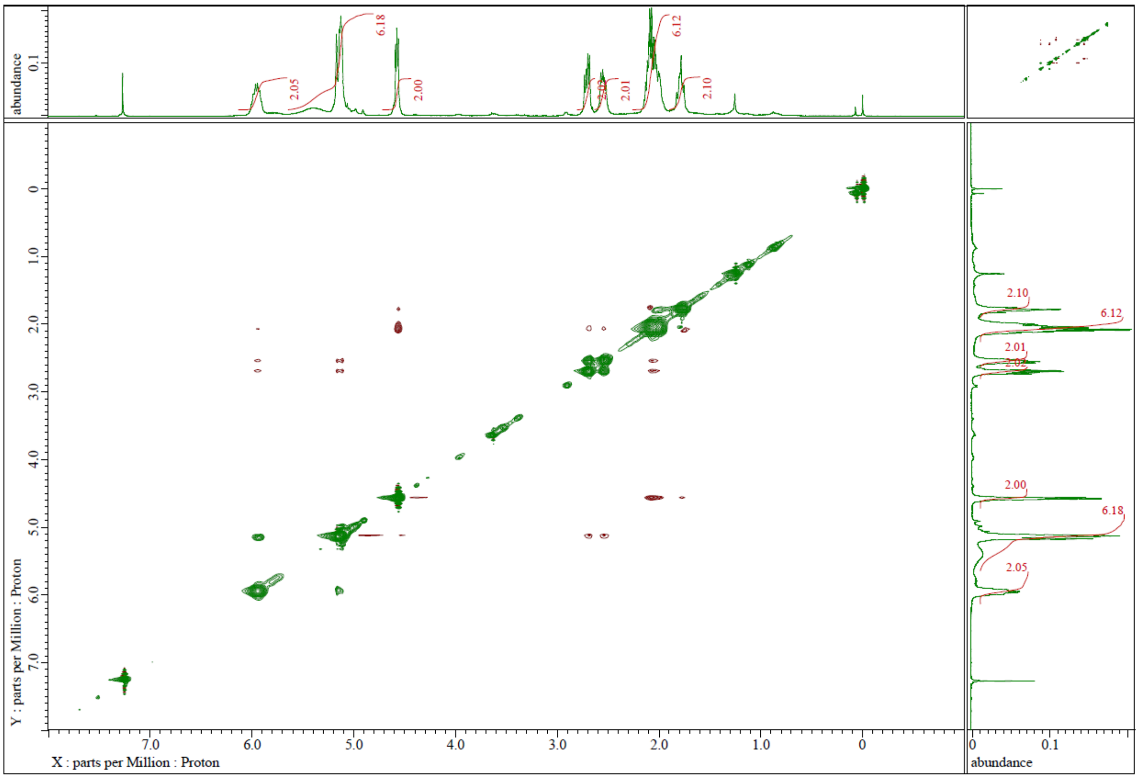

14. References

1. Rosell, M., Villa, M., Durand, T., Galano, J., Vercauteren, J., & Crauste, C. Total syntheses of two bis-allylic-deuterated DHA analogues. *Asian J. Org. Chem.* **6**, 322-334 (2017).
2. Zhang, J., Li, D., Chen, H., Wang, B., Liu, Z., & Zhang, Y. Copper(II)/silver(I)-catalyzed sequential alkynylation and annulation of aliphatic amides with alkynyl carboxylic acids: Efficient synthesis of pyrrolidones. *Adv. Synth. Catal.* **358**, 792-807 (2016).
3. Podunavac, M., Mailyan, A. K., Jackson, J. J., Lovy, A., Farias, P., Huerta, H., Molgó, J., Cardenas, C., & Zakarian, A. Scalable total synthesis, IP3R inhibitory activity of desmethylxestospongin B, and effect on mitochondrial function and cancer cell survival. *Angew. Chem. Int. Ed.* **60**, 11278-11282 (2021).
4. Kita, Y., Okunaka, R., Honda, T., Shindo, M., Taniguchi, M., Kondo, M., & Sasho, M. Strong base-induced intramolecular cycloaddition of homophthalic anhydrides: an efficient synthesis of polycyclic peri-hydroxy aromatic compounds. *J. Org. Chem.* **56**, 119-125 (1991).
5. Marino, J. P., & Nguyen, H. N. Electrotelluration: A new approach to tri- and tetrasubstituted alkenes *J. Org. Chem.* **67**, 6291-6296 (2002).
6. Yadav, J. S., & Sengupta, S. The formal total synthesis of FR252921 –An Immunosuppressant. *Eur. J. Org. Chem.* 376-388 (2013).
7. Yang, Y., Lu, Z., & Xu, X. Phenacyl xanthates: A photoremovable protecting group for alcohols under visible light. *Asian J. Org. Chem.* **8**, 2192-2195 (2019).
8. Green, R. A., Jolley, K. E., Al-Hadedi, A. A. M., Pletcher, D., Harrowven, D. C., Frutos, O. D., Mateos, C., Klauber, D. J., Rincón, J. A., & Brown, R. C. D. Electrochemical deprotection of *para*-methoxybenzyl ethers in a flow electrolysis cell. *Org. Lett.* **19**, 2050-2053 (2017).
9. Singh, P. & Panda, G. Linearization of carbohydrate derived polycyclic frameworks. *RSC Adv.* **4**, 31892-31903 (2014).
10. Sun, H., Gong, L., Tian, Y., Wu, J., Zhang, X., Liu, J., Fu, Z., & Niu, D. Metal- and base-free room-temperature amination of organoboronic acids with *N*-alkyl hydroxylamines. *Angew. Chem. Int. Ed.* **57**, 9456-9460 (2018).
11. Wang, H., Yang, J. C., & Buchwald, S. L. CuH-catalyzed regioselective intramolecular hydroamination for the synthesis of alkyl-substituted chiral aziridines. *J. Am. Chem. Soc.* **139**, 8428-8431 (2017).
12. Molander, G. A. Reductions with samarium(II) iodide. *Org. React.* **46**, 211-367 (1994).
13. Tokuyama, H., Kuboyama, T., Amano, A., Yamashita, T., & Fukuyama, T. A novel transformation of primary amines to N-monoalkylhydroxylamines. *Synthesis* **9**, 1299-1304 (2000).

14. Bode, J. W., Fox, R. M., & Baucom, K. D. Chemoselective amide ligations by decarboxylative condensations of *N*-alkylhydroxylamines and α-ketoacids. *Angew. Chem. Int. Ed.* **45**, 1248-1252 (2006).
15. Lüning, U., Kühl, C., & Uphoff, A. Four hydrogen bonds −DDAA, DADA, DAAD and ADDA hydrogen bond motifs. *Eur. J. Org. Chem.* 4063-4070 (2002).
16. Huang, K. S., Lee, E. H., Olmstead, M. M., & Kurth, M. J. Sequential 1,3-dipolar cycloadditions in the synthesis of bis-isoxazolo substituted piperidinones. *J. Org. Chem*. **65**, 499-503 (2000).
17. Frisch, M. J., Trucks, G. W., Schlegel, H. B., Scuseria, G. E., Robb, M. A., Cheeseman, J. R., Scalmani, G., Barone, V., Petersson, G. A., Nakatsuji, H., Li, X., Caricato, M., Marenich, A. V., Bloino, J., Janesko, B. G., Gomperts, R., Mennucci, B., Hratchian, H. P., Ortiz, J. V., Izmaylov, A. F., Sonnenberg, J. L., Williams-Young, D., Ding, F., Lipparini, F., Egidi, F., Goings, J., Peng, B., Petrone, A., Henderson, T., Ranasinghe, D., Zakrzewski, V. G., Gao, J., Rega, N., Zheng, G., Liang, W., Hada, M., Ehara, M., Toyota, K., Fukuda, R., Hasegawa, J., Ishida, M., Nakajima, T., Honda, Y., Kitao, O., Nakai, H., Vreven, T., Throssell, K., Montgomery, J. A. Jr., Peralta, J. E., Ogliaro, F., Bearpark, M. J., Heyd, J. J., Brothers, E. N., Kudin, K. N., Staroverov, V. N., Keith, T. A., Kobayashi, R., Normand, J., Raghavachari, K., Rendell, A. P., Burant, J. C., Iyengar, S. S., Tomasi, J., Cossi, M., Millam, J. M., Klene, M., Adamo, C., Cammi, R., Ochterski, J. W., Martin, R. L., Morokuma, K., Farkas, O., Foresman, J. B., & Fox, D. J. Gaussian 16, Revision B.01, Gaussian, Inc., Wallingford CT, (2016).
18. Lee, C., Yang, W., & Parr, R. G. Development of the Colle-Salvetti correlation-energy formula into a functional of the electron density *Phys. Rev. B* **1988**, **37**, 785-789.
19. Ditchfield, R., Hehre, W. J., & Pople, J. A. Self-consistent molecular-orbital methods. IX. An extended Gaussian-type basis for molecular-orbital studies of organic molecules. *J. Chem. Phys.* **54**, 724-728 (1971).
20. Hehre, W. J., Ditchfield, R., & Pople, J. A. Self—consistent molecular orbital methods. XII. Further extensions of Gaussian—type basis sets for use in molecular orbital studies of organic molecules *J. Chem. Phys*. **56**, 2257-2261 (1972).
21. Hariharan, P. C. & Pople, J. A. The influence of polarization functions on molecular orbital hydrogenation energies. *Theoret. chim. Acta (Berl.)* **28**, 213-222 (1973).
22. Clark, T., Chandrasekhar, J., Spitznagel, G. W., & Schleyer, P. v. R. Efficient diffuse function-augmented basis sets for anion calculations. III. The 3-21+G basis set for first-row elements, Li–F. *J. Comp. Chem.* **4**, 294-301 (1983).
23. Cancès, E., Mennucci, B., & Tomasi, J. A new integral equation formalism for the polarizable continuum model: Theoretical background and applications to isotropic and anisotropic dielectrics. *J. Chem. Phys.* **107**, 3032-3041 (1997).

24. Marenich, A. V., Cramer, C. J., & Truhlar, D. G. Universal solvation model based on solute electron density and on a continuum model of the solvent defined by the bulk dielectric constant and atomic surface tensions. *J. Phys. Chem. B* **113**, 6378-6396 (2009).
25. Keese, R. Carbon flatland: Planar tetracoordinate carbon and fenestranes. *Chem. Rev.* **106**, 4787-4808 (2006).
